# Reassessing the risk-modifying effects of novel antidiabetic agents on asthma–COPD overlap syndrome: a dose-stratified network meta-analysis of 316,832 adults from 128 randomised trials

**DOI:** 10.1016/j.eclinm.2026.104026

**Published:** 2026-06-19

**Authors:** Bing-Yan Zeng, Chih-Wei Hsu, Chao-Ming Hung, Wei-Chieh Yang, Brendon Stubbs, Yen-Wen Chen, Wei-Te Lei, Jiann-Jy Chen, Tien-Yu Chen, Shih-Pin Hsu, Hung-Yu Wang, Yow-Ling Shiue, Bing-Syuan Zeng, Cheng-Ta Li, Kuan-Pin Su, Chih-Sung Liang, Mein-Woei Suen, Ping-Tao Tseng

**Affiliations:** a Institute of Biomedical Sciences, National Sun Yat-sen University, Kaohsiung, Taiwan; b Department of Internal Medicine, E-Da Dachang Hospital, I-Shou University, Kaohsiung, Taiwan; c Department of Psychiatry, Kaohsiung Chang Gung Memorial Hospital and Chang Gung University College of Medicine, Kaohsiung, Taiwan; d Division of General Surgery, Department of Surgery, E-Da Cancer Hospital, I-Shou University, Kaohsiung, Taiwan; e School of Medicine, College of Medicine, I-Shou University, Kaohsiung, Taiwan; f Department of Pediatrics, Ping An Medical Clinic, Tainan, Taiwan; g Psychological Medicine, Institute of Psychiatry, Psychology and Neuroscience (IoPPN), King's College London, London, UK; h Comprehensive Centre for Clinical Neurosciences and Mental Health (C3NMH), Medical University of Vienna, Vienna, Austria; i Clinical Division of Social Psychiatry, Department of Psychiatry and Psychotherapy, Medical University of Vienna, Vienna, Austria; j Division of Psychology and Mental Health, Manchester Academic Health Science Centre, University of Manchester, Manchester, UK; k Prospect Clinic for Otorhinolaryngology & Neurology, Kaohsiung, Taiwan; l Division of Pediatric Allergy, Immunology, and Rheumatology, Department of Pediatrics, Hsinchu Municipal MacKay Children's Hospital, Hsinchu City, Taiwan; m Graduate Institute of Clinical Medical Sciences, College of Medicine, Chang Gung University, Taoyuan City, Taiwan; n Department of internal medicine, E-Da Cancer Hospital, I-Shou University, Kaohsiung, Taiwan; o Department of Psychiatry, Tri-Service General Hospital, Taipei, Taiwan; p Department of Psychiatry, College of Medicine, National Defense Medical University, Taipei, Taiwan; q Department of Neurology, E-Da hospital, I-Shou University, Kaohsiung, Taiwan; r Kaohsiung Municipal Kai-Syuan Psychiatric Hospital, Kaohsiung City, Taiwan; s College of Semiconductor and Advanced Technology Research, National Sun Yat-sen University Kaohsiung, Taiwan; t Institute of Precision Medicine, National Sun Yat-sen University, Kaohsiung City, Taiwan; u Department of Psychiatry, Taipei Veterans General Hospital, Taipei, Taiwan; v Division of Psychiatry, School of Medicine, National Yang Ming Chiao Tung University, Taipei 112, Taiwan; w Institute of Brain Science and Brain Research Center, School of Medicine, National Yang Ming Chiao Tung University, Taipei 112, Taiwan; x Office of Research and Development, Asia University, Taichung, Taiwan; y College of Medicine, China Medical University, Taichung, Taiwan; z An-Nan Hospital, China Medical University, Tainan, Taiwan; aa Mind-Body Interface Research Center (MBI Lab & Care), China Medical University Hospital, Taichung, Taiwan; ab Department of Psychiatry, Beitou Branch, Tri-Service General Hospital; School of Medicine, National Defense Medical University, Taipei, Taiwan; ac Department of Psychiatry, National Defense Medical University, Taipei, Taiwan; ad Department of Psychology, College of Medical and Health Science, Asia University, Taichung, Taiwan; ae Gender Equality Education and Research Center, Asia University, Taichung, Taiwan; af Department of Medical Research, Asia University Hospital, Asia University, Taichung, Taiwan; ag Department of Medical Research, China Medical University Hospital, China Medical University, Taichung, Taiwan; ah School of Medicine, College of Medicine, National Sun Yat-sen University, Kaohsiung, Taiwan

**Keywords:** Network meta-analysis, DPP4 inhibitor, GLP-1 receptor agonist, SGLT2 inhibitor, Asthma, COPD, Asthma-COPD overlap syndrome

## Abstract

**Background:**

Asthma–chronic obstructive pulmonary disease (COPD) overlap syndrome (ACOS) accounts for 15–25% of chronic obstructive airway disease and is linked to frequent exacerbations and excess mortality. Newer glucose-lowering drugs may affect respiratory outcomes, but agent-level and dose-specific effects on ACOS are uncertain.

**Methods:**

We searched PubMed, Embase, Cochrane CENTRAL, Web of Science, ClinicalTrials.gov, ClinicalKey, ScienceDirect, and ProQuest from inception to April 03, 2026, with an initial search on Dec 12, 2024. Eligible studies were randomised controlled trials in adult participants receiving eligible glucose-lowering therapies and systematically recording ACOS-related, asthma, or COPD events during follow-up. Trials compared dipeptidyl peptidase-4 (DPP-4) inhibitors, glucagon-like peptide-1 (GLP-1) receptor agonists, sodium–glucose cotransporter 2 (SGLT2) inhibitors, and other eligible antidiabetic regimens against standard care and/or placebo control. Risk ratios (RRs) with 95% CIs were estimated relative to this control group for ACOS, asthma, and COPD outcomes. Heterogeneity was assessed using tau-squared and *I*^*2*^ statistics, and small-study effects/publication bias were assessed using comparison-adjusted funnel plots and Egger's regression. Outcome was trial-reported ACOS-related respiratory events. This study is registered with PROSPERO, CRD42024626613.

**Findings:**

Canagliflozin (RR 0.62, 95% CI 0.40–0.97), empagliflozin (0.70, 0.51–0.95), dapagliflozin (0.76, 0.63–0.92), and injectable semaglutide (0.64, 0.49–0.84) were associated with lower ACOS risk than control. Dose-stratified analyses suggested stronger associations for selected regimens, with signals more evident in participants with diabetes. Dapagliflozin was associated with lower asthma risk, whereas canagliflozin, empagliflozin, and injectable semaglutide were associated with lower COPD risk. Saxagliptin was associated with higher asthma risk (2.09, 1.01–4.33). No major heterogeneity, inconsistency, or small-study effects were detected.

**Interpretation:**

Respiratory associations of newer glucose-lowering therapies were heterogeneous and agent specific. Selected SGLT2 inhibitors and injectable semaglutide were associated with lower ACOS-related risk, whereas saxagliptin may warrant caution in people prone to asthma. These findings support further prospective evaluation.

**Funding:**

Taiwan National Science and Technology Council.

**Research in context**

**Evidence before this study**

We searched PubMed, Embase, Cochrane CENTRAL, Web of Science, ClinicalTrials.gov, ClinicalKey, ScienceDirect, and ProQuest from inception to April 03, 2026, with an initial search on Dec 12, 2024. We used combinations of terms related to “type 2 diabetes,” “glucose-lowering drugs,” “GLP-1 receptor agonist,” “SGLT2 inhibitor,” “DPP-4 inhibitor,” “asthma,” “COPD,” “overlap,” and “asthma–COPD overlap syndrome.” We screened reference lists of relevant randomised trials, systematic reviews, and meta-analyses. We included randomised controlled trials that prospectively recorded respiratory adverse events and reported incident asthma, chronic obstructive pulmonary disease (COPD), or asthma–COPD overlap syndrome (ACOS) in adults without those diagnoses at baseline. Most prior quantitative syntheses used traditional pairwise meta-analysis to study individual drug classes and typically evaluated asthma or COPD separately, rather than ACOS as an integrated phenotype. These analyses generally suggested no clear preventive effect of GLP-1 receptor agonists on asthma or COPD and a class-level reduction in ACOS risk with SGLT2 inhibitors, but they did not differentiate individual agents, doses, or diabetes status, and they did not systematically evaluate DPP-4 inhibitors in this context. No prior study provided a comprehensive, agent-specific, dose-stratified network meta-analysis of newer antidiabetic therapies on ACOS risk. Overall evidence quality was limited by heterogeneous outcome definitions, small numbers of ACOS events, and lack of drug-level comparisons.

**Added value of this study**

This study brings together data from 128 randomised trials and 316,832 adults into a single, agent- and dose-resolved network meta-analysis focused on ACOS and its component conditions. By simultaneously modelling ACOS, asthma, and COPD events, and stratifying by individual drug, dose, and diabetes status, we were able to show that only a subset of newer glucose-lowering agents—canagliflozin, empagliflozin, dapagliflozin, and injectable semaglutide—are associated with reduced ACOS risk, with clear dose–response patterns and stronger protection among people with diabetes. We also identify saxagliptin as a potential asthma-promoting agent, a safety signal not previously highlighted at the network level. Beyond class-level comparisons, this work provides randomised comparative evidence at the agent level.

**Implications of all the available evidence**

Taken together with existing literature, our findings indicate that respiratory effects of newer antidiabetic agents are heterogeneous within as well as between drug classes. Clinically, they argue against treating all SGLT2 inhibitors or GLP-1 receptor agonists as interchangeable for patients at risk of ACOS and instead support preferential use of canagliflozin, empagliflozin, dapagliflozin, or injectable semaglutide when respiratory prevention is a priority, while exercising caution with saxagliptin in individuals prone to asthma. For policy and guideline makers, these data reinforce the need for drug-level recommendations rather than broad class labels when managing multimorbidity at the interface of metabolic and respiratory medicine. For future research, they highlight the importance of prospective trials and pragmatic real-world studies that prespecify ACOS and airway outcomes, as well as mechanistic work to explain the divergent pulmonary profiles of individual agents. Collectively, the available evidence supports a move toward more personalised, data-driven selection of glucose-lowering therapy to optimise both cardiometabolic and respiratory health.

## Introduction

Recent advances in pharmacotherapy have uncovered unexpected systemic benefits of novel antidiabetic medications beyond glycaemic control. Notably, dipeptidyl peptidase-4 (DPP-4) inhibitors, glucagon-like peptide-1 (GLP-1) receptor agonists, and sodium–glucose cotransporter 2 (SGLT2) inhibitors, originally developed for the management of type 2 diabetes mellitus, have demonstrated potential in modulating inflammatory and metabolic pathways implicated in pulmonary disorders.^1^^,^^2^ Given the established link between metabolic syndromes such as diabetes and increased susceptibility to chronic respiratory diseases,^3^ the possibility of leveraging antidiabetic agents to reduce pulmonary disease burden has generated considerable clinical interest.

Among respiratory conditions, asthma–chronic obstructive pulmonary disease (COPD) overlap syndrome (ACOS) is increasingly recognised as a distinct phenotype associated with heightened symptom severity, exacerbation frequency, and mortality compared to asthma or COPD alone.^3^ Individuals with type 2 diabetes or obesity, the primary recipients of antidiabetic therapy, frequently present with overlapping pulmonary vulnerabilities, underscoring the importance of evaluating respiratory outcomes within this population. However, traditional randomised trials often lack the statistical power or targeted design to evaluate adverse events such as incident ACOS. In such scenarios, meta-analytic approaches serve as a critical bridge, synthesising data across multiple trials to illuminate clinically meaningful associations.^4^^,^^5^

Previous meta-analyses have yielded inconclusive or conflicting results. Some pairwise analyses found no significant benefit of GLP-1 receptor agonists in preventing either asthma^6^ or COPD,^7^ while others reported an overall reduction in ACOS risk with SGLT2 inhibitors as a drug class.^8^, ^9^, ^10^ However, these analyses did not distinguish between individual agents nor assess dose-dependent effects, limiting clinical applicability. Furthermore, pooling heterogeneous regimens within a pharmacological class may mask drug-specific differences.

Network meta-analysis (NMA) offers a methodological advantage in this context. By enabling direct and indirect comparisons across multiple interventions, NMAs can provide a hierarchical assessment of treatment efficacy and safety with greater granularity.^11^ Our research group has previously applied this technique to evaluate extra-glycaemic outcomes of antidiabetic agents across a variety of conditions, including neurodegenerative diseases,^12^ metastatic malignancies,^13^ colorectal cancer,^14^ gynaecologic cancer,^15^ haematologic malignancy,^16^ and auditory–vestibular dysfunction.^17^

In the present study, we conducted a comprehensive NMA to systematically examine the impact of novel antidiabetic agents on the risk of developing ACOS. By stratifying agents by class, individual compound, dosage, and population subgroup, we aimed to clarify whether certain medications may offer respiratory protection—and if so, to what extent such benefits are specific to asthma, COPD, or their overlap.

## Methods

### Study design and registration

This network meta-analysis (NMA) was performed following a confirmatory analytical framework in line with Cochrane recommendations for investigating prespecified adverse outcomes of pharmacologic interventions.^18^ The reporting of this study adheres to the PRISMA extension statement for network meta-analyses (PRISMA-NMA). The protocol was prospectively registered in PROSPERO (CRD42024626613).

### Data sources and search strategy

We conducted a systematic literature search across eight databases: PubMed, Embase, Cochrane CENTRAL, Web of Science, ClinicalTrials.gov, ClinicalKey, ScienceDirect, and ProQuest, from database inception to April 03, 2026. The initial search was performed on Dec 12, 2024, and the final updated search was performed on April 03, 2026. The full strategy of literature search had been summarised in eTable S1. Briefly, we included keywords of asthma/COPD combined with the targeted regimens in all eight databases. We applied no language restrictions. Two independent reviewers (PT Tseng and BY Zeng) screened titles, abstracts, and full texts for eligibility. Discrepancies were resolved by consensus or consultation with a third reviewer. To ensure comprehensiveness, we also reviewed reference lists of relevant reviews and prior meta-analyses.

### Inclusion and exclusion criteria

The study was designed based on a predefined PICOS framework:

• Population: adult participants in randomised controlled trials
• Intervention: Exposure to any antidiabetic agent under evaluation
• Comparator: standard care and/or placebo control, according to the original trial design. In the included trials, some control groups received standard care plus placebo, whereas others received standard care alone. The specific components of standard care were variably reported across trials and were often not described in sufficient detail to permit consistent classification (for example, metformin, sulfonylureas, or insulin).
• Outcomes: Newly diagnosed ACOS, asthma, or COPD events during trial follow-up
• Study design: Randomised controlled trials (RCTs) only

Although the detailed components of background standard care were often unavailable, standard care was typically part of the original trial design and was not intended to differ systematically between randomised arms within the same study. Eligible agents included DPP-4 inhibitors, SGLT2 inhibitors, and GLP-1 receptor acting agents [including GLP-1 receptor agonists, dual glucose-dependent insulinotropic polypeptide (GIP)/GLP-1 agonists (e.g., tirzepatide), and triple GIP/GLP-1/glucagon receptor agonists (e.g., retatrutide)].

Inclusion criteria were as follows: (1) randomised controlled trials in adult human participants; (2) trials involving the eligible glucose-lowering regimens specified above; and (3) trials systematically recording respiratory adverse events or specifically reporting ACOS-related, asthma, or COPD events during follow-up. Exclusion criteria were as follows: (1) studies lacking systematic recording of respiratory events; (2) studies comparing non-target interventions; (3) animal or non-randomised studies; or (4) studies with major baseline imbalances (e.g., age, sex, comorbidities, or background therapy).

### Risk of bias assessment

Two reviewers independently evaluated methodological quality using the Cochrane Risk of Bias 2.0 tool. Disagreements were adjudicated by a third investigator.

### Outcome definitions

The primary outcome was reported asthma–COPD overlap syndrome (ACOS)-related events identified during trial follow-up from the terminology used in the original trial publications, Supplementary Appendices, and, where available, regulatory materials. Because the included randomised trials were not designed to prospectively adjudicate asthma–COPD overlap as a prespecified endpoint, a uniform diagnostic definition was not available across studies. Accordingly, the primary analysis should be interpreted as reflecting reported ACOS-related events rather than uniformly adjudicated incident ACOS defined by a single standardised clinical criterion. In current practice, asthma–COPD overlap syndrome was generally used to describe patients with chronic airflow obstruction who also show asthmatic features, such as a prior history of asthma, marked bronchodilator reversibility, or eosinophilic inflammation, although thresholds and algorithms vary across statements.^19^, ^20^, ^21^ Asthma cases due to cardiac or other secondary causes were excluded. Where trial reports did not provide detailed diagnostic criteria, outcomes were classified according to the reported investigator- or registry-level terminology, and no attempt was made to retrospectively impose a single external definition across all studies. Secondary endpoints included asthma severity (e.g., status asthmaticus) and COPD subtypes (e.g., emphysema, chronic bronchitis). Acceptability was assessed through all-cause treatment discontinuation rates.

### Sensitivity analysis and subgroup stratifications

In order to explore the potentially confounding effects related to dosage, baseline diseases, treatment duration, age, and sex, we arrange subgroup analyses based on dose-stratification, baseline disease stratification (i.e. diabetes mellitus), treatment duration stratification (i.e. at least 1 year), age stratification (i.e. at least 65 years old or younger than 65 years old), and sex stratification (i.e. male predominance or female predominance). Dose categories were defined within each drug according to the original trial protocols, which were not intended to imply pharmacologic equivalence across different agents (eTable S2). We choose baseline diseases with diabetes mellitus because this disease was the major indication of those medications. The “1 year treatment duration” was chosen to represent the cut-off point in our study for treatment duration stratification based on the frequently recommended period for long term treatment or follow-up in guideline or large-scale trial.^22^ We choose 65 years old to be our cut-off points of age stratification because it was a world widely accepted age cut-off point.^23^ We recognised the fact that it would be difficult to retrieve the detailed data of asthma-COPD overlap syndrome in pure male or female groups. Therefore, we choose to stratify subgroup by male predominance (i.e. male proportion >50%) and female predominance (i.e. female proportion >50%). Formal interaction tests for whether treatment effects differed across subgroup factors were not performed within our current STATA-based network meta-analysis framework; subgroup findings were therefore interpreted descriptively. Post-hoc analyses added during peer review included treatment-duration stratification, hazard ratio-based sensitivity analysis using time-to-event data when available, and trial-level meta-regression by treatment duration.

We performed sensitivity analysis using NMA based on hazard ratio (HR) estimates derived from time-to-event data, when available, because this approach can retain event-time and censoring information.^24^

### Data extraction and handling

Two authors (PT Tseng and BY Zeng) independently extracted trial characteristics, population demographics, intervention protocols, primary and secondary outcomes, and safety data. Regarding data extraction for main outcomes, we intended to extract original data (i.e. numbers of participants reported asthma/COPD) but not the calculated data (i.e. odds ratio or risk ratio data) if available. Missing values were requested from corresponding authors when feasible. Extraction was based on Cochrane Handbook protocols.

### Statistical analysis

We performed a frequentist random-effects NMA using STATA (version 16.0, StataCorp, College Station, TX).^25^ This network meta-analysis was conducted using generalised linear mixed models within a mixed-treatment comparison framework to integrate both direct and indirect evidence. Indirect estimates were derived under the transitivity assumption, whereby the relative effect between treatments A and B could be inferred through their respective comparisons with a common comparator, C. When studies included more than two intervention arms, direct and indirect data were synthesised jointly across the network. Between-study variance was estimated with the restricted maximum likelihood approach.^26^ Treatment comparisons were expressed as risk ratios (RRs) with corresponding 95% confidence intervals (95%CIs). Surface under the cumulative ranking (SUCRA) probabilities were computed to rank interventions by efficacy.

Between-study heterogeneity was quantified using τ^2^ and *I*^*2*^ statistics, where applicable. Global and local inconsistencies were assessed via loop-specific estimates, node-splitting models, and the design-by-treatment interaction approach. The certainty of evidence was graded using GRADE methodology adapted for NMA. Publication bias was examined through Egger's regression and small study effects were examined via comparison-adjusted funnel plots. We also performed trial-level meta-regression to examine whether treatment duration was associated with the primary outcome.

### Ethics

The study protocol was submitted to the Institutional Review Board of the Tri-Service General Hospital, National Defence Medical Centre, which determined that this study qualified for exempt review because it was a systematic review and network meta-analysis of published aggregate data and did not involve direct participation of human participants or identifiable personal data (TSGHIRB E202516007; determination date Feb 10, 2025). Written informed consent was therefore not required.

### Role of the funding source

The funders had no involvement in study design, data collection, data analyses, data interpretation, or the writing of the report.

## Results

### Eligibility of the studies

**Fig. 1:**
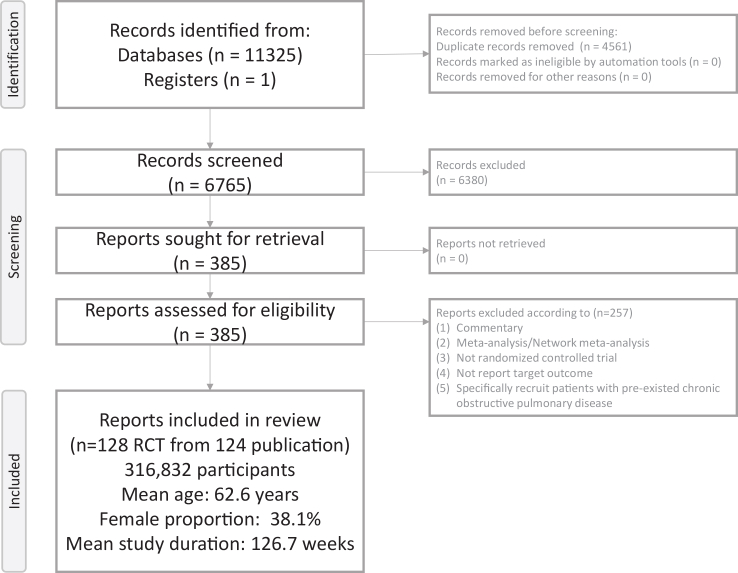
**PRISMA2020 Flowchart of current network meta-analysis.** Abbreviation: PRISMA: preferred reporting items for systematic reviews and meta-analyses; RCT: randomised controlled trial.

The systematic screening process is summarised in Fig. 1. Of 385 initially eligible articles, 257 were excluded during full-text assessment (eTable S3). Ultimately, 128 randomised controlled trials (from 124 publications) comprising 316,832 participants were included in the NMA.^27^, ^28^, ^29^, ^30^, ^31^, ^32^, ^33^, ^34^, ^35^, ^36^, ^37^, ^38^, ^39^, ^40^, ^41^, ^42^, ^43^, ^44^, ^45^, ^46^, ^47^, ^48^, ^49^, ^50^, ^51^, ^52^, ^53^, ^54^, ^55^, ^56^, ^57^, ^58^, ^59^, ^60^, ^61^, ^62^, ^63^, ^64^, ^65^, ^66^, ^67^, ^68^, ^69^, ^70^, ^71^, ^72^, ^73^, ^74^, ^75^, ^76^, ^77^, ^78^, ^79^, ^80^, ^81^, ^82^, ^83^, ^84^, ^85^, ^86^, ^87^, ^88^, ^89^, ^90^, ^91^, ^92^, ^93^, ^94^, ^95^, ^96^, ^97^, ^98^, ^99^, ^100^, ^101^, ^102^, ^103^, ^104^, ^105^, ^106^, ^107^, ^108^, ^109^, ^110^, ^111^, ^112^, ^113^, ^114^, ^115^, ^116^, ^117^, ^118^, ^119^, ^120^, ^121^, ^122^, ^123^, ^124^, ^125^, ^126^, ^127^, ^128^, ^129^, ^130^, ^131^, ^132^, ^133^, ^134^, ^135^, ^136^, ^137^, ^138^, ^139^, ^140^, ^141^, ^142^, ^143^, ^144^, ^145^, ^146^, ^147^, ^148^, ^149^, ^150^ A summary of the included trial characteristics is shown in Table 1, and full study-level details are provided in eTable S4. The mean age was 62.6 years (range 44.9–73.5), with women representing 38.1% of participants (range 21.3–78.5%). The average duration of follow-up was 126.7 weeks (range 4–338 weeks).

**Table 1:** Summary characteristics of the included randomised controlled trials.

Characteristic	Summary of included trials
Study design	128 randomised controlled trials were included in the network meta-analysis.
Total participants	316,832
Underlying populations	Most trials enrolled adults with type 2 diabetes mellitus. Additional populations included obesity, heart failure, chronic kidney disease, cardiovascular disease, and related metabolic or cardiorenal conditions.
Eligible interventions	DPP-4 inhibitors, SGLT2 inhibitors, and GLP-1 receptor acting agents, including dual GIP/GLP-1 agonists and triple GIP/GLP-1/glucagon receptor agonists where relevant.
Comparator structure	Standard care with or without placebo, according to the original trial design. Some studies used standard care plus placebo, whereas others used standard care alone.
Mean age	62.6 years overall (range across studies 44.4–73.9 years)
Female participants	38.1% overall (range across studies 17.5%–81.0%)
Male participants	61.9% overall (range across studies 19.0%–82.5%)
Follow-up duration	Mean 126.7 weeks overall (range across studies 4–338 weeks)
Primary outcome data	Trial-reported asthma-COPD syndrome-related respiratory events were extracted from publications, Supplementary Appendices, and, where available, regulatory sources.
Race and ethnicity reporting	Reporting was variable and non-standardised across trials; quantitative subgroup analysis by race/ethnicity was not feasible.
Geographical setting	Most studies were multinational; additional single-country trials were conducted in countries including Japan, China, Germany, and the USA.
Trial registration	Clinical trial registration identifiers were available for most studies.

Full trial-level characteristics, including individual study citations, baseline illness, intervention and comparator details, sample size, female and male sex distribution, event counts, follow-up duration, ethnicity reporting, registration identifiers, and country information, are provided in eTable S4.

Abbreviation: COPD: chronic obstructive pulmonary disease; DPP-4 inhibitor: dipeptidyl peptidase 4 inhibitor; GIP: glucose-dependent insulinotropic polypeptide; GLP-1 receptor: glucagon-like peptide-1 receptor; SGLT2 inhibitor: sodium–glucose cotransporter 2 inhibitor; USA: United States of America.

The antidiabetic agents analysed included:

• DPP-4 inhibitors: alogliptin, linagliptin, omarigliptin, saxagliptin, sitagliptin, teneligliptin, vildagliptin
• GLP-1 receptor agonists: albiglutide, dulaglutide, efpeglenatide, exenatide, liraglutide, lixisenatide, semaglutide (injectable or oral form), tirzepatide
• SGLT2 inhibitors: bexagliflozin, canagliflozin, dapagliflozin, empagliflozin, ertugliflozin, sotagliflozin
• Other classes: triple agonist (retatrutide), mitochondrial modulator (imeglimin), glucokinase activator (dorzagliatin), amylin analogue (petrelintide), anti-CD3 antibody (teplizumab)

However, several agents, such as imeglimin (mitochondrial modulator), dorzagliatin (glucokinase activator), petrelintide (amylin analogue), and teplizumab (anti-CD3 monoclonal antibody), lacked respiratory outcome data and were excluded from final synthesis.

### Primary outcome: overall ACOS risk

**Fig. 2:**
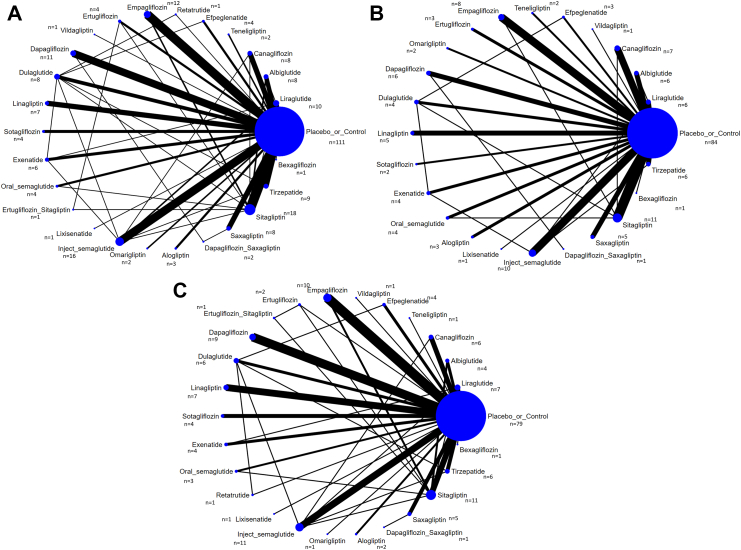
**Network structure of the (A) primary outcome: overall asthma-COPD overlap syndrome risk; (B) secondary outcome: asthma risk; (C) secondary outcome: COPD risk.** Overall structure of the network meta-analysis. The lines between nodes represent direct comparisons in various trials, and the size of each circle is proportional to the number of participants in each specific treatment. The thickness of the lines is proportional to the number of trials connected to the network. Abbreviation: COPD: chronic obstructive pulmonary disease.

**Fig. 3:**
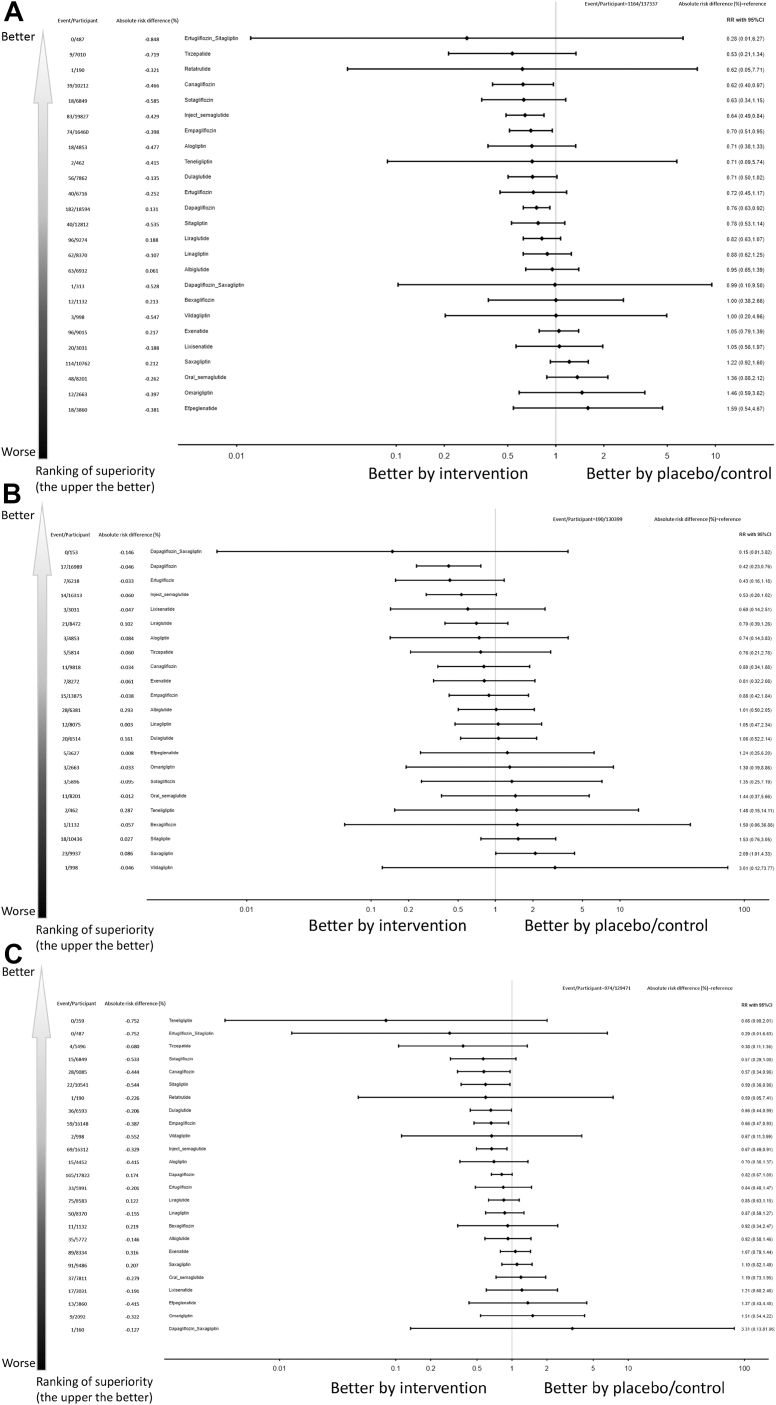
**Forest plot of the (A) primary outcome: overall asthma-COPD overlap syndrome risk; (B) secondary outcome: asthma risk; (C) secondary outcome: COPD risk.** When the effect size (expressed as risk ratio) is less than 1, the specified treatment is associated with fewer risk compared to placebo/controls. Abbreviation: 95%CIs: 95% confidence intervals; COPD: chronic obstructive pulmonary disease; RR: risk ratio.

The main results revealed that canagliflozin (RR = 0.62, 95%CIs = 0.40 to 0.97), injectable semaglutide (RR = 0.64, 95%CIs = 0.49 to 0.84), empagliflozin (RR = 0.70, 95%CIs = 0.51 to 0.95), and dapagliflozin (RR = 0.76, 95%CIs = 0.63 to 0.92) were associated with significantly lower ACOS risk than the controls (Fig. 2A, Fig. 3A, eFigure S3, and eTable S5A). Other regimens showed more favourable or less favourable point estimates to varying degrees, but these estimates were generally less precise and did not consistently exclude the null. Accordingly, the comparative pattern across agents should be interpreted in the context of both effect size and precision.

#### Subgroup analysis

Among agents with significant primary outcome associations, the reduced risk was more prominent at specific doses, including high-dose canagliflozin (300–600 mg/day) (RR = 0.52, 95%CIs = 0.29 to 0.94), high-dose injectable semaglutide (2.0–2.4 mg/week) (RR = 0.62, 95%CIs = 0.45 to 0.86), medium-dose dulaglutide (1.5 mg/week) (RR = 0.69, 95%CIs = 0.48 to 0.98), low-dose empagliflozin (1–10 mg/day) (RR = 0.69, 95%CIs = 0.50 to 0.96), and high-dose dapagliflozin (10 mg/day) (RR = 0.76, 95%CIs = 0.63 to 0.92). These findings indicate that some dose-stratified associations were observed for selected regimens, although these patterns were not uniform across all agents (eFigure S1A and eFigure S2A). Similarly, some other regimens, such as high-dose empagliflozin, showed more favourable point estimates to reduce asthma-COPD overlap syndrome, but this estimate was generally less precise and did not consistently exclude the null.

When we focused on participants with diabetes mellitus, the results of overall ACOS risk revealed that only canagliflozin (RR = 0.64, 95%CIs = 0.41 to 0.99) was still associated with significantly lower asthma-COPD overlap syndrome risk than the controls (eFigure S1B, eFigure S2B, and eTable S5B).

In age-stratification analysis, the results of overall ACOS risk revealed that only injectable semaglutide (RR = 0.63, 95%CIs = 0.48 to 0.85) and dapagliflozin (RR = 0.79, 95%CIs = 0.63 to 0.99) were still associated with significantly lower asthma-COPD overlap syndrome risk than the controls in subgroup of age younger than 65 years old (eFigure S1C, eFigure S2C, and eTable S5C). In contrast, only dulaglutide (RR = 0.65, 95%CIs = 0.43 to 0.99) was still associated with significantly lower asthma-COPD overlap syndrome risk than the controls in subgroup of age at least 65 years old (eFigure S1D and eFigure S2D, and eTable S5D).

In sex-stratification analysis, the results of overall ACOS risk revealed that only canagliflozin (RR = 0.63, 95%CIs = 0.40 to 0.99), injectable semaglutide (RR = 0.65, 95%CIs = 0.49 to 0.86), dulaglutide (RR = 0.66, 95%CIs = 0.45 to 0.95), empagliflozin (RR = 0.70, 95%CIs = 0.51 to 0.95), and dapagliflozin (RR = 0.76, 95%CIs = 0.63 to 0.92) were still associated with significantly lower asthma-COPD overlap syndrome risk than the controls in trials with male predominance (eFigure S1E, eFigure S2E, and eTable S5E). In contrast, none regimens was associated with significantly different asthma-COPD overlap syndrome risk compared to controls in trials with female predominance (eFigure S1F, eFigure S2F, and eTable S5F).

In treatment duration-stratification analysis, the results of overall ACOS risk revealed that only injectable semaglutide (RR = 0.64, 95%CIs = 0.48 to 0.85), empagliflozin (RR = 0.72, 95%CIs = 0.52 to 0.98), and dapagliflozin (RR = 0.77, 95%CIs = 0.63 to 0.95) were still associated with significantly lower asthma-COPD overlap syndrome risk than the controls in trials with treatment duration at least 1 year (eFigure S1G, eFigure S2G, and eTable S5G). However, we could not perform analytic process for subgroup analysis of treatment duration less than 1 year due to insufficient data.

#### Sensitivity analysis

When we analysed HR based on time-to-event data, canagliflozin (HR = 0.62, 95%CIs = 0.40 to 0.97), injectable semaglutide (HR = 0.64, 95%CIs = 0.49 to 0.84), empagliflozin (HR = 0.70, 95%CIs = 0.51 to 0.95), and dapagliflozin (HR = 0.76, 95%CIs = 0.62 to 0.92) were associated with significantly lower ACOS risk than the controls (eFigure S1H, eFigure S2H, and eTable S5H).

#### Secondary outcome: asthma-specific events and COPD-specific events

In our secondary outcome of asthma events, only the dapagliflozin (RR = 0.42, 95%CIs = 0.23 to 0.76) was still associated with significantly decreased risk of asthma compared to control. Among the regimens investigated, the saxagliptin (RR = 2.09, 95%CIs = 1.01 to 4.33) was associated with significantly increased risk of asthma compared to controls (Figs. 2B and 3B, and eTable S5I). Several other regimens had point estimates on either side of the null, but confidence intervals were wider and did not support firm distinctions between agents.

Regarding secondary outcome of COPD events, the canagliflozin (RR = 0.57, 95%CIs = 0.34 to 0.96), sitagliptin (RR = 0.59, 95%CIs = 0.36 to 0.96), dulaglutide (RR = 0.66, 95%CIs = 0.44 to 0.99), empagliflozin (RR = 0.66, 95%CIs = 0.47 to 0.93), and injectable semaglutide (RR = 0.67, 95%CIs = 0.49 to 0.91) were associated with significantly decreased risk of COPD compared to control (Figs. 2C and 3C, and eTable S5J). Although some additional regimens showed numerically favourable point estimates, the corresponding intervals were wider, indicating lower precision and limiting confident differentiation across agents.

None of the regimens investigated in this NMA was associated with significantly different status asthmaticus in asthmatic episode (eFigure S1I, eFigure S2I, and eTable S5K), emphysema in COPD episode (eFigure S1J, eFigure S2J, and eTable S5L), or chronic bronchitis in COPD episode (eFigure S1K, eFigure S2K, and eTable S5M) compared to the controls.

### Acceptability

The tirzepatide (RR = 0.73, 95%CIs = 0.60 to 0.89), canagliflozin (RR = 0.73, 95%CIs = 0.61 to 0.88), dulaglutide (RR = 0.76, 95%CIs = 0.63 to 0.92), injectable semaglutide (RR = 0.78, 95%CIs = 0.67 to 0.91), sitagliptin (RR = 0.81, 95%CIs = 0.73 to 0.91), saxagliptin (RR = 0.82, 95%CIs = 0.69 to 0.96), albiglutide (RR = 0.84, 95%CIs = 0.72 to 0.99), and empagliflozin (RR = 0.85, 95%CIs = 0.75 to 0.97) were associated with significantly less treatment discontinuation rates than the control group did (eFigure S1L, eFigure S2L, and eTable S5N).

### Meta-regression, assessment of bias, treatment ranking, heterogeneity, sensitivity analyses, risk of bias, and certainty of evidence

There was no significant association between treatment duration and risk of ACOS according to the meta-regression result (eTable S6). Race and ethnicity were not entered into quantitative meta-regression because reporting was sparse and non-standardised across trials; these data were therefore summarised descriptively in Table 1 and eTable S4. No evidence of small-study effects was found on visual inspection of comparison-adjusted funnel plots (eFigure S4A–C), and Egger's regression tests were non-significant (eFigure S5A–SSC). SUCRA demonstrated the relative rankings of risk (eTable S7A–7C), which should be interpreted with caution because of the rare events of asthma-COPD overlap syndrome. Heterogeneity across the primary and secondary outcome networks was generally low, with most comparisons showing *I*^*2*^ values of 0% and tau-squared values of 0; only a small number of individual comparisons showed moderate heterogeneity (eTable S8A–C). No major inconsistency was detected using loop-specific, node-splitting, or design-by-treatment interaction methods (eTable S9A–D). Using the Cochrane Risk of Bias 2.0 tool, 80 of the 128 trials (62.5%) were judged to be at low risk, 37/128 (28.9%) had some concerns, and 11/128 (8.6%) were rated as high risk (eFigure S6). Certainty of evidence across comparisons ranged from moderate to high under GRADE ratings (eTable S10), with most downgrades attributed to imprecision due to wide confidence intervals.

## Discussion

We summarised the main findings of this study in Table 2. This network meta-analysis provides compelling evidence that select novel antidiabetic agents exhibit protective effects against asthma–COPD overlap syndrome (ACOS), with potential implications for respiratory disease prevention in at-risk populations. Specifically, we identified that three sodium–glucose cotransporter 2 (SGLT2) inhibitors—canagliflozin, empagliflozin, and dapagliflozin—and one injectable GLP-1 receptor agonist—semaglutide—were significantly associated with reduced risk of ACOS. These effects were dose-responsive and particularly evident among individuals with type 2 diabetes. In contrast, saxagliptin, a DPP-4 inhibitor, was linked to increased asthma risk.

**Table 2:** Summary of the principal findings by outcome domain.

Outcome domain	Principal findings
Primary outcome: overall ACOS risk	Canagliflozin, injectable semaglutide, empagliflozin, and dapagliflozin were associated with lower ACOS risk than control in the primary analysis. Significant dose-stratified associations were observed for high-dose canagliflozin, high-dose injectable semaglutide, medium-dose dulaglutide, low-dose empagliflozin, and high-dose dapagliflozin.
Key subgroup signals	In the diabetes mellitus subgroup, only canagliflozin remained significantly associated with lower ACOS risk. In trials with participants aged younger than 65 years, injectable semaglutide and dapagliflozin remained significant; in trials with participants aged 65 years or older, only dulaglutide remained significant. In male-predominant trials, canagliflozin, injectable semaglutide, dulaglutide, empagliflozin, and dapagliflozin were associated with lower ACOS risk, whereas no regimen reached statistical significance in female-predominant trials. In trials with treatment duration ≥1 year, injectable semaglutide, empagliflozin, and dapagliflozin remained significant.
Secondary respiratory outcomes	For asthma, only dapagliflozin was associated with lower risk, whereas saxagliptin was associated with higher risk. For COPD, lower risk was observed with canagliflozin, sitagliptin, dulaglutide, empagliflozin, and injectable semaglutide. No regimen was associated with significant differences in status asthmaticus, emphysema, or chronic bronchitis.
Acceptability	Lower dropout rates than control were observed with tirzepatide, canagliflozin, dulaglutide, injectable semaglutide, sitagliptin, saxagliptin, albiglutide, and empagliflozin.
Robustness	The primary asthma-COPD syndrome associations for canagliflozin, injectable semaglutide, empagliflozin, and dapagliflozin were reproduced in the HR-based sensitivity analysis. Meta-regression showed no significant association between treatment duration and ACOS risk. Small-study effects, major heterogeneity, and inconsistency were not detected, and certainty of evidence was generally moderate to high.

Summary of statistically significant findings across the primary, subgroup, secondary, and sensitivity analyses. Sex-related subgroup findings reflect trial-level composition only; race and ethnicity were not analysed because reporting was limited and non-standardised.

Abbreviation: ACOS: asthma–COPD overlap syndrome; COPD: chronic obstructive pulmonary disease.

One of the most notable findings was the disease-specific benefit of injectable semaglutide, especially in reducing COPD-related risk. Prior pairwise meta-analyses had not demonstrated consistent pulmonary benefits for GLP-1 receptor agonists,^6^^,^^7^ likely due to their pooled analytical approach and lack of differentiation among agents. Wang et al. conducted a NMA on this topic,^151^ but their grouping of multiple GLP-1RAs into a single treatment node diluted potential compound-specific effects. Our approach, by evaluating semaglutide as an individual agent, allowed the detection of its distinct preventive signal for COPD.

Mechanistically, animal studies support this observation. Semaglutide has been shown to exert anti-inflammatory and barrier-stabilising effects in lung tissue. In obese murine models, Yang et al. demonstrated that semaglutide restores expression of lung-specific proteins including LYVE1, BRAF, RGCC, and CHMP5.^1^ Similarly, Jiang et al. reported that semaglutide inhibited histone deacetylase 5-mediated nuclear factor kappa-light-chain-enhancer of activated B cells activation, a pathway implicated in chronic airway inflammation and ACOS pathophysiology.^152^^,^^153^ These findings suggest plausible molecular mechanisms for the respiratory benefits observed in clinical data.

In terms of SGLT2 inhibitors, our analysis revealed differentiated efficacy profiles: dapagliflozin showed a significant protective effect for asthma events, while canagliflozin and empagliflozin demonstrated greater risk reduction for COPD. This divergence may reflect underlying differences in pharmacokinetics, metabolic pathways, and organ-specific anti-inflammatory properties.^154^ For instance, in an animal model of allergic asthma, dapagliflozin mitigated ovalbumin-induced bronchial hyperreactivity and eosinophilic inflammation, a hallmark of asthma pathogenesis.^2^ Conversely, both canagliflozin and empagliflozin have been associated with reduced pulmonary CO_2_ retention and lower systemic oxidative stress in COPD models.^155^ Regarding the dose-stratification analysis for empagliflozin, the low-dose signal should be interpreted cautiously, as the high-dose category also showed a directionally favourable estimate but with less precision, precluding firm inference regarding within-drug dose hierarchy.

An important safety signal was the observed association between saxagliptin and increased asthma incidence. Although definitive causal mechanisms remain elusive, one hypothesis is that saxagliptin may increase vulnerability to upper respiratory tract infections,^156^ which in turn are known precipitants of asthma exacerbations.^157^ While this association was statistically significant in both general and diabetic subgroups, further mechanistic or prospective studies are warranted to validate this risk.

Our study design confers several strengths. First, we exclusively included RCTs with systematic adverse event surveillance, thereby enhancing the reliability of safety outcome estimates. Second, our agent-specific, dose-stratified NMA allowed a level of resolution not possible in conventional pairwise analyses, revealing distinct protective profiles among agents within the same pharmacologic class. Third, subgroup analyses by diabetic status provide clinically actionable insights, particularly for tailoring prevention strategies in high-risk populations.

However, several limitations should be acknowledged. By focussing exclusively on RCTs, we may have excluded valuable observational data that capture long-term respiratory outcomes. An important limitation concerns outcome ascertainment. ACO/ACOS remains a heterogeneous clinical construct without a universally accepted diagnostic definition, and the included trials were not primarily designed to prospectively detect or adjudicate ACOS as a prespecified endpoint. Consequently, our primary analysis relied on reported ACOS-related events as described in trial publications and Supplementary Sources. This may have introduced misclassification or under-ascertainment, which could in turn have influenced agent-level estimates, particularly for rare-event comparisons. An important limitation is that the precision of the treatment estimates was not uniform across the network. Some regimens were informed by larger evidence bases and more events, whereas others were supported by relatively sparse data and wider confidence intervals. Consequently, apparent differences in ranking or point estimates should not be overinterpreted as definitive evidence that a given agent is the most or least favourable option. The present study is therefore better suited to identifying comparative signals than to establishing a reliable best-to-worst hierarchy across regimens. Finally, although subgroup analyses were performed, formal interaction tests for whether treatment effects differed across subgroup factors could not be conducted within our current STATA-based network meta-analytic framework. Therefore, these subgroup findings should be interpreted descriptively rather than as definitive evidence of effect modification. Most ranking evidence was indirect rather than head-to-head, increasing susceptibility to between-trial bias and limiting confidence in apparent treatment hierarchies.

In conclusion, this large-scale network meta-analysis synthesising data from 316,832 participants across 128 randomised trials reveals significant heterogeneity in the respiratory effects of novel antidiabetic agents. Our findings indicate that only specific compounds—namely canagliflozin, empagliflozin, dapagliflozin, and injectable semaglutide—confer measurable prophylactic benefits against asthma–COPD overlap syndrome (ACOS), and that these effects are both agent-specific and dose-dependent. These findings indicate comparative associations with reported ACOS-related events and should be interpreted cautiously until prospectively designed respiratory-outcome trials are available. Prospective head-to-head trials with prespecified respiratory outcomes are needed to confirm these signals and clarify whether apparent differences reflect true regimen-specific effects.

## Contributors

BYZ and C-WH contributed equally as co-first authors. M-WS and PTT contributed equally as corresponding authors. PTT, C-WH, BYZ, M-WS, C-MH, and W-CY contributed to the literature search, data extraction, data verification, statistical analysis, and drafting of the manuscript. BS, Y-WC, W-TL, J-JC, T-YC, and S-PH contributed to study design, conceptualisation, and critical revision of the manuscript. PTT, H-YW, Y-LS, B-SZ, C-TL, K-PS, and C-SL contributed to supervision, major revision of the manuscript, and submission management. PTT, C-WH, and BYZ accessed and verified the underlying extracted data. All authors had full access to the data, read and approved the final version of the manuscript, and accepted responsibility for the decision to submit for publication.

## Data sharing statement

The extracted study-level data, statistical code, and data dictionary will be made available from the corresponding authors upon reasonable request. The study protocol and statistical analysis plan will also be available on request. Data will be shared with researchers providing a methodologically sound proposal, and a data access agreement may be required.

## Declaration of interests

C-WH reports support from the Taiwan National Science and Technology Council for the present manuscript. All other authors declare no competing interests. This study was supported by the Taiwan National Science and Technology Council (112-2314-B-182-070-MY3) to Chih-Wei Hsu. Brendon Stubbs receives support from the NIHR, the NIHR Biomedical Research Centre at South London and Maudsley NHS Foundation Trust, and the Maudsley Charity, King's College London.
