# Supplementary material for: Reassessing the risk-modifying effects of novel antidiabetic agents on asthma–COPD overlap syndrome: a dose-stratified network meta-analysis of 316,832 adults from 128 randomised trials
Source: eClinicalMedicine. 2026 Jun 19;97:104026. doi: 10.1016/j.eclinm.2026.104026 (PMC13311999; doi:10.1016/j.eclinm.2026.104026)
Supplement: Supplementary Material [file mmc1.pdf]

<u>**List of content in supplement materials**</u>

Reassessing the risk-modifying effects of novel antidiabetic agents on asthma–COPD overlap syndrome

A dose-stratified network meta-analysis of 316,832 adults from 128 randomised trials

Ping-Tao Tseng, et al.

eFigure 1	(A) Network structure of primary outcome: overall asthma-COPD overlap syndrome risk in aspect of various dosage subgroups
	(B) Network structure of primary outcome: overall asthma-COPD overlap syndrome risk focusing on participants with diabetes mellitus
	(C) Network structure of primary outcome: overall asthma-COPD overlap syndrome risk focusing on participants with age younger than 65 years old
	(D) Network structure of primary outcome: overall asthma-COPD overlap syndrome risk focusing on participants with age at least 65 years old
	(E) Network structure of primary outcome: overall asthma-COPD overlap syndrome risk focusing on trials with male predominance
	(F) Network structure of primary outcome: overall asthma-COPD overlap syndrome risk focusing on trials with female predominance
	(G) Network structure of primary outcome: overall asthma-COPD overlap syndrome risk focusing on trials with treatment duration at least 1 year

	(H) Network structure of primary outcome: overall asthma-COPD overlap syndrome risk using hazard ratio based on time-to-event data
	(I) Network structure of secondary outcome: status asthmaticus in asthmatic episode
	(J) Network structure of secondary outcome: emphysema in COPD episode
	(K) Network structure of secondary outcome: chronic bronchitis in COPD episode
	(L) Network structure of acceptability: treatment discontinuation rate
eFigure 2	(A) Forest plot of primary outcome: overall asthma-COPD overlap syndrome risk in aspect of various dosage subgroups
	(B) Forest plot of primary outcome: overall asthma-COPD overlap syndrome risk focusing on participants with diabetes mellitus
	(C) Forest plot of primary outcome: overall asthma-COPD overlap syndrome risk focusing on participants with age younger than 65 years old
	(D) Forest plot of primary outcome: overall asthma-COPD overlap syndrome risk focusing on participants with age at least 65 years old
	(E) Forest plot of primary outcome: overall asthma-COPD overlap syndrome risk focusing on trials with male predominance
	(F) Forest plot of primary outcome: overall asthma-COPD overlap syndrome risk focusing on trials with female predominance
	(G) Forest plot of primary outcome: overall asthma-COPD overlap syndrome risk focusing on trials with treatment duration at least 1 year
	(H) Forest plot of primary outcome: overall asthma-COPD overlap syndrome risk using hazard ratio based on time-to-event data
	(I) Forest plot of secondary outcome: status asthmaticus in asthmatic episode
	(J) Forest plot of secondary outcome: emphysema in COPD episode
	(K) Forest plot of secondary outcome: chronic bronchitis in COPD episode
	(L) Forest plot of acceptability: treatment discontinuation rate
eFigure 3	Individual study result of primary outcome: overall asthma-COPD overlap syndrome risk

eFigure 4	(A) Funnel plot for primary outcome: overall asthma-COPD overlap syndrome risk
	(B) Funnel plot for primary outcome: subgroup of asthma risk
	(C) Funnel plot for primary outcome: subgroup of COPD risk
eFigure 5	(A) Egger test for primary outcome: overall asthma-COPD overlap syndrome risk
	(B) Egger test for primary outcome: subgroup of asthma risk
	(C) Egger test for primary outcome: subgroup of COPD risk
eFigure 6	Risk of bias tool 2.0
eTable 1	Keyword used in each database and search results
eTable 2	Dosage stratification (stratified according to the included original RCTs)
eTable 3	Excluded studies and reason
eTable 4	Characteristics of the included studies
eTable 5	(A) League table of primary outcome: overall asthma-COPD overlap syndrome risk
	(B) League table of primary outcome: overall asthma-COPD overlap syndrome risk focusing on participants with diabetes mellitus
	(C) League table of primary outcome: overall asthma-COPD overlap syndrome risk focusing on participants with age younger than 65 years old
	(D) League table of primary outcome: overall asthma-COPD overlap syndrome risk focusing on participants with age at least 65 years old
	(E) League table of primary outcome: overall asthma-COPD overlap syndrome risk focusing on trials with male predominance
	(F) League table of primary outcome: overall asthma-COPD overlap syndrome risk focusing on trials with female predominance
	(G) League table of primary outcome: overall asthma-COPD overlap syndrome risk focusing on trials with treatment duration at least 1 year
	(H) League table of primary outcome: overall asthma-COPD overlap syndrome risk using hazard ratio based on time-to-event data

	(I) League table of secondary outcome: asthma risk
	(J) League table of secondary outcome: COPD risk
	(K) League table of secondary outcome: status asthmaticus in asthmatic episode
	(L) League table of secondary outcome: emphysema in COPD episode
	(M) League table of secondary outcome: chronic bronchitis in COPD episode
	(N) League table of acceptability: treatment discontinuation rate
eTable 6	Meta-regression for primary outcome: overall asthma-COPD overlap syndrome risk associated with treatment duration
eTable 7	(A) SUCRA for primary outcome: overall asthma-COPD overlap syndrome risk
	(B) SUCRA for primary outcome: subgroup of asthma risk
	(C) SUCRA for primary outcome: subgroup of COPD risk
eTable 8	(A) Heterogeneity for primary outcome: overall asthma-COPD overlap syndrome risk
	(B) Heterogeneity for primary outcome: subgroup of asthma risk
	(C) Heterogeneity for primary outcome: subgroup of COPD risk
eTable 9	(A) Side-splitting model inconsistency for primary outcome: overall asthma-COPD overlap syndrome risk
	(B) Side-splitting model inconsistency for primary outcome: subgroup of asthma risk
	(C) Side-splitting model inconsistency for primary outcome: subgroup of COPD risk
	(D) Design-by-treatment model and loop inconsistency for all primary outcomes
eTable 10	GRADE for primary outcome: overall asthma-COPD overlap syndrome risk

eFigure 1A Network structure of primary outcome: overall asthma-COPD overlap syndrome risk in aspect of various dosage subgroups

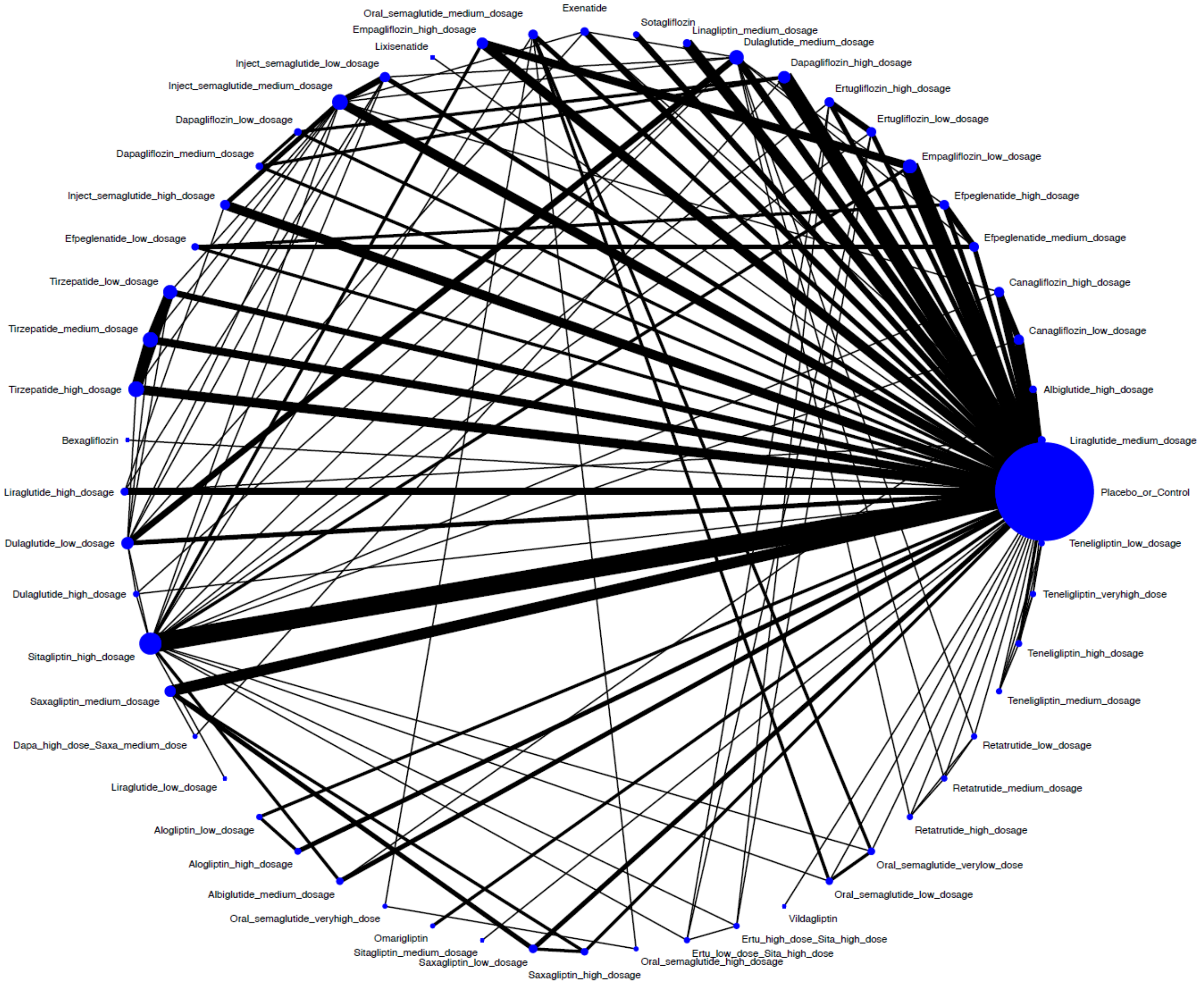

eFigure 1B Network structure of primary outcome: overall asthma-COPD overlap syndrome risk focusing on participants with diabetes mellitus

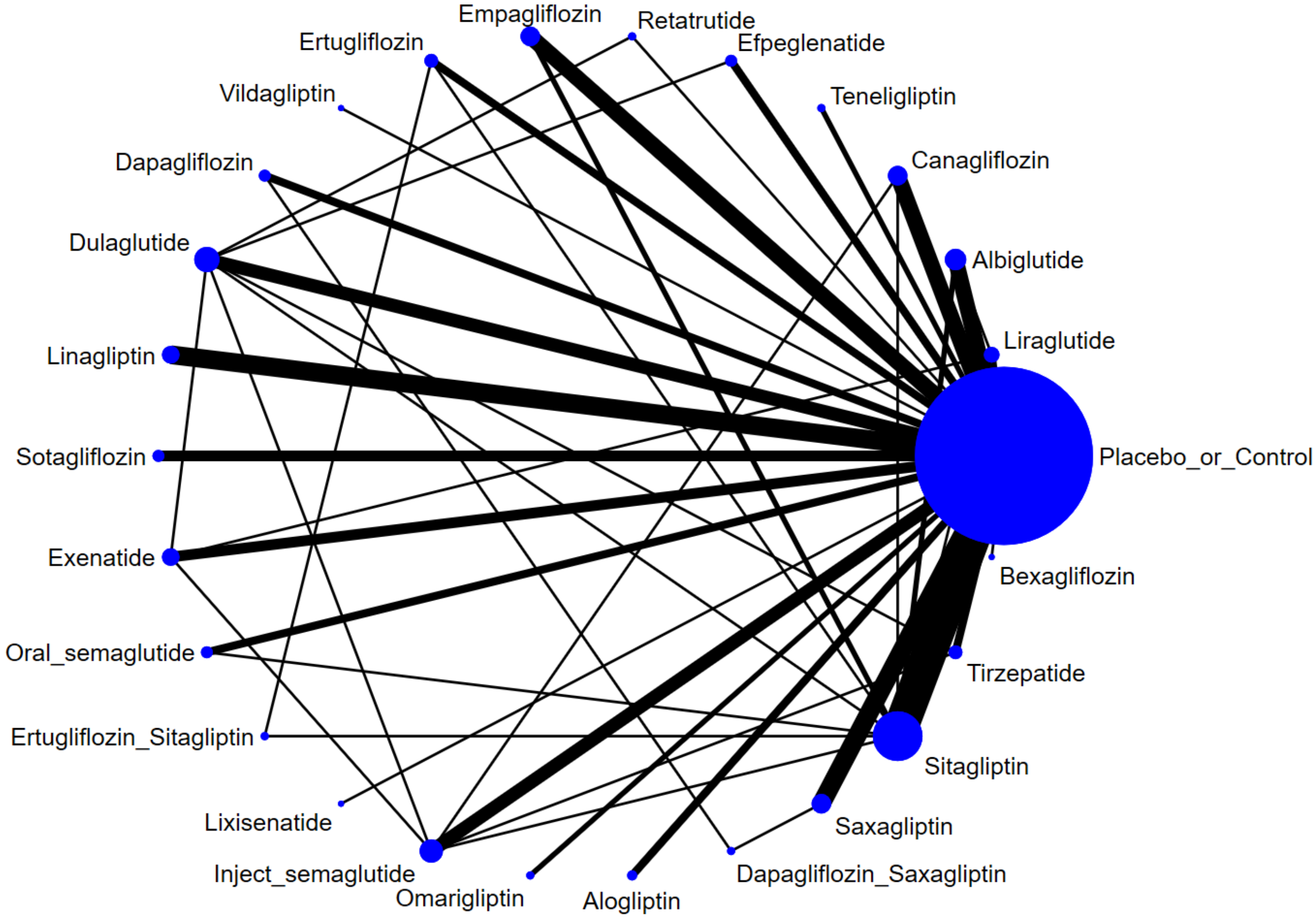

eFigure 1C Network structure of primary outcome: overall asthma-COPD overlap syndrome risk focusing on participants with age younger than 65 years old

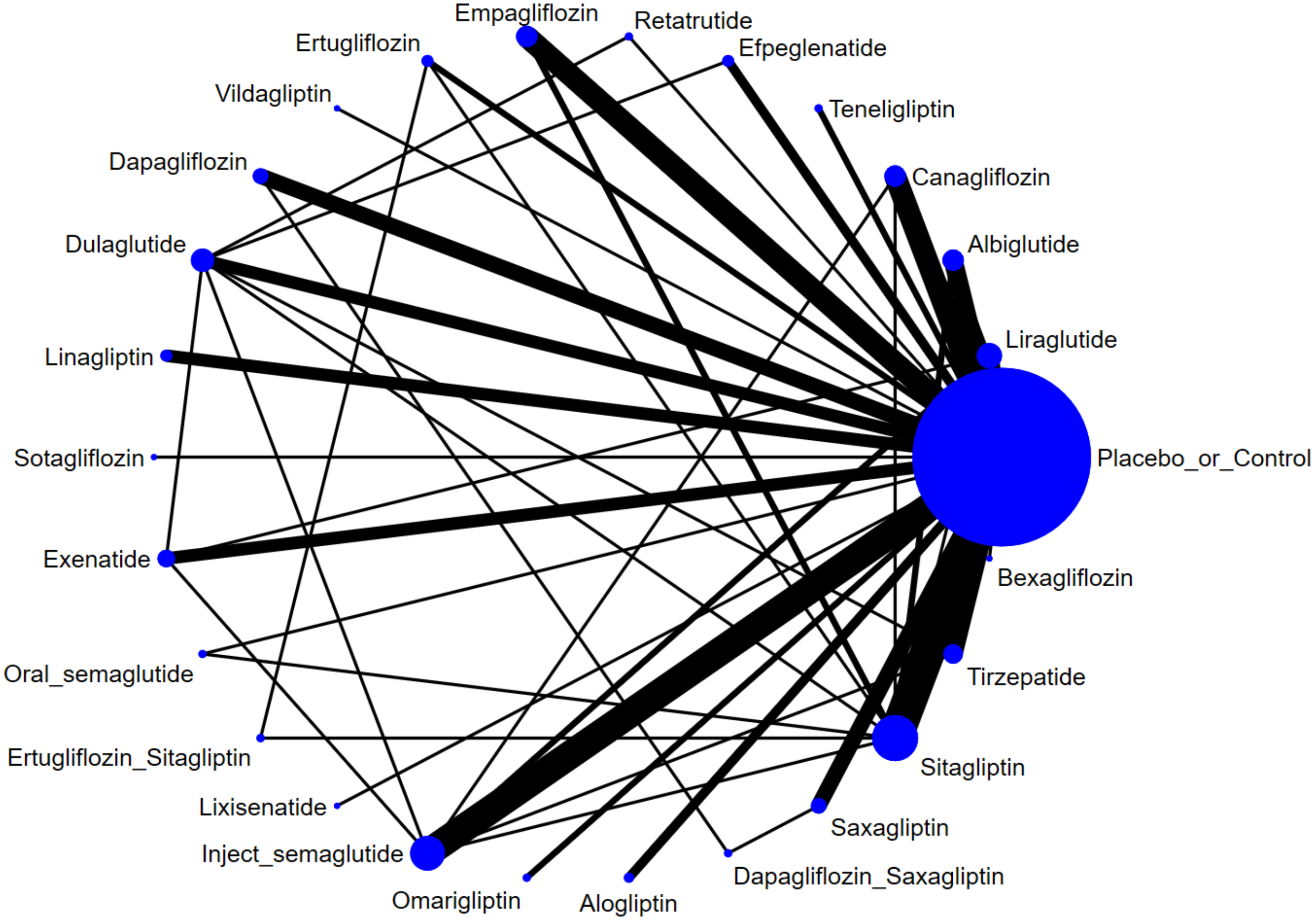

eFigure 1D Network structure of primary outcome: overall asthma-COPD overlap syndrome risk focusing on participants with age at least 65 years old

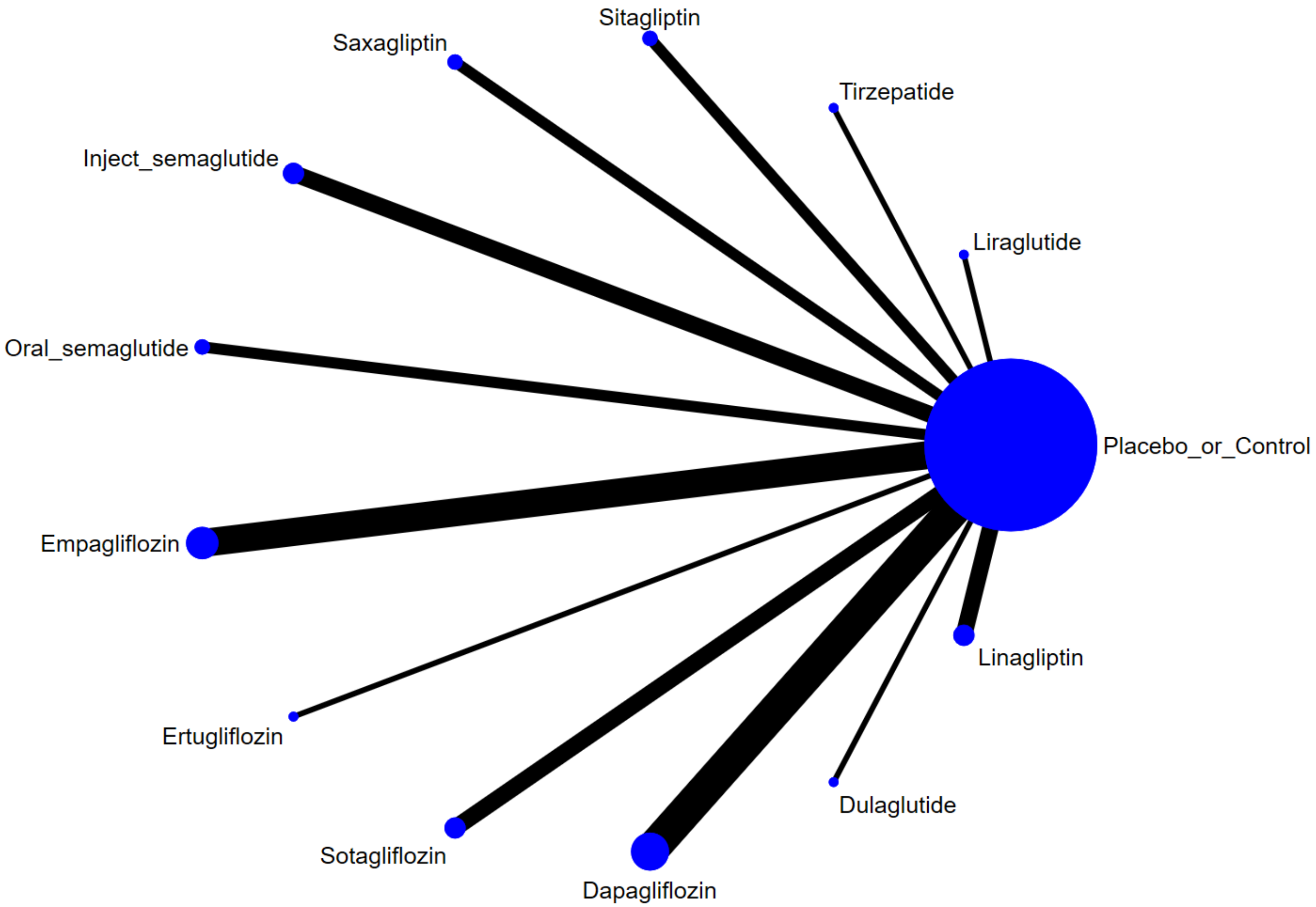

eFigure 1E Network structure of primary outcome: overall asthma-COPD overlap syndrome risk focusing on trials with male predominance

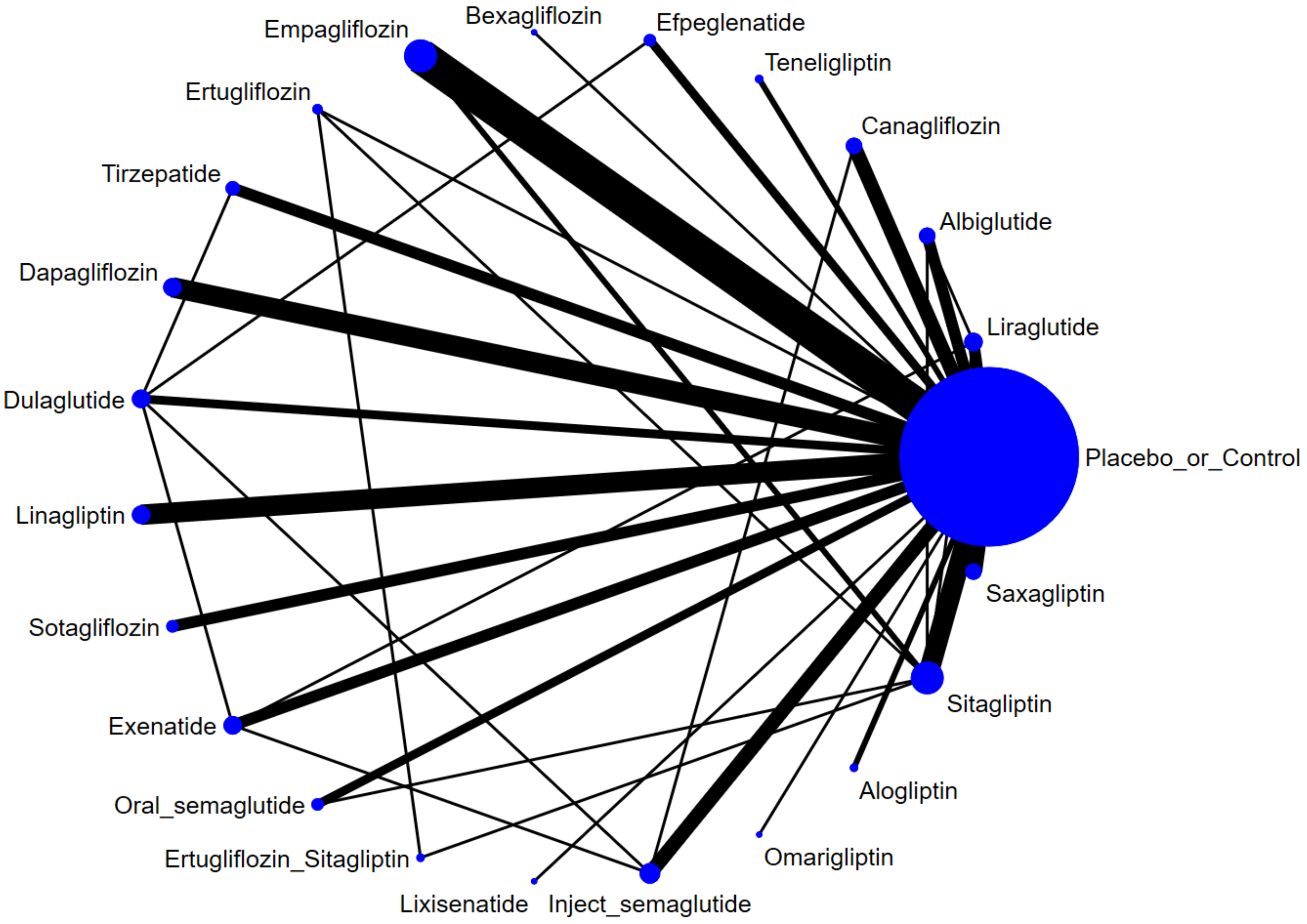

eFigure 1F Network structure of primary outcome: overall asthma-COPD overlap syndrome risk focusing on trials with female predominance

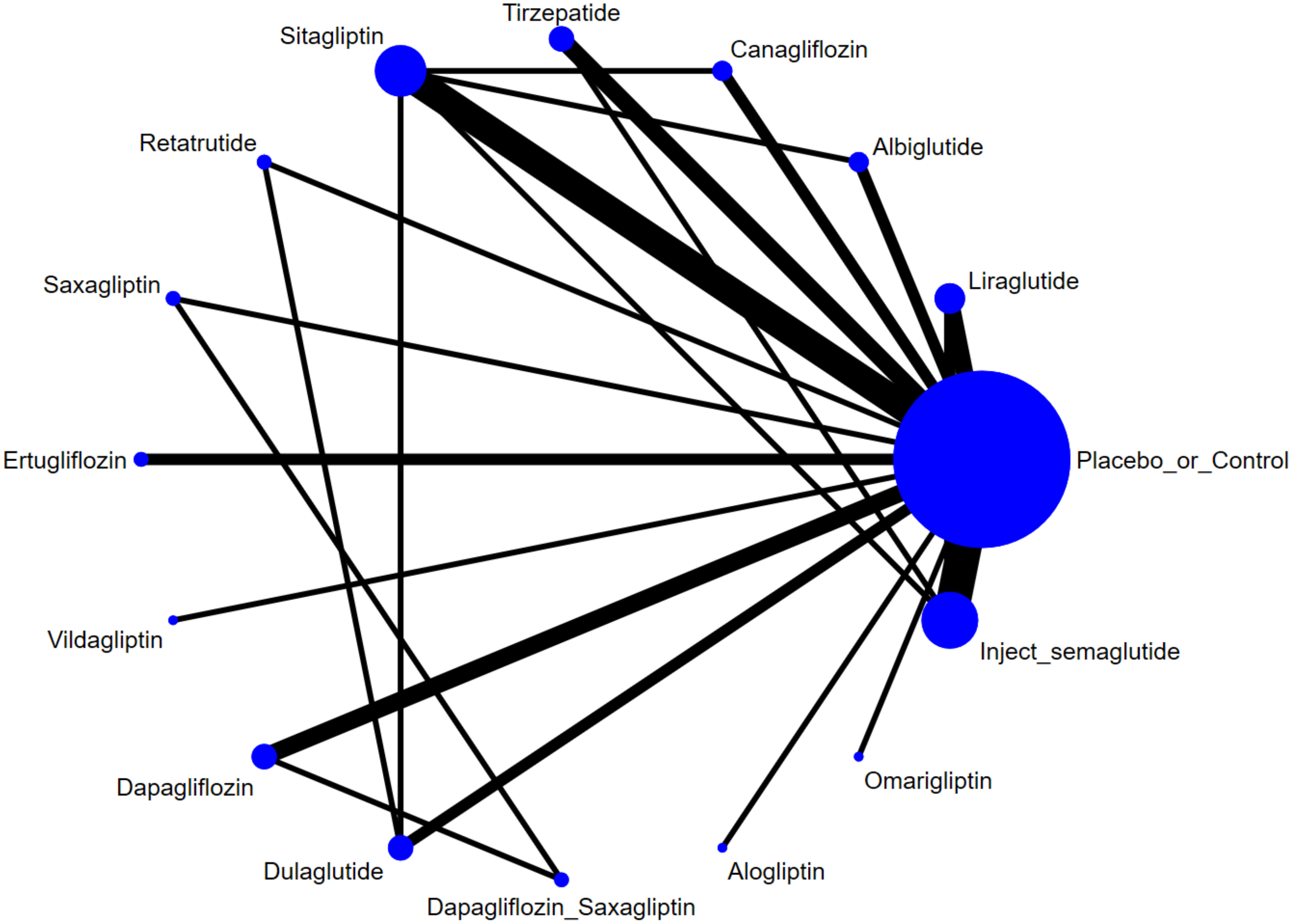

eFigure 1G Network structure of primary outcome: overall asthma-COPD overlap syndrome risk focusing on trials with treatment duration at least 1 year

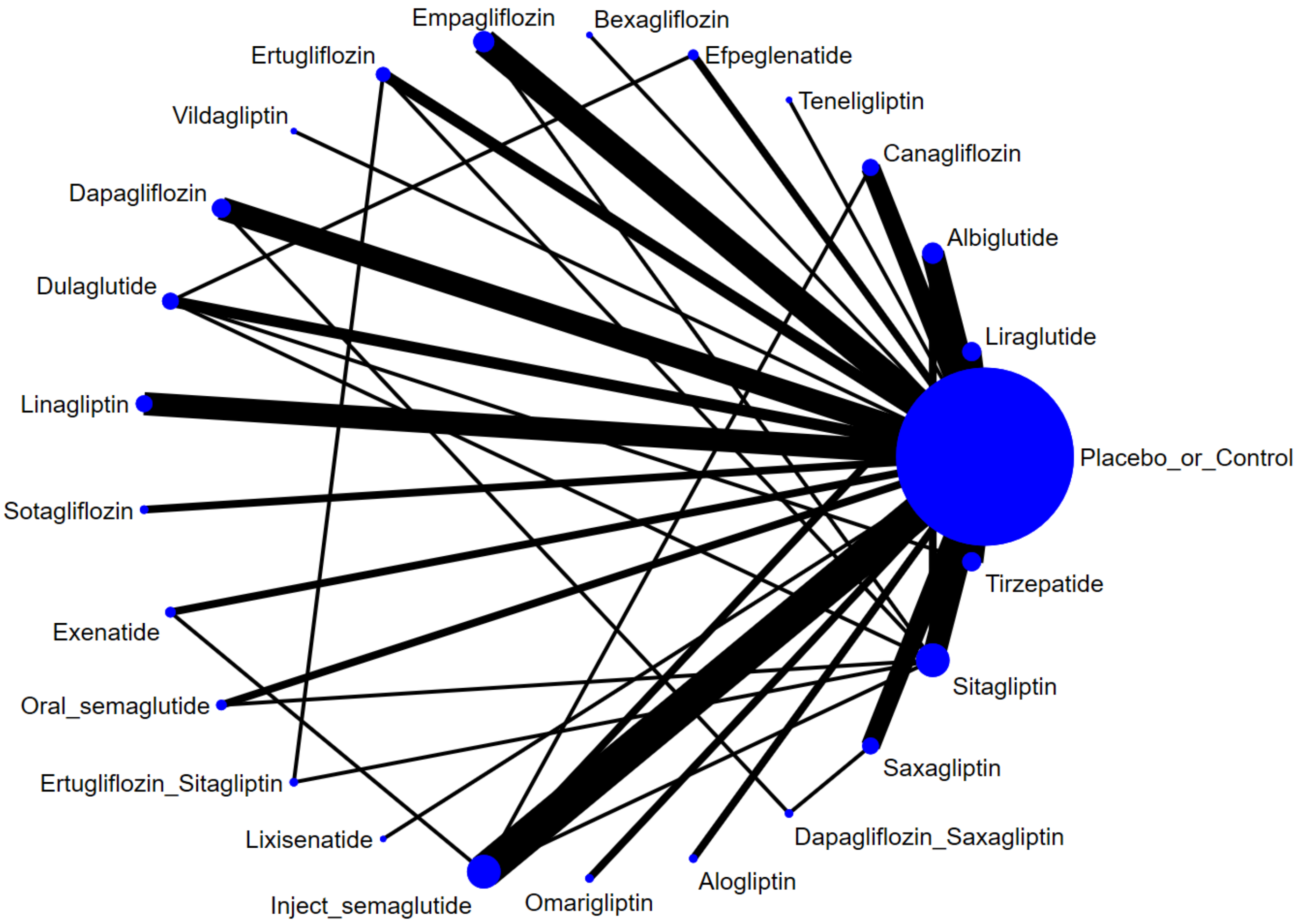

eFigure 1H Network structure of primary outcome: overall asthma-COPD overlap syndrome risk using hazard ratio based on time-to-event data

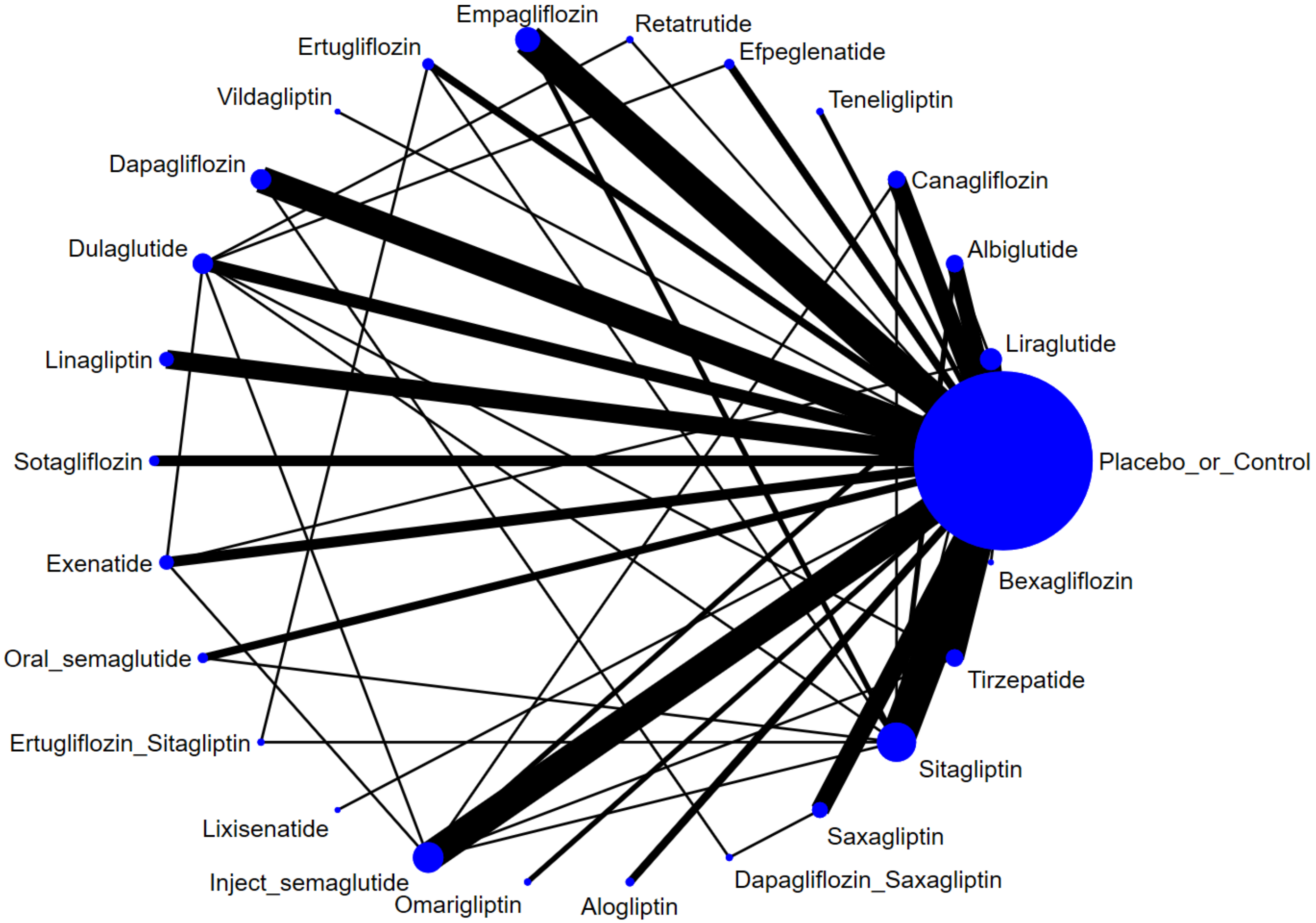

eFigure 1I Network structure of secondary outcome: status asthmaticus in asthmatic episode

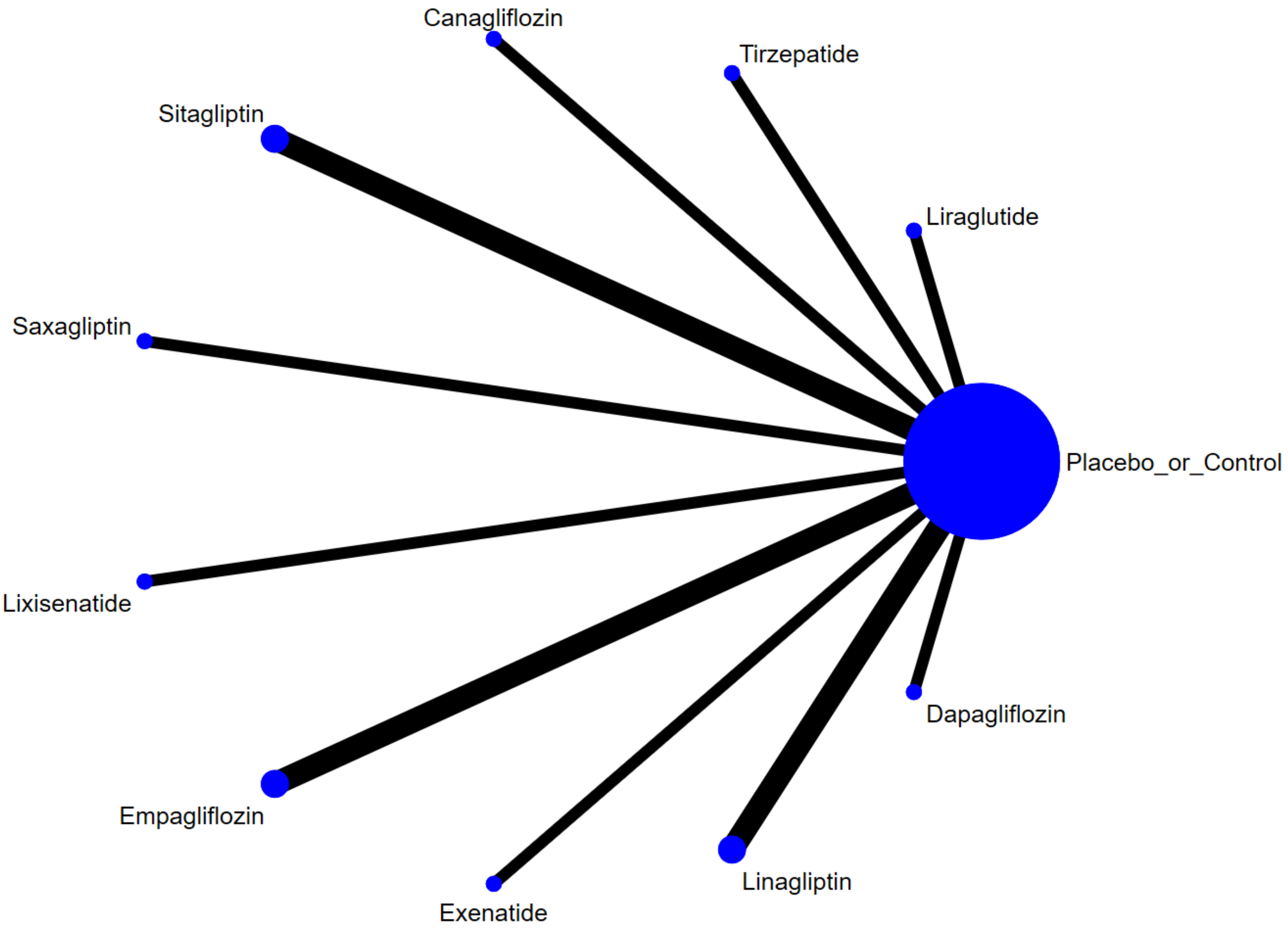

eFigure 1J Network structure of secondary outcome: emphysema in COPD episode

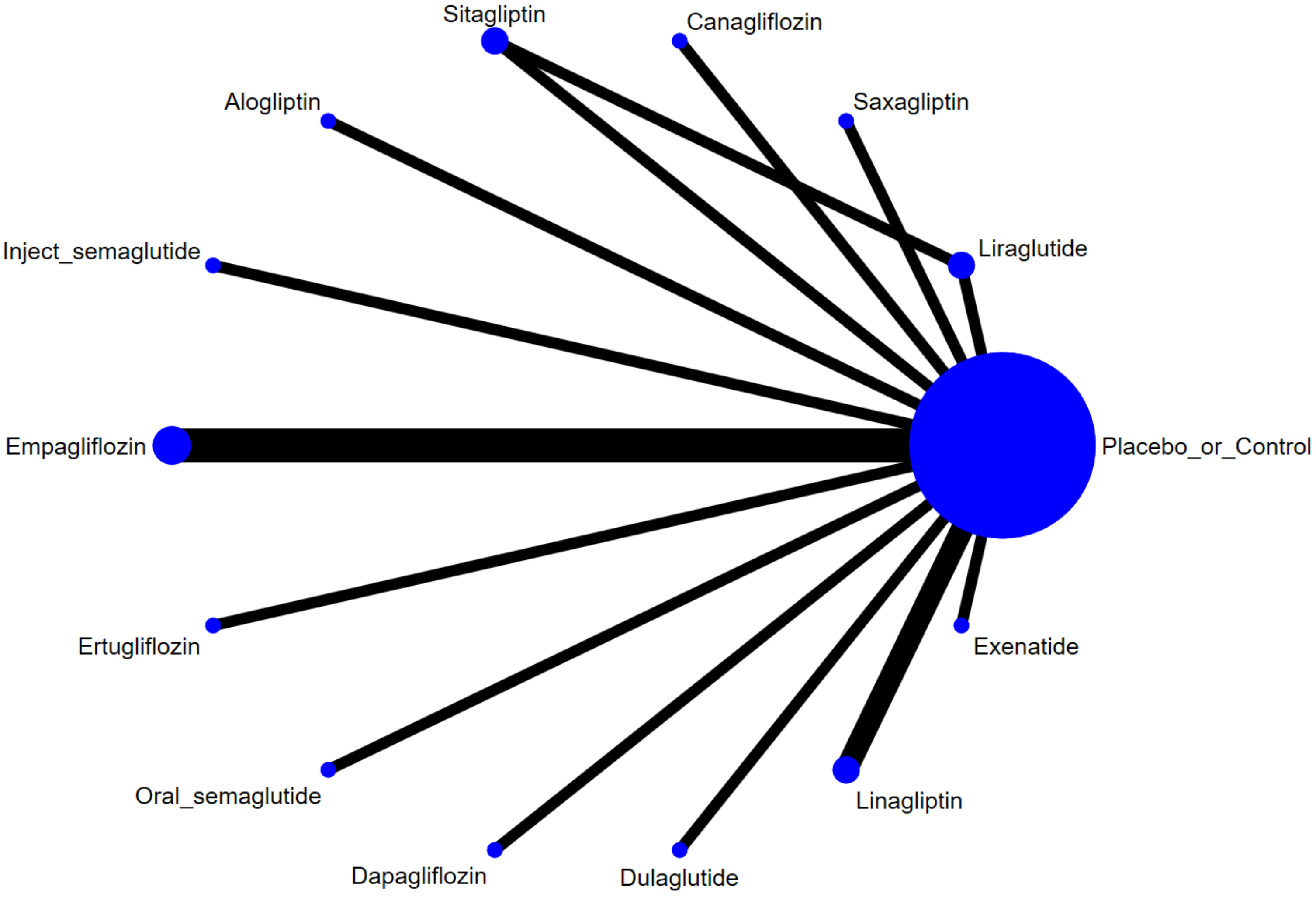

eFigure 1K Network structure of secondary outcome: chronic bronchitis in COPD episode

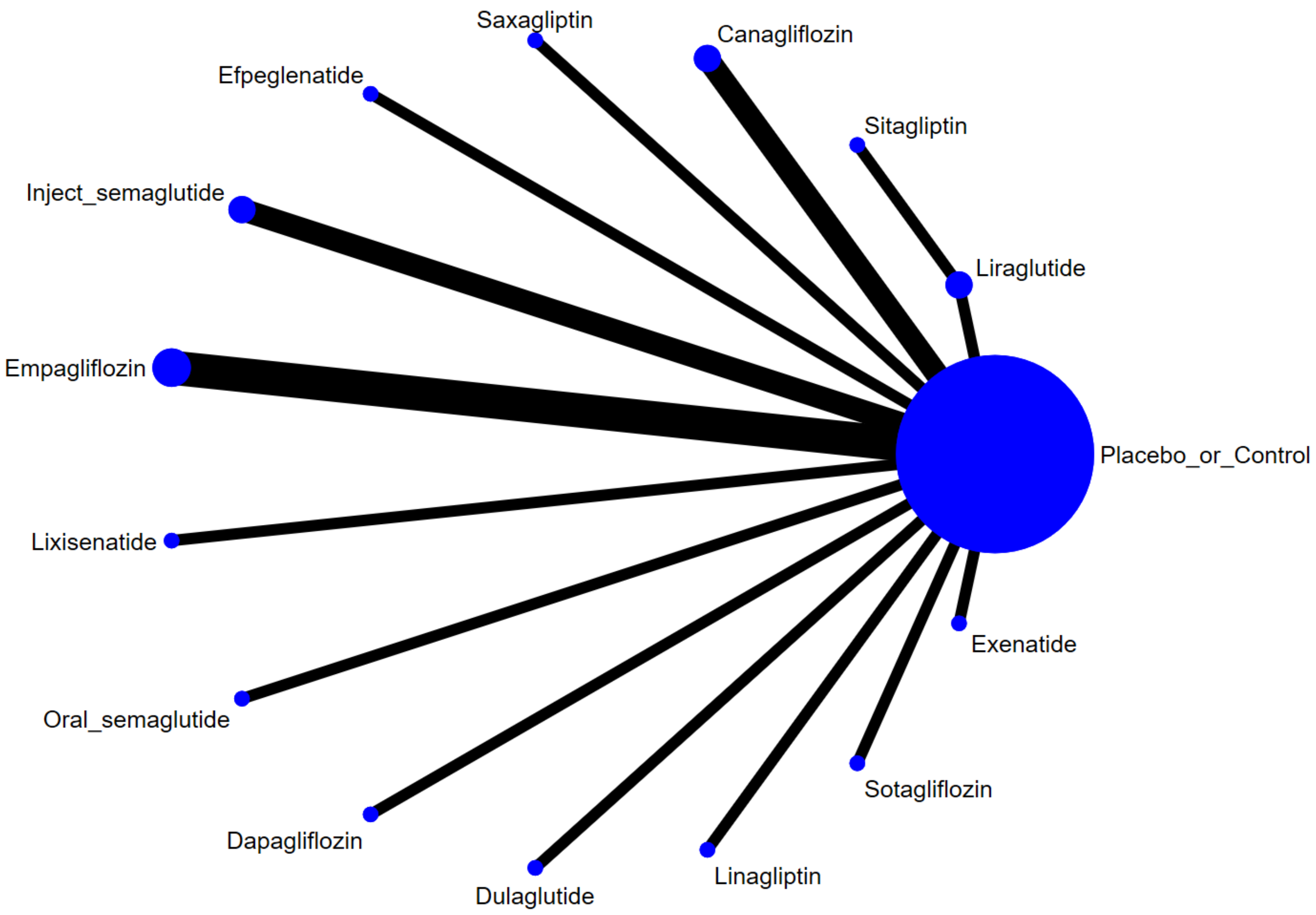

eFigure 1L Network structure of acceptability: treatment discontinuation rate

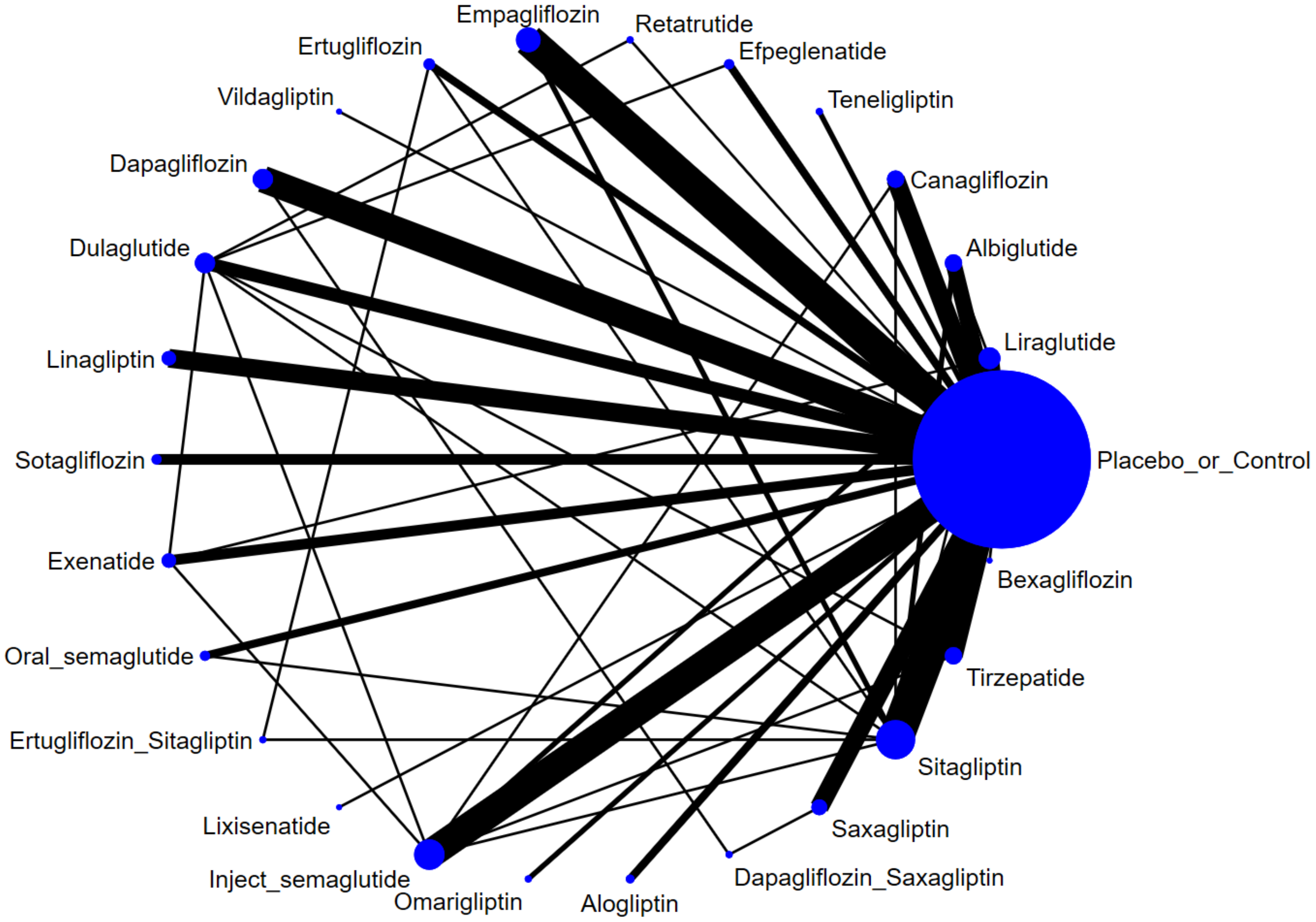

Figure legend of eFigure 1A-1L

Overall structure of the network meta-analysis. The lines between nodes represent direct comparisons in various trials, and the size of each circle is proportional to the number of participants in each specific treatment. The thickness of the lines is proportional to the number of trials connected to the network.

Abbreviation for eFigure 1A-1L:

95%CIs: 95% confidence intervals; DPP4 inhibitor: dipeptidyl peptidase 4 inhibitor; GLP-1 agonist: glucagon-like peptide-1 agonist; NMA: network meta-analysis; RCT: randomised controlled trial; RR: risk ratio; SGLT2 inhibitor: sodium–glucose cotransporter 2 inhibitor

eFigure 2A Forest plot of primary outcome: overall asthma-COPD overlap syndrome risk in aspect of various dosage subgroups

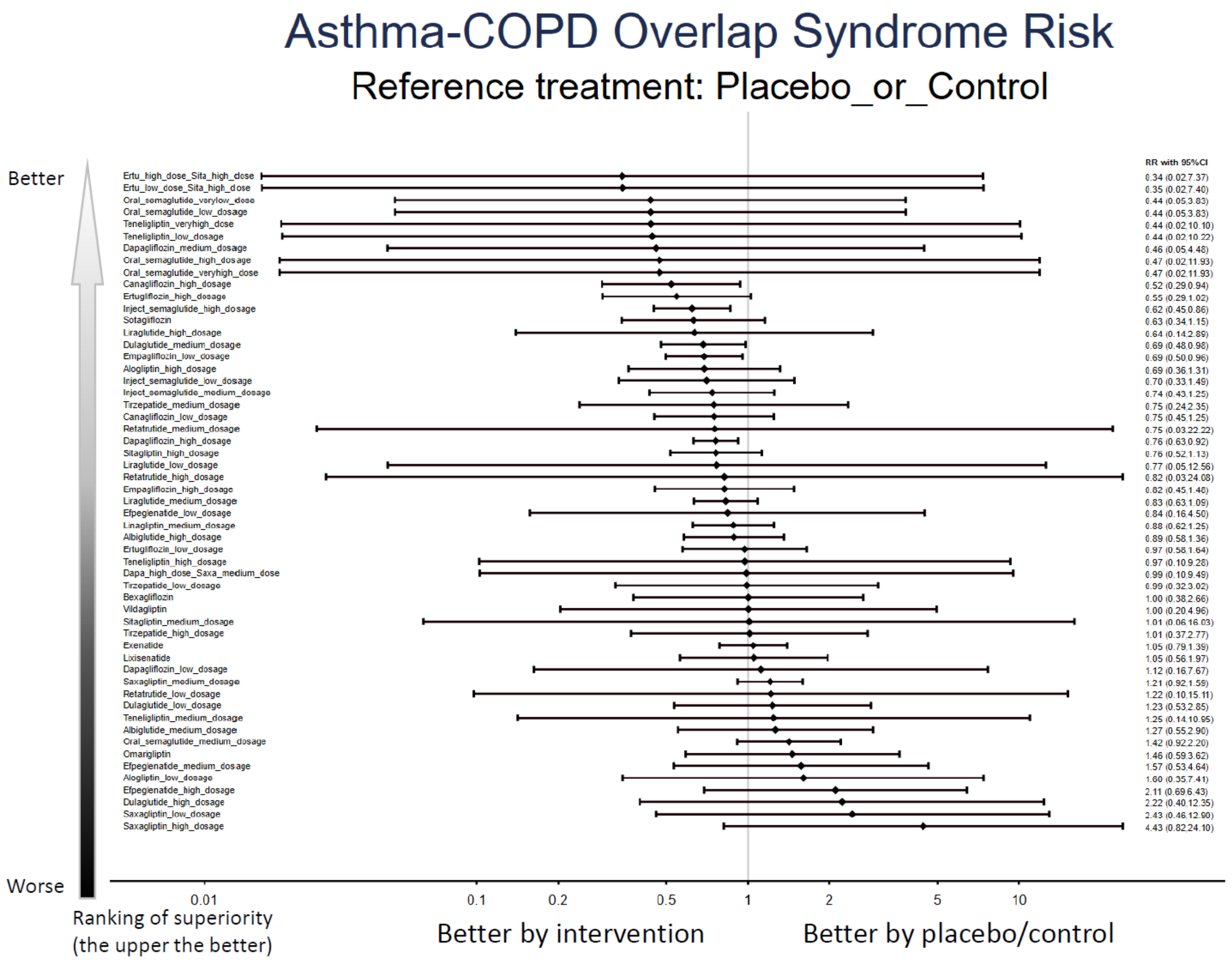

eFigure 2B Forest plot of primary outcome: overall asthma-COPD overlap syndrome risk focusing on participants with diabetes mellitus

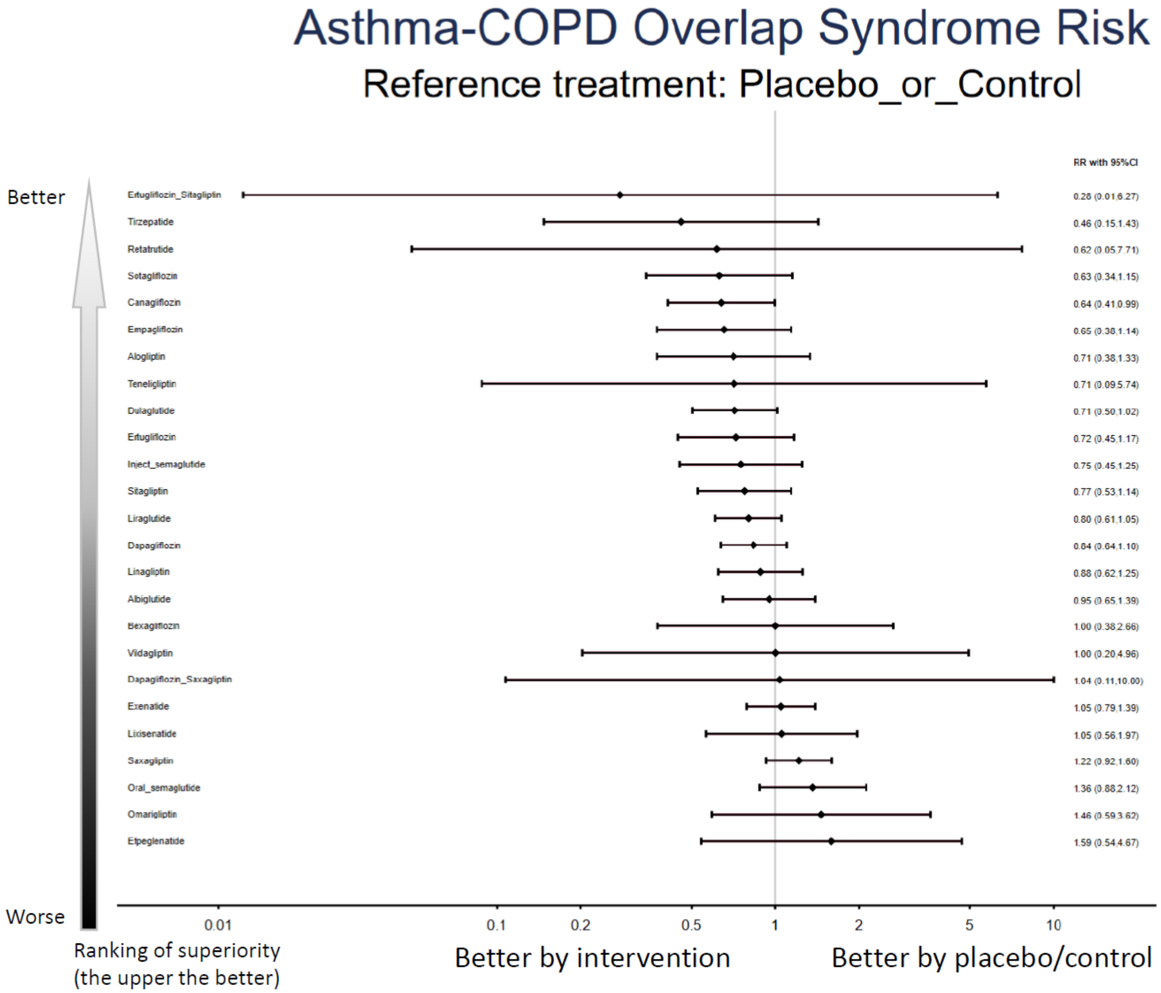

eFigure 2C Forest plot of primary outcome: overall asthma-COPD overlap syndrome risk focusing on participants with age younger than 65 years old

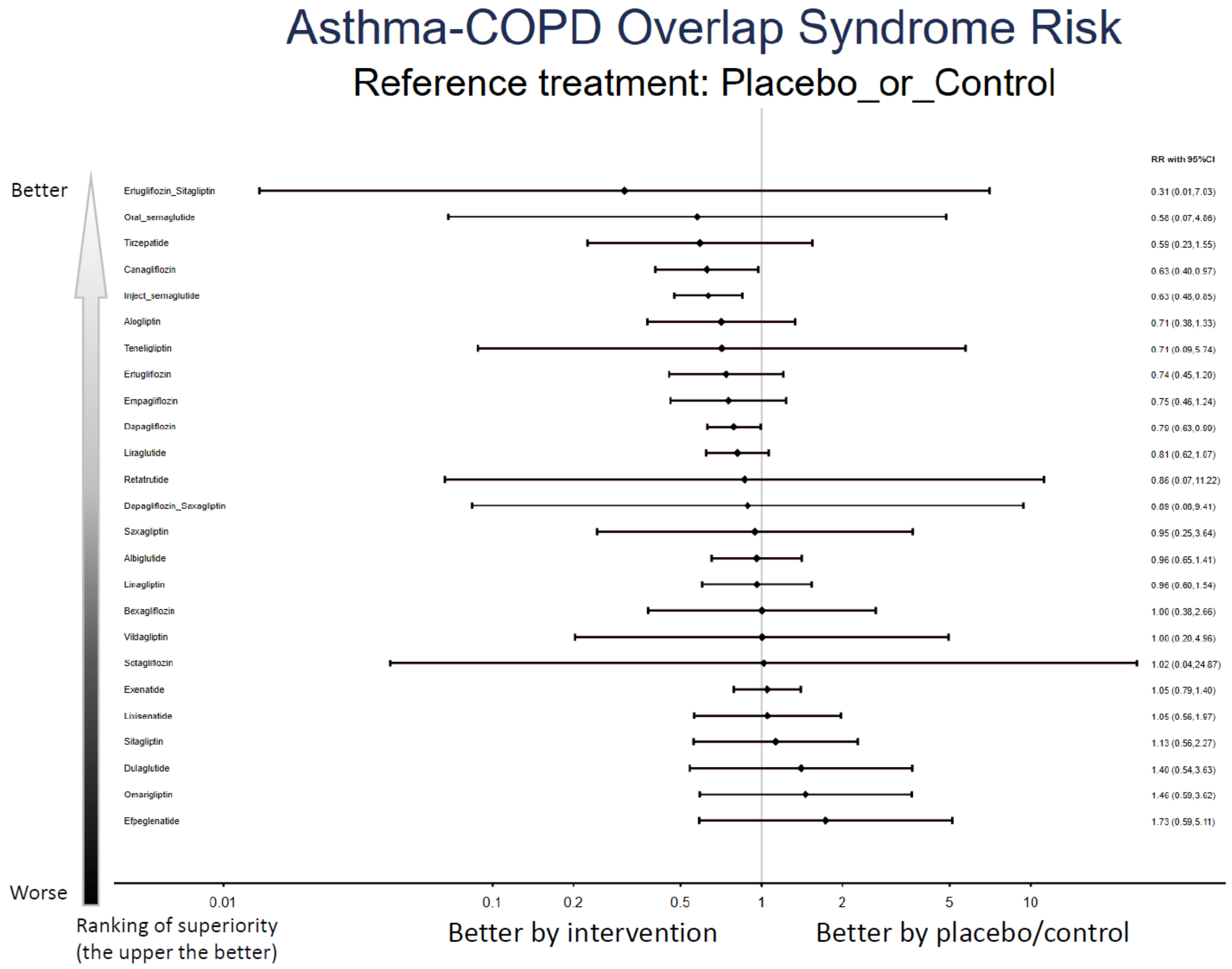

eFigure 2D Forest plot of primary outcome: overall asthma-COPD overlap syndrome risk focusing on participants with age at least 65 years old

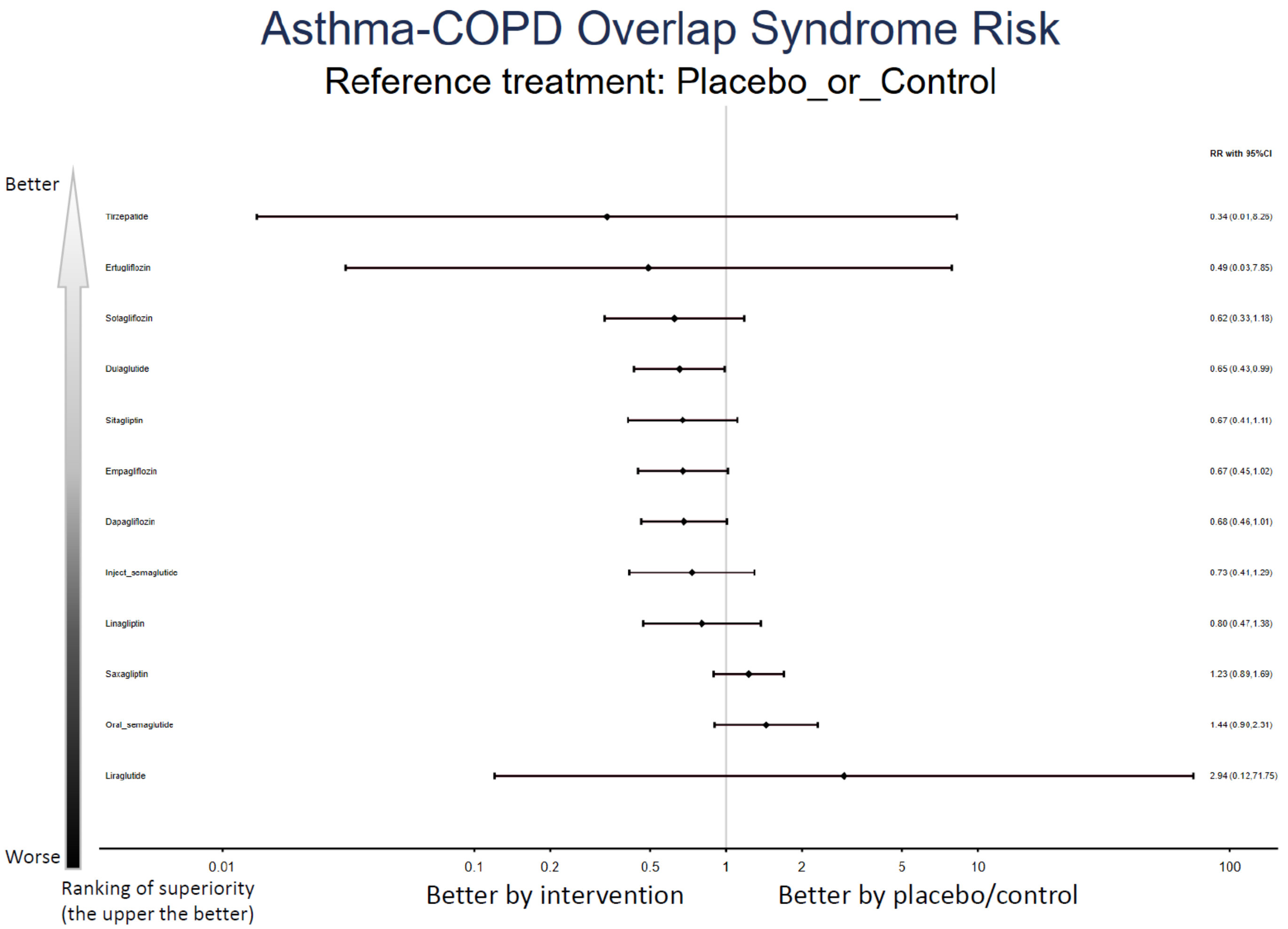

eFigure 2E N Forest plot of primary outcome: overall asthma-COPD overlap syndrome risk focusing on trials with male predominance

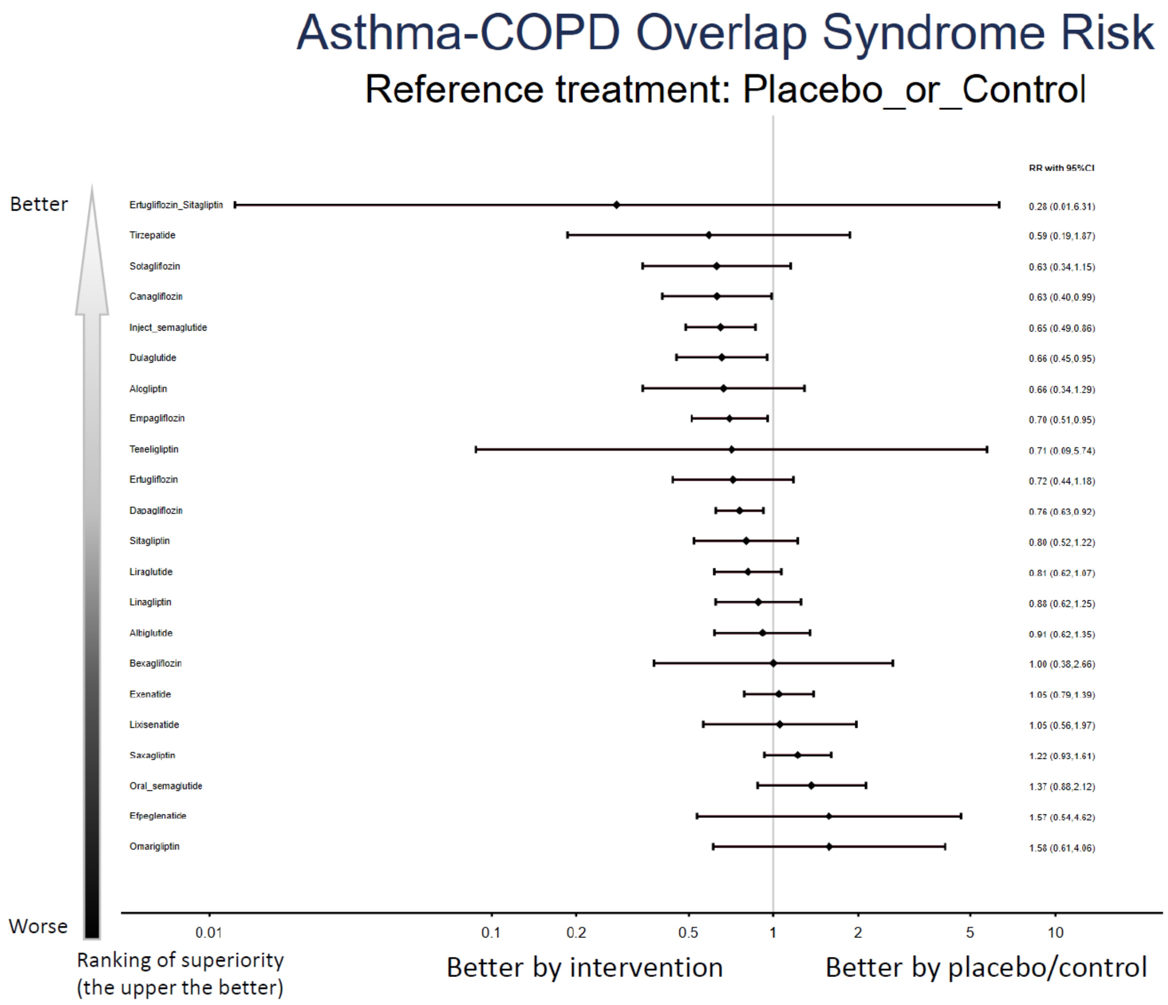

eFigure 2F Forest plot of primary outcome: overall asthma-COPD overlap syndrome risk focusing on trials with female predominance

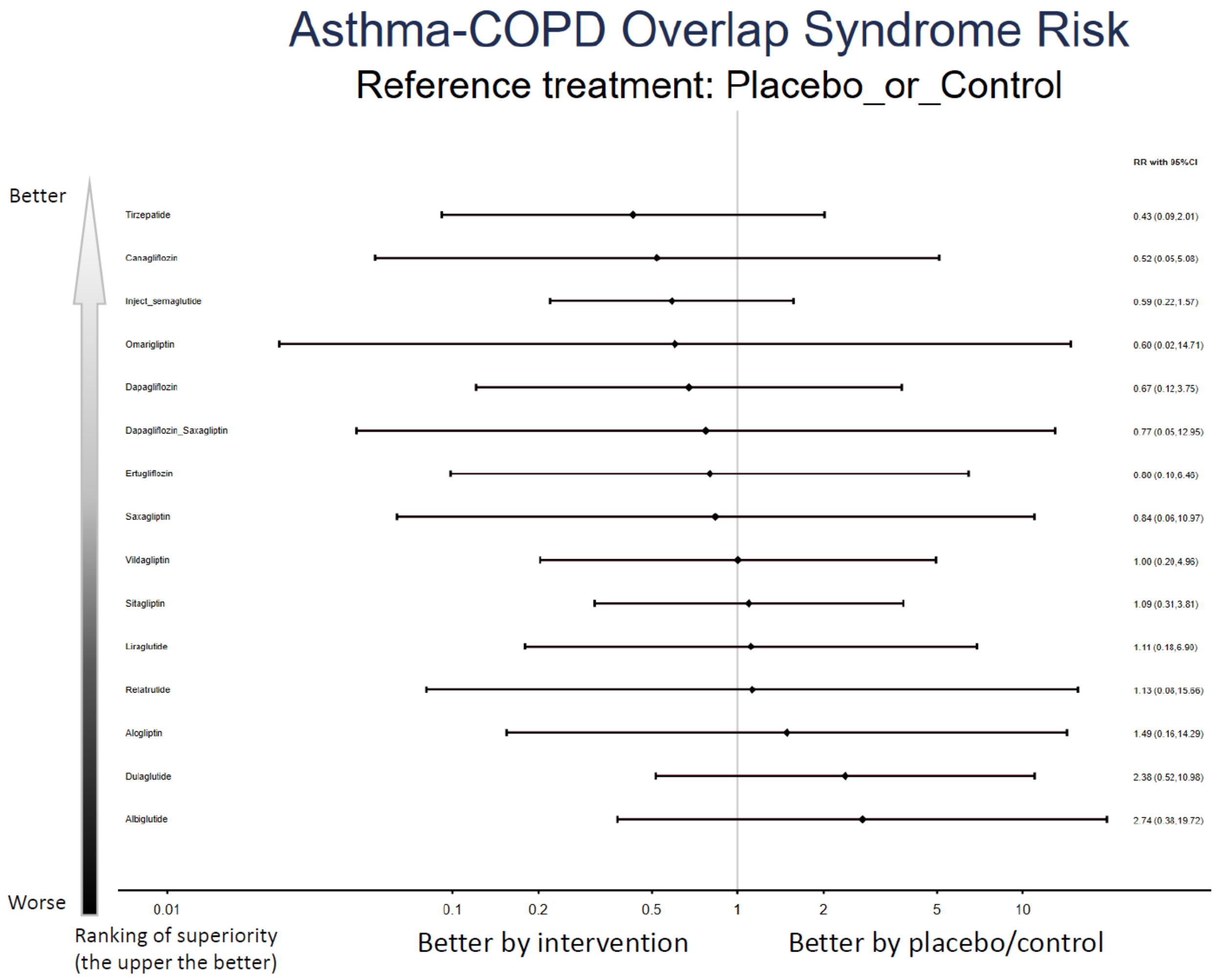

eFigure 2G Forest plot of primary outcome: overall asthma-COPD overlap syndrome risk focusing on trials with treatment duration at least 1 year

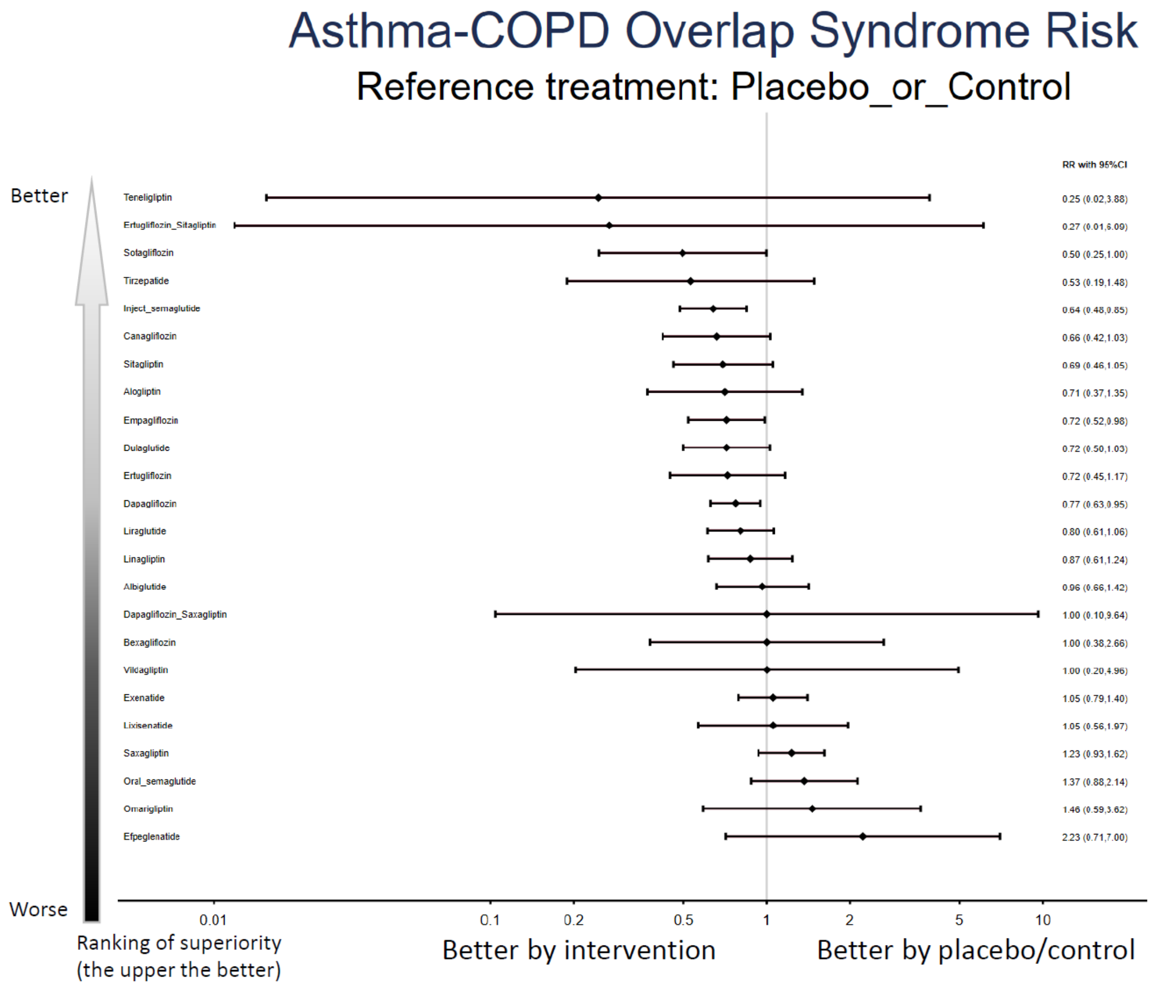

eFigure 2H Forest plot of primary outcome: overall asthma-COPD overlap syndrome risk using hazard ratio based on time-to-event data

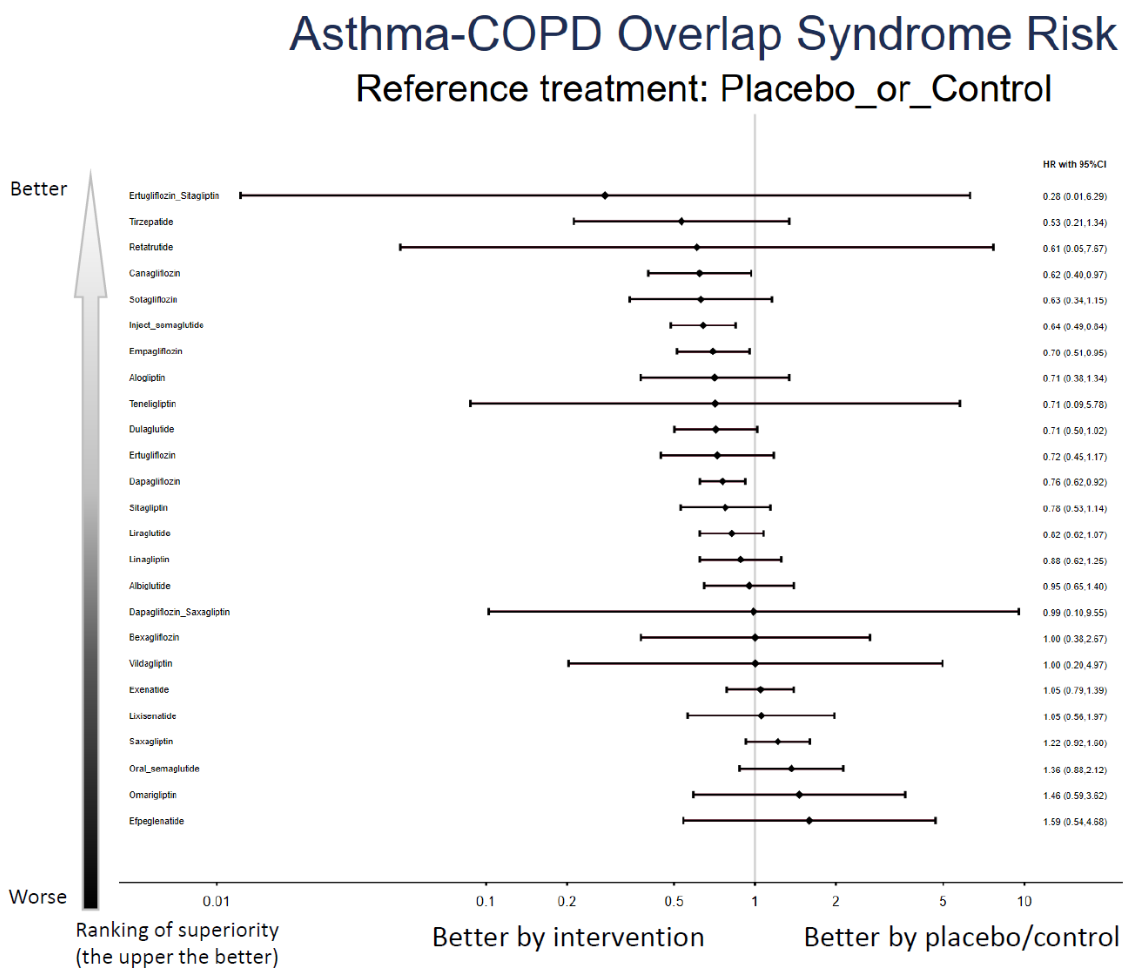

eFigure 2I Forest plot of secondary outcome: status asthmaticus in asthmatic episode

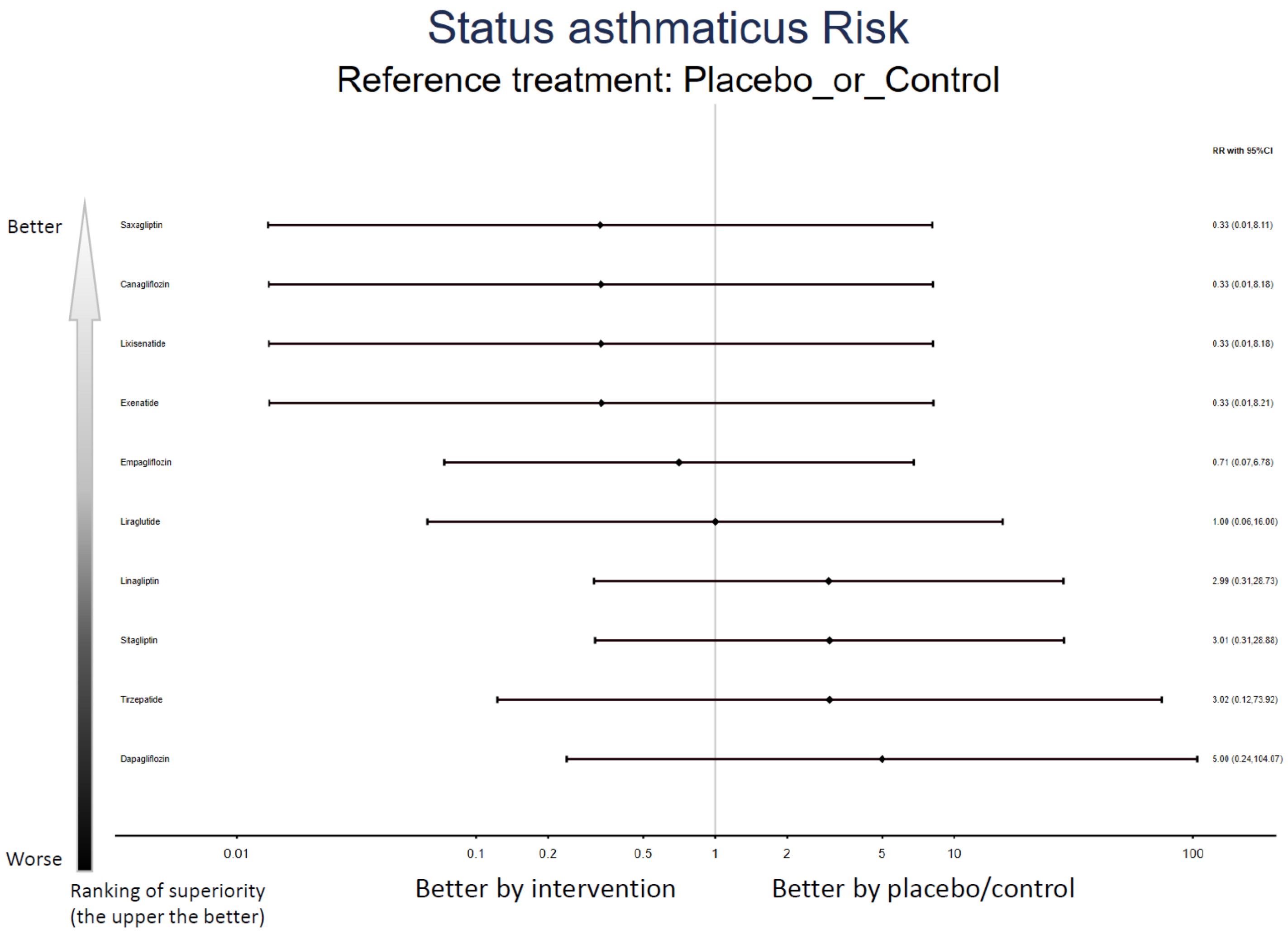

eFigure 2J Forest plot of secondary outcome: emphysema in COPD episode

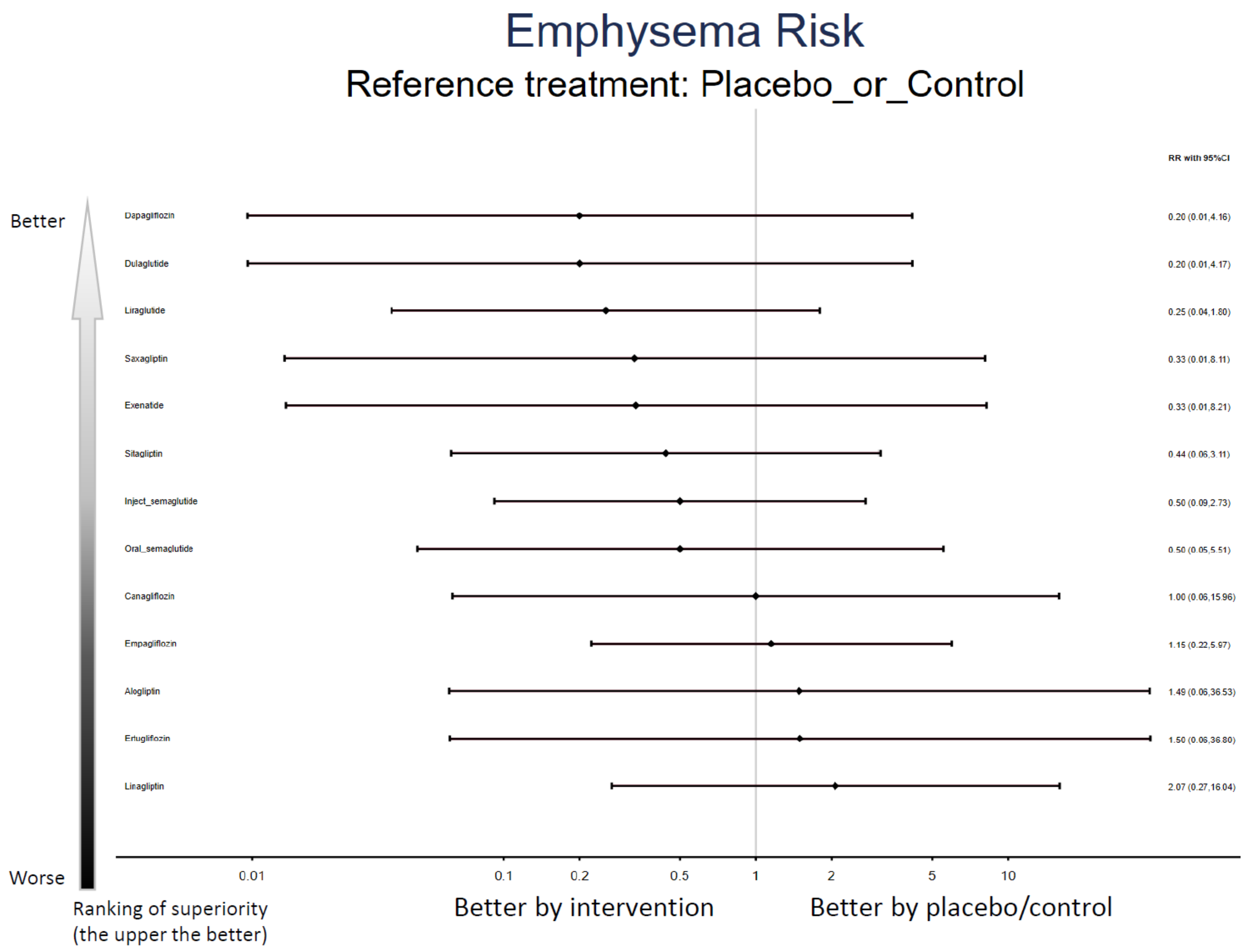

eFigure 2K Forest plot of secondary outcome: chronic bronchitis in COPD episode

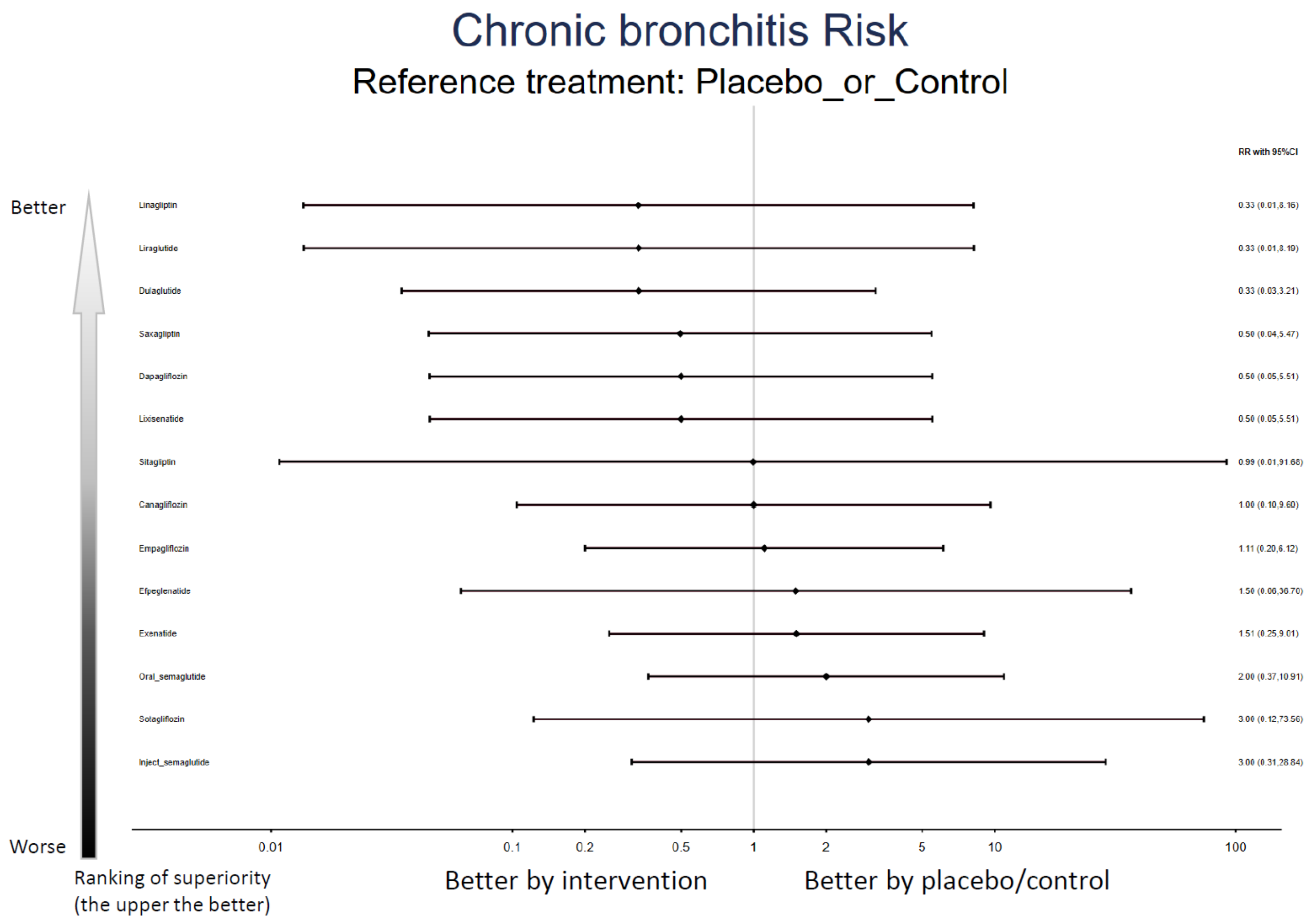

eFigure 2L Forest plot of acceptability: treatment discontinuation rate

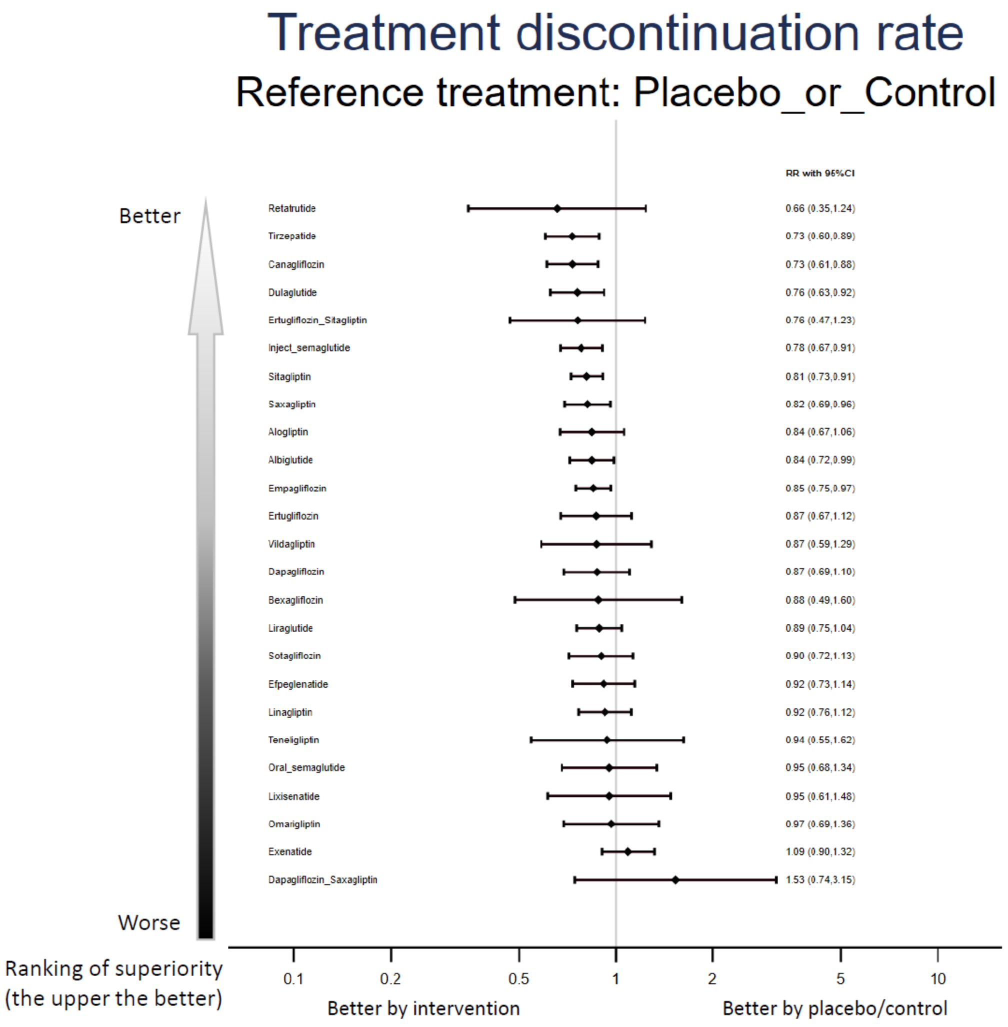

Abbreviation for eFigure 2A-2L:

95%CIs: 95% confidence intervals; DPP4 inhibitor: dipeptidyl peptidase 4 inhibitor; GLP-1 agonist: glucagon-like peptide-1 agonist; NMA: network meta-analysis; RCT: randomised controlled trial; RR: risk ratio; SGLT2 inhibitor: sodium–glucose cotransporter 2 inhibitor

eFigure 3 Individual study result of primary outcome: overall asthma-COPD overlap syndrome risk

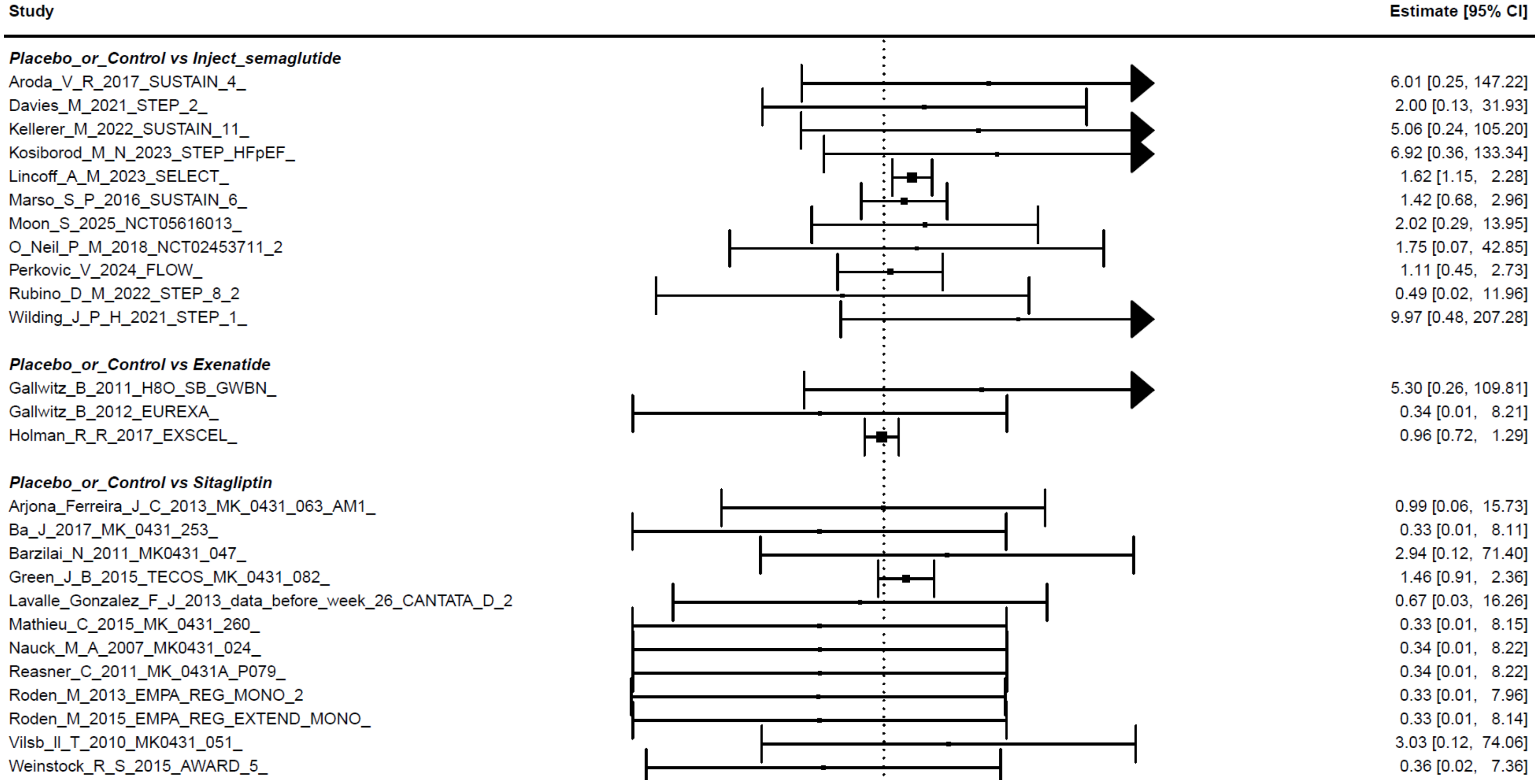

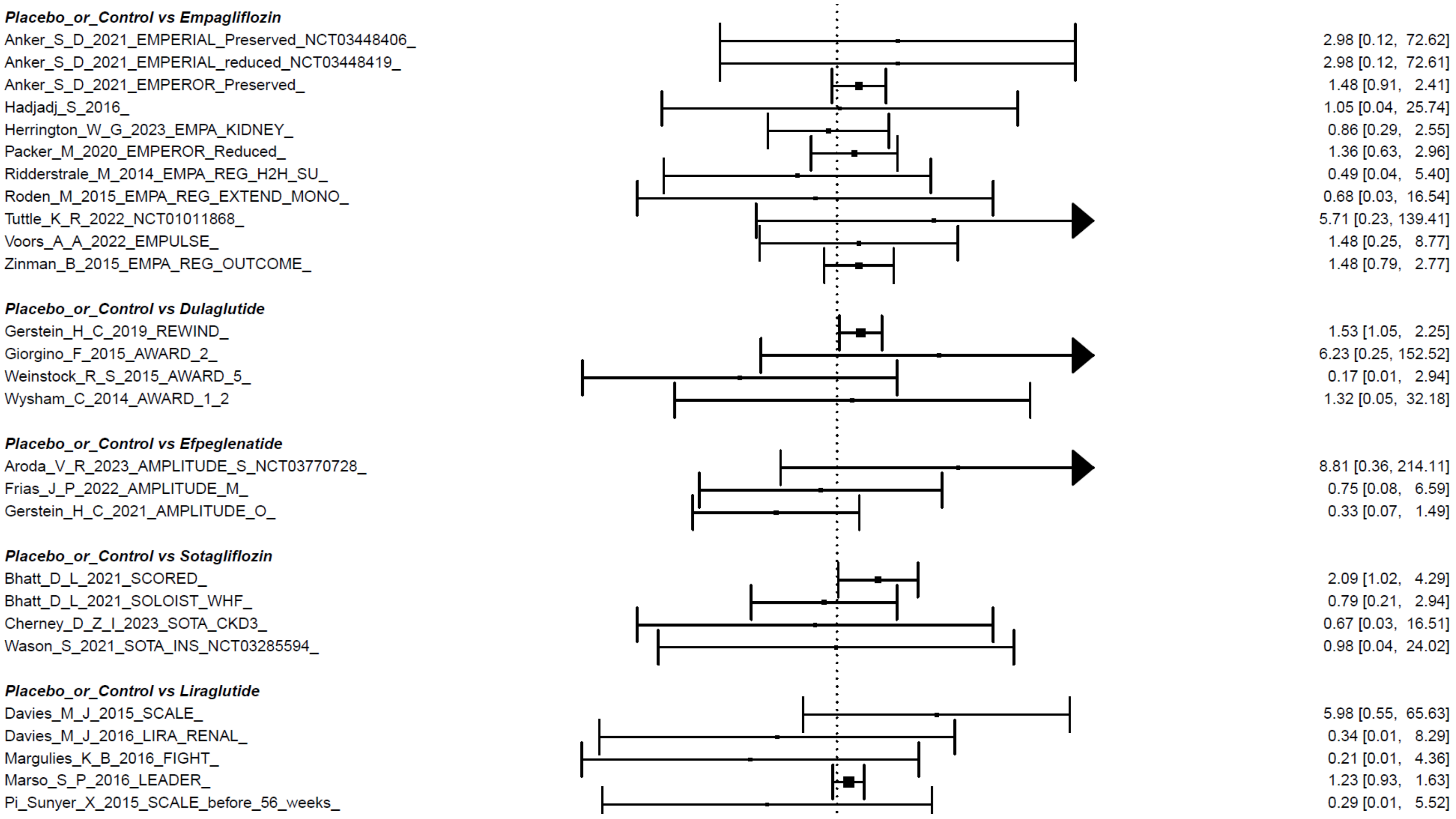

Placebo_or_Control vs Empagliflozin
Anker_S_D_2021_EMPERIAL_Preserved_NCT03448406_ 2.98 [0.12, 72.62]
Anker_S_D_2021_EMPERIAL_reduced_NCT03448419_ 2.98 [0.12, 72.61]
Anker_S_D_2021_EMPEROR_Preserved_ 1.48 [0.91, 2.41]
Hadjadj_S_2016_ 1.05 [0.04, 25.74]
Herrington_W_G_2023_EMPA_KIDNEY_ 0.86 [0.29, 2.55]
Packer_M_2020_EMPEROR_Reduced_ 1.36 [0.63, 2.96]
Ridderstrale_M_2014_EMPA_REG_H2H_SU_ 0.49 [0.04, 5.40]
Roden_M_2015_EMPA_REG_EXTEND_MONO_ 0.68 [0.03, 16.54]
Tuttle_K_R_2022_NCT01011868_ 5.71 [0.23, 139.41]
Voors_A_A_2022_EMPULSE_ 1.48 [0.25, 8.77]
Zinman_B_2015_EMPA_REG_OUTCOME_ 1.48 [0.79, 2.77]
Placebo_or_Control vs Dulaglutide
Gerstein_H_C_2019_REWIND_ 1.53 [1.05, 2.25]
Giorgino_F_2015_AWARD_2_ 6.23 [0.25, 152.52]
Weinstock_R_S_2015_AWARD_5_ 0.17 [0.01, 2.94]
Wysham_C_2014_AWARD_1_2 1.32 [0.05, 32.18]
Placebo_or_Control vs Efpeglenatide
Aroda_V_R_2023_AMPLITUDE_S_NCT03770728_ 8.81 [0.36, 214.11]
Frias_J_P_2022_AMPLITUDE_M_ 0.75 [0.08, 6.59]
Gerstein_H_C_2021_AMPLITUDE_O_ 0.33 [0.07, 1.49]
Placebo_or_Control vs Sotagliflozin
Bhatt_D_L_2021_SCORED_ 2.09 [1.02, 4.29]
Bhatt_D_L_2021_SOLOIST_WHF_ 0.79 [0.21, 2.94]
Cherney_D_Z_I_2023_SOTA_CKD3_ 0.67 [0.03, 16.51]
Wason_S_2021_SOTA_INS_NCT03285594_ 0.98 [0.04, 24.02]
Placebo_or_Control vs Liraglutide
Davies_M_J_2015_SCALE_ 5.98 [0.55, 65.63]
Davies_M_J_2016_LIRA_RENAL_ 0.34 [0.01, 8.29]
Margulies_K_B_2016_FIGHT_ 0.21 [0.01, 4.36]
Marso_S_P_2016_LEADER_ 1.23 [0.93, 1.63]
Pi_Sunyer_X_2015_SCALE_before_56_weeks_ 0.29 [0.01, 5.52]

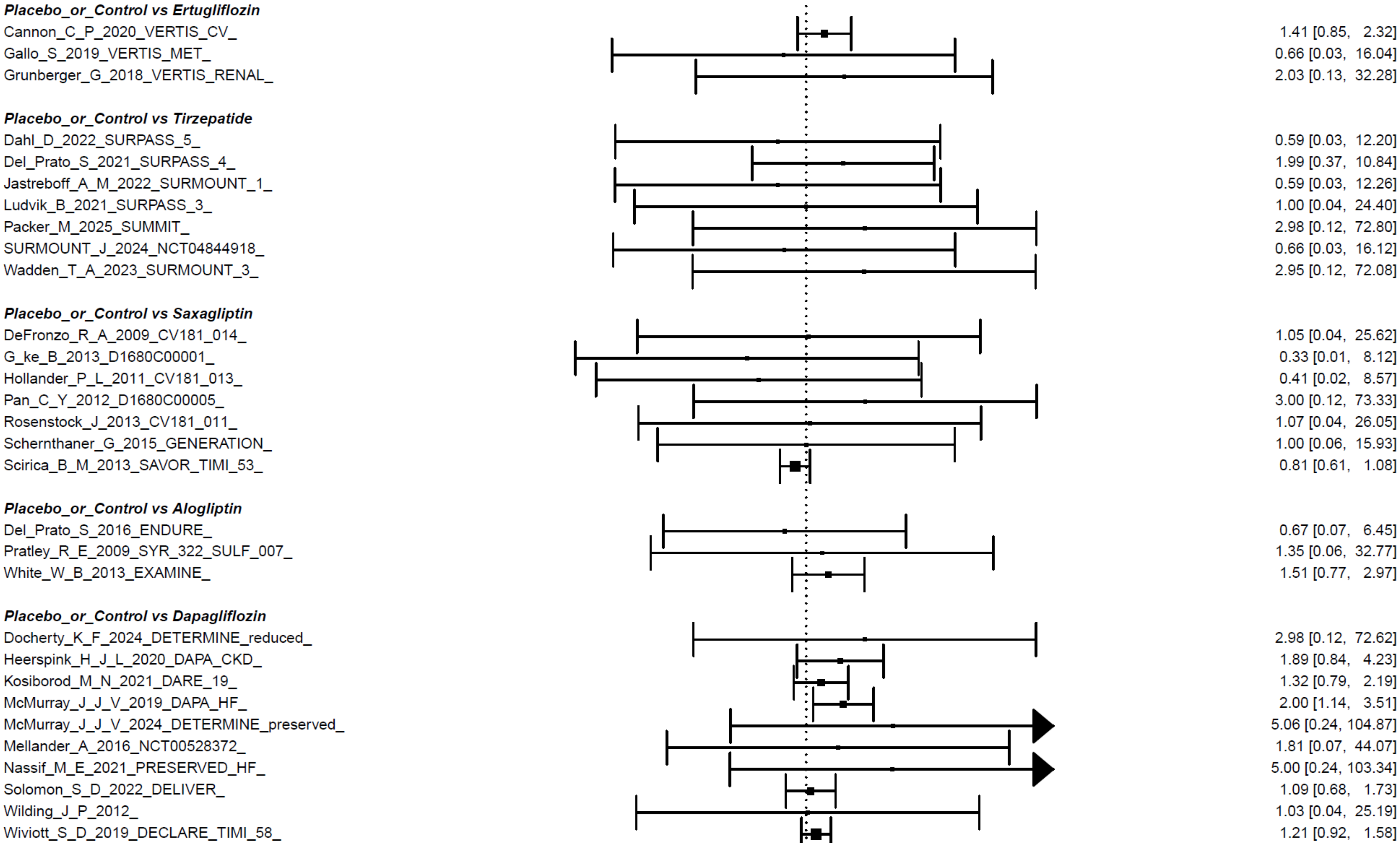
Placebo_or_Control vs Ertugliflozin
Cannon_C_P_2020_VERTIS_CV_ 1.41 [0.85, 2.32]
Gallo_S_2019_VERTIS_MET_ 0.66 [0.03, 16.04]
Grunberger_G_2018_VERTIS_RENAL_ 2.03 [0.13, 32.28]
Placebo_or_Control vs Tirzepatide
Dahl_D_2022_SURPASS_5_ 0.59 [0.03, 12.20]
Del_Prato_S_2021_SURPASS_4_ 1.99 [0.37, 10.84]
Jastreboff_A_M_2022_SURMOUNT_1_ 0.59 [0.03, 12.26]
Ludvik_B_2021_SURPASS_3_ 1.00 [0.04, 24.40]
Packer_M_2025_SUMMIT_ 2.98 [0.12, 72.80]
SURMOUNT_J_2024_NCT04844918_ 0.66 [0.03, 16.12]
Wadden_T_A_2023_SURMOUNT_3_ 2.95 [0.12, 72.08]
Placebo_or_Control vs Saxagliptin
DeFronzo_R_A_2009_CV181_014_ 1.05 [0.04, 25.62]
G_ke_B_2013_D1680C00001_ 0.33 [0.01, 8.12]
Hollander_P_L_2011_CV181_013_ 0.41 [0.02, 8.57]
Pan_C_Y_2012_D1680C00005_ 3.00 [0.12, 73.33]
Rosenstock_J_2013_CV181_011_ 1.07 [0.04, 26.05]
Schernthaner_G_2015_GENERATION_ 1.00 [0.06, 15.93]
Scirica_B_M_2013_SAVOR_TIMI_53_ 0.81 [0.61, 1.08]
Placebo_or_Control vs Alogliptin
Del_Prato_S_2016_ENDURE_ 0.67 [0.07, 6.45]
Pratley_R_E_2009_SYR_322_SULF_007_ 1.35 [0.06, 32.77]
White_W_B_2013_EXAMINE_ 1.51 [0.77, 2.97]
Placebo_or_Control vs Dapagliflozin
Docherty_K_F_2024_DETERMINE_reduced_ 2.98 [0.12, 72.62]
Heerspink_H_J_L_2020_DAPA_CKD_ 1.89 [0.84, 4.23]
Kosiborod_M_N_2021_DARE_19_ 1.32 [0.79, 2.19]
McMurray_J_J_V_2019_DAPA_HF_ 2.00 [1.14, 3.51]
McMurray_J_J_V_2024_DETERMINE_preserved_ 5.06 [0.24, 104.87]
Mellander_A_2016_NCT00528372_ 1.81 [0.07, 44.07]
Nassif_M_E_2021_PRESERVED_HF_ 5.00 [0.24, 103.34]
Solomon_S_D_2022_DELIVER_ 1.09 [0.68, 1.73]
Wilding_J_P_2012_ 1.03 [0.04, 25.19]
Wiviott_S_D_2019_DECLARE_TIMI_58_ 1.21 [0.92, 1.58]

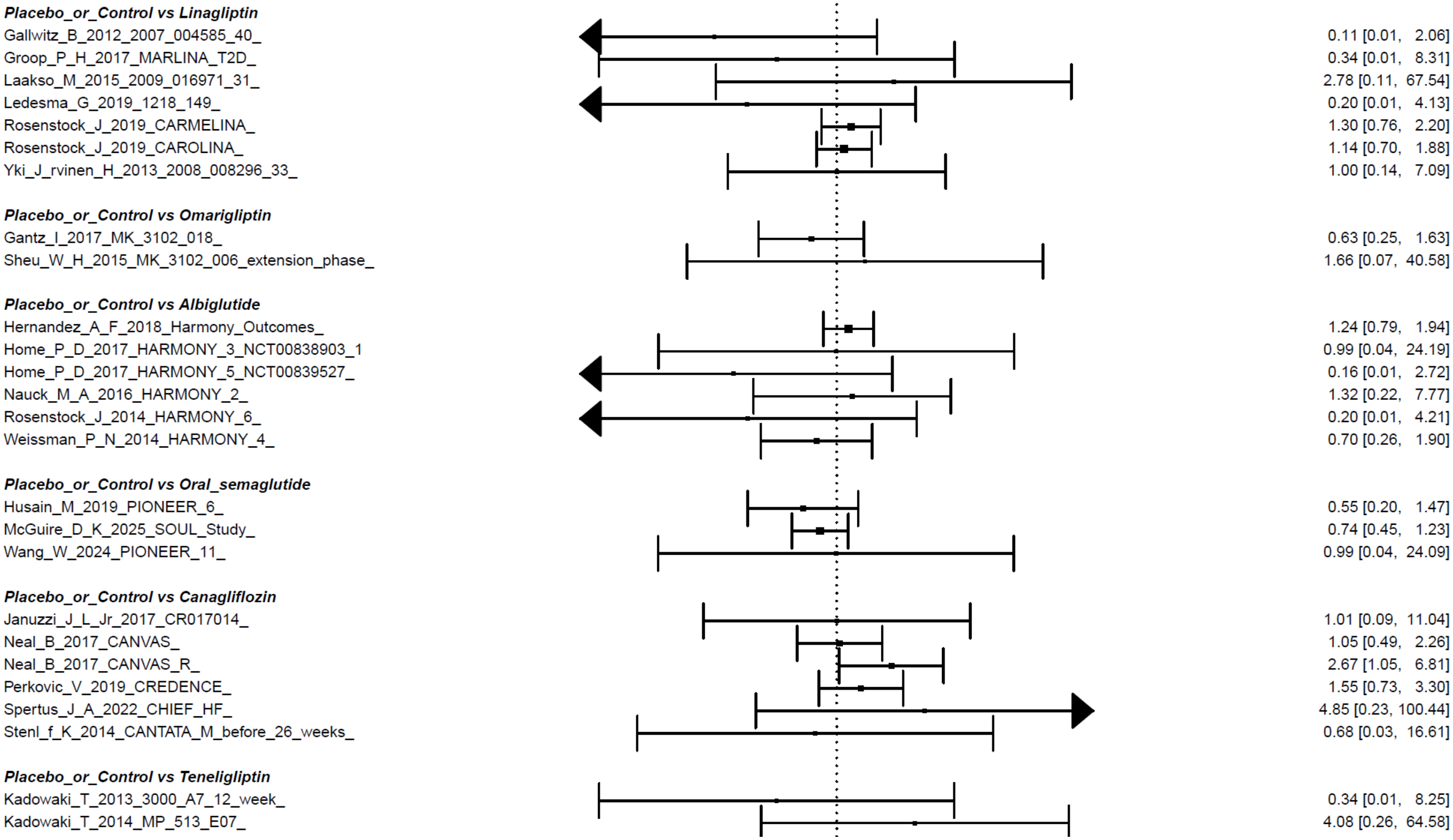
Placebo_or_Control vs Linagliptin
Gallwitz_B_2012_2007_004585_40_ 0.11 [0.01, 2.06]
Groop_P_H_2017_MARLINA_T2D_ 0.34 [0.01, 8.31]
Laakso_M_2015_2009_016971_31_ 2.78 [0.11, 67.54]
Ledesma_G_2019_1218_149_ 0.20 [0.01, 4.13]
Rosenstock_J_2019_CARMELINA_ 1.30 [0.76, 2.20]
Rosenstock_J_2019_CAROLINA_ 1.14 [0.70, 1.88]
Yki_J_rvinen_H_2013_2008_008296_33_ 1.00 [0.14, 7.09]
Placebo_or_Control vs Omarigliptin
Gantz_I_2017_MK_3102_018_ 0.63 [0.25, 1.63]
Sheu_W_H_2015_MK_3102_006_extension_phase_ 1.66 [0.07, 40.58]
Placebo_or_Control vs Albiglutide
Hernandez_A_F_2018_Harmony_Outcomes_ 1.24 [0.79, 1.94]
Home_P_D_2017_HARMONY_3_NCT00838903_1 0.99 [0.04, 24.19]
Home_P_D_2017_HARMONY_5_NCT00839527_ 0.16 [0.01, 2.72]
Nauck_M_A_2016_HARMONY_2_ 1.32 [0.22, 7.77]
Rosenstock_J_2014_HARMONY_6_ 0.20 [0.01, 4.21]
Weissman_P_N_2014_HARMONY_4_ 0.70 [0.26, 1.90]
Placebo_or_Control vs Oral_semaglutide
Husain_M_2019_PIONEER_6_ 0.55 [0.20, 1.47]
McGuire_D_K_2025_SOUL_Study_ 0.74 [0.45, 1.23]
Wang_W_2024_PIONEER_11_ 0.99 [0.04, 24.09]
Placebo_or_Control vs Canagliflozin
Januzzi_J_L_Jr_2017_CR017014_ 1.01 [0.09, 11.04]
Neal_B_2017_CANVAS_ 1.05 [0.49, 2.26]
Neal_B_2017_CANVAS_R_ 2.67 [1.05, 6.81]
Perkovic_V_2019_CREDENCE_ 1.55 [0.73, 3.30]
Spertus_J_A_2022_CHIEF_HF_ 4.85 [0.23, 100.44]
Stenl_f_K_2014_CANTATA_M_before_26_weeks_ 0.68 [0.03, 16.61]
Placebo_or_Control vs Teneligliptin
Kadowaki_T_2013_3000_A7_12_week_ 0.34 [0.01, 8.25]
Kadowaki_T_2014_MP_513_E07_ 4.08 [0.26, 64.58]

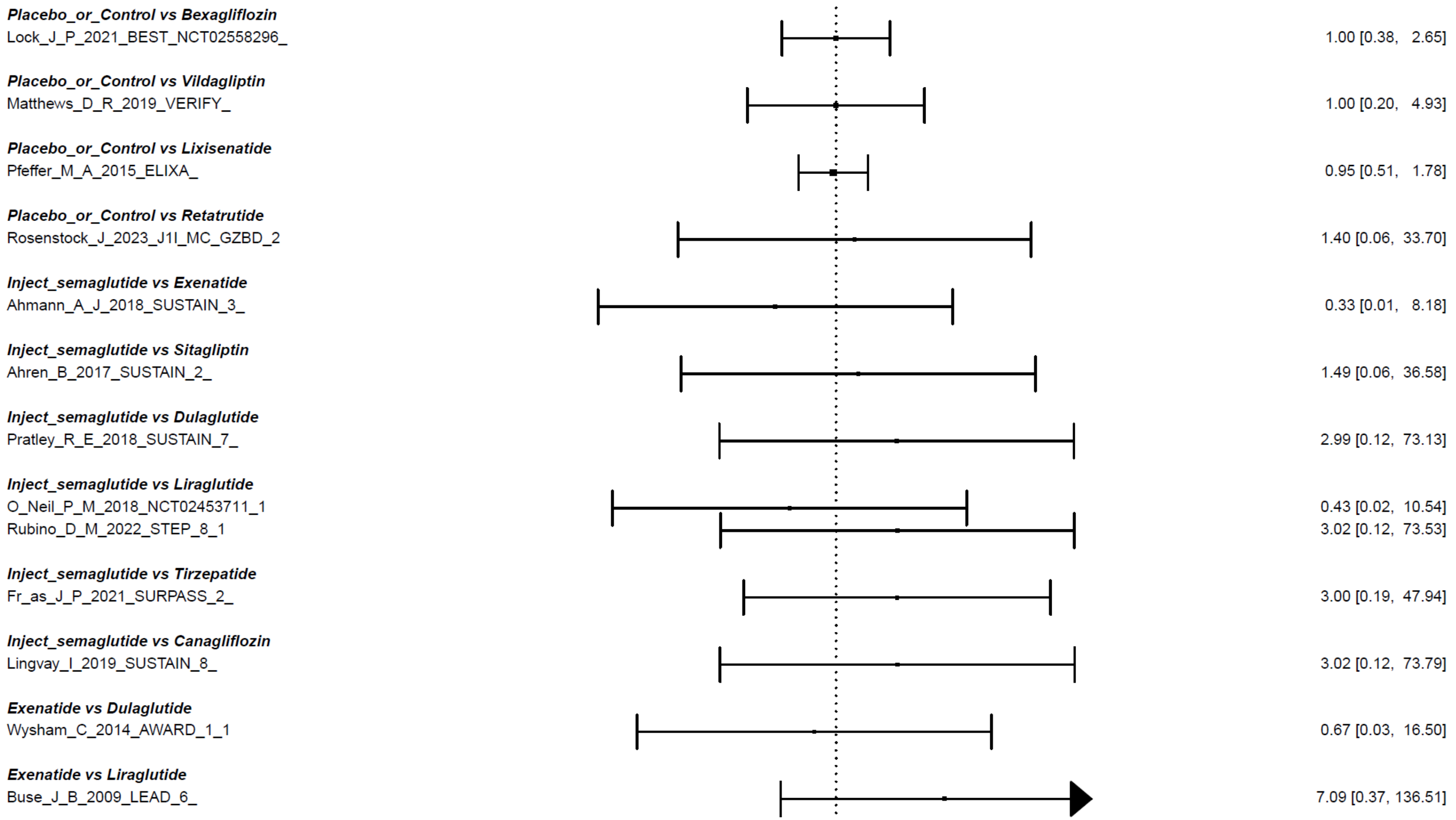
Placebo_or_Control vs Bexagliflozin
Lock_J_P_2021_BEST_NCT02558296_
1.00 [0.38, 2.65]
Placebo_or_Control vs Vildagliptin
Matthews_D_R_2019_VERIFY_
1.00 [0.20, 4.93]
Placebo_or_Control vs Lixisenatide
Pfeffer_M_A_2015_ELIXA_
0.95 [0.51, 1.78]
Placebo_or_Control vs Retatrutide
Rosenstock_J_2023_J1I_MC_GZBD_2
1.40 [0.06, 33.70]
Inject_semaglutide vs Exenatide
Ahmann_A_J_2018_SUSTAIN_3_
0.33 [0.01, 8.18]
Inject_semaglutide vs Sitagliptin
Ahren_B_2017_SUSTAIN_2_
1.49 [0.06, 36.58]
Inject_semaglutide vs Dulaglutide
Pratley_R_E_2018_SUSTAIN_7_
2.99 [0.12, 73.13]
Inject_semaglutide vs Liraglutide
O_Neil_P_M_2018_NCT02453711_1
0.43 [0.02, 10.54]
Rubino_D_M_2022_STEP_8_1
3.02 [0.12, 73.53]
Inject_semaglutide vs Tirzepatide
Fr_as_J_P_2021_SURPASS_2_
3.00 [0.19, 47.94]
Inject_semaglutide vs Canagliflozin
Lingvay_I_2019_SUSTAIN_8_
3.02 [0.12, 73.79]
Exenatide vs Dulaglutide
Wysham_C_2014_AWARD_1_1
0.67 [0.03, 16.50]
Exenatide vs Liraglutide
Buse_J_B_2009_LEAD_6_
7.09 [0.37, 136.51]

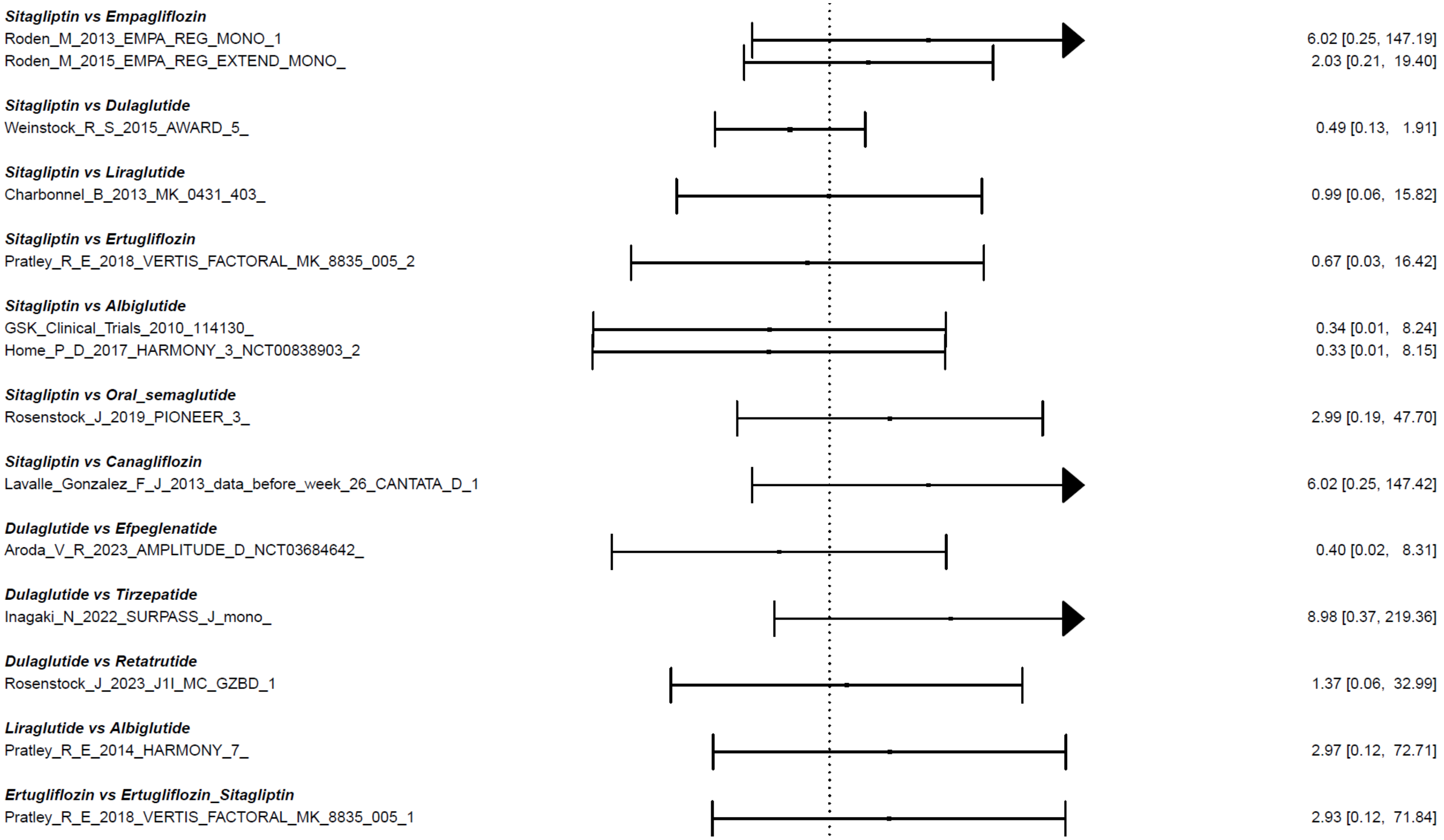
Sitagliptin vs Empagliflozin
Roden_M_2013_EMPA_REG_MONO_1
6.02 [0.25, 147.19]
Roden_M_2015_EMPA_REG_EXTEND_MONO_
2.03 [0.21, 19.40]
Sitagliptin vs Dulaglutide
Weinstock_R_S_2015_AWARD_5_
0.49 [0.13, 1.91]
Sitagliptin vs Liraglutide
Charbonnel_B_2013_MK_0431_403_
0.99 [0.06, 15.82]
Sitagliptin vs Ertugliflozin
Pratley_R_E_2018_VERTIS_FACTORAL_MK_8835_005_2
0.67 [0.03, 16.42]
Sitagliptin vs Albiglutide
GSK_Clinical_Trials_2010_114130_
0.34 [0.01, 8.24]
Home_P_D_2017_HARMONY_3_NCT00838903_2
0.33 [0.01, 8.15]
Sitagliptin vs Oral_semaglutide
Rosenstock_J_2019_PIONEER_3_
2.99 [0.19, 47.70]
Sitagliptin vs Canagliflozin
Lavalle_Gonzalez_F_J_2013_data_before_week_26_CANTATA_D_1
6.02 [0.25, 147.42]
Dulaglutide vs Efpeglenatide
Aroda_V_R_2023_AMPLITUDE_D_NCT03684642_
0.40 [0.02, 8.31]
Dulaglutide vs Tirzepatide
Inagaki_N_2022_SURPASS_J_mono_
8.98 [0.37, 219.36]
Dulaglutide vs Retatrutide
Rosenstock_J_2023_J1I_MC_GZBD_1
1.37 [0.06, 32.99]
Liraglutide vs Albiglutide
Pratley_R_E_2014_HARMONY_7_
2.97 [0.12, 72.71]
Ertugliflozin vs Ertugliflozin_Sitagliptin
Pratley_R_E_2018_VERTIS_FACTORAL_MK_8835_005_1
2.93 [0.12, 71.84]

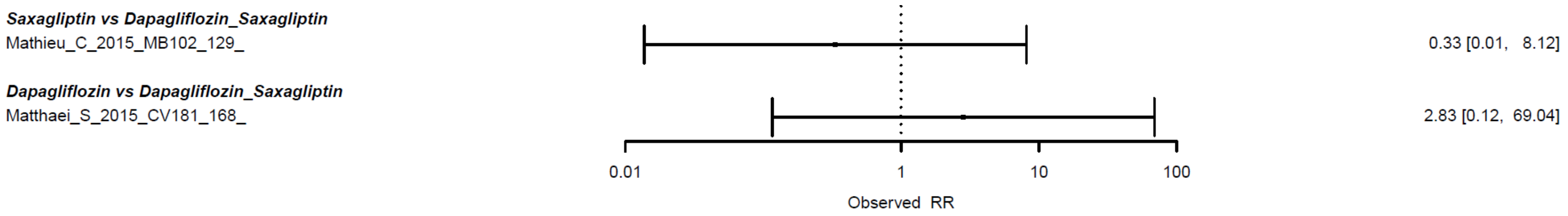

Abbreviation for eFigure 3:

95%CIs: 95% confidence intervals; DPP4 inhibitor: dipeptidyl peptidase 4 inhibitor; GLP-1 agonist: glucagon-like peptide-1 agonist; NMA: network meta-analysis; RCT: randomised controlled trial; RR: risk ratio; SGLT2 inhibitor: sodium–glucose cotransporter 2 inhibitor

eFigure 4A Funnel plot for primary outcome: overall asthma-COPD overlap syndrome risk

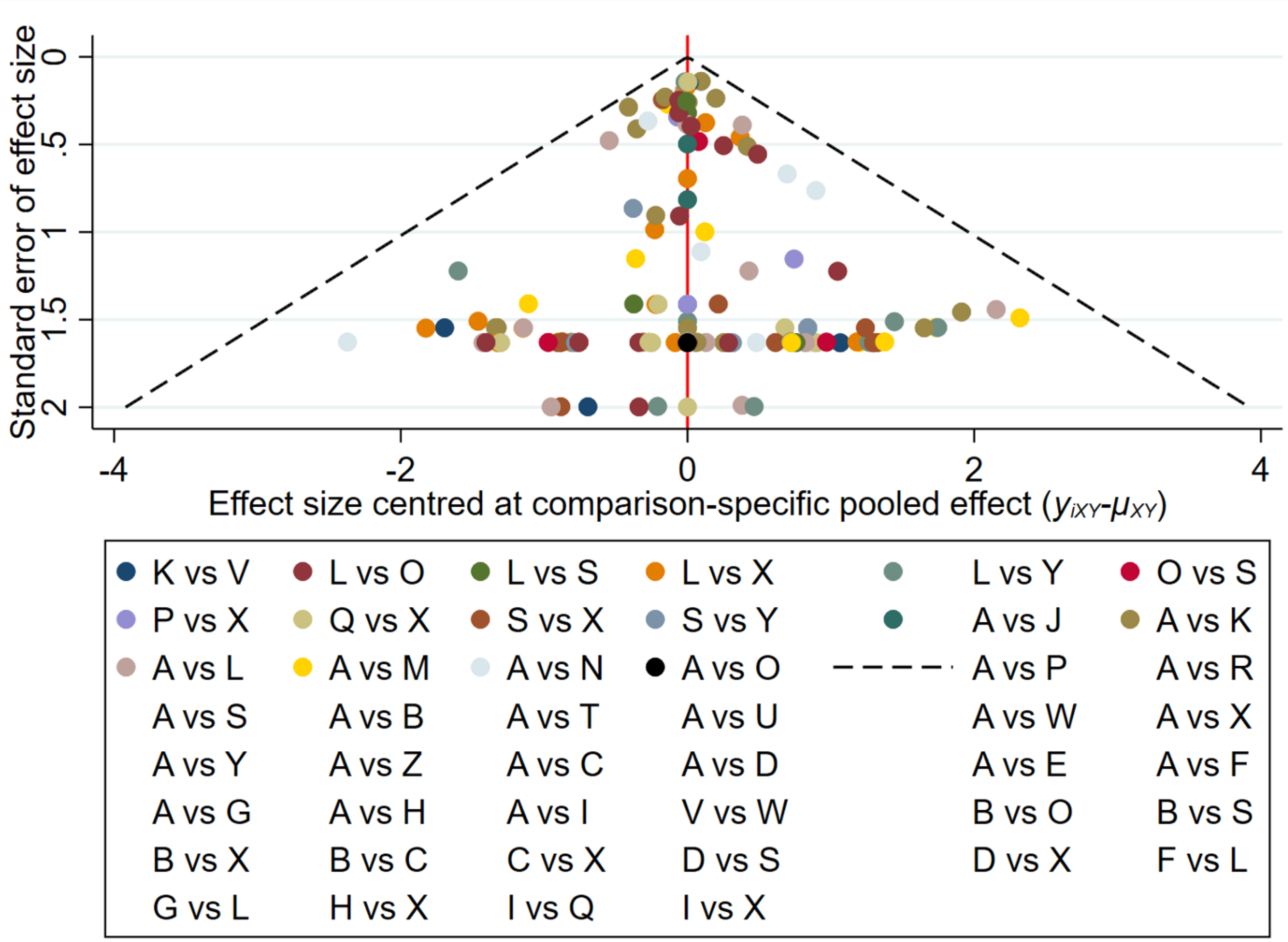

Treatments used

A:	Placebo_or_Control
B:	Liraglutide
C:	Albiglutide
D:	Canagliflozin
E:	Teneligliptin
F:	Efpeglenatide
G:	Retatrutide
H:	Empagliflozin
I:	Ertugliflozin
J:	Vildagliptin
K:	Dapagliflozin
L:	Dulaglutide
M:	Linagliptin
N:	Sotagliflozin
O:	Exenatide
P:	Oral_semaglutide
Q:	Ertugliflozin_Sitagliptin
R:	Lixisenatide
S:	Inject_semaglutide
T:	Omarigliptin
U:	Alogliptin
V:	Dapagliflozin_Saxagliptin
W:	Saxagliptin
X:	Sitagliptin
Y:	Tirzepatide
Z:	Bexagliflozin

eFigure 4B Funnel plot for primary outcome: subgroup of asthma risk

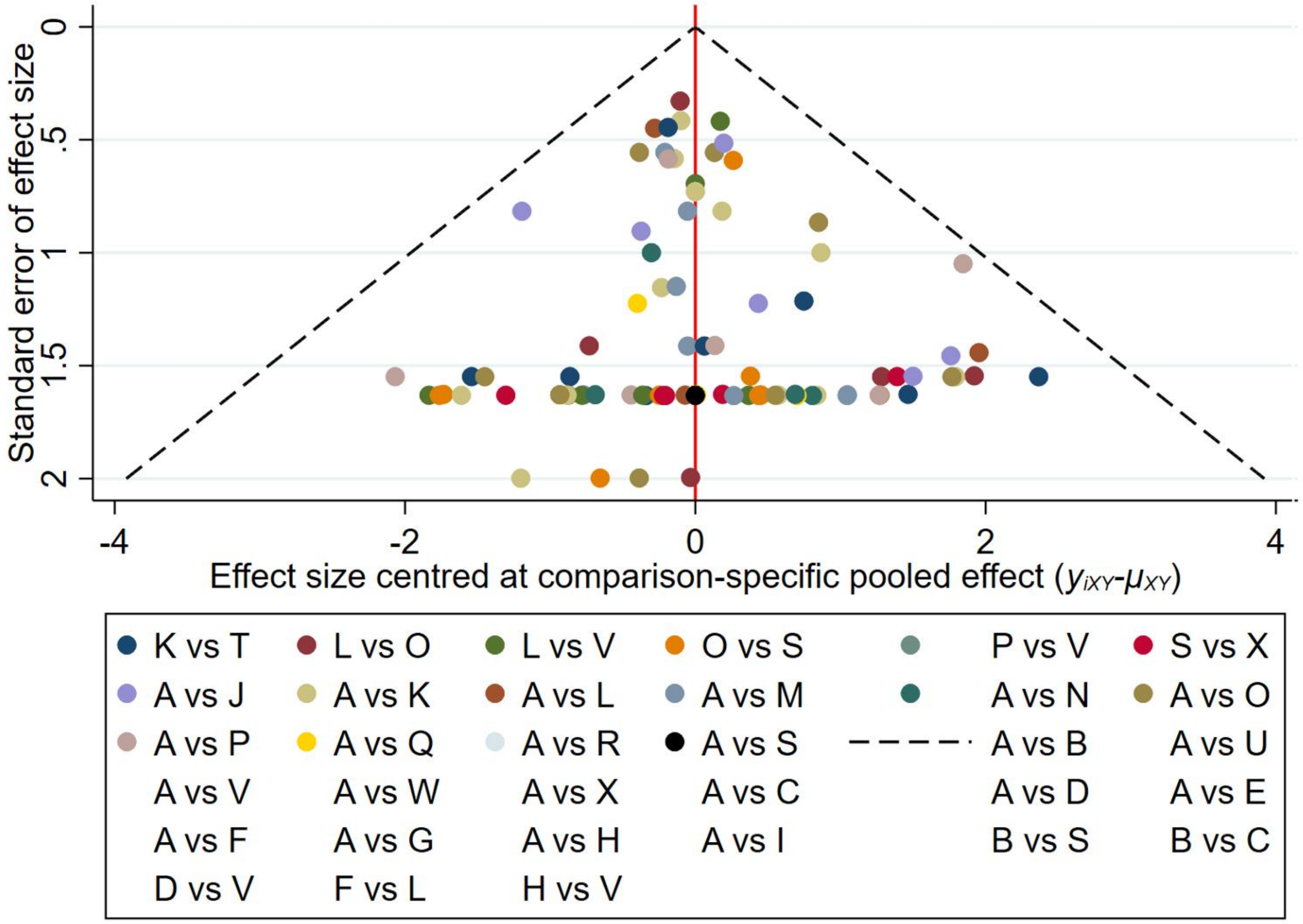

Treatments used

A:	Placebo_or_Control
B:	Liraglutide
C:	Albiglutide
D:	Canagliflozin
E:	Vildagliptin
F:	Efpeglenatide
G:	Teneligliptin
H:	Empagliflozin
I:	Ertugliflozin
J:	Omarigliptin
K:	Dapagliflozin
L:	Dulaglutide
M:	Linagliptin
N:	Sotagliflozin
O:	Exenatide
P:	Oral_semaglutide
Q:	Alogliptin
R:	Lixisenatide
S:	Inject_semaglutide
T:	Dapagliflozin_Saxagliptin
U:	Saxagliptin
V:	Sitagliptin
W:	Bexagliflozin
X:	Tirzepatide

eFigure 4C Funnel plot for primary outcome: subgroup of COPD risk

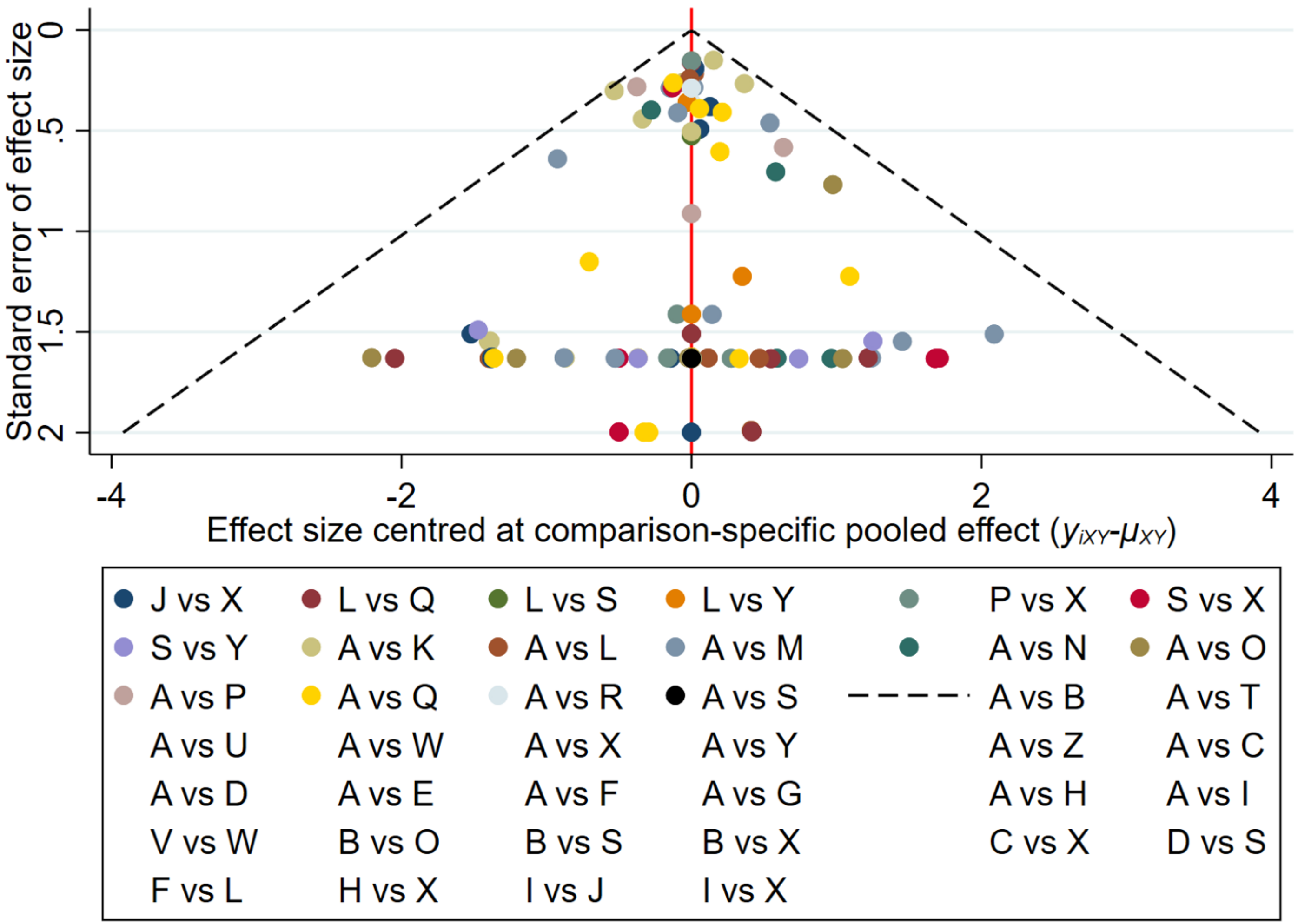

Treatments used

A:	Placebo_or_Control
B:	Liraglutide
C:	Albiglutide
D:	Canagliflozin
E:	Teneligliptin
F:	Efpeglenatide
G:	Vildagliptin
H:	Empagliflozin
I:	Ertugliflozin
J:	Ertugliflozin_Sitagliptin
K:	Dapagliflozin
L:	Dulaglutide
M:	Linagliptin
N:	Sotagliflozin
O:	Exenatide
P:	Oral_semaglutide
Q:	Retatrutide
R:	Lixisenatide
S:	Inject_semaglutide
T:	Omarigliptin
U:	Alogliptin
V:	Dapagliflozin_Saxagliptin
W:	Saxagliptin
X:	Sitagliptin
Y:	Tirzepatide
Z:	Bexagliflozin

Abbreviation for eFigure 4A-4C:

95%CIs: 95% confidence intervals; DPP4 inhibitor: dipeptidyl peptidase 4 inhibitor; GLP-1 agonist: glucagon-like peptide-1 agonist; NMA: network meta-analysis; RCT: randomised controlled trial; RR: risk ratio; SGLT2 inhibitor: sodium–glucose cotransporter 2 inhibitor

eFigure 5A Egger test for primary outcome: overall asthma-COPD overlap syndrome risk

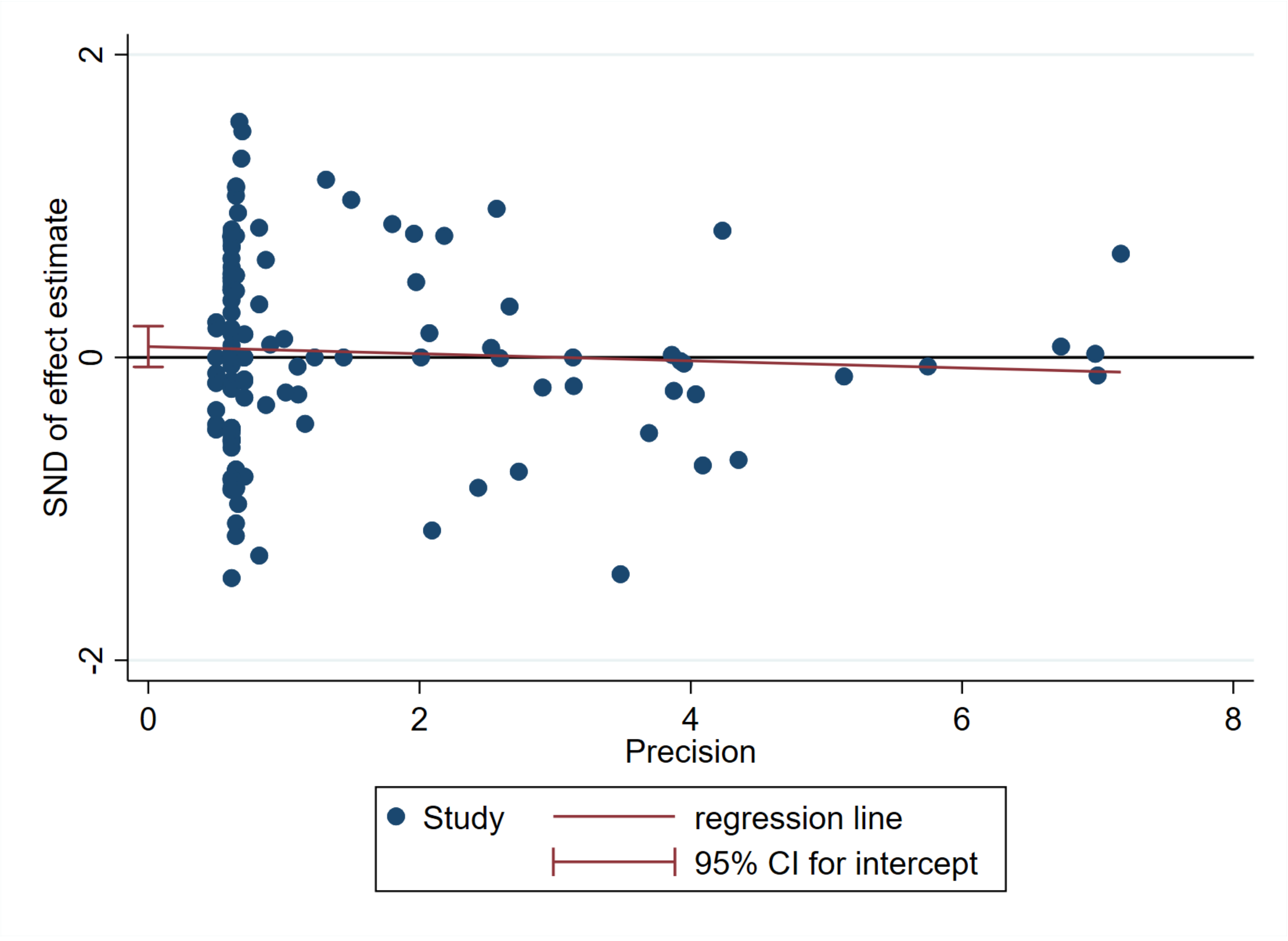

eFigure 5B Egger test for primary outcome: subgroup of asthma risk

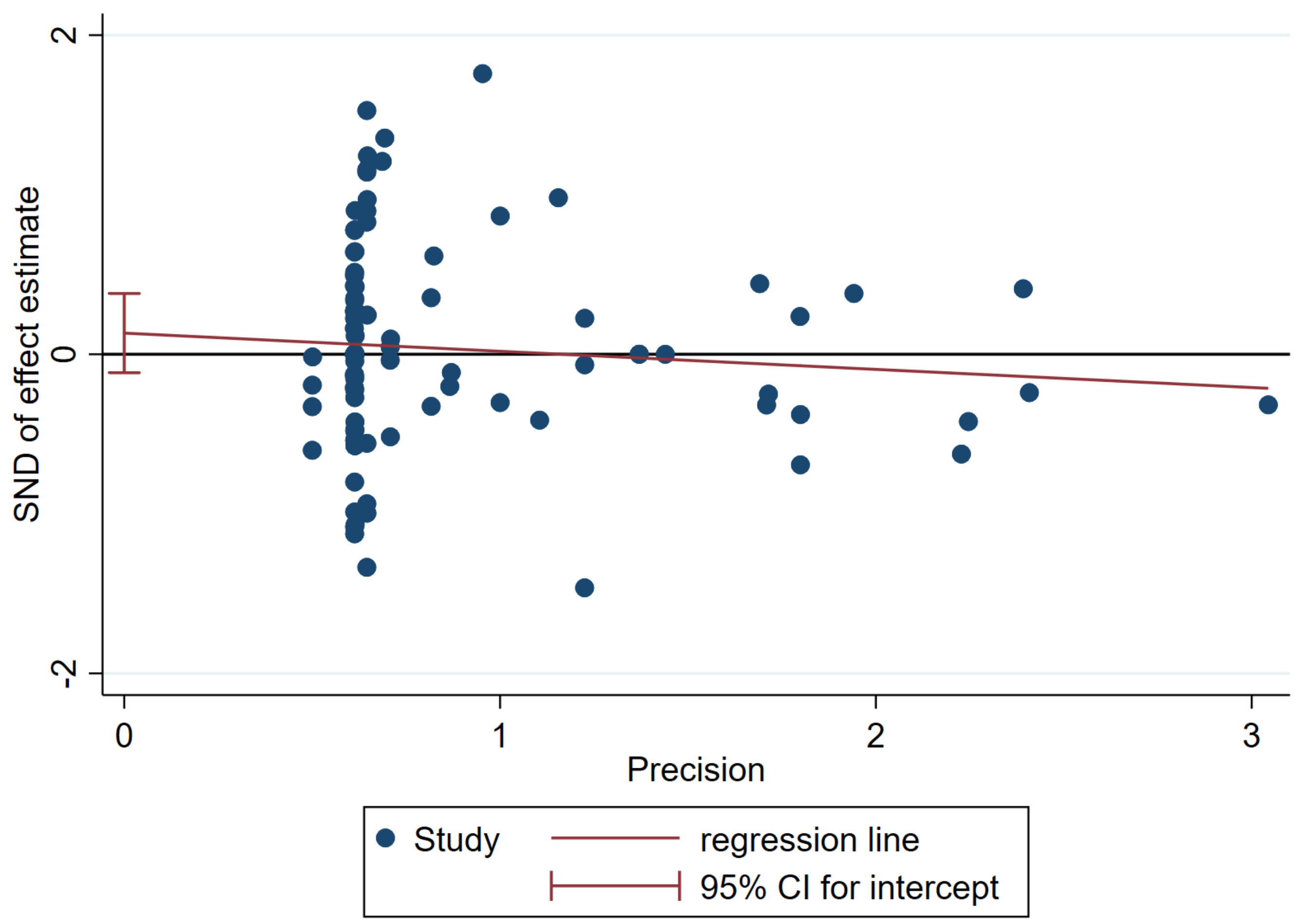

eFigure 5C Egger test for primary outcome: subgroup of COPD risk

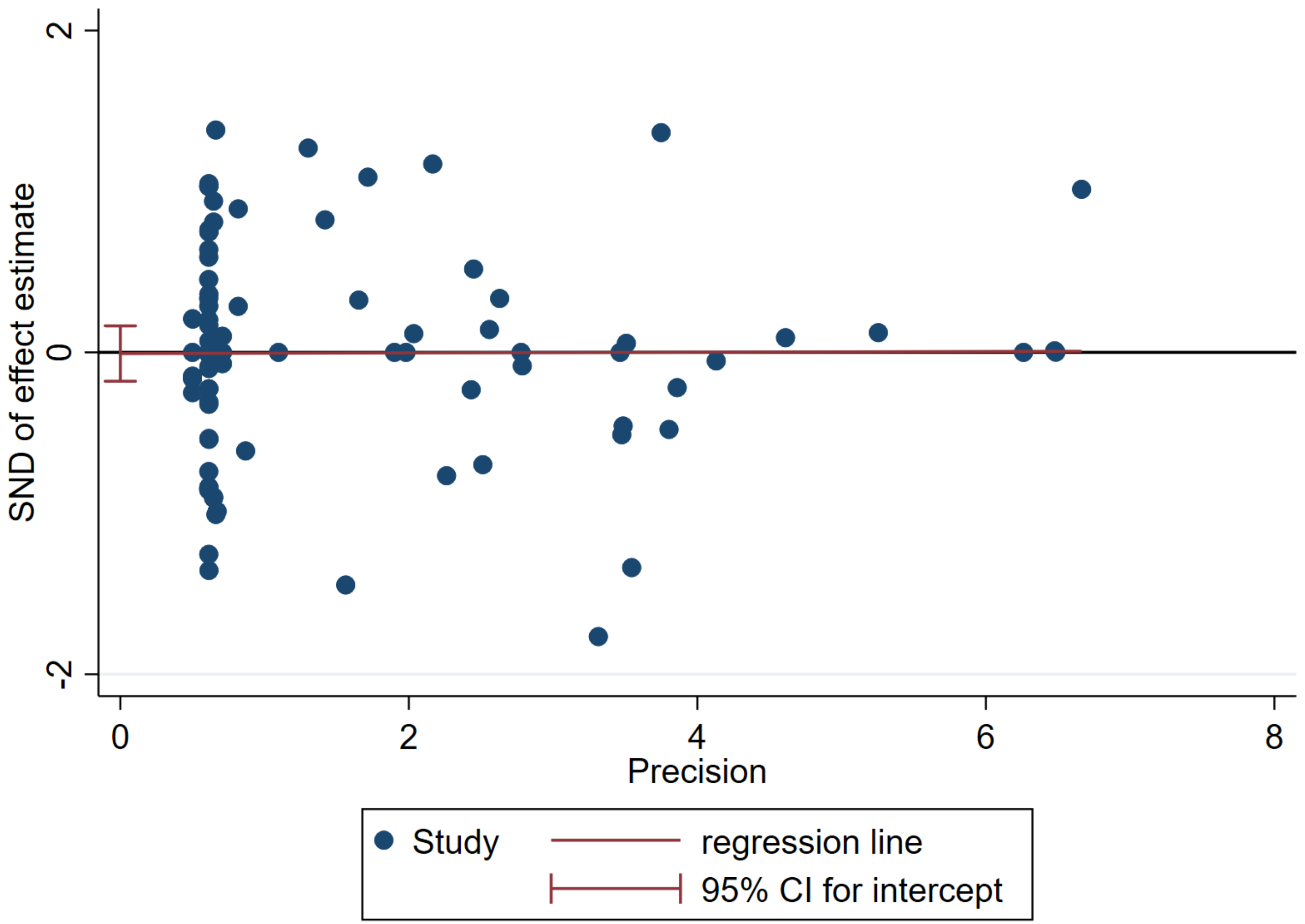

Abbreviation for eFigure 5A-5C:

95%CIs: 95% confidence intervals; DPP4 inhibitor: dipeptidyl peptidase 4 inhibitor; GLP-1 agonist: glucagon-like peptide-1 agonist; NMA: network meta-analysis; RCT: randomised controlled trial; RR: risk ratio; SGLT2 inhibitor: sodium–glucose cotransporter 2 inhibitor

eFigure 6 Risk of bias tool 2.0

Study name	Randomization process	Intervention adherence	Missing outcome data	Outcome measurement	Selective reporting	Overall RoB
Ahmann, A. J. (2018) (SUSTAIN 3)	🟢	🟡	🟢	🟢	🟢	🟡
Ahren, B. (2017) (SUSTAIN 2)	🟢	🟢	🟡	🟢	🟢	🟡
Anker, S.D. (2021) (EMPERIAL - Preserved) (NCT03448406)	🟢	🟢	🟢	🟢	🟡	🟡
Anker, S.D. (2021) (EMPERIAL-reduced) (NCT03448419)	🟢	🟢	🟢	🟢	🟡	🟡
Anker, S.D. (2021) (EMPEROR-Preserved)	🟢	🟢	🟢	🟢	🟢	🟢
Arjona Ferreira, J.C. (2013) (MK-0431-063 AM1)	🟢	🟢	🟢	🟢	🟢	🟢
Aroda, V.R. (2017) (SUSTAIN 4)	🟢	🟡	🟢	🟢	🟢	🟡
Aroda, V.R. (2023) (AMPLITUDE-D, NCT03684642)	🟢	🟡	🟢	🟢	🟢	🟡
Aroda, V.R. (2023) (AMPLITUDE-S, NCT03770728)	🟢	🟢	🟢	🟢	🟢	🟢
Aroda, V.R. (2023) (PIONEER PLUS)	🟢	🟢	🟢	🟢	🟢	🟢
Ba, J. (2017) (MK-0431-253)	🟢	🟢	🟢	🟢	🟢	🟢
Barzilai, N. (2011) (MK0431-047)	🟢	🟡	🟡	🟡	🟡	🔴
Bhatt, D.L. (2021) (SCORED)	🟢	🟢	🟢	🟢	🔴	🔴
Bhatt, D.L. (2021) (SOLOIST-WHF)	🟢	🟢	🟢	🟢	🔴	🔴
Buse, J.B. (2009) (LEAD-6)	🟢	🟡	🔴	🟢	🟢	🔴
Cannon, C.P. (2020) (VERTIS CV)	🟢	🟢	🟢	🟢	🟢	🟢
Charbonnel, B. (2013) (MK-0431-403)	🟢	🟡	🔴	🟢	🟢	🔴
Cherney, D.Z.I.(2023) (SOTA-CKD3)	🟢	🟢	🟢	🟢	🟢	🟢
Dahl, D. (2022) (SURPASS-5)	🟢	🟢	🟢	🟢	🟢	🟢
Davies, M. (2021) (STEP 2)	🟢	🟢	🟢	🟢	🟢	🟢
Davies, M.J. (2015) (SCALE)	🟢	🟢	🟡	🟢	🟢	🟡
Davies, M.J. (2016) (LIRA-RENAL)	🟢	🟢	🟡	🟢	🟢	🟡
DeFronzo, R.A. (2009) (CV181-014)	🟢	🟢	🟢	🟢	🟢	🟢

Del Prato, S. (2016) (ENDURE)	🟡	🟢	🟡	🟢	🔴	🔴
Del Prato, S. (2021) (SURPASS-4)	🟢	🟢	🟢	🟢	🟢	🟢
Docherty, K.F. (2024) (DETERMINE-reduced)	🟢	🟢	🟢	🟢	🟢	🟢
Frías, J.P. (2021) (SURPASS-2)	🟢	🟢	🟢	🟢	🟢	🟢
Frías, J.P. (2021) (SUSTAIN FORTE)	🟢	🟢	🟢	🟢	🟢	🟢
Frias, J.P. (2022) (AMPLITUDE-M)	🟢	🟢	🟢	🟢	🟢	🟢
Gallo, S. (2019) (VERTIS MET)	🟢	🟢	🟢	🟢	🟢	🟢
Gallwitz, B. (2011) (H8O-SB-GWBN)	🟢	🟢	🟡	🟢	🟢	🟡
Gallwitz, B. (2012) (2007-004585-40)	🟢	🟢	🟡	🟢	🟢	🟡
Gallwitz, B. (2012) (EUREXA)	🟢	🟡	🟢	🟢	🟢	🟡
Gantz, I. (2017) (MK-3102-018)	🟢	🟢	🟢	🟢	🟢	🟢
Gerstein, H.C. (2019) (REWIND)	🟢	🟢	🟢	🟢	🟢	🟡
Gerstein, H.C. (2021) (AMPLITUDE-O)	🟢	🟢	🟢	🟢	🟢	🟢
Giorgino, F. (2015) (AWARD-2)	🟢	🟢	🟢	🟢	🟢	🟢
Göke, B. (2013) (D1680C00001)	🟢	🟢	🟡	🟢	🟢	🟡
Green, J.B. (2015) (TECOS - MK-0431-082)	🟢	🟢	🟢	🟢	🟢	🟢
Groop, P.H. (2017) (MARLINA-T2D)	🟢	🟢	🟢	🟢	🟢	🟢
Grunberger, G. (2018) (VERTIS RENAL)	🟢	🟢	🟢	🟢	🟢	🟢
GSK Clinical Trials (2010) (114130)	🟢	🟡	🟡	🟡	🔴	🔴
Hadjadj, S. (2016)	🟢	🟢	🟢	🟢	🟢	🟢
Heerspink, H.J.L. (2020) (DAPA-CKD)	🟢	🟢	🟢	🟢	🟢	🟡
Hernandez, A.F. (2018) (Harmony Outcomes)	🟢	🟢	🟢	🟢	🟢	🟢
Herrington, W.G. (2023) (EMPA-KIDNEY)	🟢	🟢	🟢	🟢	🟢	🟢
Hollander, P.L. (2011) (CV181-013)	🟢	🟢	🟡	🟢	🟢	🟡
Holman, R.R. (2017) (EXSCEL)	🟢	🟢	🟢	🟢	🟢	🟢
Home, P.D. (2017) (HARMONY 3-NCT00838903)	🟢	🟢	🟡	🟢	🟢	🟡
Home, P.D. (2017) (HARMONY 5-NCT00839527)	🟢	🟢	🟡	🟢	🟢	🟡

Husain, M. (2019) (PIONEER 6)	● green	● green	● green	● green	● green	● green
Inagaki, N. (2022) (SURPASS J-mono)	● green	● green	● green	● green	● green	● green
Januzzi, J.L. Jr. (2017) (CR017014)	● green	● green	● green	● green	● green	● green
Jastreboff, A.M. (2022) (SURMOUNT-1)	● green	● green	● green	● green	● green	● green
Kadowaki, T. (2013) (3000-A7) (12 week)	● green	● green	● green	● green	● green	● green
Kadowaki, T. (2014) (MP-513-E07)	● green	● green	● green	● green	● green	● green
Kellerer, M. (2022) (SUSTAIN 11)	● green	● yellow	● green	● green	● green	● yellow
Kosiborod, M.N. (2021) (DARE-19)	● green	● green	● green	● green	● green	● green
Kosiborod, M.N. (2023) (STEP-HFpEF)	● green	● green	● green	● green	● green	● green
Laakso, M. (2015) (2009-016971-31)	● green	● green	● green	● green	● green	● green
Lavalle-Gonzalez, F.J. (2013) (data before week 26) (CANTATA-D)	● green	● green	● green	● green	● green	● green
Ledesma, G. (2019) (1218.149)	● green	● green	● yellow	● green	● green	● yellow
Lincoff, A.M. (2023) (SELECT)	● green	● green	● green	● green	● green	● green
Lingvay, I. (2019) (SUSTAIN 8)	● green	● green	● green	● green	● green	● green
Lock, J.P. (2021) (BEST) (NCT02558296)	● green	● yellow	● red	● yellow	● yellow	● red
Ludvik, B. (2021) (SURPASS-3)	● green	● yellow	● green	● green	● green	● yellow
Margulies, K.B. (2016) (FIGHT)	● green	● green	● green	● green	● green	● green
Marso, S.P. (2016) (LEADER)	● green	● green	● green	● green	● green	● green
Marso, S.P. (2016) (SUSTAIN-6)	● green	● green	● green	● green	● green	● green
Mathieu, C. (2015) (MB102-129)	● green	● green	● green	● green	● green	● green
Mathieu, C. (2015) (MK-0431-260)	● green	● green	● green	● green	● green	● green
Matthaei, S. (2015) (CV181-168)	● green	● green	● yellow	● green	● green	● yellow
Matthews, D.R. (2019) (VERIFY)	● green	● green	● yellow	● green	● green	● yellow
McGuire, D.K. (2025) (SOUL Study)	● green	● green	● green	● green	● green	● green

McMurray, J.J.V. (2019) (DAPA-HF)	🟢	🟢	🟢	🟢	🟢	🟡
McMurray, J.J.V. (2024) (DETERMINE-preserved)	🟢	🟢	🟢	🟢	🟢	🟢
Mellander, A. (2016) (NCT00528372)	🟢	🟢	🟢	🟢	🟢	🟢
Moon, S. (2025) (NCT05616013)	🟢	🟢	🟢	🟢	🟢	🟢
Nassif, M.E. (2021) (PRESERVED-HF)	🟢	🟢	🟢	🟢	🟢	🟢
Nauck, M.A. (2007) (MK0431-024)	🟢	🟢	🟡	🟢	🟢	🟡
Nauck, M.A. (2016) (HARMONY 2)	🟢	🟢	🟢	🟢	🟢	🟢
Neal, B. (2017) (CANVAS)	🟢	🟢	🟢	🟢	🟢	🟢
Neal, B. (2017) (CANVAS-R)	🟢	🟢	🟢	🟢	🟢	🟢
O'Neil, P.M. (2018) (NCT02453711)	🟢	🟢	🟢	🟢	🟢	🟢
Packer, M. (2020) (EMPEROR-Reduced)	🟢	🟢	🟢	🟢	🟢	🟢
Packer, M. (2025) (SUMMIT)	🟢	🟢	🟢	🟢	🟢	🟢
Pan, C.Y. (2012) (D1680C00005)	🟢	🟢	🟢	🟢	🟢	🟢
Perkovic, V. (2019) (CREDENCE)	🟢	🟢	🟢	🟢	🟢	🟢
Perkovic, V. (2024) (FLOW)	🟢	🟢	🟢	🟢	🟢	🟢
Pfeffer, M.A. (2015) (ELIXA)	🟢	🟢	🟢	🟢	🟢	🟢
Pi-Sunyer, X. (2015) (SCALE) (before 56 weeks)	🟢	🟢	🟡	🟢	🟢	🟡
Pratley, R.E. (2009) (SYR-322-SULF-007)	🟢	🟢	🟢	🟢	🟢	🟢
Pratley, R.E. (2014) (HARMONY 7)	🟢	🟡	🟢	🟢	🟢	🟡
Pratley, R.E. (2018) (SUSTAIN 7)	🟢	🟢	🟢	🟢	🟢	🟢
Pratley, R.E. (2018) (VERTIS FACTORAL, MK-8835-005)	🟢	🟢	🟢	🟢	🟢	🟢
Reasner, C. (2011) (MK-0431A P079)	🟢	🟢	🟢	🟢	🟢	🟢
Ridderstrale, M. (2014) (EMPA-REG H2H-SU)	🟢	🟢	🟢	🟢	🟢	🟢
Roden, M. (2013) (EMPA-REG MONO)	🟢	🟢	🔴	🟢	🟢	🔴
Roden, M. (2015) (EMPA-REG EXTEND MONO)	🟢	🟢	🟡	🟢	🟢	🟡
Rosenstock, J. (2013) (CV181-011)	🟢	🟢	🟡	🟢	🟢	🟡

Rosenstock, J. (2014) (HARMONY 6)	🟢	🟡	🟢	🟢	🟢	🟡
Rosenstock, J. (2019) (CARMELINA)	🟢	🟢	🟢	🟢	🟢	🟢
Rosenstock, J. (2019) (CAROLINA)	🟢	🟢	🟢	🟢	🟢	🟢
Rosenstock, J. (2019) (PIONEER 3)	🟢	🟢	🟢	🟢	🟢	🟢
Rosenstock, J. (2023) (J1I-MC-GZBD)	🟢	🟢	🟢	🟢	🟢	🟢
Rubino, D.M. (2022) (STEP 8)	🟢	🟢	🟢	🟢	🟢	🟢
Schernthaner, G. (2015) (GENERATION)	🟢	🟢	🟢	🟢	🟢	🟢
Scirica, B.M. (2013) (SAVOR- TIMI 53)	🟢	🟢	🟢	🟢	🟢	🟢
Sheu, W.H. (2015) (MK-3102-006) (extension phase)	🟢	🟢	🟢	🟢	🟢	🟢
Solomon, S.D. (2022) (DELIVER)	🟢	🟢	🟢	🟢	🟢	🟡
Spertus, J.A. (2022) (CHIEF-HF)	🟢	🟢	🟡	🟢	🟢	🟡
Stenlöf, K. (2014) (CANTATA-M) (before 26 weeks)	🟢	🟢	🟢	🟢	🟢	🟢
SURMOUNT-J (2024) (NCT04844918)	🟢	🟡	🔴	🟡	🟡	🔴
Tuttle, K.R. (2022) (NCT01011868)	🟢	🟢	🟢	🟢	🟢	🟢
Vilsbøll, T. (2010) (MK0431-051)	🟢	🟢	🟢	🟢	🟢	🟢
Voors, A.A. (2022) (EMPULSE)	🟢	🟢	🟢	🟢	🟢	🟢
Wadden, T.A. (2023) (SURMOUNT-3)	🟢	🟢	🟡	🟢	🟢	🟡
Wang, W. (2024) (PIONEER 11)	🟢	🟢	🟢	🟢	🟢	🟢
Wason, S. (2021) (SOTA-INS) (NCT03285594)	🟢	🟡	🟡	🟡	🔴	🔴
Weinstock, R.S. (2015) (AWARD-5)	🟢	🟢	🟡	🟢	🟢	🟡
Weissman, P.N. (2014) (HARMONY 4)	🟢	🟢	🟢	🟢	🟢	🟢
White, W.B. (2013) (EXAMINE)	🟢	🟢	🟡	🟢	🟢	🟡
Wilding, J.P. (2012)	🟢	🟢	🟡	🟢	🟢	🟡
Wilding, J.P.H. (2021) (STEP 1)	🟢	🟢	🟢	🟢	🟢	🟢
Wiviott, S.D. (2019) (DECLARE-TIMI 58)	🟢	🟢	🟢	🟢	🟢	🟡
Wysham, C. (2014) (AWARD-1)	🟢	🟢	🟢	🟢	🟢	🟢
Yki-Järvinen, H. (2013) (2008-008296-33)	🟢	🟢	🟡	🟢	🟢	🟡
Zinman, B. (2015) (EMPA-REG OUTCOME)	🟢	🟢	🟢	🟢	🟢	🟢

Abbreviation: RoB: risk of bias

eTable 1: Keyword used in each database and search results

Database	Keyword	Filter	Date*	Result
PubMed	(COPD OR chronic obstructive pulmonary disease OR chronic obstruction pulmonary disease OR asthma OR asthma-COPD overlap syndrome OR asthma-COPD overlapping syndrome) AND (glucagon-like peptide-1 receptor agonist OR Sodium Glucose Cotransporter 2 Inhibitor OR dipeptidyl peptidase-4 inhibitor OR lixisenatide OR orforglipron OR exenatide OR semaglutide OR liraglutide OR albiglutide OR dulaglutide OR tirzepatide OR bexagliflozin OR canagliflozin OR dapagliflozin OR empagliflozin OR ertugliflozin OR ipragliflozin OR luseogliflozin OR remogliflozin OR sergliflozin OR sotagliflozin OR tofogliflozin OR henagliflozin OR janagliflozin OR mizagliflozin OR velagliflozin OR enavogliflozin OR licogliflozin OR rongliflozin OR sitagliptin OR vildagliptin OR saxagliptin OR linagliptin OR gemigliptin OR anagliptin OR teneligliptin OR alogliptin OR trelagliptin OR omarigliptin OR evogliptin OR gosogliptin OR dutogliptin OR neogliptin OR retagliptin OR denagliptin OR cofrogliptin OR fotagliptin OR prusogliptin OR cetagliptin OR retatrutide) AND (random OR randomized OR randomised)	N/A	2026/04/03	33
ClinicalKey	(COPD OR chronic obstructive pulmonary disease OR chronic obstruction pulmonary disease OR asthma OR asthma-COPD overlap syndrome OR asthma-COPD overlapping syndrome) AND (glucagon-like peptide-1 receptor agonist OR Sodium Glucose Cotransporter 2 Inhibitor OR dipeptidyl peptidase-4 inhibitor OR lixisenatide OR orforglipron OR exenatide OR semaglutide OR liraglutide OR albiglutide OR dulaglutide OR	N/A	2026/04/03	0

	tirzepatide OR bexagliflozin OR canagliflozin OR dapagliflozin OR empagliflozin OR ertugliflozin OR ipragliflozin OR luseogliflozin OR remogliflozin OR sergliflozin OR sotagliflozin OR tofogliflozin OR henagliflozin OR janagliflozin OR mizagliflozin OR velagliflozin OR enavogliflozin OR licogliflozin OR rongliflozin OR sitagliptin OR vildagliptin OR saxagliptin OR linagliptin OR gemigliptin OR anagliptin OR teneligliptin OR alogliptin OR trelagliptin OR omarigliptin OR evogliptin OR gosogliptin OR dutogliptin OR neogliptin OR retagliptin OR denagliptin OR cofrogliptin OR fotagliptin OR prusogliptin OR cetagliptin OR retatrutide) AND (random OR randomized OR randomised)			
Cochrane CENTRAL	(COPD OR chronic obstructive pulmonary disease OR chronic obstruction pulmonary disease OR asthma OR asthma-COPD overlap syndrome OR asthma-COPD overlapping syndrome) AND (glucagon-like peptide-1 receptor agonist OR Sodium Glucose Cotransporter 2 Inhibitor OR dipeptidyl peptidase-4 inhibitor OR lixisenatide OR orforglipron OR exenatide OR semaglutide OR liraglutide OR albiglutide OR dulaglutide OR tirzepatide OR bexagliflozin OR canagliflozin OR dapagliflozin OR empagliflozin OR ertugliflozin OR ipragliflozin OR luseogliflozin OR remogliflozin OR sergliflozin OR sotagliflozin OR tofogliflozin OR henagliflozin OR janagliflozin OR mizagliflozin OR velagliflozin OR enavogliflozin OR licogliflozin OR rongliflozin OR sitagliptin OR vildagliptin OR saxagliptin OR linagliptin OR gemigliptin OR anagliptin OR teneligliptin OR alogliptin OR trelagliptin OR omarigliptin OR evogliptin OR gosogliptin OR dutogliptin OR neogliptin OR retagliptin OR denagliptin OR cofrogliptin OR fotagliptin OR prusogliptin OR cetagliptin OR retatrutide) AND (random OR randomized OR randomised)	N/A	2026/04/03	23
Embase	(COPD OR chronic obstructive pulmonary disease OR chronic obstruction pulmonary	N/A	2026/04/03	270

	disease OR asthma OR asthma-COPD overlap syndrome OR asthma-COPD overlapping syndrome) AND (glucagon-like peptide-1 receptor agonist OR Sodium Glucose Cotransporter 2 Inhibitor OR dipeptidyl peptidase-4 inhibitor OR lixisenatide OR orforglipron OR exenatide OR semaglutide OR liraglutide OR albiglutide OR dulaglutide OR tirzepatide OR bexagliflozin OR canagliflozin OR dapagliflozin OR empagliflozin OR ertugliflozin OR ipragliflozin OR luseogliflozin OR remogliflozin OR sergliflozin OR sotagliflozin OR tofogliflozin OR henagliflozin OR janagliflozin OR mizagliflozin OR velagliflozin OR enavogliflozin OR licogliflozin OR rongliflozin OR sitagliptin OR vildagliptin OR saxagliptin OR linagliptin OR gemigliptin OR anagliptin OR teneligliptin OR alogliptin OR trelagliptin OR omarigliptin OR evogliptin OR gosogliptin OR dutogliptin OR neogliptin OR retagliptin OR denagliptin OR cofrogliptin OR fotagliptin OR prusogliptin OR cetagliptin OR retatrutide) AND (random OR randomized OR randomised)			
ProQuest	(COPD OR chronic obstructive pulmonary disease OR chronic obstruction pulmonary disease OR asthma OR asthma-COPD overlap syndrome OR asthma-COPD overlapping syndrome) AND (glucagon-like peptide-1 receptor agonist OR Sodium Glucose Cotransporter 2 Inhibitor OR dipeptidyl peptidase-4 inhibitor OR lixisenatide OR orforglipron OR exenatide OR semaglutide OR liraglutide OR albiglutide OR dulaglutide OR tirzepatide OR bexagliflozin OR canagliflozin OR dapagliflozin OR empagliflozin OR ertugliflozin OR ipragliflozin OR luseogliflozin OR remogliflozin OR sergliflozin OR sotagliflozin OR tofogliflozin OR henagliflozin OR janagliflozin OR mizagliflozin OR velagliflozin OR enavogliflozin OR licogliflozin OR rongliflozin OR sitagliptin OR vildagliptin OR saxagliptin OR linagliptin OR gemigliptin OR anagliptin OR teneligliptin OR alogliptin OR	N/A	2026/04/03	6436

	trelagliptin OR omarigliptin OR evogliptin OR gosogliptin OR dutogliptin OR neogliptin OR retagliptin OR denagliptin OR cofrogliptin OR fotagliptin OR prusogliptin OR cetagliptin OR retatrutide) AND (random OR randomized OR randomised)			
ScienceDirect	(COPD OR chronic obstructive pulmonary disease OR chronic obstruction pulmonary disease OR asthma) AND (glucagon-like peptide-1 receptor agonist OR Sodium Glucose Cotransporter 2 Inhibitor OR dipeptidyl peptidase-4 inhibitor)	N/A	2026/04/03	7223
Web of Science	(COPD OR chronic obstructive pulmonary disease OR chronic obstruction pulmonary disease OR asthma OR asthma-COPD overlap syndrome OR asthma-COPD overlapping syndrome) AND (glucagon-like peptide-1 receptor agonist OR Sodium Glucose Cotransporter 2 Inhibitor OR dipeptidyl peptidase-4 inhibitor OR lixisenatide OR orforglipron OR exenatide OR semaglutide OR liraglutide OR albiglutide OR dulaglutide OR tirzepatide OR bexagliflozin OR canagliflozin OR dapagliflozin OR empagliflozin OR ertugliflozin OR ipragliflozin OR luseogliflozin OR remogliflozin OR sergliflozin OR sotagliflozin OR tofogliflozin OR henagliflozin OR janagliflozin OR mizagliflozin OR velagliflozin OR enavogliflozin OR licogliflozin OR rongliflozin OR sitagliptin OR vildagliptin OR saxagliptin OR linagliptin OR gemigliptin OR anagliptin OR teneligliptin OR alogliptin OR trelagliptin OR omarigliptin OR evogliptin OR gosogliptin OR dutogliptin OR neogliptin OR retagliptin OR denagliptin OR cofrogliptin OR fotagliptin OR prusogliptin OR cetagliptin OR retatrutide) AND (random OR randomized OR randomised)	N/A	2026/04/03	21
ClinicalTrials.gov	(COPD OR chronic obstructive pulmonary disease OR chronic obstruction pulmonary disease OR asthma OR asthma-COPD overlap syndrome OR asthma-COPD overlapping syndrome) AND (glucagon-like peptide-1 receptor agonist OR Sodium Glucose	N/A	2026/04/03	1

	Cotransporter 2 Inhibitor OR dipeptidyl peptidase-4 inhibitor OR lixisenatide OR orforglipron OR exenatide OR semaglutide OR liraglutide OR albiglutide OR dulaglutide OR tirzepatide OR bexagliflozin OR canagliflozin OR dapagliflozin OR empagliflozin OR ertugliflozin OR ipragliflozin OR luseogliflozin OR remogliflozin OR sergliflozin OR sotagliflozin OR tofogliflozin OR henagliflozin OR janagliflozin OR mizagliflozin OR velagliflozin OR enavogliflozin OR licogliflozin OR rongliflozin OR sitagliptin OR vildagliptin OR saxagliptin OR linagliptin OR gemigliptin OR anagliptin OR teneligliptin OR alogliptin OR trelagliptin OR omarigliptin OR evogliptin OR gosogliptin OR dutogliptin OR neogliptin OR retagliptin OR denagliptin OR cofrogliptin OR fotagliptin OR prusogliptin OR cetagliptin OR retatrutide) AND (random OR randomized OR randomised)

*: first searched on 2024/12/12 and last updated on 2026/04/03

Abbreviation: N/A: not applied

eTable 2: Dosage stratification (stratified according to the included original RCTs)

Medication	Low-dose	Medium-dose	High-dose
Albiglutide	15 mg/week	30 mg/week	50 mg/week
Alogliptin	12.5 mg/day	NA	25 mg/day
Canagliflozin	50-100 mg/day	200 mg/day	300-600 mg/day
Dapagliflozin	2.5 mg/day	5 mg/day	10 mg/day
Dulaglutide	<1.5 mg/week	1.5 mg/week	>1.5 mg/week
Efpeglenatide	2mg/week	4 mg/week	6 mg/week
Empagliflozin	1-10 mg/day	NA	25-50 mg/day
Ertugliflozin	5 mg/day	NA	15 mg/day
Injectable semaglutide	0.05-0.5 mg/week	1.0-1.7 mg/week	2.0-2.4 mg/week
Linagliptin	2.5 mg/day	5 mg/day	10 mg/day
Liraglutide	1.2 mg/day	1.8 mg/day	3.0 mg/day
Oral semaglutide	7-10 mg/day	14 mg/day	20-25 mg/day
Saxagliptin	2.5 mg/day	5 mg/day	10 mg/day
Sitagliptin	25 mg/day	50 mg/day	100 mg/day
Teneligliptin	5 mg/day	10 mg/day	20 mg/day
Tirzepatide	1-5 mg/week	10 mg/week	15 mg/week

Abbreviation: NA: not applied; RCT: randomised controlled trial

eTable 3: Excluded studies and reason

Reason	Numbers	References
Commentary	1	[2]
Meta-analysis/Network meta-analysis	9	[3-11]
Not randomised controlled trial	7	[12-18]
Not report target outcome	238	[19-168] [169-256]
Specifically recruit patients with pre-existed chronic obstructive pulmonary disease	2	[257,258]

eTable 4: Characteristics of the included studies

Study name	Baseline illness	Comparison	Participants	Mean age (year)	Female (%)	ACOS event/participants*	Follow-up length	Ethnicity	ClinicalTrials.gov	Country
McGuire, D.K. (2025) (SOUL Study)[95]	patients with type 2 diabetes mellitus	Oral semaglutide 14mg/day<br>Placebo	4825<br>4825	66.1±7.6<br>66.1±7.5	28.5<br>29.3	35/4825<br>26/4825	104 weeks	Hispanic or Latino: 14.3%<br>Not Hispanic or Latino: 84.7%	NCT03914326	Multiple countries
Moon, S. (2025) (NCT05616013)[99]	patients with obesity	Inject semaglutide 1.0 mg/week<br>Inject semaglutide 2.4 mg/week<br>Placebo	56<br>57<br>56	50.3±11.2<br>49.6±11.8<br>47.8±14.6	57.1<br>57.9<br>57.1	2/111<br>2/55	40 weeks	Hispanic or Latino: 10.5%<br>Not Hispanic or Latino: 87.8%	NCT05616013	Multiple countries
Packer, M. (2025) (SUMMIT)[106]	patients with obesity and heart failure	Tirzepatide 15mg/week<br>Placebo	364<br>367	65.5±10.5<br>65.0±10.9	54.9<br>52.6	0/364<br>1/367	160 weeks	Hispanic or Latino: 54.7%<br>Not Hispanic or Latino: 44.2%	NCT04847557	Multiple countries
Docherty, K.F. (2024) (DETERMINE-reduced)[50]	patients with heart failure	Dapagliflozin 10mg/day<br>Placebo	156<br>157	68.4±9.8<br>67.3±11.0	28.8<br>22.3	0/156<br>1/157	16 weeks	Hispanic or Latino: 12.5%<br>Not Hispanic or Latino: 87.5%	NCT03877237	Multiple countries
McMurray, J.J.V. (2024) (DETERMINE-preserved)[96]	patients with heart failure	Dapagliflozin 10mg/day<br>Placebo	253<br>251	72.0±9.1<br>71.7±9.7	36.0<br>37.1	0/252<br>2/249	16 weeks	Hispanic or Latino: 11.7%<br>Not Hispanic or Latino: 88.3%	NCT03877224	Multiple countries
Perkovic, V. (2024) (FLOW)[109]	patients with type 2 diabetes mellitus and	Inject semaglutide 1.0 mg/week<br>Placebo	1767<br>1766	66.6±9.0<br>66.7±9.0	29.4<br>31.1	9/1767<br>10/1766	208 weeks	Hispanic or Latino: 15.7%	NCT03819153	Multiple countries

	chronic renal insufficiency							Not Hispanic or Latino: 80.2%		
SURMOUNT-J (2024) (NCT04844918)[133]	patients with obesity	Tirzepatide 10mg/week<br>Tirzepatide 15mg/week<br>Placebo	73<br>77<br>75	49.0±10.9<br>51.1±10.3<br>52.3±10.9	41.1<br>41.6<br>40.0	1/150<br>0/75	72 weeks	NA	NCT04844918	Japan
Wang, W. (2024) (PIONEER 11)[140]	patients with type 2 diabetes mellitus	Oral semaglutide 3 mg/day<br>Oral semaglutide 7 mg/day<br>Oral semaglutide 14 mg/day<br>Placebo	130<br>130<br>130<br>131	54.0±11.0<br>52.0±11.0<br>53.0±10.0<br>51.0±11.0	44.6<br>31.5<br>36.2<br>32.8	1/389<br>0/131	26 weeks	Hispanic or Latino: 0.2%<br>Not Hispanic or Latino: 99.8%	NCT04109547	Multiple countries
Aroda, V.R. (2023) (AMPLITUDE-D, NCT03684642)[34]	patients with type 2 diabetes mellitus	Efpeglenatide 4 mg/week<br>Efpeglenatide 6 mg/week<br>Dulaglutide 1.5 mg/week	303<br>302<br>303	60.3±9.6<br>60.0±10.1<br>59.4±10.1	46.9<br>52.0<br>50.5	2/605<br>0/302	56 weeks	White: 89.1%<br>Black or African American: 7.9%<br>Asian: 1.5%	NCT03684642	Multiple countries
Aroda, V.R. (2023) (AMPLITUDE-S, NCT03770728)[34]	patients with type 2 diabetes mellitus	Efpeglenatide 2 mg/week<br>Efpeglenatide 4 mg/week<br>Efpeglenatide 6 mg/week<br>Placebo	78<br>77<br>78<br>79	60.1±10.9<br>57.9±10.5<br>58.8±11.5<br>58.9±10.6	43.6<br>42.9<br>50.0<br>43.0	0/233<br>1/79	30 weeks	White: 57.4%<br>Black or African American: 10.3%<br>Asian: 30.8%	NCT03770728	Multiple countries
Aroda, V.R. (2023) (PIONEER PLUS)[32]	patients with type 2 diabetes mellitus	Oral semaglutide 14mg/day<br>Oral semaglutide 25mg/day<br>Oral semaglutide 50mg/day	536<br>535<br>535	58.4±10.4<br>58.8±10.7<br>57.6±11.2	39.4<br>43.2<br>42.6	1/1602	68 weeks	Hispanic or Latino: 6.9%<br>Not Hispanic or Latino: 93.1%	NCT04707469	Multiple countries
Cherney, D.Z.I.(2023) (SOTA-CKD3)[42]	patients with type 2 diabetes mellitus and chronic kidney disease	Sotagliflozin<br>Placebo	527<br>260	69.5±7.9<br>69.3±8.1	44.0<br>42.7	1/527<br>0/260	26 weeks	Hispanic or Latino: 25.2%	NCT03242252	Multiple countries

								Not Hispanic or Latino: 74.8%		
Herrington, W.G. (2023) (EMPA-KIDNEY)[134]	patients with renal failure	Empagliflozin 10mg/day<br>Placebo	3304<br>3305	63.9±13.9<br>63.8±13.9	33.2<br>33.1	7/3304<br>6/3305	104 weeks	Hispanic or Latino: 3.4%<br>Not Hispanic or Latino: 21.7%	NCT03594110	Multiple countries
Kosiborod, M.N. (2023) (STEP-HFpEF)[79]	patients with heart failure and obesity	Inject semaglutide 2.4 mg/week<br>Placebo	263<br>266	70.0<br>69.0	56.7<br>55.6	0/263<br>3/266	52 weeks	Hispanic or Latino: 6.8%<br>Not Hispanic or Latino: 93.2%	NCT04788511	Multiple countries
Lincoff, A.M. (2023) (SELECT)[84]	patients with obesity	Inject Semaglutide 2.4 mg/week<br>Placebo	8803<br>8801	61.6±8.9<br>61.6±8.8	27.8<br>27.5	53/8803<br>86/8801	104 weeks	Hispanic or Latino: 10.3%<br>Not Hispanic or Latino: 88.7%	NCT03574597	Multiple countries
Rosenstock, J. (2023) (J1I-MC-GZBD)[122]	patients with type 2 diabetes mellitus	Dulaglutide 1.5 mg/week<br>Retatrutide 0.4-4.0 mg/week<br>Retatrutide 8.0 mg/week<br>Retatrutide 12.0 mg/week<br>Placebo	46<br>94<br>50<br>46<br>45	54.9±10.4<br>57.4±9.4<br>55.5±8.4<br>54.4±9.7<br>57.6±10.8	71.7<br>45.7<br>62.0<br>56.5<br>51.1	0/46<br>1/190<br>0/45	36 weeks	Hispanic or Latino: 46.6%<br>Not Hispanic or Latino: 52.7%	NCT04867785	USA
Wadden, T.A. (2023) (SURMOUNT-3)[139]	patients with obesity	Tirzepatide 10-15mg/week<br>Placebo	287<br>292	45.4±12.6<br>45.7±11.8	63.1<br>62.7	0/287<br>1/292	84 weeks	Hispanic or Latino: 53.9%<br>Not Hispanic or Latino: 45.1%	NCT04657016	Multiple countries
Dahl, D. (2022) (SURPASS-5)[43]	patients with type 2 diabetes mellitus	Tirzepatide 5mg/week<br>Tirzepatide 10mg/week<br>Tirzepatide 15mg/week<br>Placebo	116<br>119<br>120<br>120	62.0±10.0<br>60.0±10.0<br>61.0±10.0<br>60.0±10.0	47.4<br>39.5<br>45.8<br>45.0	2/355<br>0/120	40 weeks	Hispanic or Latino: 46.3%<br>Not Hispanic or Latino: 80.0%	NCT04039503	Multiple countries

Frias, J.P. (2022) (AMPLITUDE-M)[52]	patients with type 2 diabetes mellitus	Efpeglenatide 2 mg/week<br>Efpeglenatide 4 mg/week<br>Efpeglenatide 6 mg/week<br>Placebo	100<br>101<br>103<br>102	58.6±10.5<br>56.3±11.5<br>59.6±10.7<br>59.5±11.7	55.0<br>51.5<br>59.2<br>50.0	4/304<br>1/102	62 weeks	White: 88.9%<br>Black or African American: 9.1%<br>Asian: 1.2%	NCT03353350	Multiple countries
Inagaki, N. (2022) (SURPASS J-mono)[73]	patients with type 2 diabetes mellitus	Tirzepatide 5mg/week<br>Tirzepatide 10mg/week<br>Tirzepatide 15mg/week<br>Dulaglutide 0.75 mg/week	159<br>158<br>160<br>159	56.8±10.1<br>56.2±10.3<br>56.0±10.7<br>57.5±10.2	28.9<br>24.7<br>17.5<br>26.4	0/477<br>1/159	52 weeks	Asian: 100.0%	NCT03861052	Japan
Jastreboff, A.M. (2022) (SURMOUNT-1)[75]	patients with obesity	Tirzepatide 5mg/week<br>Tirzepatide 10mg/week<br>Tirzepatide 15mg/week<br>Placebo	630<br>636<br>630<br>643	45.6±12.7<br>44.7±12.4<br>44.9±12.3<br>44.4±12.5	67.6<br>67.1<br>67.5<br>67.8	2/1896<br>0/643	72 weeks	White: 70.6%<br>Black or African American: 7.9%<br>Asian: 10.9%	NCT04184622	Multiple countries
Kellerer, M. (2022) (SUSTAIN 11)[78]	patients with type 2 diabetes mellitus	Inject semaglutide 1.0 mg/week<br>Control	874<br>874	60.8±9.4<br>61.5±9.5	49.1<br>48.6	0/874<br>2/864	52 weeks	Hispanic or Latino: 2.6%<br>Not Hispanic or Latino: 97.4%	NCT03689374	Multiple countries
Rubino, D.M. (2022) (STEP 8)[126]	patients with obesity	Inject semaglutide 2.4 mg/week<br>Liraglutide 3.0 mg/day<br>Placebo	126<br>127<br>85	48.0±14.0<br>49.0±13.0<br>51.0±12.0	81.0<br>76.4<br>77.6	1/126<br>0/127<br>0/85	68 weeks	Hispanic or Latino: 11.5%<br>Not Hispanic or Latino: 88.5%	NCT04074161	Multiple countries
Solomon, S.D. (2022) (DELIVER)[130]	patients with stabilized heart failure	Dapagliflozin 10mg/day<br>Placebo	3131<br>3132	71.8±9.6<br>71.5±9.5	43.6<br>44.2	34/3126<br>37/3127	120 weeks	Hispanic or Latino: 19.6%<br>Not Hispanic or Latino: 80.4%	NCT03619213	Multiple countries

Spertus, J.A. (2022) (CHIEF-HF)[131]	patients with stable symptomatic heart failure	Canagliflozin 100 mg/day<br>Placebo	222<br>226	62.9±13.2<br>64.0±13.5	46.8<br>42.9	0/224<br>2/231	12 weeks	White: 84.2%<br>Black or African American: 14.3%<br>Asian: 0.4%	NCT04252287	USA
Tuttle, K.R. (2022) (NCT01011868)[136]	patients with type 2 diabetes mellitus and chronic kidney disease	Empagliflozin 10mg/day<br>Empagliflozin 25mg/day<br>Placebo	169<br>155<br>170	58.6±9.8<br>59.9±10.5<br>58.1±9.4	45.0<br>40.0<br>47.1	0/324<br>1/170	78 weeks	NA	NCT01011868	Multiple countries
Voors, A.A. (2022) (EMPULSE)[138]	patients with acute heart failure and dyspnoea	Empagliflozin 10mg/day<br>Placebo	265<br>265	71.0<br>70.0	32.5<br>35.1	2/260<br>3/264	13 weeks	Hispanic or Latino: 2.8%<br>Not Hispanic or Latino: 97.2%	NCT04157751	Multiple countries
Anker, S.D. (2021) (EMPERIAL - Preserved) (NCT03448406)[30]	patients with heart failure	Empagliflozin 10mg/day<br>Placebo	157<br>158	73.0±9.0<br>73.9±8.6	44.6<br>41.8	0/157<br>1/158	12 weeks	Hispanic or Latino: 11.7%<br>Not Hispanic or Latino: 87.9%	NCT03448406	Multiple countries
Anker, S.D. (2021) (EMPERIAL-reduced) (NCT03448419)[30]	patients with heart failure	Empagliflozin 10mg/day<br>Placebo	156<br>156	68.7±9.9<br>69.3±10.6	22.4<br>28.8	0/155<br>1/156	12 weeks	Hispanic or Latino: 13.8%<br>Not Hispanic or Latino: 85.9%	NCT03448419	Multiple countries
Anker, S.D. (2021) (EMPEROR-Preserved)[29]	patients with heart failure with preserved ejection fraction	Empagliflozin 10mg/day<br>Placebo	2997<br>2991	71.8±9.3<br>71.9±9.6	44.6<br>44.7	27/2996<br>40/2989	156 weeks	Hispanic or Latino: 25.5%<br>Not Hispanic or Latino: 74.5%	NCT03057951	Multiple countries
Bhatt, D.L. (2021) (SCORED)[37]	patients with type 2 diabetes mellitus and chronic kidney disease	Sotagliflozin 200-400mg/day<br>Placebo	5292<br>5292	68.4±8.4<br>68.2±8.4	44.3<br>45.5	11/5291<br>23/5286	116 weeks	Hispanic or Latino: 31.6%	NCT03315143	Multiple countries

								Not Hispanic or Latino: 67.8%		
Bhatt, D.L. (2021) (SOLOIST-WHF)[38]	patients with type 2 diabetes mellitus and heart failure	Sotagliflozin 200mg/day<br>Placebo	608<br>614	68.6±9.5<br>69.3±8.8	32.6<br>34.9	5/605<br>4/611	36 weeks	Hispanic or Latino: 26.2%<br>Not Hispanic or Latino: 73.1%	NCT03521934	Multiple countries
Davies, M. (2021) (STEP 2)[44]	patients with type 2 diabetes mellitus and obesity	Inject semaglutide 1.0 mg/week<br>Inject Semaglutide 2.4 mg/week<br>Placebo	403<br>404<br>403	56.0±10.0<br>55.0±11.0<br>55.0±11.0	50.4<br>55.2<br>47.1	1/805<br>1/402	68 weeks	White: 62.1%<br>Black or African American: 8.3%<br>Asian: 26.2%	NCT03552757	Multiple countries
Del Prato, S. (2021) (SURPASS-4)[49]	patients with type 2 diabetes mellitus	Tirzepatide 5mg/week<br>Tirzepatide 10mg/week<br>Tirzepatide 15mg/week<br>Control	329<br>328<br>338<br>1000	62.9±8.6<br>63.7±8.7<br>63.7±8.6<br>63.8±8.5	39.8<br>36.3<br>39.9<br>36.4	2/995<br>4/1000	108 weeks	Hispanic or Latino: 47.5%<br>Not Hispanic or Latino: 51.7%	NCT03730662	Multiple countries
Frías, J.P. (2021) (SURPASS-2)[53]	patients with type 2 diabetes mellitus	Tirzepatide 5mg/week<br>Tirzepatide 10mg/week<br>Tirzepatide 15mg/week<br>Inject semaglutide 1.0 mg/week	470<br>469<br>470<br>469	56.3±10.0<br>57.2±10.5<br>55.9±10.4<br>56.9±10.8	56.4<br>49.3<br>54.5<br>52.0	1/1409<br>1/469	40 weeks	Hispanic or Latino: 70.1%<br>Not Hispanic or Latino: 29.9%	NCT03987919	Multiple countries
Frías, J.P. (2021) (SUSTAIN FORTE)[51]	patients with type 2 diabetes mellitus	Inject semaglutide 1.0 mg/week<br>Inject semaglutide 2.0 mg/week	481<br>480	58.2±9.9<br>57.9±10.0	41.0<br>41.9	1/959	40 weeks	Hispanic or Latino: 11.6%<br>Not Hispanic or Latino: 88.4%	NCT03989232	Multiple countries

Gerstein, H.C. (2021) (AMPLITUDE-O)[60]	patients with type 2 diabetes mellitus	Efpeglenatide 4 mg/week<br>Efpeglenatide 6 mg/week<br>Placebo	1359<br>1358<br>1359	64.6±8.2<br>64.7±8.2<br>64.4±8.3	32.5<br>35.6<br>30.8	12/2718<br>2/1355	104 weeks	White: 86.7%<br>Black or African American: 3.5%<br>Asian: 6.6%	NCT03496298	Multiple countries
Kosiborod, M.N. (2021) (DARE-19)[80]	patients with COVID-19	Dapagliflozin 10mg/day<br>Placebo	625<br>625	61.0±13.4<br>61.8±13.5	41.6<br>43.7	25/625<br>33/625	4 weeks	Hispanic or Latino: 61.0%<br>Not Hispanic or Latino: 27.7%	NCT04350593	Multiple countries
Lock, J.P. (2021) (BEST) (NCT02558296)[86]	patients with type 2 diabetes mellitus	Bexagliflozin 20mg/day<br>Placebo	1132<br>567	64.4±7.9<br>64.6±8.0	30.1<br>31.2	12/1132<br>6/567	52 weeks	Hispanic or Latino: 15.2%<br>Not Hispanic or Latino: 84.8%	NCT02558296	Multiple countries
Ludvik, B. (2021) (SURPASS-3)[87]	patients with type 2 diabetes mellitus	Tirzepatide 5mg/week<br>Tirzepatide 10mg/week<br>Tirzepatide 15mg/week<br>Control	358<br>360<br>359<br>360	57.2±10.1<br>57.4±9.7<br>57.5±10.2<br>57.5±10.1	44.1<br>45.8<br>46.0<br>40.8	1/1077<br>0/360	67 weeks	Hispanic or Latino: 29.2%<br>Not Hispanic or Latino: 70.3%	NCT03882970	Multiple countries
Nassif, M.E. (2021) (PRESERVED-HF)[100]	patients with heart failure	Dapagliflozin 10mg/day<br>Placebo	162<br>162	69.0<br>71.0	56.8<br>56.8	0/162<br>2/162	12 weeks	White: 66.4%<br>Black or African American: 29.9%<br>Asian: 0.9%	NCT03030235	USA
Wason, S. (2021) (SOTA-INS) (NCT03285594)[141]	patients with type 2 diabetes mellitus	Sotagliflozin 200-400mg/day<br>Placebo	427<br>144	62.5±9.5<br>62.2±8.9	46.2<br>40.3	1/426<br>0/144	52 weeks	Hispanic or Latino: 13.8%	NCT03285594	Multiple countries

								Not Hispanic or Latino: 84.2%		
Wilding, J.P.H. (2021) (STEP 1)[146]	patients with obesity	Inject semaglutide 2.4 mg/week<br>Placebo	1306<br>655	46.0±13.0<br>47.0±12.0	73.1<br>76.0	0/1306<br>2/655	68 weeks	Hispanic or Latino: 12.0%<br>Not Hispanic or Latino: 85.1%	NCT03548935	Multiple countries
Cannon, C.P. (2020) (VERTIS CV)[40]	patients with type 2 diabetes mellitus	Ertugliflozin 5 mg/day<br>Ertugliflozin 15 mg/day<br>Placebo	2752<br>2747<br>2747	64.3±8.2<br>64.4±8.0<br>64.4±8.0	29.1<br>30.3<br>30.7	37/5493<br>26/2745	182 weeks	White: 87.8%<br>Black or African American: 2.8%<br>Asian: 6.0%	NCT01986881	Multiple countries
Heerspink, H.J.L. (2020) (DAPA-CKD)[67]	patients with renal failure	Dapagliflozin 10mg/day<br>Placebo	2152<br>2152	61.8±12.1<br>61.9±12.1	32.9<br>33.3	9/2149<br>17/2149	125 weeks	Hispanic or Latino: 24.9%<br>Not Hispanic or Latino: 75.1%	NCT03036150	Multiple countries
Packer, M. (2020) (EMPEROR-Reduced)[105]	patients with chronic heart failure	Empagliflozin 10mg/day<br>Placebo	1863<br>1867	67.2±10.8<br>66.5±11.2	23.5<br>24.4	11/1863<br>15/1863	64 weeks	Hispanic or Latino: 32.9%<br>Not Hispanic or Latino: 62.8%	NCT03057977	Multiple countries
Gallo, S. (2019) (VERTIS MET)[54]	patients with type 2 diabetes mellitus	Ertugliflozin 5 mg/day<br>Ertugliflozin 15 mg/day<br>Placebo	207<br>205<br>209	56.6±8.2<br>56.9±9.4<br>56.5±8.7	53.1<br>54.6<br>53.1	1/412<br>0/209	104 weeks	White: 66.2%<br>Black or African American: 10.3%<br>Asian: 16.1%	NCT02033889	Multiple countries

Gerstein, H.C. (REWIND)[59]	(2019)	patients with type 2 diabetes mellitus	Dulaglutide 1.5 mg/week<br>Placebo	4949<br>4952	66.2±6.5<br>66.2±6.5	46.6<br>46.1	43/4943<br>66/4949	281 weeks	Hispanic or Latino: 1.3%<br>Not Hispanic or Latino: 8.1%	NCT01394952	Multiple countries
Husain, M. (PIONEER 6)[72]	(2019)	patients with type 2 diabetes mellitus and cardiovascular disease or chronic kidney disease	Oral semaglutide 14mg/day<br>Placebo	1591<br>1592	66.0±7.0<br>66.0±7.0	31.9<br>31.4	11/1591<br>6/1591	64 weeks	Hispanic or Latino: 16.1%<br>Not Hispanic or Latino: 83.9%	NCT02692716	Multiple countries
Ledesma, G. (1218.149)[83]	(2019)	patients with type 2 diabetes mellitus	Linagliptin 5 mg/day<br>Placebo	151<br>151	72.3±5.1<br>72.5±5.6	39.1<br>39.7	2/151<br>0/151	52 weeks	Hispanic or Latino: 11.3%<br>Not Hispanic or Latino: 88.7%	NCT02240680	Multiple countries
Lingvay, I. (SUSTAIN 8)[85]	(2019)	patients with type 2 diabetes mellitus	Inject semaglutide 1.0 mg/week<br>Canagliflozin 300 mg/day	394<br>394	55.7±11.1<br>57.5±10.7	43.4<br>49.0	1/392<br>0/394	52 weeks	Hispanic or Latino: 37.2%<br>Not Hispanic or Latino: 62.8%	NCT03136484	Multiple countries
Matthews, D.R. (VERIFY)[94]	(2019)	patients with type 2 diabetes mellitus	Vildagliptin 100mg/day<br>Placebo	998<br>1003	54.1±9.5<br>54.6±9.2	54.6<br>51.3	3/998<br>3/1001	260 weeks	White: 60.8%<br>Black or African American: 2.4%<br>Asian: 18.6%	NCT01528254	Multiple countries
McMurray, J.J.V. (DAPA-HF)[97]	(2019)	patients with stabilized heart failure	Dapagliflozin 10mg/day<br>Placebo	2373<br>2371	66.2±11.0<br>66.5±10.8	23.8<br>23.0	18/2368<br>36/2368	73 weeks	Hispanic or Latino: 16.2%<br>Not Hispanic or Latino: 83.8%	NCT03036124	Multiple countries

Perkovic, V. (2019) (CREDENCE)[108]	patients with type 2 diabetes mellitus and nephropathy	Canagliflozin 100 mg/day<br>Placebo	2202<br>2199	62.9±9.2<br>63.2±9.2	34.6<br>33.3	11/2200<br>17/2197	130 weeks	Hispanic or Latino: 32.3%<br>Not Hispanic or Latino: 65.7%	NCT02065791	Multiple countries
Rosenstock, J. (2019) (CARMELINA)[125]	patients with type 2 diabetes mellitus	Linagliptin 5 mg/day<br>Placebo	3494<br>3485	66.1±9.1<br>65.6±9.1	38.5<br>35.7	24/3494<br>31/3485	182 weeks	Hispanic or Latino: 35.8%<br>Not Hispanic or Latino: 64.2%	NCT01897532	Multiple countries
Rosenstock, J. (2019) (CAROLINA)[124]	patients with type 2 diabetes mellitus	Linagliptin 5 mg/day<br>Placebo	3023<br>3010	63.9±9.5<br>64.2±9.5	39.2<br>40.8	29/3023<br>33/3010	338 weeks	Hispanic or Latino: 17.1%<br>Not Hispanic or Latino: 82.6%	NCT01243424	Multiple countries
Rosenstock, J. (2019) (PIONEER 3)[120]	patients with type 2 diabetes mellitus	Oral semaglutide 3 mg/day<br>Oral semaglutide 7 mg/day<br>Oral semaglutide 14 mg/day<br>Sitagliptin 100 mg/day	466<br>465<br>465<br>467	58.0±10.0<br>58.0±10.0<br>57.0±10.0<br>58.0±10.0	45.5<br>47.3<br>46.9<br>49.0	1/1396<br>1/467	78 weeks	White: 71.1%<br>Black or African American: 8.6%<br>Asian: 13.2%	NCT02607865	Multiple countries
Wiviott, S.D. (2019) (DECLARE-TIMI 58)[147]	patients with type 2 diabetes mellitus and atherosclerotic vascular disease	Dapagliflozin 10mg/day<br>Placebo	8582<br>8578	63.9±6.8<br>64.0±6.8	36.9<br>37.9	93/8574<br>112/8569	206 weeks	Hispanic or Latino: 15.0%<br>Not Hispanic or Latino: 85.0%	NCT01730534	Multiple countries
Ahmann, A. J. (2018) (SUSTAIN 3)[27]	patients with type 2 diabetes mellitus	Inject semaglutide 1.0 mg/week<br>Exenatide	404<br>405	56.4<br>56.7	45.8<br>43.7	0/404<br>1/405	56 weeks	NA	NCT01885208	Multiple countries

Grunberger, G. (2018) (VERTIS RENAL)[65]	patients with type 2 diabetes mellitus with chronic kidney disease	Ertugliflozin 5 mg/day<br>Ertugliflozin 15 mg/day<br>Placebo	158<br>155<br>154	66.7±8.3<br>67.5±8.5<br>67.5±8.9	46.8<br>51.6<br>53.2	1/313<br>1/154	54 weeks	NA	NCT01986855	Multiple countries
Hernandez, A.F. (2018) (Harmony Outcomes)[68]	patients with type 2 diabetes mellitus	Albiglutide 50 mg/week<br>Placebo	4731<br>4732	64.1±8.7<br>64.2±8.7	30.2<br>31.0	34/4717<br>42/4715	86 weeks	White: 84.9%<br>Black or African American: 2.5%<br>Asian: 4.4%	NCT02465515	Multiple countries
O'Neil, P.M. (2018) (NCT02453711)[104]	patients with obesity	Inject semaglutide 0.05-0.4 mg/week<br>Liraglutide 3.0mg/day<br>Placebo	718<br>103<br>136	46.4±12.6<br>48.5±11.2<br>46.4±12.8	64.6<br>65.0<br>64.7	1/718<br>0/103<br>0/136	52 weeks	White: 73.1%<br>Black or African American: 6.4%<br>Asian: 0.9%	NCT02453711	Multiple countries
Pratley, R.E. (2018) (SUSTAIN 7)[112]	patients with type 2 diabetes mellitus	Inject semaglutide 0.5 mg/week<br>Inject semaglutide 1.0 mg/week<br>Dulaglutide 0.75 mg/week<br>Dulaglutide 1.5 mg/week	301<br>300<br>299<br>299	56.0±10.9<br>55.0±10.6<br>55.0±10.4<br>56.0±10.6	43.9<br>46.0<br>53.5<br>57.2	1/601<br>0/598	40 weeks	Hispanic or Latino: 11.5%<br>Not Hispanic or Latino: 88.5%	NCT02648204	Multiple countries
Pratley, R.E. (2018) (VERTIS FACTORAL, MK-8835-005)[113]	patients with type 2 diabetes mellitus	Ertugliflozin 5 mg/day<br>Ertugliflozin 15 mg/day<br>Sitagliptin 100 mg/day<br>Ertugliflozin 5 mg/day + Sitagliptin 100 mg/day<br>Ertugliflozin 15 mg/day + Sitagliptin 100 mg/day	250<br>248<br>247<br>243<br>244	55.1±10.1<br>55.3±9.5<br>54.8±10.7<br>55.2±10.4<br>55.1±9.8	49.2<br>46.0<br>37.7<br>49.4<br>48.4	1/498<br>0/247<br>0/487	52 weeks	NA	NCT02099110	Multiple countries
Ahren, B. (2017) (SUSTAIN 2)[28]	patients with type 2 diabetes mellitus	Inject semaglutide 1.0 mg/week<br>Inject semaglutide 0.5 mg/week<br>Sitagliptin 100 mg/day	409<br>409<br>407	56.0±9.4<br>54.8±10.2<br>54.6±10.4	49.9<br>50.6<br>51.1	1/818<br>0/407	56 weeks	NA	NCT01930188	Multiple countries

Aroda, V.R. (2017) (SUSTAIN 4)[33]	patients with type 2 diabetes mellitus	Inject semaglutide 0.5 mg/week<br>Inject semaglutide 1.0 mg/week<br>Control	362<br>360<br>360	56.5±10.3<br>56.7±10.4<br>56.2±10.6	45.6<br>49.4<br>45.8	0/722<br>1/360	30 weeks	NA	NCT02128932	Multiple countries
Ba, J. (2017) (MK-0431-253)[35]	patients with type 2 diabetes mellitus	Sitagliptin 100 mg/day<br>Placebo	249<br>249	57.5±9.5<br>56.5±9.3	53.0<br>47.0	1/248<br>0/249	24 weeks	NA	NCT01590771	China
Gantz, I. (2017) (MK-3102-018)[58]	patients with type 2 diabetes mellitus and cardiovascular disease	Omarigliptin 25 mg/week<br>Placebo	2100<br>2102	63.7±8.5<br>63.6±8.5	30.4<br>29.3	11/2092<br>7/2100	156 weeks	NA	NCT01703208	Multiple countries
Groop, P.H. (2017) (MARLINA-T2D)[64]	patients with type 2 diabetes mellitus	Linagliptin 5 mg/day<br>Placebo	182<br>178	61.0±10.0<br>60.1±9.3	36.3<br>36.5	1/182<br>0/178	24 weeks	NA	NCT01792518	Multiple countries
Holman, R.R. (2017) (EXSCEL)[70]	patients with type 2 diabetes mellitus	Exenatide 2mg/day<br>Placebo	7356<br>7396	61.8±9.4<br>61.9±9.4	38.0<br>38.0	91/7344<br>88/7372	166 weeks	White: 75.8%<br>Black or African American: 6.0%<br>Asian: 9.8%	NCT01144338	Multiple countries
Home, P.D. (2017) (HARMONY 3-NCT00838903)[71]	patients with type 2 diabetes mellitus	Albiglutide 30 mg/week<br>Placebo<br>Sitagliptin 100 mg/day	302<br>101<br>302	54.3±10.1<br>56.1±10.0	55.3<br>50.5	1/302<br>0/101<br>0/302	104 weeks	White: 71.4%<br>Black or African American: 14.8%<br>Asian: 3.4%	NCT00838903	Multiple countries
Home, P.D. (2017) (HARMONY 5-NCT00839527)[71]	patients with type 2 diabetes mellitus	Albiglutide 50 mg/week<br>Placebo	271<br>115	54.5±9.5<br>55.7±9.6	50.2<br>39.1	7/271<br>0/115	156 weeks	White: 68.8%<br>Black or African American: 10.3%<br>Asian: 6.8%	NCT00839527	Multiple countries
Januzzi, J.L. Jr. (2017) (CR017014)[74]	patients with type 2 diabetes mellitus	Canagliflozin 100 mg/day<br>Canagliflozin 300 mg/day<br>Placebo	241<br>236<br>237	64.3±6.5<br>63.4±6.0<br>63.2±6.2	48.5<br>45.3<br>39.7	2/477<br>1/237	104 weeks	NA	NCT01106651	Multiple countries

Neal, B. (2017) (CANVAS)[103]	patients with type 2 diabetes mellitus	Canagliflozin 100 mg/day<br>Canagliflozin 300 mg/day<br>Placebo	1445<br>1443<br>1442	62.2±8.0<br>62.8±8.1<br>62.3±7.9	33.5<br>34.6<br>33.7	19/2886<br>10/1441	126 weeks	Hispanic or Latino: 9.6%<br>Not Hispanic or Latino: 90.2%	NCT01032629	Multiple countries
Neal, B. (2017) (CANVAS-R)[103]	patients with type 2 diabetes mellitus	Canagliflozin 300 mg/day<br>Placebo	2907<br>2905	63.9±8.4<br>64.0±8.3	36.2<br>38.2	6/2904<br>16/2903	126 weeks	Hispanic or Latino: 20.5%<br>Not Hispanic or Latino: 79.0%	NCT01989754	Multiple countries
Davies, M.J. (2016) (LIRA-RENAL)[45]	patients with type 2 diabetes mellitus	Liraglutide 1.8 mg/day<br>Placebo	140<br>137	68.0±8.3<br>66.3±8.0	46.4<br>52.6	1/140<br>0/137	26 weeks	NA	NCT01620489	Multiple countries
Del Prato, S. (2016) (ENDURE)[48]	patients with type 2 diabetes mellitus	Alogliptin 12.5 mg/day<br>Alogliptin 25 mg/day<br>Placebo	880<br>885<br>874	55.2±9.6<br>55.5±9.8<br>55.4±9.6	52.4<br>48.9<br>49.5	3/1751<br>1/869	104 weeks	White: 62.3%<br>Black or African American: 8.4%<br>Asian:22.8%	NCT00856284	Multiple countries
Hadjadj, S. (2016)[66]	patients with type 2 diabetes mellitus	Empagliflozin 10mg/day<br>Empagliflozin 25mg/day<br>Placebo	497<br>551<br>332	52.5±11.2<br>52.4±10.7<br>52.5±10.9	41.1<br>42.5<br>46.4	1/1048<br>0/332	24 weeks	NA	NCT01719003	Multiple countries
Margulies, K.B. (2016) (FIGHT)[88]	patients with heart failure	Liraglutide 1.8 mg/day<br>Placebo	154<br>146	62.0<br>61.0	20.1<br>22.6	2/154<br>0/146	26 weeks	Hispanic or Latino: 5.0%<br>Not Hispanic or Latino: 95.0%	NCT01800968	Multiple countries
Marso, S.P. (2016) (LEADER)[90]	patients with type 2 diabetes mellitus	Liraglutide 1.8mg/day<br>Placebo	4668<br>4672	64.2±7.2<br>64.4±7.2	35.5<br>36.0	97/4668<br>107/4672	198 weeks	NA	NCT01179048	Multiple countries
Marso, S.P. (2016) (SUSTAIN-6)[89]	patients with type 2 diabetes mellitus	Inject semaglutide 0.5 mg/week<br>Inject semaglutide 1.0 mg/week<br>Placebo	826<br>822<br>1649	NA	40.1<br>37.0<br>40.0	12/1648<br>17/1649	109 weeks	NA	NCT01720446	Multiple countries

Mellander, A. (2016) (NCT00528372)[98]	patients with type 2 diabetes mellitus	Dapagliflozin 2.5 mg/day<br>Dapagliflozin 5 mg/day<br>Dapagliflozin 10 mg/day<br>Placebo	132<br>132<br>146<br>75	NA	50.7<br>54.6<br>50.0<br>58.7	1/410<br>0/75	102 weeks	White: 94.8%<br>Black or African American: 1.8%<br>Asian: 2.2%	NCT00528372	Multiple countries
Nauck, M.A. (2016) (HARMONY 2)[102]	patients with type 2 diabetes mellitus	Albiglutide 30 mg/week<br>Albiglutide 50 mg/week<br>Placebo	101<br>99<br>101	53.6±10.9<br>52.0±11.8<br>53.1±11.7	42.6<br>49.5<br>42.6	3/200<br>2/101	52 weeks	White: 79.7%<br>Black or African American: 12.6%<br>Asian: 7.6%	NCT00849017	Multiple countries
Davies, M.J. (2015) (SCALE)[46]	patients with type 2 diabetes mellitus and obesity	Liraglutide 3.0 mg/day<br>Liraglutide 1.8 mg/day<br>Placebo	423<br>211<br>212	55.0±10.8<br>54.9±10.7<br>54.7±9.8	48.0<br>48.8<br>54.2	1/634<br>2/212	56 weeks	Hispanic or Latino: 10.3%<br>Not Hispanic or Latino: 89.4%	NCT01272232	Multiple countries
Giorgino, F. (2015) (AWARD-2)[61]	patients with type 2 diabetes mellitus	Dulaglutide 0.75 mg/week<br>Dulaglutide 1.5 mg/week<br>Control	272<br>273<br>262	56.6±9.3<br>56.2±9.8<br>57.0±9.0	50.0<br>47.3<br>48.9	0/545<br>1/262	78 weeks	Hispanic or Latino: 36.1%<br>Not Hispanic or Latino: 63.9%	NCT01075282	Multiple countries
Green, J.B. (2015) (TECOS - MK-0431-082)[63]	patients with type 2 diabetes mellitus	Sitagliptin 100 mg/day<br>Placebo	7332<br>7339	65.4±7.9<br>65.5±8.0	29.1<br>29.5	28/7266<br>41/7274	192 weeks	NA	NCT00790205	Multiple countries
Laakso, M. (2015) (2009-016971-31)[81]	patients with type 2 diabetes mellitus	Linagliptin 5 mg/day<br>Placebo	113<br>122	67.3±9.2<br>65.9±9.4	38.1<br>35.2	0/113<br>1/122	52 weeks	NA	NCT01087502	Multiple countries
Mathieu, C. (2015) (MB102-129)[91]	patients with type 2 diabetes mellitus	Dapagliflozin 10mg/day + saxagliptin 5mg/day<br>Saxagliptin 5mg/day + Placebo	160<br>160	55.0±9.6<br>55.2±8.6	52.5<br>56.3	1/160<br>0/160	52 weeks	White: 92.8%<br>Black or African American: 5.6%	NCT01646320	Multiple countries

								Asian: 0.6%		
Mathieu, C. (2015) (MK-0431-260)[92]	patients with type 2 diabetes mellitus	Sitagliptin 100 mg/day<br>Placebo	329<br>329	59.3±8.9<br>58.3±9.7	54.1<br>50.2	1/329<br>0/329	24 weeks	NA	NCT01462266	Multiple countries
Matthaei, S. (2015) (CV181-168)[93]	patients with type 2 diabetes mellitus	Dapagliflozin 10 mg/day + saxagliptin 5 mg/day<br>Dapagliflozin 10 mg/day + Placebo	153<br>162	54.7±9.8<br>54.5±9.3	52.3<br>53.1	0/153<br>1/162	52 weeks	White: 87.9%<br>Black or African American: 6.3%<br>Asian: 4.1%	NCT01619059	Multiple countries
Pfeffer, M.A. (2015) (ELIXA)[110]	patients with type 2 diabetes mellitus and recent acute coronary syndrome	Lixisenatide 20ug/day<br>Placebo	3034<br>3034	59.9±9.7<br>60.6±9.6	30.4<br>30.9	20/3031<br>19/3032	100 weeks	White: 75.4%<br>Black or African American: 3.6%<br>Asian: 12.7%	NCT01147250	Multiple countries
Pi-Sunyer, X. (2015) (SCALE) (before 56 weeks)[111]	patients with obesity	Liraglutide 3.0mg/day<br>Placebo	2487<br>1244	45.2±12.1<br>45.0±12.0	78.7<br>78.1	3/2481<br>0/1242	56 weeks	NA	NCT01272219	Multiple countries
Roden, M. (2015) (EMPA-REG EXTEND MONO)[118]	patients with type 2 diabetes mellitus	Empagliflozin 10mg/day<br>Empagliflozin 25mg/day<br>Sitagliptin 100 mg/day<br>Placebo	224<br>224<br>223<br>228	56.2±11.6<br>53.8±11.6<br>55.1±9.9<br>54.9±10.9	36.6<br>35.3<br>36.8<br>46.1	1/453<br>1/223<br>0/223	76 weeks	NA	NCT01289990	Multiple countries
Schernthaner, G. (2015) (GENERATION)[127]	patients with type 2 diabetes mellitus	Saxagliptin 5mg/day<br>Placebo	360<br>360	72.5±5.7<br>72.7±5.4	39.7<br>36.7	1/359<br>1/359	52 weeks	NA	NCT01006603	Multiple countries
Sheu, W.H. (2015) (MK-3102-006) (extension phase)[129]	patients with type 2 diabetes mellitus	Omarigliptin 25 mg/week<br>Placebo	571<br>114	55.0±9.0<br>55.9±8.4	43.6<br>43.0	1/571<br>0/114	66 weeks	NA	NCT01217073	Multiple countries

Weinstock, R.S. (2015) (AWARD-5)[142]	patients with type 2 diabetes mellitus	Dulaglutide 0.25-1.0 mg/week<br>Dulaglutide 1.5 mg/week<br>Dulaglutide 2.0-3.0 mg/week<br>Placebo<br>Sitagliptin 100 mg/day	361<br>304<br>45<br>177<br>315	54.5±9.6<br>53.7±10.0<br>52.6±10.9<br>54.9±9.1	55.9<br>52.0<br>71.1<br>49.2	11/710<br>0/177<br>2/315	104 weeks	White: 51.0%<br>Black or African American: 4.2%<br>Asian: 15.6%	NCT00734474	Multiple countries
Zinman, B. (2015) (EMPA-REG OUTCOME)[150]	patients with type 2 diabetes mellitus	Empagliflozin 10mg/day<br>Empagliflozin 25mg/day<br>Placebo	2345<br>2342<br>2333	63.0±8.6<br>63.2±8.6<br>63.2±8.8	29.5<br>28.1<br>28.0	23/4687<br>17/2333	135 weeks	NA	NCT01131676	Multiple countries
Kadowaki, T. (2014) (MP-513-E07)[77]	patients with type 2 diabetes mellitus	Teneligliptin 5 mg/day<br>Teneligliptin 10 mg/day<br>Teneligliptin 20 mg/day<br>Teneligliptin 40 mg/day<br>Placebo	87<br>93<br>91<br>88<br>88	58.8±7.7<br>58.5±8.4<br>58.3±9.5<br>58.2±8.6<br>58.9±8.2	47.1<br>45.2<br>38.5<br>40.9<br>46.6	1/359<br>1/88	52 weeks	NA	NCT00971243	Japan
Pratley, R.E. (2014) (HARMONY 7)[115]	patients with type 2 diabetes mellitus	Albiglutide 50 mg/week<br>Liraglutide 1.8mg/day	404<br>408	55.4±10.0<br>55.8±10.0	52.7<br>46.6	0/404<br>1/408	32 weeks	White: 69.1%<br>Black or African American: 9.4%<br>Asian: 5.4%	NCT01128894	Multiple countries
Ridderstrale, M. (2014) (EMPA-REG H2H-SU)[117]	patients with type 2 diabetes mellitus and moderate-to-severe chronic kidney disease	Empagliflozin 25mg/day<br>Control	765<br>780	56.2±10.3<br>55.7±10.4	43.5<br>46.0	2/765<br>1/780	104 weeks	NA	NCT01167881	Multiple countries
Rosenstock, J. (2014) (HARMONY 6)[121]	patients with type 2 diabetes mellitus	Albiglutide 50 mg/week<br>Control	285<br>281	54.8±9.1<br>56.3±8.9	53.7<br>51.6	2/285<br>0/281	52 weeks	White: 61.0%<br>Black or African American: 12.9%<br>Asian: 8.7%	NCT00976391	Multiple countries

Stenlöf, K. (2014) (CANTATA-M)[132]	patients with type 2 diabetes mellitus and moderate-to-severe chronic kidney disease	Canagliflozin 100 mg/day<br>Canagliflozin 300 mg/day<br>Placebo	195<br>197<br>192	55.1±10.8<br>55.3±10.2<br>55.7±10.9	58.5<br>54.8<br>54.2	1/392<br>0/192	52 weeks	NA	NCT01081834	Multiple countries
Weissman, P.N. (2014) (HARMONY 4)[143]	patients with type 2 diabetes mellitus	Albiglutide 30 mg/week<br>Control	504<br>241	55.8±9.3<br>54.7±9.8	43.3<br>45.2	15/504<br>5/241	52 weeks	White: 67.1%<br>Black or African American: 26.0%<br>Asian: 3.2%	NCT00838916	Multiple countries
Wysham, C. (2014) (AWARD-1)[148]	patients with type 2 diabetes mellitus	Dulaglutide 0.75 mg/week<br>Dulaglutide 1.5 mg/week<br>Exenatide<br>Placebo	280<br>279<br>276<br>141	55.8±9.5<br>56.3±9.7<br>55.0±10.0<br>55.0±10.0	40.0<br>41.6<br>43.5<br>41.1	1/559<br>0/276<br>0/141	26 weeks	Hispanic or Latino: 33.9%<br>Not Hispanic or Latino: 66.0%	NCT01064687	Multiple countries
Arjona Ferreira, J.C. (2013) (MK-0431-063 AM1)[31]	patients with type 2 diabetes mellitus and chronic renal insufficiency	Sitagliptin 50 mg/day<br>Placebo	135<br>142	64.8±10.6<br>64.3±9.2	40.7<br>45.1	1/210<br>1/212	54 weeks	NA	NCT00509262	Multiple countries
Charbonnel, B. (2013) (MK-0431-403)[41]	patients with type 2 diabetes mellitus	Liraglutide 1.2 mg/day<br>Sitagliptin 100 mg/day	327<br>326	57.6±10.8<br>56.9±10.0	45.0<br>45.4	1/324<br>1/326	26 weeks	NA	NCT01296412	Multiple countries
Göke, B. (2013) (D1680C00001)[62]	patients with type 2 diabetes mellitus	Saxagliptin 5mg/day<br>Placebo	428<br>430	57.5<br>57.6	50.5<br>46.0	1/428<br>0/430	104 weeks	NA	NCT00575588	Multiple countries
Kadowaki, T. (2013) (3000-A7) (12 week)[76]	patients with type 2 diabetes mellitus	Teneligliptin 20 mg/day<br>Placebo	103<br>101	59.7±9.7<br>61.1±8.9	34.0<br>24.8	1/103<br>1/101	12 weeks	NA	NCT01026194	Japan
Lavalle-Gonzalez, F.J. (2013) (data before week 26) (CANTATA-D)[82]	patients with type 2 diabetes mellitus	Canagliflozin 100 mg/day<br>Canagliflozin 300 mg/day<br>Placebo<br>Sitagliptin 100 mg/day	368<br>367<br>183<br>366	55.5±9.4<br>55.3±9.2<br>55.3±9.8	52.7<br>55.0<br>48.6	0/735<br>0/183<br>1/366	26 weeks	NA	NCT01106677	Multiple countries

Roden, M. (2013) (EMPA-REG MONO)[119]	patients with type 2 diabetes mellitus	Empagliflozin 10mg/day<br>Empagliflozin 25mg/day<br>Placebo<br>Sitagliptin 100 mg/day	224<br>224<br>228<br>223	56.2±11.6<br>53.8±11.6<br>54.9±10.9	36.6<br>35.3<br>46.1	0/448<br>0/228<br>1/223	24 weeks	NA	NCT01177813	Multiple countries
Rosenstock, J. (2013) (CV181-011)[123]	patients with type 2 diabetes mellitus	Saxagliptin 2.5mg/day<br>Saxagliptin 5mg/day<br>Saxagliptin 10mg/day<br>Placebo	102<br>106<br>98<br>95	53.3±10.1<br>53.9±11.6<br>52.7±11.3<br>53.9±12.3	43.1<br>49.1<br>54.1<br>50.5	1/306<br>0/95	168 weeks	White: 85.0%<br>Black or African American: 5.5%<br>Asian: 4.5%	NCT00121641	Multiple countries
Scirica, B.M. (2013) (SAVOR- TIMI 53)[128]	patients with type 2 diabetes mellitus	Saxagliptin 5mg/day<br>Placebo	8280<br>8212	65.1±8.5<br>65.0±8.6	33.4<br>32.7	108/8280<br>87/8212	128 weeks	Hispanic or Latino: 21.5%<br>Not Hispanic or Latino: 78.5%	NCT01107886	Multiple countries
White, W.B. (2013) (EXAMINE)[144]	patients with type 2 diabetes mellitus	Alogliptin 25 mg/day<br>Placebo	2701<br>2679	61.0<br>61.0	32.3<br>32.0	14/2701<br>21/2679	52 weeks	Hispanic or Latino: 28.6%<br>Not Hispanic or Latino: 71.4%	NCT00968708	Multiple countries
Yki-Järvinen, H. (2013) (2008-008296-33)[149]	patients with type 2 diabetes mellitus	Linagliptin 5 mg/day<br>Placebo	631<br>630	59.7±9.9<br>60.4±10.0	47.9<br>47.8	2/631<br>2/630	52 weeks	NA	NCT00954447	Multiple countries
Gallwitz, B. (2012) (2007-004585-40)[57]	patients with type 2 diabetes mellitus	Linagliptin 5 mg/day<br>Placebo	776<br>775	59.8±9.4<br>59.8±9.4	40.5<br>39.2	4/776<br>0/775	104 weeks	NA	NCT00622284	Multiple countries
Gallwitz, B. (2012) (EUREXA)[56]	patients with type 2 diabetes mellitus	Exenatide<br>Control	490<br>487	56.0±10.0<br>56.0±9.1	44.5<br>48.3	1/511<br>0/508	104 weeks	NA	NCT00359762	Multiple countries
Pan, C.Y. (2012) (D1680C00005)[107]	patients with type 2 diabetes mellitus	Saxagliptin 5mg/day<br>Placebo	284<br>284	51.2±10.0<br>51.6±10.3	43.7<br>45.4	0/284<br>1/284	24 weeks	NA	NCT00698932	Multiple countries

Wilding, J.P. (2012)[145]	patients with type 2 diabetes mellitus and moderate-to-severe chronic kidney disease	Dapagliflozin 2.5 mg/day<br>Dapagliflozin 5 mg/day<br>Dapagliflozin 10 mg/day<br>Placebo	202<br>211<br>194<br>193	59.8±7.6<br>59.3±7.9<br>59.3±8.8<br>58.8±8.6	50.5<br>52.6<br>55.2<br>50.8	1/610<br>0/197	104 weeks	White: 95.0%<br>Black or African American: 2.4%<br>Asian: 1.6%	NCT00673231	Multiple countries
Barzilai, N. (2011) (MK0431-047)[36]	patients with type 2 diabetes mellitus	Sitagliptin 100 mg/day<br>Placebo	102<br>104	71.6±6.1<br>72.1±6.0	52.9<br>52.9	0/102<br>1/104	24 weeks	White: 78.6%<br>Black or African American: 9.2%<br>Asian: 2.9%	NCT00305604	Multiple countries
Gallwitz, B. (2011) (H8O-SB-GWBN)[55]	patients with type 2 diabetes mellitus	Exenatide 10-20mcg/day<br>Control	181<br>173	57.2±10.0<br>56.9±9.9	40.3<br>44.5	0/247<br>2/233	26 weeks	NA	NCT00434954	Germany
Hollander, P.L. (2011) (CV181-013)[69]	patients with type 2 diabetes mellitus	Saxagliptin 2.5mg/day<br>Saxagliptin 5mg/day<br>Placebo	195<br>186<br>184	54.9±9.7<br>53.2±10.6<br>54.0±10.1	45.6<br>52.2<br>53.8	2/381<br>0/184	76 weeks	NA	NCT00295633	Multiple countries
Reasner, C. (2011) (MK-0431A P079)[116]	patients with type 2 diabetes mellitus	Sitagliptin 100 mg/day<br>Placebo	625<br>621	49.4±10.5<br>50.0±10.5	43.5<br>42.7	1/625<br>0/621	44 weeks	White: 80.0%<br>Black or African American: 13.6%<br>Asian: 3.3%	NCT00482729	Multiple countries
GSK Clinical Trials (2010) (114130)[135]	patients with type 2 diabetes mellitus and chronic kidney disease	Albiglutide 30 mg/week<br>Sitagliptin 100 mg/day	249<br>246	63.2±8.4<br>63.5±9.0	45.4<br>47.2	1/249<br>0/246	52 weeks	White: 45.1%<br>Black or African American: 15.8%<br>Asian: 15.8%	NCT01098539	Multiple countries
Vilsbøll, T. (2010) (MK0431-051)[137]	patients with type 2 diabetes mellitus	Sitagliptin 100 mg/day<br>Placebo	322<br>319	58.3±9.1<br>57.2±9.3	51.2<br>47.0	0/322<br>1/319	24 weeks	White: 69.7%	NCT00395343	Multiple countries

								Black or African American: 6.9%<br>Asian: 18.1%		
Buse, J.B. (2009) (LEAD-6)[39]	patients with type 2 diabetes mellitus and moderate-to-severe chronic kidney disease	Liraglutide 1.8 mg/day<br>Exenatide	233<br>231	56.3±9.8<br>57.1±10.8	51.1<br>45.0	0/235<br>3/232	26 weeks	Hispanic or Latino: 12.3%<br>Not Hispanic or Latino: 87.7%	NCT00518882	Multiple countries
DeFronzo, R.A. (2009) (CV181-014)[47]	patients with type 2 diabetes mellitus	Saxagliptin 2.5mg/day<br>Saxagliptin 5mg/day<br>Saxagliptin 10mg/day<br>Placebo	192<br>191<br>181<br>179	54.7±10.1<br>54.7±9.6<br>54.2±10.1<br>54.8±10.2	56.8<br>46.1<br>47.5<br>46.4	1/564<br>0/179	24 weeks	Hispanic or Latino: 14.5%<br>Not Hispanic or Latino: 30.8%	NCT00121667	Multiple countries
Pratley, R.E. (2009) (SYR-322-SULF-007)[114]	patients with type 2 diabetes mellitus	Alogliptin 12.5 mg/day<br>Alogliptin 25 mg/day<br>Placebo	203<br>198<br>99	56.5±11.1<br>56.5±11.7<br>57.1±10.0	45.3<br>50.0<br>48.5	1/401<br>0/99	26 weeks	NA	NCT00286468	Multiple countries
Nauck, M.A. (2007) (MK0431-024)[101]	patients with type 2 diabetes mellitus	Sitagliptin 100 mg/day<br>Placebo	588<br>584	56.8±9.3<br>56.6±9.8	42.9<br>38.7	1/588<br>0/584	52 weeks	White: 73.9%<br>Black or African American: 6.5%<br>Asian: 8.4%	NCT00094770	Multiple countries

*: pool the same regimens with different dosage into one group

Abbreviations: DPP4 inhibitor: dipeptidyl peptidase 4 inhibitor; GLP-1 agonist: glucagon-like peptide-1 agonist; NA: not available; SGLT2 inhibitor: sodium–glucose cotransporter 2 inhibitor

eTable 5A League table of primary outcome: overall asthma-COPD overlap syndrome risk

Tirzepatide	0.52 (0.02,13.42)	1.20 (0.46,3.11)	1.17 (0.42,3.23)	1.18 (0.39,3.55)	1.31 (0.50,3.45)	1.34 (0.51,3.55)	1.33 (0.43,4.05)	1.36 (0.48,3.83)	1.42 (0.56,3.63)	1.16 (0.08,16.96)	1.45 (0.54,3.93)	1.33 (0.14,13.05)	1.53 (0.59,4.00)	1.85 (0.16,21.30)	1.66 (0.62,4.43)	1.88 (0.30,11.88)	1.88 (0.49,7.17)	1.78 (0.66,4.82)	1.97 (0.65,6.00)	1.87 (0.75,4.70)	1.96 (0.75,5.13)	2.28 (0.87,5.94)	2.74 (0.75,9.95)	2.98 (0.72,12.27)	2.56 (0.92,7.09)
1.93 (0.07,49.75)	Ertugliflozin_ Sitagliptin	2.31 (0.10,53.00)	2.25 (0.10,52.45)	2.27 (0.09,54.51)	2.52 (0.11,57.85)	2.58 (0.11,59.50)	2.55 (0.11,61.61)	2.61 (0.12,57.97)	2.73 (0.12,62.25)	2.23 (0.04,123.17)	2.80 (0.12,63.99)	2.57 (0.06,109.50)	2.95 (0.13,67.60)	3.56 (0.08,168.13)	3.19 (0.14,73.59)	3.62 (0.11,120.42)	3.61 (0.14,94.92)	3.43 (0.15,79.41)	3.80 (0.16,91.50)	3.61 (0.16,81.66)	3.77 (0.16,86.54)	4.38 (0.19,100.43)	5.27 (0.20,135.64)	5.74 (0.21,155.62)	4.92 (0.21,114.93)
0.83 (0.32,2.15)	0.43 (0.02,9.90)	Inject_semaglutide	0.97 (0.58,1.63)	0.98 (0.50,1.91)	1.09 (0.72,1.64)	1.11 (0.71,1.74)	1.10 (0.55,2.20)	1.13 (0.65,1.96)	1.18 (0.85,1.65)	0.96 (0.08,12.19)	1.21 (0.76,1.93)	1.11 (0.14,9.11)	1.28 (0.87,1.87)	1.54 (0.16,15.06)	1.38 (0.89,2.14)	1.56 (0.31,7.91)	1.56 (0.57,4.30)	1.48 (0.93,2.37)	1.64 (0.83,3.25)	***1.56 (1.19,2.05)**	***1.63 (1.10,2.42)**	***1.89 (1.29,2.79)**	2.28 (0.88,5.87)	2.48 (0.82,7.53)	***2.13 (1.27,3.58)**
0.86 (0.31,2.37)	0.44 (0.02,10.38)	1.03 (0.62,1.72)	Canagliflozin	1.01 (0.48,2.14)	1.12 (0.66,1.92)	1.15 (0.65,2.01)	1.14 (0.53,2.46)	1.16 (0.61,2.23)	1.22 (0.75,1.96)	0.99 (0.08,12.85)	1.24 (0.70,2.22)	1.14 (0.14,9.64)	1.31 (0.78,2.20)	1.58 (0.16,15.90)	1.42 (0.81,2.48)	1.61 (0.31,8.44)	1.61 (0.55,4.68)	1.53 (0.85,2.73)	1.69 (0.79,3.63)	***1.61 (1.03,2.49)**	1.68 (0.99,2.83)	***1.95 (1.16,3.27)**	2.34 (0.86,6.42)	2.55 (0.80,8.17)	***2.19 (1.18,4.08)**
0.85 (0.28,2.55)	0.44 (0.02,10.56)	1.02 (0.52,1.98)	0.99 (0.47,2.09)	Sotagliflozin	1.11 (0.56,2.19)	1.14 (0.56,2.29)	1.12 (0.47,2.70)	1.15 (0.53,2.49)	1.20 (0.64,2.27)	0.98 (0.07,13.15)	1.23 (0.60,2.52)	1.13 (0.13,9.93)	1.30 (0.67,2.52)	1.57 (0.15,16.34)	1.40 (0.70,2.82)	1.59 (0.29,8.80)	1.59 (0.50,5.01)	1.51 (0.74,3.09)	1.67 (0.70,4.00)	1.59 (0.87,2.91)	1.66 (0.85,3.25)	1.93 (0.99,3.75)	2.32 (0.78,6.90)	2.53 (0.73,8.69)	***2.17 (1.02,4.59)**
0.76 (0.29,2.02)	0.40 (0.02,9.12)	0.92 (0.61,1.39)	0.89 (0.52,1.53)	0.90 (0.46,1.78)	Empagliflozin	1.02 (0.64,1.64)	1.01 (0.50,2.05)	1.04 (0.59,1.83)	1.09 (0.76,1.56)	0.88 (0.07,11.25)	1.11 (0.68,1.81)	1.02 (0.12,8.41)	1.17 (0.78,1.77)	1.41 (0.14,13.90)	1.27 (0.80,2.02)	1.44 (0.28,7.32)	1.44 (0.52,3.99)	1.36 (0.83,2.23)	1.51 (0.75,3.03)	***1.43 (1.05,1.95)**	1.50 (0.98,2.28)	***1.74 (1.15,2.63)**	2.09 (0.80,5.45)	2.28 (0.74,6.99)	***1.96 (1.14,3.35)**
0.75 (0.28,1.98)	0.39 (0.02,8.94)	0.90 (0.58,1.40)	0.87 (0.50,1.53)	0.88 (0.44,1.78)	0.98 (0.61,1.56)	Dulaglutide	0.99 (0.48,2.04)	1.01 (0.56,1.84)	1.06 (0.71,1.58)	0.86 (0.07,10.78)	1.08 (0.66,1.79)	0.99 (0.12,8.27)	1.14 (0.73,1.78)	1.38 (0.14,13.66)	1.24 (0.75,2.03)	1.40 (0.27,7.21)	1.40 (0.50,3.95)	1.33 (0.79,2.24)	1.47 (0.72,3.02)	1.40 (0.98,1.99)	1.46 (0.93,2.30)	***1.70 (1.09,2.66)**	2.04 (0.77,5.40)	2.23 (0.73,6.81)	***1.91 (1.09,3.36)**
0.75 (0.25,2.30)	0.39 (0.02,9.44)	0.91 (0.45,1.81)	0.88 (0.41,1.90)	0.89 (0.37,2.14)	0.99 (0.49,1.99)	1.01 (0.49,2.08)	Alogliptin	1.02 (0.46,2.26)	1.07 (0.55,2.07)	0.87 (0.06,11.77)	1.09 (0.52,2.30)	1.00 (0.11,8.90)	1.16 (0.58,2.30)	1.39 (0.13,14.63)	1.25 (0.61,2.57)	1.42 (0.25,7.90)	1.41 (0.44,4.52)	1.34 (0.64,2.81)	1.49 (0.61,3.62)	1.41 (0.75,2.66)	1.48 (0.74,2.96)	1.72 (0.86,3.42)	2.06 (0.68,6.23)	2.25 (0.64,7.84)	1.93 (0.89,4.17)
0.74 (0.26,2.08)	0.38 (0.02,8.51)	0.89 (0.51,1.54)	0.86 (0.45,1.65)	0.87 (0.40,1.89)	0.96 (0.55,1.71)	0.99 (0.54,1.79)	0.98 (0.44,2.17)	Ertugliflozin	1.05 (0.62,1.76)	0.85 (0.07,11.15)	1.07 (0.58,1.97)	0.98 (0.12,8.38)	1.13 (0.65,1.96)	1.36 (0.13,13.81)	1.22 (0.68,2.21)	1.39 (0.26,7.35)	1.38 (0.47,4.11)	1.31 (0.71,2.43)	1.46 (0.66,3.20)	1.38 (0.86,2.23)	1.45 (0.83,2.53)	1.68 (0.97,2.92)	2.02 (0.72,5.63)	2.20 (0.68,7.15)	1.89 (0.98,3.62)
0.70 (0.28,1.80)	0.37 (0.02,8.33)	0.85 (0.61,1.18)	0.82 (0.51,1.33)	0.83 (0.44,1.57)	0.92 (0.64,1.32)	0.94 (0.63,1.41)	0.93 (0.48,1.81)	0.95 (0.57,1.60)	Dapagliflozin	0.81 (0.06,10.24)	1.02 (0.67,1.57)	0.94 (0.12,7.64)	1.08 (0.78,1.50)	1.30 (0.14,12.53)	1.17 (0.78,1.73)	1.32 (0.26,6.62)	1.32 (0.49,3.57)	1.25 (0.82,1.92)	1.39 (0.72,2.67)	***1.32 (1.09,1.60)**	1.38 (0.98,1.95)	***1.60 (1.15,2.24)**	1.93 (0.76,4.87)	2.10 (0.70,6.27)	***1.80 (1.11,2.91)**
0.86 (0.06,12.66)	0.45 (0.01,24.82)	1.04 (0.08,13.16)	1.01 (0.08,13.08)	1.02 (0.08,13.68)	1.13 (0.09,14.38)	1.16 (0.09,14.45)	1.15 (0.08,15.48)	1.17 (0.09,15.30)	1.23 (0.10,15.43)	Retatrutide	1.26 (0.10,16.10)	1.15 (0.04,30.48)	1.33 (0.10,16.78)	1.60 (0.05,47.47)	1.43 (0.11,18.30)	1.62 (0.08,32.22)	1.62 (0.11,24.28)	1.54 (0.12,19.77)	1.71 (0.13,22.98)	1.62 (0.13,20.21)	1.69 (0.13,21.49)	1.97 (0.16,24.93)	2.36 (0.16,34.56)	2.58 (0.17,39.96)	2.21 (0.17,28.66)
0.69 (0.25,1.86)	0.36 (0.02,8.18)	0.83 (0.52,1.32)	0.80 (0.45,1.44)	0.81 (0.40,1.67)	0.90 (0.55,1.46)	0.92 (0.56,1.53)	0.91 (0.44,1.92)	0.93 (0.51,1.72)	0.98 (0.64,1.50)	0.80 (0.06,10.22)	Sitagliptin	0.92 (0.11,7.67)	1.06 (0.66,1.68)	1.27 (0.13,12.66)	1.14 (0.68,1.92)	1.29 (0.25,6.69)	1.29 (0.45,3.69)	1.23 (0.72,2.09)	1.36 (0.65,2.83)	1.29 (0.88,1.90)	1.35 (0.84,2.18)	1.57 (0.98,2.51)	1.88 (0.70,5.04)	2.05 (0.66,6.43)	1.76 (0.99,3.14)
0.75 (0.08,7.34)	0.39 (0.01,16.63)	0.90 (0.11,7.41)	0.88 (0.10,7.40)	0.89 (0.10,7.79)	0.98 (0.12,8.10)	1.01 (0.12,8.35)	1.00 (0.11,8.82)	1.02 (0.12,8.67)	1.07 (0.13,8.67)	0.87 (0.03,22.98)	1.09 (0.13,9.11)	Teneligliptin	1.15 (0.14,9.44)	1.39 (0.06,30.20)	1.24 (0.15,10.32)	1.41 (0.10,19.55)	1.41 (0.14,14.11)	1.34 (0.16,11.16)	1.48 (0.17,13.09)	1.41 (0.17,11.34)	1.47 (0.18,12.10)	1.71 (0.21,14.03)	2.05 (0.21,19.99)	2.24 (0.21,23.43)	1.92 (0.23,16.21)
0.65 (0.25,1.70)	0.34 (0.01,7.75)	0.78 (0.54,1.15)	0.76 (0.45,1.27)	0.77 (0.40,1.49)	0.85 (0.57,1.28)	0.87 (0.56,1.36)	0.87 (0.43,1.72)	0.88 (0.51,1.53)	0.93 (0.67,1.29)	0.75 (0.06,9.55)	0.95 (0.59,1.51)	0.87 (0.11,7.13)	Liraglutide	1.21 (0.12,11.79)	1.08 (0.70,1.68)	1.23 (0.24,6.20)	1.22 (0.45,3.37)	1.16 (0.73,1.85)	1.29 (0.65,2.54)	1.22 (0.93,1.60)	1.28 (0.87,1.89)	***1.48 (1.01,2.18)**	1.78 (0.69,4.59)	1.94 (0.64,5.90)	1.67 (1.00,2.80)
0.54 (0.05,6.22)	0.28 (0.01,13.25)	0.65 (0.07,6.36)	0.63 (0.06,6.33)	0.64 (0.06,6.65)	0.71 (0.07,6.95)	0.72 (0.07,7.16)	0.72 (0.07,7.53)	0.73 (0.07,7.41)	0.77 (0.08,7.39)	0.63 (0.02,18.57)	0.79 (0.08,7.80)	0.72 (0.03,15.67)	0.83 (0.08,8.10)	Dapagliflozin_ Saxagliptin	0.90 (0.09,8.85)	1.02 (0.06,16.23)	1.01 (0.09,11.94)	0.96 (0.10,9.56)	1.07 (0.10,11.17)	1.01 (0.11,9.75)	1.06 (0.11,10.38)	1.23 (0.13,11.84)	1.48 (0.13,16.95)	1.61 (0.13,19.77)	1.38 (0.14,13.88)
0.60 (0.23,1.61)	0.31 (0.01,7.23)	0.73 (0.47,1.13)	0.70 (0.40,1.23)	0.71 (0.35,1.43)	0.79 (0.50,1.26)	0.81 (0.49,1.33)	0.80 (0.39,1.65)	0.82 (0.45,1.48)	0.86 (0.58,1.27)	0.70 (0.05,8.93)	0.88 (0.52,1.47)	0.80 (0.10,6.68)	0.93 (0.60,1.44)	1.12 (0.11,11.03)	Linagliptin	1.13 (0.22,5.82)	1.13 (0.40,3.19)	1.07 (0.64,1.80)	1.19 (0.58,2.44)	1.13 (0.80,1.60)	1.18 (0.76,1.85)	1.37 (0.88,2.14)	1.65 (0.63,4.36)	1.80 (0.58,5.58)	1.54 (0.88,2.71)
0.53 (0.08,3.36)	0.28 (0.01,9.20)	0.64 (0.13,3.24)	0.62 (0.12,3.26)	0.63 (0.11,3.47)	0.70 (0.14,3.54)	0.71 (0.14,3.66)	0.71 (0.13,3.94)	0.72 (0.14,3.83)	0.76 (0.15,3.78)	0.62 (0.03,12.21)	0.77 (0.15,4.00)	0.71 (0.05,9.83)	0.82 (0.16,4.12)	0.98 (0.06,15.73)	0.88 (0.17,4.52)	Vildagliptin	1.00 (0.15,6.49)	0.95 (0.18,4.90)	1.05 (0.19,5.84)	1.00 (0.20,4.93)	1.04 (0.21,5.29)	1.21 (0.24,6.13)	1.46 (0.23,9.14)	1.59 (0.23,10.89)	1.36 (0.26,7.14)
0.53 (0.14,2.03)	0.28 (0.01,7.27)	0.64 (0.23,1.76)	0.62 (0.21,1.81)	0.63 (0.20,1.98)	0.70 (0.25,1.94)	0.71 (0.25,2.01)	0.71 (0.22,2.26)	0.72 (0.24,2.14)	0.76 (0.28,2.04)	0.62 (0.04,9.23)	0.77 (0.27,2.21)	0.71 (0.07,7.11)	0.82 (0.30,2.25)	0.99 (0.08,11.59)	0.88 (0.31,2.48)	1.00 (0.15,6.51)	Bexagliflozin	0.95 (0.33,2.70)	1.05 (0.33,3.35)	1.00 (0.38,2.65)	1.04 (0.38,2.88)	1.21 (0.44,3.34)	1.46 (0.38,5.52)	1.59 (0.37,6.79)	1.36 (0.47,3.97)
0.56 (0.21,1.52)	0.29 (0.01,6.76)	0.67 (0.42,1.08)	0.66 (0.37,1.17)	0.66 (0.32,1.36)	0.73 (0.45,1.20)	0.75 (0.45,1.27)	0.75 (0.36,1.56)	0.76 (0.41,1.41)	0.80 (0.52,1.22)	0.65 (0.05,8.35)	0.82 (0.48,1.39)	0.75 (0.09,6.25)	0.86 (0.54,1.37)	1.04 (0.10,10.32)	0.93 (0.55,1.56)	1.06 (0.20,5.46)	1.05 (0.37,3.00)	Albiglutide	1.11 (0.53,2.31)	1.05 (0.72,1.54)	1.10 (0.68,1.77)	1.28 (0.80,2.05)	1.54 (0.57,4.11)	1.67 (0.53,5.25)	1.44 (0.80,2.57)
0.51 (0.17,1.54)	0.26 (0.01,6.34)	0.61 (0.31,1.21)	0.59 (0.28,1.27)	0.60 (0.25,1.43)	0.66 (0.33,1.33)	0.68 (0.33,1.39)	0.67 (0.28,1.64)	0.69 (0.31,1.51)	0.72 (0.37,1.39)	0.59 (0.04,7.90)	0.74 (0.35,1.53)	0.68 (0.08,5.97)	0.78 (0.39,1.54)	0.94 (0.09,9.82)	0.84 (0.41,1.72)	0.95 (0.17,5.30)	0.95 (0.30,3.03)	0.90 (0.43,1.88)	Lixisenatide	0.95 (0.51,1.78)	0.99 (0.50,1.98)	1.15 (0.58,2.28)	1.39 (0.46,4.17)	1.51 (0.44,5.25)	1.30 (0.60,2.79)
0.53 (0.21,1.34)	0.28 (0.01,6.27)	***0.64 (0.49,0.84)**	***0.62 (0.40,0.97)**	0.63 (0.34,1.15)	***0.70 (0.51,0.95)**	0.71 (0.50,1.02)	0.71 (0.38,1.33)	0.72 (0.45,1.17)	***0.76 (0.63,0.92)**	0.62 (0.05,7.71)	0.78 (0.53,1.14)	0.71 (0.09,5.74)	0.82 (0.63,1.07)	0.99 (0.10,9.50)	0.88 (0.62,1.25)	1.00 (0.20,4.96)	1.00 (0.38,2.66)	0.95 (0.65,1.39)	1.05 (0.56,1.97)	Placebo_or_Control	1.05 (0.79,1.39)	1.22 (0.92,1.60)	1.46 (0.59,3.62)	1.59 (0.54,4.67)	1.36 (0.88,2.12)
0.51 (0.19,1.34)	0.26 (0.01,6.08)	***0.61 (0.41,0.91)**	0.60 (0.35,1.01)	0.60 (0.31,1.18)	0.67 (0.44,1.02)	0.68 (0.43,1.07)	0.68 (0.34,1.36)	0.69 (0.40,1.21)	0.72 (0.51,1.02)	0.59 (0.05,7.49)	0.74 (0.46,1.20)	0.68 (0.08,5.59)	0.78 (0.53,1.16)	0.94 (0.10,9.24)	0.85 (0.54,1.32)	0.96 (0.19,4.86)	0.96 (0.35,2.64)	0.91 (0.56,1.46)	1.01 (0.51,2.00)	0.96 (0.72,1.27)	Exenatide	1.16 (0.78,1.72)	1.40 (0.54,3.61)	1.52 (0.50,4.63)	1.30 (0.77,2.21)
0.44 (0.17,1.15)	0.23 (0.01,5.22)	***0.53 (0.36,0.78)**	***0.51 (0.31,0.86)**	0.52 (0.27,1.01)	***0.57 (0.38,0.87)**	***0.59 (0.38,0.92)**	0.58 (0.29,1.16)	0.60 (0.34,1.03)	***0.62 (0.45,0.87)**	0.51 (0.04,6.44)	0.64 (0.40,1.02)	0.59 (0.07,4.81)	***0.67 (0.46,0.99)**	0.81 (0.08,7.82)	0.73 (0.47,1.13)	0.83 (0.16,4.18)	0.82 (0.30,2.27)	0.78 (0.49,1.25)	0.87 (0.44,1.72)	0.82 (0.63,1.08)	0.86 (0.58,1.28)	Saxagliptin	1.20 (0.47,3.10)	1.31 (0.43,3.98)	1.12 (0.67,1.89)
0.37 (0.10,1.33)	0.19 (0.01,4.89)	0.44 (0.17,1.13)	0.43 (0.16,1.17)	0.43 (0.14,1.28)	0.48 (0.18,1.25)	0.49 (0.19,1.30)	0.49 (0.16,1.47)	0.50 (0.18,1.38)	0.52 (0.21,1.31)	0.42 (0.03,6.18)	0.53 (0.20,1.42)	0.49 (0.05,4.74)	0.56 (0.22,1.44)	0.68 (0.06,7.75)	0.61 (0.23,1.60)	0.69 (0.11,4.31)	0.69 (0.18,2.60)	0.65 (0.24,1.74)	0.72 (0.24,2.17)	0.69 (0.28,1.70)	0.72 (0.28,1.85)	0.83 (0.32,2.15)	Omarigliptin	1.09 (0.27,4.45)	0.93 (0.34,2.56)
0.34 (0.08,1.38)	0.17 (0.01,4.72)	0.40 (0.13,1.22)	0.39 (0.12,1.25)	0.40 (0.12,1.36)	0.44 (0.14,1.34)	0.45 (0.15,1.38)	0.44 (0.13,1.55)	0.45 (0.14,1.48)	0.48 (0.16,1.42)	0.39 (0.03,6.02)	0.49 (0.16,1.53)	0.45 (0.04,4.68)	0.51 (0.17,1.56)	0.62 (0.05,7.61)	0.56 (0.18,1.72)	0.63 (0.09,4.33)	0.63 (0.15,2.69)	0.60 (0.19,1.87)	0.66 (0.19,2.30)	0.63 (0.21,1.84)	0.66 (0.22,2.00)	0.76 (0.25,2.32)	0.92 (0.22,3.75)	Efpeglenatide	0.86 (0.27,2.74)
0.39 (0.14,1.08)	0.20 (0.01,4.74)	***0.47 (0.28,0.79)**	***0.46 (0.25,0.85)**	***0.46 (0.22,0.98)**	***0.51 (0.30,0.88)**	***0.52 (0.30,0.92)**	0.52 (0.24,1.12)	0.53 (0.28,1.02)	***0.56 (0.34,0.90)**	0.45 (0.03,5.87)	0.57 (0.32,1.01)	0.52 (0.06,4.40)	0.60 (0.36,1.00)	0.72 (0.07,7.26)	0.65 (0.37,1.14)	0.73 (0.14,3.86)	0.73 (0.25,2.14)	0.70 (0.39,1.25)	0.77 (0.36,1.66)	0.73 (0.47,1.14)	0.77 (0.45,1.30)	0.89 (0.53,1.50)	1.07 (0.39,2.93)	1.17 (0.36,3.73)	Oral_semaglutide

Data presents RR [95%CIs]. Network meta-analysis results are presented as estimate effect sizes for the outcome of overall asthma-COPD overlap syndrome risk. Interventions are reported in order of mean ranking of beneficially prophylactic effect on overall asthma-COPD overlap syndrome risk, and outcomes are expressed as risk ratio (RR) (95% confidence intervals) (95%CIs). For the upper-right portion, RR of less than 1 indicates that the treatment specified in the row got more beneficial effect than that specified in the column. For the lower-left portion, RR of less than 1 indicates that the treatment specified in the column has more beneficial effect than that specified in the row. Bold results marked with * indicate statistical significance.

eTable 5B League table of primary outcome: overall asthma-COPD overlap syndrome risk focusing on participants with diabetes mellitus

Tirzepatide	0.60 (0.02,16.68)	1.39 (0.41,4.71)	1.37 (0.38,4.96)	1.43 (0.40,5.04)	1.56 (0.48,5.04)	1.54 (0.42,5.65)	1.58 (0.46,5.40)	1.64 (0.49,5.47)	1.69 (0.51,5.58)	1.34 (0.08,21.34)	1.74 (0.54,5.59)	1.55 (0.14,16.67)	1.82 (0.57,5.84)	1.93 (0.59,6.30)	2.26 (0.18,28.47)	2.18 (0.31,15.50)	2.18 (0.49,9.73)	2.07 (0.62,6.85)	2.29 (0.63,8.37)	2.18 (0.70,6.77)	2.28 (0.71,7.34)	2.65 (0.82,8.50)	3.18 (0.74,13.58)	3.47 (0.73,16.53)	2.97 (0.88,10.03)
1.66 (0.06,45.79)	Ertugliflozin_ Sitagliptin	2.31 (0.10,53.92)	2.27 (0.09,54.51)	2.36 (0.10,56.07)	2.58 (0.11,59.51)	2.55 (0.11,61.62)	2.61 (0.12,57.97)	2.71 (0.12,63.95)	2.80 (0.12,63.99)	2.23 (0.04,123.18)	2.89 (0.13,66.10)	2.57 (0.06,109.51)	3.02 (0.13,69.07)	3.19 (0.14,73.60)	3.74 (0.08,176.83)	3.62 (0.11,120.42)	3.61 (0.14,94.93)	3.43 (0.15,79.39)	3.80 (0.16,91.50)	3.61 (0.16,81.66)	3.78 (0.16,86.64)	4.39 (0.19,100.47)	5.27 (0.20,135.64)	5.74 (0.21,155.63)	4.92 (0.21,114.93)
0.72 (0.21,2.42)	0.43 (0.02,10.11)	Canagliflozin	0.98 (0.46,2.09)	1.02 (0.50,2.08)	1.12 (0.63,1.97)	1.11 (0.51,2.40)	1.13 (0.59,2.17)	1.18 (0.60,2.29)	1.21 (0.68,2.17)	0.96 (0.07,12.52)	1.25 (0.74,2.11)	1.11 (0.13,9.39)	1.31 (0.78,2.20)	1.38 (0.79,2.43)	1.62 (0.16,16.31)	1.57 (0.30,8.23)	1.57 (0.54,4.57)	1.48 (0.83,2.67)	1.65 (0.76,3.54)	*1.56 (1.01,2.44)	1.64 (0.97,2.77)	*1.90 (1.13,3.20)	2.28 (0.83,6.26)	2.49 (0.78,7.97)	*2.13 (1.14,3.99)
0.73 (0.20,2.64)	0.44 (0.02,10.56)	1.02 (0.48,2.15)	Sotagliflozin	1.04 (0.46,2.36)	1.14 (0.56,2.29)	1.12 (0.47,2.70)	1.15 (0.53,2.49)	1.19 (0.54,2.63)	1.23 (0.60,2.52)	0.98 (0.07,13.15)	1.27 (0.65,2.47)	1.13 (0.13,9.93)	1.33 (0.68,2.58)	1.40 (0.70,2.82)	1.65 (0.16,17.20)	1.59 (0.29,8.80)	1.59 (0.50,5.01)	1.51 (0.74,3.09)	1.67 (0.70,4.00)	1.59 (0.87,2.91)	1.66 (0.85,3.25)	1.93 (0.99,3.75)	2.32 (0.78,6.90)	2.53 (0.73,8.69)	*2.17 (1.02,4.59)
0.70 (0.20,2.48)	0.42 (0.02,10.06)	0.98 (0.48,1.99)	0.96 (0.42,2.19)	Empagliflozin	1.09 (0.57,2.11)	1.08 (0.47,2.51)	1.11 (0.53,2.30)	1.15 (0.54,2.43)	1.18 (0.61,2.29)	0.94 (0.07,12.51)	1.22 (0.66,2.27)	1.09 (0.13,9.43)	1.28 (0.69,2.37)	1.35 (0.70,2.60)	1.58 (0.15,16.34)	1.53 (0.28,8.32)	1.53 (0.50,4.70)	1.45 (0.74,2.85)	1.61 (0.70,3.71)	1.53 (0.88,2.66)	1.60 (0.86,2.99)	1.86 (1.00,3.45)	2.23 (0.77,6.46)	2.43 (0.72,8.16)	*2.09 (1.03,4.23)
0.64 (0.20,2.08)	0.39 (0.02,8.94)	0.90 (0.51,1.58)	0.88 (0.44,1.78)	0.92 (0.47,1.76)	Dulaglutide	0.99 (0.48,2.04)	1.01 (0.56,1.84)	1.05 (0.57,1.94)	1.08 (0.66,1.79)	0.86 (0.07,10.78)	1.12 (0.72,1.75)	0.99 (0.12,8.27)	1.17 (0.75,1.82)	1.24 (0.75,2.03)	1.45 (0.15,14.37)	1.40 (0.27,7.21)	1.40 (0.50,3.95)	1.33 (0.79,2.24)	1.47 (0.72,3.02)	1.40 (0.98,1.99)	1.46 (0.93,2.30)	1.70 (1.09,2.66)	2.04 (0.77,5.40)	2.23 (0.73,6.81)	*1.91 (1.09,3.36)
0.65 (0.18,2.38)	0.39 (0.02,9.44)	0.90 (0.42,1.96)	0.89 (0.37,2.14)	0.92 (0.40,2.14)	1.01 (0.49,2.08)	Alogliptin	1.02 (0.46,2.26)	1.06 (0.47,2.39)	1.09 (0.52,2.30)	0.87 (0.06,11.77)	1.13 (0.57,2.25)	1.00 (0.11,8.90)	1.18 (0.59,2.35)	1.25 (0.61,2.57)	1.46 (0.14,15.40)	1.42 (0.25,7.90)	1.41 (0.44,4.52)	1.34 (0.64,2.81)	1.49 (0.61,3.62)	1.41 (0.75,2.66)	1.48 (0.74,2.96)	1.72 (0.86,3.42)	2.06 (0.68,6.23)	2.25 (0.64,7.84)	1.93 (0.89,4.17)
0.63 (0.19,2.18)	0.38 (0.02,8.51)	0.88 (0.46,1.70)	0.87 (0.40,1.89)	0.90 (0.43,1.88)	0.99 (0.54,1.79)	0.98 (0.44,2.17)	Ertugliflozin	1.04 (0.52,2.09)	1.07 (0.58,1.97)	0.85 (0.07,11.15)	1.11 (0.64,1.92)	0.98 (0.12,8.38)	1.16 (0.67,2.01)	1.22 (0.68,2.21)	1.43 (0.14,14.54)	1.39 (0.26,7.35)	1.38 (0.47,4.11)	1.31 (0.71,2.43)	1.46 (0.66,3.20)	1.38 (0.86,2.23)	1.45 (0.83,2.53)	1.68 (0.97,2.92)	2.02 (0.72,5.63)	2.20 (0.68,7.15)	1.89 (0.98,3.62)
0.61 (0.18,2.04)	0.37 (0.02,8.68)	0.85 (0.44,1.65)	0.84 (0.38,1.84)	0.87 (0.41,1.84)	0.95 (0.52,1.75)	0.94 (0.42,2.12)	0.96 (0.48,1.93)	Inject_semaglutide	1.03 (0.55,1.93)	0.82 (0.06,10.77)	1.06 (0.60,1.89)	0.95 (0.11,8.10)	1.11 (0.63,1.97)	1.18 (0.64,2.17)	1.38 (0.14,14.05)	1.33 (0.25,7.13)	1.33 (0.44,3.99)	1.26 (0.67,2.38)	1.40 (0.63,3.13)	1.33 (0.80,2.20)	1.39 (0.78,2.48)	1.62 (0.91,2.87)	1.94 (0.69,5.48)	2.12 (0.64,6.94)	1.81 (0.93,3.55)
0.59 (0.18,1.96)	0.36 (0.02,8.19)	0.83 (0.46,1.48)	0.81 (0.40,1.67)	0.84 (0.44,1.63)	0.92 (0.56,1.53)	0.91 (0.44,1.92)	0.93 (0.51,1.72)	0.97 (0.52,1.82)	Sitagliptin	0.80 (0.06,10.22)	1.03 (0.65,1.65)	0.92 (0.11,7.67)	1.08 (0.67,1.73)	1.14 (0.68,1.92)	1.34 (0.13,13.33)	1.29 (0.25,6.70)	1.29 (0.45,3.69)	1.23 (0.72,2.09)	1.36 (0.65,2.83)	1.29 (0.88,1.90)	1.35 (0.84,2.18)	1.57 (0.98,2.52)	1.88 (0.70,5.05)	2.05 (0.66,6.43)	1.76 (0.99,3.14)
0.74 (0.05,11.80)	0.45 (0.01,24.81)	1.04 (0.08,13.45)	1.02 (0.08,13.68)	1.06 (0.08,14.05)	1.16 (0.09,14.45)	1.15 (0.08,15.48)	1.17 (0.09,15.30)	1.22 (0.09,15.97)	1.25 (0.10,16.10)	Retatrutide	1.30 (0.10,16.41)	1.15 (0.04,30.48)	1.35 (0.11,17.14)	1.43 (0.11,18.30)	1.68 (0.06,49.93)	1.62 (0.08,32.22)	1.62 (0.11,24.28)	1.54 (0.12,19.76)	1.71 (0.13,22.97)	1.62 (0.13,20.21)	1.70 (0.13,21.51)	1.97 (0.16,24.94)	2.36 (0.16,34.56)	2.58 (0.17,39.96)	2.21 (0.17,28.66)
0.57 (0.18,1.84)	0.35 (0.02,7.94)	0.80 (0.47,1.35)	0.79 (0.40,1.53)	0.82 (0.44,1.52)	0.89 (0.57,1.40)	0.89 (0.44,1.76)	0.90 (0.52,1.57)	0.94 (0.53,1.67)	0.97 (0.61,1.55)	0.77 (0.06,9.78)	Liraglutide	0.89 (0.11,7.30)	1.04 (0.71,1.54)	1.11 (0.71,1.72)	1.30 (0.13,12.71)	1.25 (0.25,6.34)	1.25 (0.45,3.45)	1.19 (0.74,1.90)	1.32 (0.66,2.61)	1.25 (0.95,1.64)	1.31 (0.88,1.94)	*1.52 (1.03,2.24)	1.82 (0.71,4.71)	1.99 (0.66,6.04)	*1.71 (1.02,2.87)
0.65 (0.06,6.95)	0.39 (0.01,16.63)	0.90 (0.11,7.61)	0.89 (0.10,7.79)	0.92 (0.11,7.98)	1.01 (0.12,8.35)	1.00 (0.11,8.82)	1.02 (0.12,8.67)	1.06 (0.12,9.06)	1.09 (0.13,9.10)	0.87 (0.03,22.98)	1.12 (0.14,9.24)	Teneligliptin	1.18 (0.14,9.65)	1.24 (0.15,10.32)	1.46 (0.07,31.77)	1.41 (0.10,19.55)	1.41 (0.14,14.11)	1.34 (0.16,11.16)	1.48 (0.17,13.09)	1.41 (0.17,11.34)	1.47 (0.18,12.11)	1.71 (0.21,14.04)	2.05 (0.21,19.99)	2.24 (0.21,23.43)	1.92 (0.23,16.21)
0.55 (0.17,1.76)	0.33 (0.01,7.59)	0.77 (0.46,1.29)	0.75 (0.39,1.46)	0.78 (0.42,1.45)	0.86 (0.55,1.33)	0.85 (0.43,1.69)	0.87 (0.50,1.50)	0.90 (0.51,1.60)	0.93 (0.58,1.48)	0.74 (0.06,9.36)	0.96 (0.65,1.41)	0.85 (0.10,6.98)	Dapagliflozin	1.06 (0.68,1.64)	1.24 (0.13,11.96)	1.20 (0.24,6.07)	1.20 (0.44,3.30)	1.14 (0.71,1.82)	1.26 (0.64,2.49)	1.20 (0.91,1.57)	1.25 (0.85,1.86)	1.45 (0.99,2.14)	1.75 (0.68,4.50)	1.90 (0.63,5.78)	1.63 (0.97,2.74)
0.52 (0.16,1.70)	0.31 (0.01,7.23)	0.72 (0.41,1.27)	0.71 (0.35,1.43)	0.74 (0.38,1.42)	0.81 (0.49,1.33)	0.80 (0.39,1.65)	0.82 (0.45,1.48)	0.85 (0.46,1.57)	0.88 (0.52,1.47)	0.70 (0.05,8.93)	0.90 (0.58,1.41)	0.80 (0.10,6.68)	0.95 (0.61,1.47)	Linagliptin	1.17 (0.12,11.61)	1.13 (0.22,5.82)	1.13 (0.40,3.19)	1.07 (0.64,1.80)	1.19 (0.58,2.44)	1.13 (0.80,1.60)	1.18 (0.76,1.86)	1.38 (0.88,2.14)	1.65 (0.63,4.36)	1.80 (0.58,5.58)	1.54 (0.88,2.71)
0.44 (0.04,5.58)	0.27 (0.01,12.63)	0.62 (0.06,6.21)	0.61 (0.06,6.34)	0.63 (0.06,6.50)	0.69 (0.07,6.83)	0.68 (0.06,7.18)	0.70 (0.07,7.07)	0.73 (0.07,7.39)	0.75 (0.08,7.44)	0.60 (0.02,17.70)	0.77 (0.08,7.56)	0.69 (0.03,14.94)	0.81 (0.08,7.77)	0.85 (0.09,8.44)	Dapagliflozin_ Saxagliptin	0.97 (0.06,15.48)	0.97 (0.08,11.38)	0.92 (0.09,9.12)	1.02 (0.10,10.66)	0.96 (0.10,9.30)	1.01 (0.10,9.91)	1.17 (0.12,11.30)	1.41 (0.12,16.16)	1.53 (0.12,18.86)	1.32 (0.13,13.24)
0.46 (0.06,3.25)	0.28 (0.01,9.20)	0.64 (0.12,3.35)	0.63 (0.11,3.47)	0.65 (0.12,3.54)	0.71 (0.14,3.66)	0.71 (0.13,3.94)	0.72 (0.14,3.83)	0.75 (0.14,4.01)	0.77 (0.15,4.00)	0.62 (0.03,12.21)	0.80 (0.16,4.03)	0.71 (0.05,9.83)	0.83 (0.16,4.21)	0.88 (0.17,4.52)	1.03 (0.06,16.55)	Vildagliptin	1.00 (0.15,6.49)	0.95 (0.18,4.90)	1.05 (0.19,5.84)	1.00 (0.20,4.93)	1.04 (0.21,5.29)	1.21 (0.24,6.13)	1.46 (0.23,9.14)	1.59 (0.23,10.89)	1.36 (0.26,7.14)
0.46 (0.10,2.05)	0.28 (0.01,7.27)	0.64 (0.22,1.86)	0.63 (0.20,1.98)	0.65 (0.21,2.01)	0.71 (0.25,2.01)	0.71 (0.22,2.26)	0.72 (0.24,2.14)	0.75 (0.25,2.25)	0.77 (0.27,2.21)	0.62 (0.04,9.23)	0.80 (0.29,2.20)	0.71 (0.07,7.11)	0.83 (0.30,2.30)	0.88 (0.31,2.48)	1.04 (0.09,12.20)	1.00 (0.15,6.51)	Bexagliflozin	0.95 (0.33,2.70)	1.05 (0.33,3.35)	1.00 (0.38,2.65)	1.05 (0.38,2.89)	1.21 (0.44,3.34)	1.46 (0.38,5.52)	1.59 (0.37,6.79)	1.36 (0.47,3.97)
0.48 (0.15,1.60)	0.29 (0.01,6.76)	0.67 (0.37,1.21)	0.66 (0.32,1.36)	0.69 (0.35,1.35)	0.75 (0.45,1.27)	0.75 (0.36,1.56)	0.76 (0.41,1.41)	0.79 (0.42,1.49)	0.82 (0.48,1.39)	0.65 (0.05,8.35)	0.84 (0.53,1.34)	0.75 (0.09,6.25)	0.88 (0.55,1.41)	0.93 (0.56,1.56)	1.09 (0.11,10.87)	1.06 (0.20,5.46)	1.05 (0.37,3.01)	Albiglutide	1.11 (0.53,2.31)	1.05 (0.72,1.54)	1.10 (0.68,1.78)	1.28 (0.80,2.05)	1.54 (0.57,4.11)	1.68 (0.53,5.25)	1.44 (0.80,2.58)
0.44 (0.12,1.59)	0.26 (0.01,6.34)	0.61 (0.28,1.31)	0.60 (0.25,1.43)	0.62 (0.27,1.43)	0.68 (0.33,1.39)	0.67 (0.28,1.64)	0.69 (0.31,1.51)	0.71 (0.32,1.60)	0.74 (0.35,1.53)	0.59 (0.04,7.90)	0.76 (0.38,1.50)	0.68 (0.08,5.97)	0.79 (0.40,1.57)	0.84 (0.41,1.72)	0.98 (0.09,10.34)	0.95 (0.17,5.30)	0.95 (0.30,3.03)	0.90 (0.43,1.88)	Lixisenatide	0.95 (0.51,1.78)	0.99 (0.50,1.98)	1.15 (0.58,2.29)	1.39 (0.46,4.17)	1.51 (0.44,5.25)	1.30 (0.60,2.79)
0.46 (0.15,1.43)	0.28 (0.01,6.27)	*0.64 (0.41,0.99)	0.63 (0.34,1.15)	0.65 (0.38,1.14)	0.71 (0.50,1.02)	0.71 (0.38,1.33)	0.72 (0.45,1.17)	0.75 (0.45,1.25)	0.77 (0.53,1.14)	0.62 (0.05,7.71)	0.80 (0.61,1.05)	0.71 (0.09,5.74)	0.84 (0.64,1.10)	0.88 (0.62,1.25)	1.04 (0.11,10.00)	1.00 (0.20,4.96)	1.00 (0.38,2.66)	0.95 (0.65,1.39)	1.05 (0.56,1.97)	Placebo_or_Contro l	1.05 (0.79,1.39)	1.22 (0.92,1.60)	1.46 (0.59,3.62)	1.59 (0.54,4.67)	1.36 (0.88,2.12)
0.44 (0.14,1.41)	0.26 (0.01,6.07)	0.61 (0.36,1.04)	0.60 (0.31,1.17)	0.62 (0.33,1.17)	0.68 (0.43,1.07)	0.68 (0.34,1.35)	0.69 (0.40,1.21)	0.72 (0.40,1.28)	0.74 (0.46,1.19)	0.59 (0.05,7.48)	0.76 (0.52,1.13)	0.68 (0.08,5.59)	0.80 (0.54,1.18)	0.84 (0.54,1.32)	0.99 (0.10,9.72)	0.96 (0.19,4.85)	0.96 (0.35,2.64)	0.91 (0.56,1.46)	1.01 (0.51,2.00)	0.95 (0.72,1.27)	Exenatide	1.16 (0.78,1.72)	1.39 (0.54,3.61)	1.52 (0.50,4.63)	1.30 (0.77,2.20)
0.38 (0.12,1.21)	0.23 (0.01,5.22)	*0.53 (0.31,0.89)	0.52 (0.27,1.01)	0.54 (0.29,1.00)	0.59 (0.38,0.92)	0.58 (0.29,1.16)	0.60 (0.34,1.03)	0.62 (0.35,1.10)	0.64 (0.40,1.02)	0.51 (0.04,6.43)	*0.66 (0.45,0.97)	0.59 (0.07,4.80)	0.69 (0.47,1.01)	0.73 (0.47,1.13)	0.85 (0.09,8.22)	0.83 (0.16,4.17)	0.82 (0.30,2.27)	0.78 (0.49,1.25)	0.87 (0.44,1.71)	0.82 (0.63,1.08)	0.86 (0.58,1.28)	Saxagliptin	1.20 (0.47,3.10)	1.31 (0.43,3.97)	1.12 (0.67,1.89)
0.31 (0.07,1.34)	0.19 (0.01,4.89)	0.44 (0.16,1.20)	0.43 (0.14,1.28)	0.45 (0.15,1.30)	0.49 (0.19,1.30)	0.49 (0.16,1.47)	0.50 (0.18,1.38)	0.52 (0.18,1.45)	0.53 (0.20,1.42)	0.42 (0.03,6.18)	0.55 (0.21,1.41)	0.49 (0.05,4.74)	0.57 (0.22,1.48)	0.61 (0.23,1.60)	0.71 (0.06,8.16)	0.69 (0.11,4.31)	0.69 (0.18,2.60)	0.65 (0.24,1.74)	0.72 (0.24,2.17)	0.69 (0.28,1.70)	0.72 (0.28,1.86)	0.83 (0.32,2.15)	Omarigliptin	1.09 (0.27,4.45)	0.93 (0.34,2.56)
0.29 (0.06,1.38)	0.17 (0.01,4.72)	0.40 (0.13,1.29)	0.40 (0.12,1.36)	0.41 (0.12,1.38)	0.45 (0.15,1.38)	0.44 (0.13,1.55)	0.45 (0.14,1.48)	0.47 (0.14,1.55)	0.49 (0.16,1.53)	0.39 (0.03,6.02)	0.50 (0.17,1.53)	0.45 (0.04,4.68)	0.53 (0.17,1.59)	0.56 (0.18,1.72)	0.65 (0.05,8.01)	0.63 (0.09,4.33)	0.63 (0.15,2.69)	0.60 (0.19,1.87)	0.66 (0.19,2.30)	0.63 (0.21,1.84)	0.66 (0.22,2.00)	0.76 (0.25,2.32)	0.92 (0.22,3.75)	Efpeglenatide	0.86 (0.27,2.74)
0.34 (0.10,1.14)	0.20 (0.01,4.74)	*0.47 (0.25,0.88)	*0.46 (0.22,0.98)	*0.48 (0.24,0.97)	*0.52 (0.30,0.92)	0.52 (0.24,1.12)	0.53 (0.28,1.02)	0.55 (0.28,1.08)	0.57 (0.32,1.01)	0.45 (0.03,5.87)	*0.59 (0.35,0.98)	0.52 (0.06,4.40)	0.61 (0.37,1.03)	0.65 (0.37,1.14)	0.76 (0.08,7.64)	0.73 (0.14,3.86)	0.73 (0.25,2.14)	0.70 (0.39,1.25)	0.77 (0.36,1.66)	0.73 (0.47,1.14)	0.77 (0.45,1.30)	0.89 (0.53,1.50)	1.07 (0.39,2.93)	1.17 (0.36,3.73)	Oral_semaglutide

Data presents RR [95%CIs]. Network meta-analysis results are presented as estimate effect sizes for the outcome of overall asthma-COPD overlap syndrome risk. Interventions are reported in order of mean ranking of beneficially prophylactic effect on overall asthma-COPD overlap syndrome risk, and outcomes are expressed as risk ratio (RR) (95% confidence intervals) (95%CIs). For the upper-right portion, RR of less than 1 indicates that the treatment specified in the row got more beneficial effect than that specified in the column. For the lower-left portion, RR of less than 1 indicates that the treatment specified in the column has more beneficial effect than that specified in the row. Bold results marked with * indicate statistical significance.

eTable 5C League table of primary outcome: overall asthma-COPD overlap syndrome risk focusing on participants with age younger than 65 years old

Inject_semaglutide	0.49 (0.02,11.23)	0.99 (0.59,1.67)	0.93 (0.34,2.52)	1.12 (0.56,2.24)	0.91 (0.11,7.80)	1.16 (0.66,2.05)	1.19 (0.67,2.11)	1.24 (0.86,1.80)	1.12 (0.14,9.23)	1.28 (0.87,1.90)	1.40 (0.13,15.10)	1.36 (0.10,17.94)	1.49 (0.38,5.92)	1.61 (0.06,39.72)	1.58 (0.31,8.02)	1.58 (0.57,4.37)	1.51 (0.94,2.44)	1.52 (0.87,2.63)	1.66 (0.83,3.31)	*1.58 (1.18,2.11)	*1.66 (1.11,2.48)	1.78 (0.84,3.77)	2.21 (0.83,5.91)	2.30 (0.89,5.96)	2.73 (0.89,8.36)
2.05 (0.09,47.24)	Ertugliflozin_ Sitagliptin	2.03 (0.09,47.47)	1.91 (0.07,50.10)	2.29 (0.09,55.47)	1.87 (0.04,80.30)	2.39 (0.11,53.22)	2.44 (0.10,57.49)	2.55 (0.11,58.51)	2.30 (0.05,98.52)	2.63 (0.11,60.51)	2.87 (0.06,144.13)	2.80 (0.05,157.91)	3.06 (0.10,91.89)	3.29 (0.04,287.35)	3.24 (0.10,108.37)	3.24 (0.12,85.45)	3.10 (0.13,72.14)	3.11 (0.13,73.20)	3.40 (0.14,82.38)	3.23 (0.14,73.52)	3.40 (0.15,78.24)	3.65 (0.15,86.07)	4.53 (0.18,116.75)	4.72 (0.18,122.10)	5.60 (0.21,152.44)
1.01 (0.60,1.71)	0.49 (0.02,11.57)	Canagliflozin	0.94 (0.33,2.71)	1.13 (0.52,2.44)	0.92 (0.10,8.10)	1.18 (0.61,2.27)	1.20 (0.62,2.33)	1.26 (0.77,2.07)	1.14 (0.13,9.59)	1.30 (0.78,2.18)	1.42 (0.13,15.64)	1.38 (0.10,18.57)	1.51 (0.37,6.24)	1.63 (0.06,40.91)	1.60 (0.31,8.40)	1.60 (0.55,4.66)	1.53 (0.86,2.74)	1.53 (0.81,2.92)	1.68 (0.78,3.61)	*1.60 (1.03,2.48)	1.68 (0.99,2.83)	1.80 (0.80,4.08)	2.24 (0.79,6.36)	2.33 (0.85,6.38)	2.76 (0.86,8.89)
1.07 (0.40,2.91)	0.52 (0.02,13.75)	1.06 (0.37,3.06)	Tirzepatide	1.20 (0.38,3.80)	0.98 (0.09,10.09)	1.25 (0.43,3.68)	1.28 (0.43,3.77)	1.34 (0.50,3.60)	1.20 (0.12,12.01)	1.38 (0.51,3.75)	1.51 (0.12,19.26)	1.47 (0.10,22.30)	1.60 (0.31,8.41)	1.73 (0.06,48.56)	1.70 (0.26,10.98)	1.70 (0.43,6.68)	1.63 (0.58,4.58)	1.63 (0.56,4.76)	1.78 (0.57,5.63)	1.69 (0.65,4.44)	1.78 (0.65,4.86)	1.91 (0.59,6.19)	2.38 (0.65,8.64)	2.47 (0.66,9.29)	2.93 (0.69,12.40)
0.90 (0.45,1.80)	0.44 (0.02,10.58)	0.88 (0.41,1.91)	0.83 (0.26,2.64)	Alogliptin	0.82 (0.09,7.53)	1.04 (0.47,2.32)	1.06 (0.48,2.38)	1.11 (0.57,2.19)	1.00 (0.11,8.90)	1.15 (0.58,2.29)	1.26 (0.11,14.45)	1.22 (0.09,17.11)	1.34 (0.30,5.93)	1.44 (0.06,37.38)	1.42 (0.25,7.90)	1.41 (0.44,4.52)	1.36 (0.65,2.84)	1.36 (0.62,2.99)	1.49 (0.61,3.62)	1.41 (0.75,2.66)	1.48 (0.74,2.97)	1.59 (0.62,4.10)	1.98 (0.63,6.21)	2.06 (0.68,6.23)	2.45 (0.70,8.57)
1.10 (0.13,9.42)	0.54 (0.01,23.04)	1.09 (0.12,9.54)	1.02 (0.10,10.54)	1.23 (0.13,11.32)	Oral_semaglutide	1.28 (0.14,11.35)	1.31 (0.15,11.55)	1.37 (0.16,11.65)	1.23 (0.06,24.33)	1.41 (0.16,12.06)	1.54 (0.06,36.99)	1.50 (0.05,41.21)	1.64 (0.13,20.40)	1.76 (0.04,82.12)	1.74 (0.12,24.92)	1.74 (0.17,18.07)	1.66 (0.19,14.43)	1.66 (0.19,14.75)	1.82 (0.20,16.80)	1.73 (0.21,14.58)	1.82 (0.21,15.61)	1.96 (0.24,16.20)	2.43 (0.25,23.78)	2.53 (0.25,25.62)	3.00 (0.28,32.52)
0.86 (0.49,1.51)	0.42 (0.02,9.33)	0.85 (0.44,1.63)	0.80 (0.27,2.35)	0.96 (0.43,2.13)	0.78 (0.09,6.93)	Ertugliflozin	1.02 (0.51,2.04)	1.07 (0.62,1.83)	0.96 (0.11,8.22)	1.10 (0.63,1.92)	1.20 (0.11,13.39)	1.17 (0.09,15.89)	1.28 (0.31,5.38)	1.38 (0.05,34.94)	1.36 (0.26,7.22)	1.36 (0.46,4.03)	1.30 (0.70,2.41)	1.30 (0.66,2.56)	1.43 (0.64,3.15)	1.35 (0.83,2.21)	1.42 (0.81,2.50)	1.53 (0.66,3.54)	1.90 (0.65,5.51)	1.98 (0.71,5.54)	2.34 (0.72,7.68)
0.84 (0.47,1.49)	0.41 (0.02,9.67)	0.83 (0.43,1.61)	0.78 (0.27,2.31)	0.94 (0.42,2.10)	0.77 (0.09,6.77)	0.98 (0.49,1.96)	Empagliflozin	1.05 (0.61,1.81)	0.94 (0.11,8.06)	1.08 (0.61,1.90)	1.18 (0.11,13.14)	1.15 (0.08,15.56)	1.25 (0.30,5.28)	1.35 (0.05,34.27)	1.33 (0.25,7.09)	1.33 (0.45,3.97)	1.27 (0.68,2.38)	1.28 (0.64,2.52)	1.40 (0.63,3.10)	1.33 (0.81,2.18)	1.39 (0.79,2.47)	1.50 (0.66,3.41)	1.86 (0.64,5.37)	1.94 (0.69,5.44)	2.30 (0.70,7.54)
0.80 (0.56,1.16)	0.39 (0.02,8.99)	0.79 (0.48,1.30)	0.75 (0.28,2.01)	0.90 (0.46,1.76)	0.73 (0.09,6.24)	0.94 (0.55,1.60)	0.96 (0.55,1.65)	Dapagliflozin	0.90 (0.11,7.36)	1.03 (0.72,1.47)	1.13 (0.11,11.91)	1.10 (0.08,14.36)	1.20 (0.31,4.69)	1.29 (0.05,31.77)	1.27 (0.25,6.39)	1.27 (0.47,3.46)	1.22 (0.78,1.90)	1.22 (0.72,2.05)	1.33 (0.69,2.60)	*1.27 (1.01,1.59)	1.33 (0.92,1.92)	1.43 (0.68,2.99)	1.78 (0.67,4.73)	1.85 (0.73,4.71)	2.19 (0.73,6.63)
0.89 (0.11,7.34)	0.43 (0.01,18.63)	0.88 (0.10,7.44)	0.83 (0.08,8.27)	1.00 (0.11,8.82)	0.81 (0.04,16.03)	1.04 (0.12,8.86)	1.06 (0.12,9.06)	1.11 (0.14,9.06)	Teneligliptin	1.14 (0.14,9.39)	1.25 (0.05,29.18)	1.22 (0.04,33.15)	1.33 (0.11,15.97)	1.43 (0.03,65.11)	1.41 (0.10,19.55)	1.41 (0.14,14.11)	1.35 (0.16,11.27)	1.35 (0.16,11.49)	1.48 (0.17,13.09)	1.41 (0.17,11.34)	1.48 (0.18,12.15)	1.59 (0.18,14.36)	1.97 (0.20,19.55)	2.05 (0.21,19.99)	2.44 (0.23,25.57)
0.78 (0.53,1.15)	0.38 (0.02,8.74)	0.77 (0.46,1.29)	0.73 (0.27,1.97)	0.87 (0.44,1.73)	0.71 (0.08,6.07)	0.91 (0.52,1.58)	0.93 (0.53,1.63)	0.97 (0.68,1.38)	0.87 (0.11,7.17)	Liraglutide	1.09 (0.10,11.74)	1.06 (0.08,13.97)	1.16 (0.29,4.60)	1.25 (0.05,30.91)	1.23 (0.24,6.23)	1.23 (0.45,3.38)	1.18 (0.74,1.88)	1.18 (0.69,2.03)	1.29 (0.65,2.56)	1.23 (0.94,1.61)	1.29 (0.87,1.91)	1.39 (0.66,2.92)	1.72 (0.64,4.62)	1.79 (0.70,4.62)	2.13 (0.70,6.49)
0.71 (0.07,7.69)	0.35 (0.01,17.45)	0.70 (0.06,7.77)	0.66 (0.05,8.50)	0.80 (0.07,9.17)	0.65 (0.03,15.61)	0.83 (0.07,9.25)	0.85 (0.08,9.46)	0.89 (0.08,9.39)	0.80 (0.03,18.69)	0.92 (0.09,9.85)	Dapagliflozin_ Saxagliptin	0.97 (0.03,31.70)	1.06 (0.10,11.26)	1.15 (0.02,60.86)	1.13 (0.07,19.51)	1.13 (0.09,14.48)	1.08 (0.10,11.79)	1.08 (0.10,11.99)	1.18 (0.10,13.61)	1.13 (0.11,11.91)	1.18 (0.11,12.73)	1.27 (0.11,14.89)	1.58 (0.12,20.09)	1.64 (0.13,20.58)	1.95 (0.15,26.13)
0.73 (0.06,9.65)	0.36 (0.01,20.19)	0.72 (0.05,9.75)	0.68 (0.04,10.39)	0.82 (0.06,11.47)	0.67 (0.02,18.37)	0.85 (0.06,11.59)	0.87 (0.06,11.83)	0.91 (0.07,11.96)	0.82 (0.03,22.42)	0.94 (0.07,12.38)	1.03 (0.03,33.48)	Retatrutide	1.09 (0.06,19.80)	1.18 (0.02,70.79)	1.16 (0.06,23.77)	1.16 (0.07,17.97)	1.11 (0.08,14.79)	1.11 (0.08,15.05)	1.22 (0.09,17.03)	1.16 (0.09,15.00)	1.21 (0.09,15.99)	1.31 (0.10,17.88)	1.62 (0.13,21.03)	1.69 (0.11,25.58)	2.00 (0.13,31.68)
0.67 (0.17,2.67)	0.33 (0.01,9.83)	0.66 (0.16,2.74)	0.62 (0.12,3.28)	0.75 (0.17,3.32)	0.61 (0.05,7.60)	0.78 (0.19,3.28)	0.80 (0.19,3.36)	0.83 (0.21,3.27)	0.75 (0.06,9.03)	0.86 (0.22,3.41)	0.94 (0.09,9.94)	0.91 (0.05,16.55)	Saxagliptin	1.08 (0.03,34.56)	1.06 (0.13,8.59)	1.06 (0.20,5.60)	1.01 (0.25,4.12)	1.02 (0.24,4.24)	1.11 (0.25,4.93)	1.06 (0.27,4.08)	1.11 (0.28,4.41)	1.19 (0.26,5.46)	1.48 (0.28,7.73)	1.54 (0.30,7.84)	1.83 (0.32,10.32)
0.62 (0.03,15.40)	0.30 (0.00,26.48)	0.61 (0.02,15.47)	0.58 (0.02,16.30)	0.70 (0.03,18.05)	0.57 (0.01,26.37)	0.72 (0.03,18.37)	0.74 (0.03,18.77)	0.77 (0.03,19.07)	0.70 (0.02,31.73)	0.80 (0.03,19.73)	0.87 (0.02,46.32)	0.85 (0.01,51.01)	0.93 (0.03,29.78)	Sotagliflozin	0.98 (0.03,35.05)	0.98 (0.03,27.76)	0.94 (0.04,23.52)	0.94 (0.04,23.84)	1.03 (0.04,26.81)	0.98 (0.04,23.96)	1.03 (0.04,25.49)	1.11 (0.04,29.18)	1.38 (0.05,38.59)	1.43 (0.05,39.69)	1.70 (0.06,49.60)
0.63 (0.12,3.21)	0.31 (0.01,10.30)	0.62 (0.12,3.28)	0.59 (0.09,3.80)	0.71 (0.13,3.94)	0.58 (0.04,8.26)	0.74 (0.14,3.91)	0.75 (0.14,4.00)	0.79 (0.16,3.95)	0.71 (0.05,9.83)	0.81 (0.16,4.10)	0.89 (0.05,15.32)	0.86 (0.04,17.67)	0.94 (0.12,7.63)	1.02 (0.03,36.16)	Vildagliptin	1.00 (0.15,6.49)	0.96 (0.18,4.95)	0.96 (0.18,5.07)	1.05 (0.19,5.84)	1.00 (0.20,4.93)	1.05 (0.21,5.31)	1.13 (0.20,6.44)	1.40 (0.22,8.98)	1.46 (0.23,9.14)	1.73 (0.25,11.89)
0.63 (0.23,1.75)	0.31 (0.01,8.14)	0.63 (0.21,1.82)	0.59 (0.15,2.32)	0.71 (0.22,2.26)	0.58 (0.06,6.00)	0.74 (0.25,2.19)	0.75 (0.25,2.25)	0.79 (0.29,2.14)	0.71 (0.07,7.11)	0.81 (0.30,2.23)	0.89 (0.07,11.40)	0.86 (0.06,13.39)	0.94 (0.18,4.99)	1.02 (0.04,28.71)	1.00 (0.15,6.51)	Bexagliflozin	0.96 (0.34,2.73)	0.96 (0.33,2.83)	1.05 (0.33,3.35)	1.00 (0.38,2.65)	1.05 (0.38,2.90)	1.13 (0.34,3.74)	1.40 (0.36,5.46)	1.46 (0.38,5.52)	1.73 (0.40,7.42)
0.66 (0.41,1.07)	0.32 (0.01,7.49)	0.65 (0.36,1.17)	0.62 (0.22,1.73)	0.74 (0.35,1.55)	0.60 (0.07,5.22)	0.77 (0.41,1.43)	0.79 (0.42,1.47)	0.82 (0.53,1.29)	0.74 (0.09,6.19)	0.85 (0.53,1.35)	0.93 (0.08,10.12)	0.90 (0.07,12.01)	0.99 (0.24,4.01)	1.06 (0.04,26.52)	1.05 (0.20,5.41)	1.04 (0.37,2.98)	Albiglutide	1.00 (0.55,1.84)	1.10 (0.53,2.29)	1.04 (0.71,1.53)	1.09 (0.68,1.76)	1.18 (0.54,2.57)	1.46 (0.53,4.05)	1.52 (0.57,4.07)	1.80 (0.57,5.69)
0.66 (0.38,1.15)	0.32 (0.01,7.58)	0.65 (0.34,1.24)	0.61 (0.21,1.79)	0.74 (0.33,1.62)	0.60 (0.07,5.32)	0.77 (0.39,1.51)	0.78 (0.40,1.55)	0.82 (0.49,1.38)	0.74 (0.09,6.29)	0.85 (0.49,1.46)	0.92 (0.08,10.25)	0.90 (0.07,12.18)	0.98 (0.24,4.11)	1.06 (0.04,26.78)	1.04 (0.20,5.52)	1.04 (0.35,3.07)	1.00 (0.54,1.83)	Linagliptin	1.10 (0.50,2.40)	1.04 (0.65,1.66)	1.09 (0.63,1.89)	1.17 (0.51,2.73)	1.46 (0.50,4.21)	1.52 (0.55,4.22)	1.80 (0.55,5.86)
0.60 (0.30,1.20)	0.29 (0.01,7.11)	0.59 (0.28,1.28)	0.56 (0.18,1.77)	0.67 (0.28,1.64)	0.55 (0.06,5.05)	0.70 (0.32,1.55)	0.72 (0.32,1.59)	0.75 (0.38,1.46)	0.68 (0.08,5.97)	0.77 (0.39,1.53)	0.84 (0.07,9.70)	0.82 (0.06,11.49)	0.90 (0.20,3.97)	0.97 (0.04,25.10)	0.95 (0.17,5.30)	0.95 (0.30,3.03)	0.91 (0.44,1.90)	0.91 (0.42,2.00)	Lixisenatide	0.95 (0.51,1.78)	1.00 (0.50,1.98)	1.07 (0.42,2.74)	1.33 (0.43,4.16)	1.39 (0.46,4.17)	1.64 (0.47,5.74)
*0.63 (0.48,0.85)	0.31 (0.01,7.03)	*0.63 (0.40,0.97)	0.59 (0.23,1.55)	0.71 (0.38,1.33)	0.58 (0.07,4.86)	0.74 (0.45,1.20)	0.75 (0.46,1.24)	*0.79 (0.63,0.99)	0.71 (0.09,5.74)	0.81 (0.62,1.07)	0.89 (0.08,9.41)	0.86 (0.07,11.22)	0.95 (0.25,3.64)	1.02 (0.04,24.87)	1.00 (0.20,4.96)	1.00 (0.38,2.66)	0.96 (0.65,1.41)	0.96 (0.60,1.54)	1.05 (0.56,1.97)	Placebo_or_ Control	1.05 (0.79,1.40)	1.13 (0.56,2.27)	1.40 (0.54,3.63)	1.46 (0.59,3.62)	1.73 (0.59,5.11)
*0.60 (0.40,0.90)	0.29 (0.01,6.78)	0.60 (0.35,1.01)	0.56 (0.21,1.53)	0.67 (0.34,1.35)	0.55 (0.06,4.71)	0.70 (0.40,1.24)	0.72 (0.41,1.27)	0.75 (0.52,1.08)	0.68 (0.08,5.57)	0.77 (0.52,1.15)	0.85 (0.08,9.11)	0.82 (0.06,10.83)	0.90 (0.23,3.57)	0.97 (0.04,23.98)	0.95 (0.19,4.84)	0.95 (0.35,2.63)	0.91 (0.57,1.47)	0.92 (0.53,1.58)	1.00 (0.50,1.99)	0.95 (0.72,1.27)	Exenatide	1.07 (0.51,2.28)	1.33 (0.50,3.58)	1.39 (0.54,3.60)	1.65 (0.54,5.04)
0.56 (0.27,1.19)	0.27 (0.01,6.46)	0.55 (0.24,1.26)	0.52 (0.16,1.69)	0.63 (0.24,1.61)	0.51 (0.06,4.24)	0.65 (0.28,1.52)	0.67 (0.29,1.52)	0.70 (0.33,1.46)	0.63 (0.07,5.70)	0.72 (0.34,1.52)	0.79 (0.07,9.23)	0.77 (0.06,10.49)	0.84 (0.18,3.83)	0.90 (0.03,23.77)	0.89 (0.16,5.09)	0.89 (0.27,2.95)	0.85 (0.39,1.86)	0.85 (0.37,1.98)	0.93 (0.36,2.39)	0.89 (0.44,1.78)	0.93 (0.44,1.98)	Sitagliptin	1.24 (0.46,3.34)	1.29 (0.41,4.07)	1.53 (0.43,5.45)
0.45 (0.17,1.21)	0.22 (0.01,5.68)	0.45 (0.16,1.27)	0.42 (0.12,1.53)	0.50 (0.16,1.58)	0.41 (0.04,4.03)	0.53 (0.18,1.53)	0.54 (0.19,1.55)	0.56 (0.21,1.50)	0.51 (0.05,5.03)	0.58 (0.22,1.56)	0.63 (0.05,8.07)	0.62 (0.05,8.00)	0.67 (0.13,3.51)	0.73 (0.03,20.37)	0.72 (0.11,4.59)	0.71 (0.18,2.79)	0.68 (0.25,1.90)	0.69 (0.24,1.98)	0.75 (0.24,2.35)	0.71 (0.28,1.85)	0.75 (0.28,2.01)	0.81 (0.30,2.16)	Dulaglutide	1.04 (0.28,3.88)	1.24 (0.32,4.81)
0.43 (0.17,1.13)	0.21 (0.01,5.48)	0.43 (0.16,1.18)	0.40 (0.11,1.52)	0.49 (0.16,1.47)	0.40 (0.04,4.01)	0.51 (0.18,1.42)	0.52 (0.18,1.45)	0.54 (0.21,1.38)	0.49 (0.05,4.74)	0.56 (0.22,1.44)	0.61 (0.05,7.63)	0.59 (0.04,8.98)	0.65 (0.13,3.29)	0.70 (0.03,19.33)	0.69 (0.11,4.31)	0.69 (0.18,2.60)	0.66 (0.25,1.76)	0.66 (0.24,1.83)	0.72 (0.24,2.17)	0.69 (0.28,1.70)	0.72 (0.28,1.86)	0.77 (0.25,2.43)	0.96 (0.26,3.57)	Omarigliptin	1.19 (0.29,4.87)
0.37 (0.12,1.12)	0.18 (0.01,4.86)	0.36 (0.11,1.16)	0.34 (0.08,1.44)	0.41 (0.12,1.43)	0.33 (0.03,3.61)	0.43 (0.13,1.40)	0.44 (0.13,1.43)	0.46 (0.15,1.38)	0.41 (0.04,4.31)	0.47 (0.15,1.43)	0.51 (0.04,6.88)	0.50 (0.03,7.90)	0.55 (0.10,3.08)	0.59 (0.02,17.16)	0.58 (0.08,3.99)	0.58 (0.13,2.48)	0.55 (0.18,1.75)	0.56 (0.17,1.81)	0.61 (0.17,2.12)	0.58 (0.20,1.70)	0.61 (0.20,1.86)	0.65 (0.18,2.32)	0.81 (0.21,3.15)	0.84 (0.21,3.46)	Efpeglenatide

Data presents RR [95%CIs]. Network meta-analysis results are presented as estimate effect sizes for the outcome of overall asthma-COPD overlap syndrome risk. Interventions are reported in order of mean ranking of beneficially prophylactic effect on overall asthma-COPD overlap syndrome risk, and outcomes are expressed as risk ratio (RR) (95% confidence intervals) (95%CIs). For the upper-right portion, RR of less than 1 indicates that the treatment specified in the row got more beneficial effect than that specified in the column. For the lower-left portion, RR of less than 1 indicates that the treatment specified in the column has more beneficial effect than that specified in the row. Bold results marked with * indicate statistical significance.

eTable 5D League table of primary outcome: overall asthma-COPD overlap syndrome risk focusing on participants with age at least 65 years old

Tirzepatide	1.85 (0.07,48.50)	1.94 (0.08,48.98)	2.00 (0.08,50.55)	2.02 (0.08,50.92)	2.00 (0.08,51.08)	1.46 (0.02,100.93)	2.18 (0.08,56.27)	2.38 (0.09,61.29)	2.98 (0.12,73.10)	8.74 (0.09,805.32)	3.65 (0.15,91.21)	4.28 (0.17,108.98)
0.54 (0.02,14.12)	Sotagliflozin	1.05 (0.49,2.24)	1.08 (0.50,2.32)	1.09 (0.50,2.36)	1.08 (0.48,2.44)	0.79 (0.05,13.56)	1.18 (0.50,2.78)	1.29 (0.56,2.95)	1.61 (0.85,3.04)	4.71 (0.18,122.73)	1.97 (0.96,4.04)	***2.31 (1.05,5.08)**
0.52 (0.02,13.00)	0.95 (0.45,2.05)	Dulaglutide	1.03 (0.58,1.85)	1.04 (0.59,1.85)	1.03 (0.54,1.97)	0.75 (0.05,12.41)	1.12 (0.55,2.27)	1.23 (0.62,2.43)	***1.53 (1.01,2.32)**	4.50 (0.18,112.98)	***1.88 (1.11,3.18)**	***2.21 (1.18,4.14)**
0.50 (0.02,12.58)	0.92 (0.43,1.98)	0.97 (0.54,1.74)	Empagliflozin	1.01 (0.57,1.78)	1.00 (0.52,1.91)	0.73 (0.04,12.01)	1.09 (0.54,2.19)	1.19 (0.60,2.35)	1.48 (0.98,2.24)	4.36 (0.17,109.37)	***1.82 (1.08,3.07)**	***2.14 (1.14,4.01)**
0.49 (0.02,12.43)	0.92 (0.42,1.98)	0.96 (0.54,1.70)	0.99 (0.56,1.74)	Dapagliflozin	0.99 (0.53,1.86)	0.72 (0.04,11.87)	1.08 (0.54,2.14)	1.18 (0.60,2.32)	1.47 (0.99,2.18)	4.32 (0.17,108.07)	***1.81 (1.09,2.99)**	***2.12 (1.13,3.97)**
0.50 (0.02,12.77)	0.93 (0.41,2.10)	0.97 (0.51,1.86)	1.00 (0.52,1.91)	1.01 (0.54,1.90)	Sitagliptin	0.73 (0.04,12.22)	1.09 (0.51,2.32)	1.19 (0.57,2.49)	1.49 (0.90,2.45)	4.37 (0.17,111.00)	***1.83 (1.01,3.31)**	***2.14 (1.07,4.27)**
0.68 (0.01,47.09)	1.27 (0.07,21.73)	1.33 (0.08,21.82)	1.37 (0.08,22.52)	1.38 (0.08,22.67)	1.37 (0.08,22.79)	Ertugliflozin	1.49 (0.09,25.16)	1.63 (0.10,27.38)	2.03 (0.13,32.43)	5.97 (0.09,409.78)	2.50 (0.15,40.56)	2.93 (0.18,48.61)
0.46 (0.02,11.86)	0.85 (0.36,2.01)	0.89 (0.44,1.80)	0.92 (0.46,1.86)	0.93 (0.47,1.85)	0.92 (0.43,1.96)	0.67 (0.04,11.37)	Inject_semaglutide	1.09 (0.50,2.41)	1.37 (0.77,2.42)	4.01 (0.16,103.11)	1.68 (0.87,3.23)	1.97 (0.94,4.14)
0.42 (0.02,10.78)	0.78 (0.34,1.78)	0.81 (0.41,1.61)	0.84 (0.43,1.66)	0.85 (0.43,1.67)	0.84 (0.40,1.75)	0.61 (0.04,10.32)	0.91 (0.42,2.01)	Linagliptin	1.25 (0.73,2.14)	3.66 (0.14,93.68)	1.53 (0.82,2.87)	1.80 (0.88,3.67)
0.34 (0.01,8.26)	0.62 (0.33,1.18)	***0.65 (0.43,0.99)**	0.67 (0.45,1.02)	0.68 (0.46,1.01)	0.67 (0.41,1.11)	0.49 (0.03,7.85)	0.73 (0.41,1.29)	0.80 (0.47,1.38)	Placebo_or_ Control	2.94 (0.12,71.75)	1.23 (0.89,1.69)	1.44 (0.90,2.31)
0.11 (0.00,10.55)	0.21 (0.01,5.52)	0.22 (0.01,5.58)	0.23 (0.01,5.76)	0.23 (0.01,5.80)	0.23 (0.01,5.82)	0.17 (0.00,11.51)	0.25 (0.01,6.41)	0.27 (0.01,6.98)	0.34 (0.01,8.32)	Liraglutide	0.42 (0.02,10.38)	0.49 (0.02,12.41)
0.27 (0.01,6.83)	0.51 (0.25,1.04)	***0.53 (0.31,0.90)**	***0.55 (0.33,0.92)**	***0.55 (0.33,0.92)**	***0.55 (0.30,0.99)**	0.40 (0.02,6.51)	0.60 (0.31,1.15)	0.65 (0.35,1.22)	0.81 (0.59,1.12)	2.39 (0.10,59.39)	Saxagliptin	1.17 (0.66,2.08)
0.23 (0.01,5.94)	***0.43 (0.20,0.95)**	***0.45 (0.24,0.85)**	***0.47 (0.25,0.88)**	***0.47 (0.25,0.88)**	***0.47 (0.23,0.93)**	0.34 (0.02,5.67)	0.51 (0.24,1.07)	0.56 (0.27,1.14)	0.69 (0.43,1.11)	2.04 (0.08,51.59)	0.85 (0.48,1.51)	Oral_semaglutide

Data presents RR [95%CIs]. Network meta-analysis results are presented as estimate effect sizes for the outcome of overall asthma-COPD overlap syndrome risk. Interventions are reported in order of mean ranking of beneficially prophylactic effect on overall asthma-COPD overlap syndrome risk, and outcomes are expressed as risk ratio (RR) (95% confidence intervals) (95%CIs). For the upper-right portion, RR of less than 1 indicates that the treatment specified in the row got more beneficial effect than that specified in the column. For the lower-left portion, RR of less than 1 indicates that the treatment specified in the column has more beneficial effect than that specified in the row. Bold results marked with * indicate statistical significance.

eTable 5E League table of primary outcome: overall asthma-COPD overlap syndrome risk focusing on trials with male predominance

Ertugliflozin_ Sitagliptin	2.33 (0.10,53.58)	2.27 (0.10,53.07)	2.26 (0.09,54.37)	2.36 (0.10,54.56)	2.12 (0.08,59.05)	2.39 (0.10,58.00)	2.51 (0.11,57.74)	2.59 (0.12,57.48)	2.73 (0.12,62.15)	2.56 (0.06,109.20)	2.87 (0.12,65.96)	2.92 (0.13,66.88)	3.18 (0.14,73.41)	3.29 (0.14,76.31)	3.60 (0.14,94.68)	3.78 (0.16,91.27)	3.59 (0.16,81.45)	3.76 (0.16,86.28)	5.66 (0.21,153.55)	4.38 (0.19,100.59)	5.67 (0.22,147.81)	4.91 (0.21,114.72)
0.43 (0.02,9.84)	Inject_semaglutide	0.97 (0.57,1.65)	0.97 (0.50,1.90)	1.01 (0.63,1.61)	0.91 (0.28,2.98)	1.02 (0.50,2.10)	1.08 (0.71,1.64)	1.11 (0.63,1.96)	1.17 (0.83,1.65)	1.10 (0.13,9.01)	1.23 (0.74,2.05)	1.25 (0.84,1.86)	1.36 (0.87,2.14)	1.41 (0.87,2.29)	1.54 (0.56,4.26)	1.62 (0.81,3.23)	***1.54 (1.16,2.05)**	***1.61 (1.08,2.41)**	2.42 (0.80,7.38)	***1.88 (1.26,2.80)**	2.43 (0.90,6.53)	***2.10 (1.24,3.56)**
0.44 (0.02,10.32)	1.03 (0.61,1.75)	Canagliflozin	1.00 (0.47,2.12)	1.04 (0.58,1.85)	0.93 (0.27,3.22)	1.05 (0.47,2.34)	1.11 (0.64,1.91)	1.14 (0.59,2.22)	1.20 (0.74,1.96)	1.13 (0.13,9.53)	1.27 (0.68,2.34)	1.29 (0.76,2.17)	1.40 (0.79,2.47)	1.45 (0.80,2.62)	1.59 (0.54,4.64)	1.67 (0.77,3.60)	***1.58 (1.01,2.48)**	1.66 (0.97,2.82)	2.49 (0.78,8.00)	***1.93 (1.14,3.27)**	2.50 (0.88,7.12)	***2.16 (1.15,4.06)**
0.44 (0.02,10.62)	1.03 (0.53,2.02)	1.00 (0.47,2.13)	Sotagliflozin	1.04 (0.51,2.12)	0.94 (0.25,3.45)	1.06 (0.43,2.59)	1.11 (0.56,2.19)	1.14 (0.52,2.50)	1.20 (0.64,2.28)	1.13 (0.13,9.93)	1.27 (0.61,2.66)	1.29 (0.66,2.51)	1.40 (0.70,2.82)	1.45 (0.71,2.99)	1.59 (0.50,5.01)	1.67 (0.70,4.00)	1.59 (0.87,2.91)	1.66 (0.85,3.24)	2.50 (0.73,8.60)	1.94 (1.00,3.77)	2.51 (0.81,7.70)	***2.17 (1.03,4.59)**
0.42 (0.02,9.83)	0.99 (0.62,1.58)	0.96 (0.54,1.72)	0.96 (0.47,1.95)	Dulaglutide	0.90 (0.27,2.98)	1.01 (0.48,2.16)	1.07 (0.66,1.72)	1.10 (0.59,2.03)	1.16 (0.76,1.75)	1.08 (0.13,9.04)	1.22 (0.70,2.14)	1.24 (0.78,1.96)	1.35 (0.81,2.24)	1.40 (0.82,2.39)	1.53 (0.54,4.33)	1.61 (0.78,3.32)	***1.53 (1.06,2.20)**	1.60 (1.00,2.54)	2.40 (0.78,7.38)	***1.86 (1.18,2.95)**	2.41 (0.87,6.64)	***2.08 (1.17,3.70)**
0.47 (0.02,13.16)	1.10 (0.34,3.62)	1.07 (0.31,3.69)	1.07 (0.29,3.94)	1.11 (0.34,3.68)	Tirzepatide	1.13 (0.30,4.26)	1.18 (0.36,3.92)	1.22 (0.35,4.29)	1.29 (0.40,4.15)	1.21 (0.11,13.11)	1.36 (0.40,4.64)	1.38 (0.42,4.51)	1.50 (0.45,5.01)	1.55 (0.46,5.25)	1.70 (0.37,7.70)	1.79 (0.48,6.64)	1.70 (0.53,5.38)	1.77 (0.54,5.83)	2.67 (0.55,12.93)	2.07 (0.63,6.78)	2.68 (0.60,11.90)	2.32 (0.67,7.98)
0.42 (0.02,10.17)	0.98 (0.48,2.01)	0.95 (0.43,2.11)	0.95 (0.39,2.32)	0.99 (0.46,2.10)	0.89 (0.23,3.35)	Alogliptin	1.05 (0.51,2.18)	1.08 (0.48,2.47)	1.14 (0.57,2.27)	1.07 (0.12,9.56)	1.20 (0.55,2.63)	1.22 (0.60,2.49)	1.33 (0.63,2.80)	1.38 (0.64,2.96)	1.51 (0.46,4.89)	1.58 (0.64,3.93)	1.50 (0.78,2.91)	1.57 (0.77,3.23)	2.37 (0.67,8.37)	1.84 (0.90,3.75)	2.37 (0.75,7.52)	2.06 (0.93,4.54)
0.40 (0.02,9.17)	0.93 (0.61,1.42)	0.90 (0.52,1.56)	0.90 (0.46,1.78)	0.94 (0.58,1.52)	0.84 (0.26,2.79)	0.95 (0.46,1.97)	Empagliflozin	1.03 (0.58,1.84)	1.09 (0.75,1.56)	1.02 (0.12,8.40)	1.14 (0.68,1.92)	1.16 (0.77,1.75)	1.27 (0.80,2.01)	1.31 (0.80,2.15)	1.43 (0.52,3.99)	1.51 (0.75,3.03)	***1.43 (1.05,1.95)**	1.50 (0.98,2.28)	2.25 (0.74,6.91)	***1.75 (1.16,2.64)**	2.26 (0.84,6.11)	***1.96 (1.14,3.35)**
0.39 (0.02,8.59)	0.90 (0.51,1.60)	0.88 (0.45,1.71)	0.87 (0.40,1.91)	0.91 (0.49,1.69)	0.82 (0.23,2.88)	0.92 (0.41,2.10)	0.97 (0.54,1.74)	Ertugliflozin	1.05 (0.62,1.79)	0.99 (0.12,8.44)	1.11 (0.58,2.11)	1.13 (0.64,1.98)	1.23 (0.67,2.25)	1.27 (0.68,2.38)	1.39 (0.47,4.15)	1.46 (0.66,3.25)	1.39 (0.85,2.28)	1.45 (0.82,2.57)	2.19 (0.67,7.15)	1.69 (0.96,2.98)	2.19 (0.75,6.37)	1.90 (0.98,3.68)
0.37 (0.02,8.37)	0.86 (0.61,1.21)	0.83 (0.51,1.35)	0.83 (0.44,1.57)	0.86 (0.57,1.31)	0.78 (0.24,2.51)	0.88 (0.44,1.74)	0.92 (0.64,1.33)	0.95 (0.56,1.61)	Dapagliflozin	0.94 (0.12,7.63)	1.05 (0.66,1.68)	1.07 (0.77,1.49)	1.17 (0.78,1.73)	1.21 (0.78,1.86)	1.32 (0.49,3.57)	1.39 (0.72,2.67)	***1.32 (1.09,1.60)**	1.38 (0.98,1.94)	2.08 (0.70,6.19)	***1.61 (1.15,2.25)**	2.08 (0.79,5.46)	***1.80 (1.11,2.91)**
0.39 (0.01,16.72)	0.91 (0.11,7.51)	0.89 (0.10,7.51)	0.89 (0.10,7.79)	0.92 (0.11,7.68)	0.83 (0.08,9.01)	0.93 (0.10,8.35)	0.98 (0.12,8.10)	1.01 (0.12,8.65)	1.07 (0.13,8.68)	Teneligliptin	1.12 (0.13,9.46)	1.14 (0.14,9.37)	1.24 (0.15,10.32)	1.29 (0.15,10.76)	1.41 (0.14,14.11)	1.48 (0.17,13.09)	1.41 (0.17,11.34)	1.47 (0.18,12.09)	2.21 (0.21,23.18)	1.72 (0.21,14.09)	2.22 (0.22,21.95)	1.92 (0.23,16.22)
0.35 (0.02,8.00)	0.81 (0.49,1.35)	0.79 (0.43,1.46)	0.79 (0.38,1.65)	0.82 (0.47,1.44)	0.74 (0.22,2.52)	0.83 (0.38,1.82)	0.87 (0.52,1.46)	0.90 (0.47,1.71)	0.95 (0.60,1.51)	0.89 (0.11,7.49)	Sitagliptin	1.02 (0.62,1.67)	1.11 (0.64,1.91)	1.14 (0.65,2.03)	1.25 (0.43,3.63)	1.32 (0.62,2.80)	1.25 (0.82,1.91)	1.31 (0.79,2.18)	1.97 (0.62,6.26)	1.53 (0.92,2.53)	1.97 (0.70,5.56)	1.71 (0.94,3.12)
0.34 (0.01,7.86)	0.80 (0.54,1.19)	0.78 (0.46,1.31)	0.78 (0.40,1.51)	0.81 (0.51,1.28)	0.73 (0.22,2.38)	0.82 (0.40,1.67)	0.86 (0.57,1.30)	0.89 (0.50,1.56)	0.93 (0.67,1.31)	0.88 (0.11,7.20)	0.98 (0.60,1.62)	Liraglutide	1.09 (0.70,1.69)	1.13 (0.70,1.81)	1.23 (0.45,3.40)	1.30 (0.66,2.57)	1.23 (0.94,1.62)	1.29 (0.87,1.91)	1.94 (0.64,5.89)	***1.50 (1.02,2.21)**	1.94 (0.73,5.20)	1.68 (1.00,2.83)
0.31 (0.01,7.27)	0.73 (0.47,1.15)	0.71 (0.41,1.26)	0.71 (0.35,1.43)	0.74 (0.45,1.23)	0.67 (0.20,2.23)	0.75 (0.36,1.58)	0.79 (0.50,1.26)	0.81 (0.45,1.49)	0.86 (0.58,1.28)	0.80 (0.10,6.68)	0.90 (0.52,1.56)	0.92 (0.59,1.43)	Linagliptin	1.03 (0.61,1.74)	1.13 (0.40,3.19)	1.19 (0.58,2.44)	1.13 (0.80,1.60)	1.18 (0.75,1.85)	1.78 (0.57,5.52)	1.38 (0.89,2.15)	1.78 (0.65,4.89)	1.55 (0.88,2.71)
0.30 (0.01,7.07)	0.71 (0.44,1.15)	0.69 (0.38,1.25)	0.69 (0.33,1.42)	0.72 (0.42,1.23)	0.64 (0.19,2.18)	0.73 (0.34,1.56)	0.76 (0.46,1.26)	0.79 (0.42,1.48)	0.83 (0.54,1.28)	0.78 (0.09,6.50)	0.87 (0.49,1.55)	0.89 (0.55,1.42)	0.97 (0.57,1.63)	Albiglutide	1.10 (0.38,3.13)	1.15 (0.55,2.41)	1.09 (0.74,1.62)	1.14 (0.71,1.85)	1.72 (0.55,5.41)	1.33 (0.83,2.15)	1.72 (0.62,4.80)	1.49 (0.83,2.69)
0.28 (0.01,7.31)	0.65 (0.23,1.79)	0.63 (0.22,1.84)	0.63 (0.20,1.98)	0.65 (0.23,1.85)	0.59 (0.13,2.67)	0.66 (0.20,2.15)	0.70 (0.25,1.94)	0.72 (0.24,2.14)	0.76 (0.28,2.05)	0.71 (0.07,7.11)	0.80 (0.28,2.31)	0.81 (0.29,2.23)	0.88 (0.31,2.48)	0.91 (0.32,2.61)	Bexagliflozin	1.05 (0.33,3.35)	1.00 (0.38,2.65)	1.04 (0.38,2.88)	1.57 (0.37,6.71)	1.22 (0.44,3.35)	1.57 (0.40,6.12)	1.36 (0.47,3.97)
0.26 (0.01,6.38)	0.62 (0.31,1.23)	0.60 (0.28,1.29)	0.60 (0.25,1.43)	0.62 (0.30,1.29)	0.56 (0.15,2.08)	0.63 (0.25,1.57)	0.66 (0.33,1.33)	0.68 (0.31,1.52)	0.72 (0.37,1.39)	0.68 (0.08,5.97)	0.76 (0.36,1.61)	0.77 (0.39,1.53)	0.84 (0.41,1.72)	0.87 (0.42,1.82)	0.95 (0.30,3.03)	Lixisenatide	0.95 (0.51,1.78)	0.99 (0.50,1.98)	1.49 (0.43,5.19)	1.16 (0.58,2.30)	1.50 (0.48,4.66)	1.30 (0.60,2.79)
0.28 (0.01,6.31)	***0.65 (0.49,0.86)**	***0.63 (0.40,0.99)**	0.63 (0.34,1.15)	***0.66 (0.45,0.95)**	0.59 (0.19,1.87)	0.66 (0.34,1.29)	***0.70 (0.51,0.95)**	0.72 (0.44,1.18)	***0.76 (0.63,0.92)**	0.71 (0.09,5.74)	0.80 (0.52,1.22)	0.81 (0.62,1.07)	0.88 (0.62,1.25)	0.91 (0.62,1.35)	1.00 (0.38,2.66)	1.05 (0.56,1.97)	Placebo_or_ Control	1.05 (0.79,1.39)	1.57 (0.54,4.62)	1.22 (0.93,1.61)	1.58 (0.61,4.06)	1.37 (0.88,2.12)
0.27 (0.01,6.11)	***0.62 (0.42,0.93)**	0.60 (0.35,1.03)	0.60 (0.31,1.18)	0.63 (0.39,1.00)	0.56 (0.17,1.85)	0.64 (0.31,1.30)	0.67 (0.44,1.02)	0.69 (0.39,1.22)	0.73 (0.51,1.02)	0.68 (0.08,5.59)	0.76 (0.46,1.27)	0.78 (0.52,1.15)	0.85 (0.54,1.33)	0.87 (0.54,1.42)	0.96 (0.35,2.65)	1.01 (0.51,2.00)	0.96 (0.72,1.27)	Exenatide	1.51 (0.49,4.58)	1.17 (0.79,1.73)	1.51 (0.56,4.05)	1.31 (0.77,2.21)
0.18 (0.01,4.80)	0.41 (0.14,1.26)	0.40 (0.12,1.29)	0.40 (0.12,1.38)	0.42 (0.14,1.28)	0.37 (0.08,1.81)	0.42 (0.12,1.49)	0.44 (0.14,1.36)	0.46 (0.14,1.49)	0.48 (0.16,1.44)	0.45 (0.04,4.73)	0.51 (0.16,1.61)	0.52 (0.17,1.57)	0.56 (0.18,1.74)	0.58 (0.18,1.83)	0.64 (0.15,2.72)	0.67 (0.19,2.32)	0.64 (0.22,1.86)	0.66 (0.22,2.02)	Efpeglenatide	0.78 (0.26,2.35)	1.00 (0.24,4.20)	0.87 (0.27,2.78)
0.23 (0.01,5.23)	***0.53 (0.36,0.79)**	***0.52 (0.31,0.87)**	0.52 (0.27,1.00)	***0.54 (0.34,0.85)**	0.48 (0.15,1.58)	0.54 (0.27,1.11)	***0.57 (0.38,0.87)**	0.59 (0.34,1.04)	***0.62 (0.44,0.87)**	0.58 (0.07,4.79)	0.65 (0.40,1.08)	***0.67 (0.45,0.98)**	0.72 (0.47,1.13)	0.75 (0.46,1.21)	0.82 (0.30,2.26)	0.86 (0.44,1.71)	0.82 (0.62,1.08)	0.86 (0.58,1.27)	1.29 (0.42,3.92)	Saxagliptin	1.29 (0.48,3.46)	1.12 (0.67,1.88)
0.18 (0.01,4.60)	0.41 (0.15,1.11)	0.40 (0.14,1.14)	0.40 (0.13,1.23)	0.42 (0.15,1.15)	0.37 (0.08,1.66)	0.42 (0.13,1.33)	0.44 (0.16,1.20)	0.46 (0.16,1.33)	0.48 (0.18,1.26)	0.45 (0.05,4.46)	0.51 (0.18,1.43)	0.51 (0.19,1.38)	0.56 (0.20,1.53)	0.58 (0.21,1.61)	0.64 (0.16,2.47)	0.67 (0.21,2.07)	0.63 (0.25,1.63)	0.66 (0.25,1.78)	1.00 (0.24,4.18)	0.77 (0.29,2.07)	Omarigliptin	0.87 (0.31,2.46)
0.20 (0.01,4.76)	***0.48 (0.28,0.80)**	***0.46 (0.25,0.87)**	***0.46 (0.22,0.98)**	***0.48 (0.27,0.85)**	0.43 (0.13,1.49)	0.49 (0.22,1.08)	***0.51 (0.30,0.88)**	0.53 (0.27,1.02)	***0.56 (0.34,0.90)**	0.52 (0.06,4.40)	0.58 (0.32,1.07)	0.59 (0.35,1.00)	0.65 (0.37,1.13)	0.67 (0.37,1.21)	0.73 (0.25,2.14)	0.77 (0.36,1.66)	0.73 (0.47,1.14)	0.77 (0.45,1.29)	1.15 (0.36,3.69)	0.89 (0.53,1.50)	1.15 (0.41,3.28)	Oral_semaglutide

Data presents RR [95%CIs]. Network meta-analysis results are presented as estimate effect sizes for the outcome of overall asthma-COPD overlap syndrome risk. Interventions are reported in order of mean ranking of beneficially prophylactic effect on overall asthma-COPD overlap syndrome risk, and outcomes are expressed as risk ratio (RR) (95% confidence intervals) (95%CIs). For the upper-right portion, RR of less than 1 indicates that the treatment specified in the row got more beneficial effect than that specified in the column. For the lower-left portion, RR of less than 1 indicates that the treatment specified in the column has more beneficial effect than that specified in the row. Bold results marked with * indicate statistical significance.

eTable 5F League table of primary outcome: overall asthma-COPD overlap syndrome risk focusing on trials with female predominance

Tirzepatide	1.37 (0.26,7.25)	1.22 (0.08,18.92)	1.41 (0.04,48.85)	1.57 (0.16,15.83)	1.80 (0.07,44.83)	1.86 (0.14,25.06)	1.95 (0.10,39.22)	2.34 (0.25,21.58)	2.33 (0.50,10.93)	2.55 (0.36,18.18)	2.63 (0.12,55.27)	2.60 (0.25,26.68)	3.47 (0.22,53.69)	6.38 (0.52,77.88)	5.55 (0.64,48.05)
0.73 (0.14,3.86)	Inject_semaglutide	0.89 (0.08,10.35)	1.03 (0.04,29.01)	1.15 (0.16,8.29)	1.31 (0.07,26.02)	1.36 (0.13,13.69)	1.42 (0.09,22.36)	1.71 (0.26,11.14)	1.70 (0.64,4.55)	1.86 (0.42,8.30)	1.92 (0.12,31.20)	1.89 (0.30,11.81)	2.53 (0.21,29.82)	4.65 (0.52,41.45)	4.05 (0.70,23.46)
0.82 (0.05,12.80)	1.13 (0.10,13.15)	Canagliflozin	1.16 (0.02,58.37)	1.29 (0.07,22.35)	1.48 (0.04,55.45)	1.53 (0.07,33.65)	1.60 (0.05,49.76)	1.92 (0.12,31.00)	1.92 (0.20,18.65)	2.10 (0.20,22.03)	2.16 (0.07,65.59)	2.13 (0.12,39.05)	2.85 (0.12,70.59)	5.24 (0.27,100.57)	4.57 (0.35,59.67)
0.71 (0.02,24.73)	0.98 (0.03,27.58)	0.87 (0.02,43.70)	Omarigliptin	1.12 (0.03,42.00)	1.28 (0.02,90.75)	1.32 (0.03,60.26)	1.39 (0.02,83.87)	1.66 (0.05,59.16)	1.66 (0.07,40.44)	1.81 (0.06,55.99)	1.87 (0.03,117.24)	1.85 (0.05,73.07)	2.47 (0.05,123.66)	4.54 (0.11,194.01)	3.95 (0.11,136.33)
0.64 (0.06,6.42)	0.87 (0.12,6.32)	0.77 (0.04,13.41)	0.90 (0.02,33.66)	Dapagliflozin	1.15 (0.08,16.26)	1.19 (0.08,17.75)	1.24 (0.08,20.06)	1.49 (0.14,15.55)	1.48 (0.27,8.27)	1.62 (0.19,13.57)	1.67 (0.07,38.76)	1.65 (0.14,20.24)	2.21 (0.13,37.83)	4.06 (0.30,55.65)	3.54 (0.36,35.25)
0.56 (0.02,13.84)	0.76 (0.04,15.08)	0.68 (0.02,25.31)	0.78 (0.01,55.31)	0.87 (0.06,12.37)	Dapagliflozin_ Saxagliptin	1.03 (0.03,34.58)	1.08 (0.08,15.36)	1.30 (0.05,33.17)	1.29 (0.08,21.70)	1.42 (0.06,30.92)	1.46 (0.03,69.03)	1.44 (0.05,41.40)	1.93 (0.05,71.56)	3.54 (0.11,110.73)	3.09 (0.12,76.20)
0.54 (0.04,7.24)	0.74 (0.07,7.43)	0.65 (0.03,14.37)	0.76 (0.02,34.37)	0.84 (0.06,12.63)	0.97 (0.03,32.36)	Ertugliflozin	1.05 (0.04,28.86)	1.26 (0.09,17.46)	1.25 (0.15,10.14)	1.37 (0.12,15.65)	1.41 (0.05,40.69)	1.40 (0.09,22.35)	1.86 (0.09,40.58)	3.43 (0.19,60.83)	2.98 (0.22,39.78)
0.51 (0.03,10.31)	0.70 (0.04,11.04)	0.62 (0.02,19.35)	0.72 (0.01,43.55)	0.80 (0.05,12.99)	0.92 (0.07,13.09)	0.95 (0.03,26.27)	Saxagliptin	1.20 (0.06,24.77)	1.19 (0.09,15.65)	1.31 (0.07,22.82)	1.35 (0.03,53.41)	1.33 (0.06,31.15)	1.78 (0.06,54.68)	3.27 (0.13,83.77)	2.85 (0.14,56.75)
0.43 (0.05,3.95)	0.59 (0.09,3.83)	0.52 (0.03,8.39)	0.60 (0.02,21.39)	0.67 (0.06,7.02)	0.77 (0.03,19.68)	0.80 (0.06,11.07)	0.83 (0.04,17.25)	Vildagliptin	1.00 (0.20,4.93)	1.09 (0.14,8.29)	1.12 (0.05,24.42)	1.11 (0.10,12.54)	1.48 (0.09,23.67)	2.73 (0.22,34.61)	2.38 (0.26,21.68)
0.43 (0.09,2.01)	0.59 (0.22,1.57)	0.52 (0.05,5.08)	0.60 (0.02,14.71)	0.67 (0.12,3.75)	0.77 (0.05,12.95)	0.80 (0.10,6.46)	0.84 (0.06,10.97)	1.00 (0.20,4.96)	Placebo_or_ Control	1.09 (0.31,3.81)	1.13 (0.08,15.66)	1.11 (0.18,6.90)	1.49 (0.16,14.29)	2.74 (0.38,19.72)	2.38 (0.52,10.98)
0.39 (0.06,2.79)	0.54 (0.12,2.39)	0.48 (0.05,5.01)	0.55 (0.02,17.00)	0.62 (0.07,5.14)	0.71 (0.03,15.40)	0.73 (0.06,8.33)	0.76 (0.04,13.34)	0.92 (0.12,6.96)	0.91 (0.26,3.18)	Sitagliptin	1.03 (0.07,15.37)	1.02 (0.12,8.97)	1.36 (0.10,18.00)	2.50 (0.30,21.05)	2.18 (0.63,7.54)
0.38 (0.02,8.01)	0.52 (0.03,8.49)	0.46 (0.02,14.05)	0.53 (0.01,33.55)	0.60 (0.03,13.84)	0.69 (0.01,32.41)	0.71 (0.02,20.41)	0.74 (0.02,29.44)	0.89 (0.04,19.33)	0.89 (0.06,12.32)	0.97 (0.07,14.49)	Retatrutide	0.99 (0.04,24.06)	1.32 (0.04,42.43)	2.43 (0.10,61.80)	2.11 (0.15,29.37)
0.39 (0.04,3.96)	0.53 (0.08,3.29)	0.47 (0.03,8.57)	0.54 (0.01,21.42)	0.60 (0.05,7.40)	0.69 (0.02,19.91)	0.72 (0.04,11.49)	0.75 (0.03,17.59)	0.90 (0.08,10.17)	0.90 (0.15,5.56)	0.98 (0.11,8.66)	1.01 (0.04,24.65)	Liraglutide	1.34 (0.07,24.41)	2.46 (0.17,35.82)	2.14 (0.20,22.57)
0.29 (0.02,4.46)	0.40 (0.03,4.65)	0.35 (0.01,8.67)	0.41 (0.01,20.29)	0.45 (0.03,7.75)	0.52 (0.01,19.27)	0.54 (0.02,11.67)	0.56 (0.02,17.28)	0.67 (0.04,10.74)	0.67 (0.07,6.45)	0.74 (0.06,9.73)	0.76 (0.02,24.33)	0.75 (0.04,13.66)	Alogliptin	1.84 (0.09,37.02)	1.60 (0.10,24.53)
0.16 (0.01,1.92)	0.21 (0.02,1.91)	0.19 (0.01,3.66)	0.22 (0.01,9.42)	0.25 (0.02,3.37)	0.28 (0.01,8.82)	0.29 (0.02,5.18)	0.31 (0.01,7.84)	0.37 (0.03,4.65)	0.37 (0.05,2.63)	0.40 (0.05,3.37)	0.41 (0.02,10.49)	0.41 (0.03,5.94)	0.54 (0.03,10.96)	Albiglutide	0.87 (0.08,9.17)
0.18 (0.02,1.56)	0.25 (0.04,1.43)	0.22 (0.02,2.86)	0.25 (0.01,8.73)	0.28 (0.03,2.82)	0.32 (0.01,8.00)	0.34 (0.03,4.46)	0.35 (0.02,7.00)	0.42 (0.05,3.84)	0.42 (0.09,1.93)	0.46 (0.13,1.59)	0.47 (0.03,6.57)	0.47 (0.04,4.93)	0.62 (0.04,9.57)	1.15 (0.11,12.09)	Dulaglutide

Data presents RR [95%CIs]. Network meta-analysis results are presented as estimate effect sizes for the outcome of overall asthma-COPD overlap syndrome risk. Interventions are reported in order of mean ranking of beneficially prophylactic effect on overall asthma-COPD overlap syndrome risk, and outcomes are expressed as risk ratio (RR) (95% confidence intervals) (95%CIs). For the upper-right portion, RR of less than 1 indicates that the treatment specified in the row got more beneficial effect than that specified in the column. For the lower-left portion, RR of less than 1 indicates that the treatment specified in the column has more beneficial effect than that specified in the row. Bold results marked with * indicate statistical significance.

eTable 5G League table of primary outcome: overall asthma-COPD overlap syndrome risk focusing on trials with treatment duration at least 1 year

Sotagliflozin	0.49 (0.03,8.55)	0.54 (0.02,13.29)	1.29 (0.61,2.74)	1.07 (0.31,3.71)	1.33 (0.58,3.05)	1.40 (0.62,3.16)	1.44 (0.67,3.12)	1.44 (0.66,3.17)	1.43 (0.55,3.70)	1.46 (0.62,3.40)	1.55 (0.75,3.23)	1.62 (0.76,3.44)	1.76 (0.80,3.85)	2.02 (0.19,21.62)	2.02 (0.35,11.59)	2.02 (0.61,6.71)	1.95 (0.88,4.33)	2.13 (0.83,5.44)	2.02 (1.00,4.06)	2.12 (1.00,4.53)	*2.48 (1.17,5.26)	2.95 (0.94,9.26)	*2.76 (1.21,6.33)	*4.50 (1.18,17.21)
2.02 (0.12,34.92)	Teneligliptin	1.10 (0.02,70.82)	2.61 (0.16,41.87)	2.16 (0.11,41.19)	2.68 (0.16,44.02)	2.83 (0.17,46.25)	2.92 (0.18,47.08)	2.92 (0.18,47.32)	2.88 (0.17,49.16)	2.94 (0.18,48.58)	3.14 (0.20,50.14)	3.28 (0.20,52.63)	3.56 (0.22,57.54)	4.08 (0.11,145.28)	4.09 (0.17,99.47)	4.09 (0.22,76.46)	3.94 (0.24,64.00)	4.30 (0.25,72.94)	4.08 (0.26,64.58)	4.29 (0.27,68.99)	5.01 (0.31,80.49)	5.96 (0.33,109.00)	5.58 (0.34,91.61)	9.10 (0.46,180.91)
1.84 (0.08,45.04)	0.91 (0.01,58.74)	Ertugliflozin_ Sitagliptin	2.37 (0.10,54.44)	1.97 (0.07,52.57)	2.44 (0.10,57.10)	2.58 (0.11,59.15)	2.66 (0.12,61.17)	2.66 (0.12,61.41)	2.62 (0.11,63.49)	2.68 (0.12,59.56)	2.86 (0.13,65.24)	2.99 (0.13,68.44)	3.24 (0.14,74.74)	3.72 (0.08,175.63)	3.73 (0.11,124.04)	3.72 (0.14,97.77)	3.58 (0.15,83.07)	3.91 (0.16,94.25)	3.72 (0.16,84.11)	3.91 (0.17,89.70)	4.57 (0.20,104.66)	5.42 (0.21,139.71)	5.09 (0.22,118.79)	8.29 (0.30,229.91)
0.78 (0.36,1.65)	0.38 (0.02,6.16)	0.42 (0.02,9.65)	Inject_semaglutide	0.83 (0.29,2.41)	1.03 (0.61,1.74)	1.09 (0.66,1.79)	1.12 (0.73,1.71)	1.12 (0.71,1.77)	1.11 (0.55,2.23)	1.13 (0.65,1.97)	1.20 (0.85,1.71)	1.26 (0.85,1.86)	1.36 (0.87,2.13)	1.57 (0.16,15.34)	1.57 (0.31,7.95)	1.57 (0.57,4.32)	1.51 (0.94,2.43)	1.65 (0.83,3.27)	***1.56 (1.18,2.07)**	***1.65 (1.10,2.46)**	***1.92 (1.30,2.85)**	2.28 (0.88,5.90)	***2.14 (1.27,3.62)**	***3.49 (1.08,11.33)**
0.94 (0.27,3.25)	0.46 (0.02,8.82)	0.51 (0.02,13.57)	1.21 (0.42,3.50)	Tirzepatide	1.24 (0.40,3.81)	1.31 (0.43,3.97)	1.35 (0.46,3.97)	1.35 (0.46,3.97)	1.33 (0.40,4.49)	1.36 (0.44,4.24)	1.45 (0.51,4.15)	1.52 (0.52,4.40)	1.64 (0.56,4.87)	1.89 (0.16,22.72)	1.89 (0.28,12.66)	1.89 (0.46,7.80)	1.82 (0.61,5.46)	1.99 (0.60,6.63)	1.89 (0.67,5.28)	1.99 (0.68,5.78)	2.32 (0.80,6.73)	2.76 (0.70,10.86)	2.58 (0.84,7.92)	4.21 (0.91,19.58)
0.75 (0.33,1.73)	0.37 (0.02,6.12)	0.41 (0.02,9.57)	0.97 (0.57,1.64)	0.81 (0.26,2.48)	Canagliflozin	1.06 (0.57,1.94)	1.09 (0.63,1.89)	1.09 (0.61,1.94)	1.07 (0.49,2.36)	1.10 (0.57,2.12)	1.17 (0.71,1.92)	1.22 (0.72,2.07)	1.33 (0.75,2.34)	1.52 (0.15,15.32)	1.53 (0.29,8.02)	1.52 (0.52,4.46)	1.47 (0.81,2.65)	1.60 (0.74,3.46)	1.52 (0.97,2.38)	1.60 (0.94,2.73)	***1.87 (1.10,3.16)**	2.22 (0.81,6.11)	***2.08 (1.11,3.92)**	3.39 (0.99,11.59)
0.71 (0.32,1.61)	0.35 (0.02,5.76)	0.39 (0.02,8.89)	0.92 (0.56,1.51)	0.76 (0.25,2.31)	0.95 (0.51,1.74)	Sitagliptin	1.03 (0.61,1.73)	1.03 (0.61,1.75)	1.02 (0.47,2.19)	1.04 (0.55,1.95)	1.11 (0.70,1.76)	1.16 (0.70,1.90)	1.26 (0.73,2.16)	1.44 (0.14,14.41)	1.44 (0.28,7.53)	1.44 (0.50,4.16)	1.39 (0.80,2.43)	1.52 (0.72,3.21)	1.44 (0.95,2.18)	1.52 (0.91,2.51)	***1.77 (1.08,2.91)**	2.10 (0.78,5.70)	***1.97 (1.08,3.59)**	3.21 (0.95,10.83)
0.69 (0.32,1.49)	0.34 (0.02,5.52)	0.38 (0.02,8.65)	0.89 (0.58,1.37)	0.74 (0.25,2.17)	0.92 (0.53,1.59)	0.97 (0.58,1.63)	Empagliflozin	1.00 (0.62,1.62)	0.99 (0.48,2.03)	1.01 (0.57,1.80)	1.08 (0.73,1.58)	1.12 (0.74,1.71)	1.22 (0.76,1.95)	1.40 (0.14,13.77)	1.40 (0.27,7.15)	1.40 (0.50,3.90)	1.35 (0.82,2.22)	1.47 (0.73,2.97)	***1.40 (1.02,1.92)**	1.47 (0.96,2.26)	***1.72 (1.13,2.62)**	2.04 (0.78,5.34)	***1.91 (1.11,3.31)**	3.12 (0.95,10.22)
0.69 (0.32,1.52)	0.34 (0.02,5.55)	0.38 (0.02,8.68)	0.89 (0.57,1.41)	0.74 (0.25,2.17)	0.92 (0.52,1.63)	0.97 (0.57,1.64)	1.00 (0.62,1.62)	Dulaglutide	0.99 (0.47,2.07)	1.01 (0.55,1.84)	1.08 (0.71,1.63)	1.12 (0.71,1.77)	1.22 (0.74,2.01)	1.40 (0.14,13.85)	1.40 (0.27,7.21)	1.40 (0.50,3.96)	1.35 (0.80,2.28)	1.47 (0.71,3.03)	1.40 (0.97,2.00)	1.47 (0.93,2.33)	***1.72 (1.09,2.70)**	2.04 (0.77,5.41)	***1.91 (1.08,3.39)**	3.12 (0.95,10.18)
0.70 (0.27,1.82)	0.35 (0.02,5.92)	0.38 (0.02,9.22)	0.90 (0.45,1.83)	0.75 (0.22,2.53)	0.93 (0.42,2.04)	0.98 (0.46,2.12)	1.01 (0.49,2.08)	1.01 (0.48,2.12)	Alogliptin	1.02 (0.46,2.28)	1.09 (0.55,2.15)	1.14 (0.56,2.30)	1.23 (0.59,2.57)	1.42 (0.13,14.93)	1.42 (0.25,7.96)	1.42 (0.44,4.57)	1.37 (0.64,2.90)	1.49 (0.61,3.66)	1.42 (0.74,2.70)	1.49 (0.73,3.02)	1.74 (0.86,3.51)	2.07 (0.68,6.29)	1.94 (0.88,4.25)	3.16 (0.85,11.75)
0.69 (0.29,1.60)	0.34 (0.02,5.61)	0.37 (0.02,8.29)	0.89 (0.51,1.54)	0.73 (0.24,2.29)	0.91 (0.47,1.76)	0.96 (0.51,1.80)	0.99 (0.56,1.77)	0.99 (0.54,1.81)	0.98 (0.44,2.19)	Ertugliflozin	1.07 (0.63,1.80)	1.11 (0.64,1.94)	1.21 (0.67,2.19)	1.39 (0.14,14.04)	1.39 (0.26,7.37)	1.39 (0.47,4.12)	1.34 (0.72,2.47)	1.46 (0.66,3.21)	1.39 (0.86,2.24)	1.46 (0.83,2.55)	1.70 (0.98,2.96)	2.02 (0.73,5.65)	1.90 (0.99,3.65)	3.09 (0.89,10.69)
0.64 (0.31,1.34)	0.32 (0.02,5.08)	0.35 (0.02,7.97)	0.83 (0.59,1.18)	0.69 (0.24,1.96)	0.85 (0.52,1.40)	0.90 (0.57,1.43)	0.93 (0.63,1.36)	0.93 (0.61,1.41)	0.92 (0.47,1.81)	0.94 (0.55,1.58)	Dapagliflozin	1.04 (0.74,1.47)	1.13 (0.75,1.70)	1.30 (0.14,12.51)	1.30 (0.26,6.52)	1.30 (0.48,3.52)	1.25 (0.81,1.94)	1.37 (0.71,2.64)	1.30 (1.05,1.60)	1.37 (0.96,1.95)	***1.60 (1.13,2.25)**	1.90 (0.75,4.81)	***1.78 (1.09,2.91)**	2.90 (0.91,9.26)
0.62 (0.29,1.31)	0.30 (0.02,4.89)	0.33 (0.01,7.67)	0.80 (0.54,1.17)	0.66 (0.23,1.91)	0.82 (0.48,1.38)	0.86 (0.53,1.42)	0.89 (0.58,1.36)	0.89 (0.57,1.40)	0.88 (0.44,1.77)	0.90 (0.52,1.56)	0.96 (0.68,1.35)	Liraglutide	1.08 (0.70,1.69)	1.25 (0.13,12.19)	1.25 (0.25,6.31)	1.25 (0.45,3.43)	1.20 (0.75,1.93)	1.31 (0.66,2.59)	1.24 (0.95,1.64)	1.31 (0.88,1.95)	***1.53 (1.04,2.26)**	1.82 (0.70,4.68)	***1.70 (1.01,2.87)**	2.78 (0.86,9.00)
0.57 (0.26,1.24)	0.28 (0.02,4.55)	0.31 (0.01,7.13)	0.73 (0.47,1.15)	0.61 (0.21,1.80)	0.75 (0.43,1.33)	0.80 (0.46,1.37)	0.82 (0.51,1.32)	0.82 (0.50,1.36)	0.81 (0.39,1.69)	0.83 (0.46,1.50)	0.88 (0.59,1.33)	0.92 (0.59,1.44)	Linagliptin	1.15 (0.12,11.36)	1.15 (0.22,5.91)	1.15 (0.41,3.24)	1.11 (0.66,1.86)	1.21 (0.59,2.47)	1.15 (0.81,1.63)	1.21 (0.77,1.90)	1.41 (0.90,2.20)	1.68 (0.63,4.43)	1.57 (0.89,2.77)	2.56 (0.77,8.47)
0.49 (0.05,5.30)	0.24 (0.01,8.71)	0.27 (0.01,12.70)	0.64 (0.07,6.25)	0.53 (0.04,6.37)	0.66 (0.07,6.61)	0.69 (0.07,6.93)	0.71 (0.07,7.04)	0.72 (0.07,7.08)	0.71 (0.07,7.44)	0.72 (0.07,7.30)	0.77 (0.08,7.41)	0.80 (0.08,7.86)	0.87 (0.09,8.61)	Dapagliflozin_ Saxagliptin	1.00 (0.06,16.01)	1.00 (0.09,11.78)	0.96 (0.10,9.58)	1.05 (0.10,11.02)	1.00 (0.10,9.62)	1.05 (0.11,10.31)	1.23 (0.13,11.82)	1.46 (0.13,16.72)	1.37 (0.14,13.75)	2.23 (0.18,28.18)
0.49 (0.09,2.83)	0.24 (0.01,5.94)	0.27 (0.01,8.93)	0.64 (0.13,3.23)	0.53 (0.08,3.53)	0.66 (0.12,3.45)	0.69 (0.13,3.61)	0.71 (0.14,3.64)	0.71 (0.14,3.67)	0.70 (0.13,3.95)	0.72 (0.14,3.82)	0.77 (0.15,3.85)	0.80 (0.16,4.06)	0.87 (0.17,4.46)	1.00 (0.06,15.95)	Vildagliptin	1.00 (0.15,6.49)	0.96 (0.19,4.98)	1.05 (0.19,5.84)	1.00 (0.20,4.93)	1.05 (0.21,5.32)	1.23 (0.24,6.20)	1.46 (0.23,9.14)	1.36 (0.26,7.17)	2.22 (0.31,15.87)
0.49 (0.15,1.64)	0.24 (0.01,4.58)	0.27 (0.01,7.06)	0.64 (0.23,1.76)	0.53 (0.13,2.18)	0.66 (0.22,1.92)	0.69 (0.24,2.00)	0.71 (0.26,1.99)	0.71 (0.25,2.02)	0.71 (0.22,2.27)	0.72 (0.24,2.14)	0.77 (0.28,2.08)	0.80 (0.29,2.21)	0.87 (0.31,2.45)	1.00 (0.08,11.76)	1.00 (0.15,6.51)	Bexagliflozin	0.96 (0.34,2.75)	1.05 (0.33,3.35)	1.00 (0.38,2.65)	1.05 (0.38,2.90)	1.23 (0.45,3.38)	1.46 (0.38,5.52)	1.37 (0.47,3.99)	2.23 (0.50,10.01)
0.51 (0.23,1.14)	0.25 (0.02,4.13)	0.28 (0.01,6.47)	0.66 (0.41,1.07)	0.55 (0.18,1.65)	0.68 (0.38,1.23)	0.72 (0.41,1.26)	0.74 (0.45,1.22)	0.74 (0.44,1.26)	0.73 (0.35,1.55)	0.75 (0.40,1.38)	0.80 (0.52,1.24)	0.83 (0.52,1.34)	0.90 (0.54,1.52)	1.04 (0.10,10.32)	1.04 (0.20,5.38)	1.04 (0.36,2.96)	Albiglutide	1.09 (0.52,2.28)	1.04 (0.70,1.52)	1.09 (0.67,1.77)	1.27 (0.79,2.05)	1.51 (0.56,4.05)	1.42 (0.79,2.56)	2.31 (0.69,7.73)
0.47 (0.18,1.20)	0.23 (0.01,3.95)	0.26 (0.01,6.16)	0.61 (0.31,1.21)	0.50 (0.15,1.68)	0.62 (0.29,1.35)	0.66 (0.31,1.40)	0.68 (0.34,1.37)	0.68 (0.33,1.40)	0.67 (0.27,1.65)	0.69 (0.31,1.51)	0.73 (0.38,1.41)	0.76 (0.39,1.51)	0.83 (0.40,1.69)	0.95 (0.09,9.96)	0.95 (0.17,5.30)	0.95 (0.30,3.03)	0.92 (0.44,1.91)	Lixisenatide	0.95 (0.51,1.78)	1.00 (0.50,1.99)	1.17 (0.59,2.31)	1.39 (0.46,4.17)	1.30 (0.60,2.80)	2.12 (0.58,7.80)
0.50 (0.25,1.00)	0.25 (0.02,3.88)	0.27 (0.01,6.09)	***0.64 (0.48,0.85)**	0.53 (0.19,1.48)	0.66 (0.42,1.03)	0.69 (0.46,1.05)	***0.72 (0.52,0.98)**	0.72 (0.50,1.03)	0.71 (0.37,1.35)	0.72 (0.45,1.17)	***0.77 (0.63,0.95)**	0.80 (0.61,1.06)	0.87 (0.61,1.24)	1.00 (0.10,9.64)	1.00 (0.20,4.96)	1.00 (0.38,2.66)	0.96 (0.66,1.42)	1.05 (0.56,1.97)	Placebo_or_ Control	1.05 (0.79,1.40)	1.23 (0.93,1.62)	1.46 (0.59,3.62)	1.37 (0.88,2.14)	2.23 (0.71,7.00)
0.47 (0.22,1.00)	0.23 (0.01,3.74)	0.26 (0.01,5.87)	***0.61 (0.41,0.91)**	0.50 (0.17,1.47)	0.62 (0.37,1.06)	0.66 (0.40,1.09)	0.68 (0.44,1.05)	0.68 (0.43,1.08)	0.67 (0.33,1.36)	0.69 (0.39,1.20)	0.73 (0.51,1.05)	0.76 (0.51,1.14)	0.83 (0.53,1.30)	0.95 (0.10,9.33)	0.95 (0.19,4.83)	0.95 (0.34,2.63)	0.92 (0.57,1.48)	1.00 (0.50,1.99)	0.95 (0.71,1.27)	Exenatide	1.17 (0.78,1.74)	1.39 (0.54,3.59)	1.30 (0.77,2.21)	2.12 (0.65,6.90)
***0.40 (0.19,0.86)**	0.20 (0.01,3.20)	0.22 (0.01,5.02)	***0.52 (0.35,0.77)**	0.43 (0.15,1.25)	***0.53 (0.32,0.91)**	***0.56 (0.34,0.93)**	***0.58 (0.38,0.89)**	***0.58 (0.37,0.92)**	0.57 (0.28,1.16)	0.59 (0.34,1.02)	***0.63 (0.44,0.88)**	***0.65 (0.44,0.97)**	0.71 (0.45,1.11)	0.81 (0.08,7.84)	0.82 (0.16,4.13)	0.81 (0.30,2.24)	0.78 (0.49,1.26)	0.86 (0.43,1.70)	0.81 (0.62,1.07)	0.86 (0.57,1.28)	Saxagliptin	1.19 (0.46,3.06)	1.11 (0.66,1.88)	1.82 (0.56,5.89)
0.34 (0.11,1.07)	0.17 (0.01,3.07)	0.18 (0.01,4.75)	**0.44 (0.17,1.13)**	0.36 (0.09,1.43)	0.45 (0.16,1.24)	0.48 (0.18,1.29)	0.49 (0.19,1.28)	0.49 (0.18,1.30)	0.48 (0.16,1.47)	0.49 (0.18,1.38)	0.53 (0.21,1.34)	0.55 (0.21,1.42)	0.60 (0.23,1.58)	0.69 (0.06,7.86)	0.69 (0.11,4.31)	0.69 (0.18,2.60)	0.66 (0.25,1.77)	0.72 (0.24,2.17)	0.69 (0.28,1.70)	0.72 (0.28,1.87)	0.84 (0.33,2.17)	Omarigliptin	0.94 (0.34,2.57)	1.53 (0.36,6.58)
***0.36 (0.16,0.83)**	0.18 (0.01,2.94)	0.20 (0.01,4.59)	*0.47 (0.28,0.79)	0.39 (0.13,1.19)	***0.48 (0.26,0.90)**	***0.51 (0.28,0.92)**	***0.52 (0.30,0.90)**	***0.52 (0.30,0.93)**	0.52 (0.24,1.13)	0.53 (0.27,1.01)	***0.56 (0.34,0.92)**	***0.59 (0.35,0.99)**	0.64 (0.36,1.12)	0.73 (0.07,7.35)	0.73 (0.14,3.85)	0.73 (0.25,2.14)	0.70 (0.39,1.27)	0.77 (0.36,1.66)	0.73 (0.47,1.14)	0.77 (0.45,1.31)	0.90 (0.53,1.52)	1.07 (0.39,2.93)	Oral_semaglutide	1.63 (0.48,5.56)
***0.22 (0.06,0.85)**	0.11 (0.01,2.18)	0.12 (0.00,3.34)	***0.29 (0.09,0.93)**	0.24 (0.05,1.10)	0.29 (0.09,1.01)	0.31 (0.09,1.05)	0.32 (0.10,1.05)	0.32 (0.10,1.05)	0.32 (0.09,1.18)	0.32 (0.09,1.12)	0.35 (0.11,1.10)	0.36 (0.11,1.17)	0.39 (0.12,1.29)	0.45 (0.04,5.67)	0.45 (0.06,3.21)	0.45 (0.10,2.02)	0.43 (0.13,1.45)	0.47 (0.13,1.74)	0.45 (0.14,1.41)	0.47 (0.14,1.53)	0.55 (0.17,1.79)	0.65 (0.15,2.82)	0.61 (0.18,2.09)	Efpeglenatide

Data presents RR [95%CIs]. Network meta-analysis results are presented as estimate effect sizes for the outcome of overall asthma-COPD overlap syndrome risk. Interventions are reported in order of mean ranking of beneficially prophylactic effect on overall asthma-COPD overlap syndrome risk, and outcomes are expressed as risk ratio (RR) (95% confidence intervals) (95%CIs). For the upper-right portion, RR of less than 1 indicates that the treatment specified in the row got more beneficial effect than that specified in the column. For the lower-left portion, RR of less than 1 indicates that the treatment specified in the column has more beneficial effect than that specified in the row. Bold results marked with * indicate statistical significance.

eTable 5H League table of primary outcome: overall asthma-COPD overlap syndrome risk using hazard ratio based on time-to-event data

Tirzepatide	0.52 (0.02,13.48)	1.20 (0.46,3.12)	1.17 (0.42,3.24)	1.18 (0.39,3.56)	1.31 (0.50,3.46)	1.34 (0.51,3.56)	1.33 (0.43,4.07)	1.36 (0.48,3.84)	1.42 (0.56,3.64)	1.14 (0.08,16.91)	1.46 (0.54,3.95)	1.34 (0.14,13.17)	1.54 (0.59,4.01)	1.85 (0.16,21.46)	1.66 (0.62,4.45)	1.88 (0.30,11.93)	1.88 (0.49,7.22)	1.78 (0.66,4.84)	1.98 (0.65,6.03)	1.88 (0.75,4.72)	1.96 (0.75,5.15)	2.28 (0.87,5.96)	2.74 (0.75,9.99)	2.99 (0.72,12.34)	2.56 (0.92,7.12)
1.92 (0.07,49.78)	Ertugliflozin_ Sitagliptin	2.31 (0.10,53.13)	2.25 (0.10,52.55)	2.27 (0.09,54.60)	2.52 (0.11,58.01)	2.58 (0.11,59.65)	2.55 (0.11,61.78)	2.61 (0.12,58.10)	2.73 (0.12,62.38)	2.19 (0.04,122.32)	2.80 (0.12,64.20)	2.57 (0.06,110.16)	2.95 (0.13,67.79)	3.56 (0.08,168.99)	3.19 (0.14,73.81)	3.62 (0.11,120.81)	3.61 (0.14,95.29)	3.43 (0.15,79.56)	3.80 (0.16,91.75)	3.61 (0.16,81.85)	3.78 (0.16,86.77)	4.38 (0.19,100.66)	5.26 (0.20,135.94)	5.74 (0.21,156.18)	4.92 (0.21,115.20)
0.83 (0.32,2.15)	0.43 (0.02,9.92)	Inject_semaglutide	0.97 (0.58,1.63)	0.98 (0.50,1.91)	1.09 (0.72,1.65)	1.11 (0.71,1.74)	1.10 (0.55,2.21)	1.13 (0.65,1.97)	1.18 (0.84,1.65)	0.95 (0.07,12.14)	1.21 (0.76,1.94)	1.11 (0.13,9.17)	1.28 (0.87,1.87)	1.54 (0.16,15.14)	1.38 (0.88,2.15)	1.56 (0.31,7.93)	1.56 (0.56,4.32)	1.48 (0.92,2.38)	1.64 (0.83,3.26)	***1.56 (1.18,2.05)**	***1.63 (1.10,2.42)**	***1.89 (1.28,2.79)**	2.27 (0.88,5.88)	2.48 (0.82,7.56)	***2.13 (1.26,3.58)**
0.86 (0.31,2.37)	0.45 (0.02,10.41)	1.03 (0.61,1.73)	Canagliflozin	1.01 (0.48,2.14)	1.12 (0.65,1.92)	1.15 (0.65,2.02)	1.14 (0.53,2.46)	1.16 (0.60,2.23)	1.22 (0.75,1.97)	0.98 (0.07,12.80)	1.25 (0.70,2.23)	1.14 (0.13,9.72)	1.31 (0.78,2.21)	1.59 (0.16,16.00)	1.42 (0.81,2.49)	1.61 (0.31,8.47)	1.61 (0.55,4.71)	1.53 (0.85,2.74)	1.69 (0.79,3.64)	***1.61 (1.03,2.49)**	1.68 (0.99,2.84)	***1.95 (1.16,3.28)**	2.34 (0.85,6.43)	2.56 (0.80,8.20)	***2.19 (1.17,4.09)**
0.85 (0.28,2.55)	0.44 (0.02,10.60)	1.02 (0.52,1.99)	0.99 (0.47,2.10)	Sotagliflozin	1.11 (0.56,2.19)	1.14 (0.56,2.30)	1.13 (0.47,2.71)	1.15 (0.53,2.50)	1.20 (0.64,2.28)	0.97 (0.07,13.11)	1.23 (0.60,2.53)	1.13 (0.13,10.01)	1.30 (0.67,2.53)	1.57 (0.15,16.44)	1.41 (0.70,2.83)	1.59 (0.29,8.83)	1.59 (0.50,5.04)	1.51 (0.74,3.10)	1.67 (0.70,4.01)	1.59 (0.87,2.92)	1.66 (0.85,3.26)	1.93 (0.99,3.76)	2.32 (0.78,6.92)	2.53 (0.73,8.73)	***2.17 (1.02,4.60)**
0.76 (0.29,2.01)	0.40 (0.02,9.14)	0.92 (0.61,1.39)	0.89 (0.52,1.53)	0.90 (0.46,1.78)	Empagliflozin	1.02 (0.64,1.64)	1.01 (0.50,2.06)	1.04 (0.58,1.84)	1.09 (0.75,1.56)	0.87 (0.07,11.20)	1.11 (0.68,1.81)	1.02 (0.12,8.47)	1.17 (0.78,1.77)	1.41 (0.14,13.97)	1.27 (0.79,2.02)	1.44 (0.28,7.33)	1.44 (0.51,4.01)	1.36 (0.83,2.23)	1.51 (0.75,3.04)	***1.43 (1.05,1.95)**	1.50 (0.98,2.29)	***1.74 (1.15,2.63)**	2.09 (0.80,5.46)	2.28 (0.74,7.01)	***1.95 (1.14,3.35)**
0.74 (0.28,1.98)	0.39 (0.02,8.97)	0.90 (0.57,1.40)	0.87 (0.50,1.53)	0.88 (0.44,1.78)	0.98 (0.61,1.56)	Dulaglutide	0.99 (0.48,2.05)	1.01 (0.56,1.84)	1.06 (0.71,1.59)	0.85 (0.07,10.74)	1.09 (0.65,1.80)	0.99 (0.12,8.33)	1.15 (0.73,1.79)	1.38 (0.14,13.73)	1.24 (0.75,2.03)	1.40 (0.27,7.23)	1.40 (0.49,3.97)	1.33 (0.79,2.24)	1.47 (0.72,3.03)	1.40 (0.98,1.99)	1.46 (0.93,2.30)	***1.70 (1.09,2.66)**	2.04 (0.77,5.41)	2.23 (0.73,6.84)	***1.91 (1.08,3.36)**
0.75 (0.25,2.30)	0.39 (0.02,9.47)	0.91 (0.45,1.81)	0.88 (0.41,1.90)	0.89 (0.37,2.14)	0.99 (0.49,2.00)	1.01 (0.49,2.09)	Alogliptin	1.02 (0.46,2.27)	1.07 (0.55,2.08)	0.86 (0.06,11.72)	1.10 (0.52,2.30)	1.00 (0.11,8.97)	1.16 (0.58,2.31)	1.39 (0.13,14.72)	1.25 (0.61,2.58)	1.42 (0.25,7.92)	1.41 (0.44,4.55)	1.34 (0.64,2.82)	1.49 (0.61,3.63)	1.41 (0.75,2.67)	1.48 (0.74,2.97)	1.72 (0.86,3.43)	2.06 (0.68,6.24)	2.25 (0.64,7.86)	1.93 (0.89,4.18)
0.74 (0.26,2.08)	0.38 (0.02,8.53)	0.89 (0.51,1.54)	0.86 (0.45,1.65)	0.87 (0.40,1.89)	0.96 (0.54,1.71)	0.99 (0.54,1.80)	0.98 (0.44,2.17)	Ertugliflozin	1.05 (0.62,1.76)	0.84 (0.06,11.10)	1.07 (0.58,1.98)	0.98 (0.11,8.44)	1.13 (0.65,1.97)	1.36 (0.13,13.89)	1.22 (0.67,2.22)	1.39 (0.26,7.38)	1.38 (0.46,4.13)	1.31 (0.71,2.43)	1.46 (0.66,3.21)	1.38 (0.85,2.24)	1.45 (0.83,2.53)	1.68 (0.96,2.93)	2.02 (0.72,5.64)	2.20 (0.68,7.17)	1.89 (0.98,3.63)
0.70 (0.27,1.80)	0.37 (0.02,8.35)	0.85 (0.60,1.18)	0.82 (0.51,1.33)	0.83 (0.44,1.57)	0.92 (0.64,1.33)	0.94 (0.63,1.41)	0.93 (0.48,1.82)	0.95 (0.57,1.61)	Dapagliflozin	0.80 (0.06,10.20)	1.02 (0.67,1.58)	0.94 (0.11,7.70)	1.08 (0.77,1.51)	1.30 (0.13,12.61)	1.17 (0.78,1.74)	1.32 (0.26,6.64)	1.32 (0.49,3.59)	1.25 (0.81,1.93)	1.39 (0.72,2.68)	***1.32 (1.09,1.60)**	1.38 (0.98,1.95)	***1.60 (1.15,2.24)**	1.93 (0.76,4.88)	2.10 (0.70,6.29)	***1.80 (1.11,2.92)**
0.88 (0.06,13.00)	0.46 (0.01,25.47)	1.06 (0.08,13.54)	1.03 (0.08,13.45)	1.04 (0.08,14.06)	1.15 (0.09,14.80)	1.18 (0.09,14.87)	1.17 (0.09,15.93)	1.19 (0.09,15.75)	1.25 (0.10,15.88)	Retatrutide	1.28 (0.10,16.58)	1.17 (0.04,31.42)	1.35 (0.11,17.28)	1.63 (0.05,48.88)	1.46 (0.11,18.84)	1.65 (0.08,33.14)	1.65 (0.11,25.02)	1.56 (0.12,20.34)	1.73 (0.13,23.65)	1.65 (0.13,20.80)	1.72 (0.13,22.12)	2.00 (0.16,25.65)	2.40 (0.16,35.54)	2.62 (0.17,41.15)	2.25 (0.17,29.49)
0.69 (0.25,1.86)	0.36 (0.02,8.19)	0.83 (0.52,1.32)	0.80 (0.45,1.44)	0.81 (0.39,1.67)	0.90 (0.55,1.47)	0.92 (0.56,1.53)	0.91 (0.43,1.92)	0.93 (0.51,1.72)	0.98 (0.63,1.50)	0.78 (0.06,10.17)	Sitagliptin	0.92 (0.11,7.72)	1.06 (0.66,1.69)	1.27 (0.13,12.72)	1.14 (0.68,1.92)	1.29 (0.25,6.71)	1.29 (0.45,3.70)	1.22 (0.72,2.10)	1.36 (0.65,2.84)	1.29 (0.88,1.90)	1.35 (0.83,2.18)	1.57 (0.98,2.52)	1.88 (0.70,5.05)	2.05 (0.65,6.45)	1.76 (0.98,3.14)
0.75 (0.08,7.38)	0.39 (0.01,16.73)	0.90 (0.11,7.46)	0.88 (0.10,7.45)	0.88 (0.10,7.84)	0.98 (0.12,8.16)	1.01 (0.12,8.42)	1.00 (0.11,8.89)	1.02 (0.12,8.73)	1.07 (0.13,8.74)	0.85 (0.03,22.93)	1.09 (0.13,9.18)	Teneligliptin	1.15 (0.14,9.52)	1.39 (0.06,30.48)	1.24 (0.15,10.40)	1.41 (0.10,19.70)	1.41 (0.14,14.24)	1.34 (0.16,11.24)	1.48 (0.17,13.20)	1.41 (0.17,11.43)	1.47 (0.18,12.19)	1.71 (0.21,14.14)	2.05 (0.21,20.14)	2.24 (0.21,23.64)	1.92 (0.23,16.33)
0.65 (0.25,1.70)	0.34 (0.01,7.77)	0.78 (0.53,1.15)	0.76 (0.45,1.28)	0.77 (0.40,1.50)	0.85 (0.56,1.29)	0.87 (0.56,1.36)	0.86 (0.43,1.73)	0.88 (0.51,1.54)	0.93 (0.66,1.29)	0.74 (0.06,9.51)	0.95 (0.59,1.51)	0.87 (0.11,7.19)	Liraglutide	1.21 (0.12,11.86)	1.08 (0.69,1.68)	1.23 (0.24,6.21)	1.22 (0.44,3.38)	1.16 (0.73,1.85)	1.29 (0.65,2.55)	1.22 (0.93,1.60)	1.28 (0.86,1.89)	***1.48 (1.01,2.19)**	1.78 (0.69,4.60)	1.94 (0.64,5.92)	1.67 (0.99,2.80)
0.54 (0.05,6.24)	0.28 (0.01,13.32)	0.65 (0.07,6.39)	0.63 (0.06,6.36)	0.64 (0.06,6.68)	0.71 (0.07,6.99)	0.72 (0.07,7.20)	0.72 (0.07,7.57)	0.73 (0.07,7.46)	0.77 (0.08,7.42)	0.62 (0.02,18.50)	0.79 (0.08,7.85)	0.72 (0.03,15.81)	0.83 (0.08,8.15)	Dapagliflozin_ Saxagliptin	0.90 (0.09,8.90)	1.02 (0.06,16.33)	1.01 (0.09,12.02)	0.96 (0.10,9.61)	1.07 (0.10,11.24)	1.01 (0.10,9.80)	1.06 (0.11,10.44)	1.23 (0.13,11.91)	1.48 (0.13,17.03)	1.61 (0.13,19.90)	1.38 (0.14,13.95)
0.60 (0.22,1.61)	0.31 (0.01,7.25)	0.73 (0.47,1.13)	0.70 (0.40,1.23)	0.71 (0.35,1.43)	0.79 (0.50,1.26)	0.81 (0.49,1.33)	0.80 (0.39,1.65)	0.82 (0.45,1.48)	0.86 (0.57,1.28)	0.69 (0.05,8.89)	0.88 (0.52,1.48)	0.80 (0.10,6.73)	0.93 (0.59,1.44)	1.12 (0.11,11.09)	Linagliptin	1.13 (0.22,5.83)	1.13 (0.40,3.21)	1.07 (0.64,1.80)	1.19 (0.58,2.44)	1.13 (0.80,1.60)	1.18 (0.75,1.86)	1.37 (0.88,2.14)	1.65 (0.62,4.37)	1.80 (0.58,5.59)	1.54 (0.88,2.71)
0.53 (0.08,3.36)	0.28 (0.01,9.22)	0.64 (0.13,3.24)	0.62 (0.12,3.26)	0.63 (0.11,3.47)	0.70 (0.14,3.55)	0.71 (0.14,3.67)	0.71 (0.13,3.95)	0.72 (0.14,3.84)	0.76 (0.15,3.79)	0.61 (0.03,12.15)	0.77 (0.15,4.01)	0.71 (0.05,9.90)	0.82 (0.16,4.14)	0.98 (0.06,15.82)	0.88 (0.17,4.54)	Vildagliptin	1.00 (0.15,6.52)	0.95 (0.18,4.91)	1.05 (0.19,5.86)	1.00 (0.20,4.94)	1.04 (0.21,5.30)	1.21 (0.24,6.14)	1.45 (0.23,9.16)	1.59 (0.23,10.94)	1.36 (0.26,7.16)
0.53 (0.14,2.04)	0.28 (0.01,7.29)	0.64 (0.23,1.77)	0.62 (0.21,1.82)	0.63 (0.20,1.99)	0.70 (0.25,1.95)	0.71 (0.25,2.02)	0.71 (0.22,2.27)	0.72 (0.24,2.15)	0.76 (0.28,2.05)	0.61 (0.04,9.20)	0.77 (0.27,2.22)	0.71 (0.07,7.17)	0.82 (0.30,2.26)	0.99 (0.08,11.67)	0.88 (0.31,2.50)	1.00 (0.15,6.54)	Bexagliflozin	0.95 (0.33,2.72)	1.05 (0.33,3.37)	1.00 (0.37,2.66)	1.04 (0.38,2.90)	1.21 (0.44,3.36)	1.46 (0.38,5.54)	1.59 (0.37,6.83)	1.36 (0.46,3.99)
0.56 (0.21,1.52)	0.29 (0.01,6.78)	0.68 (0.42,1.08)	0.66 (0.37,1.18)	0.66 (0.32,1.36)	0.74 (0.45,1.20)	0.75 (0.45,1.27)	0.75 (0.35,1.57)	0.76 (0.41,1.41)	0.80 (0.52,1.23)	0.64 (0.05,8.32)	0.82 (0.48,1.40)	0.75 (0.09,6.30)	0.86 (0.54,1.38)	1.04 (0.10,10.39)	0.93 (0.55,1.57)	1.06 (0.20,5.48)	1.05 (0.37,3.02)	Albiglutide	1.11 (0.53,2.32)	1.05 (0.72,1.55)	1.10 (0.68,1.78)	1.28 (0.80,2.05)	1.54 (0.57,4.12)	1.68 (0.53,5.27)	1.44 (0.80,2.58)
0.51 (0.17,1.54)	0.26 (0.01,6.36)	0.61 (0.31,1.21)	0.59 (0.27,1.27)	0.60 (0.25,1.43)	0.66 (0.33,1.34)	0.68 (0.33,1.40)	0.67 (0.28,1.64)	0.69 (0.31,1.52)	0.72 (0.37,1.39)	0.58 (0.04,7.87)	0.74 (0.35,1.54)	0.68 (0.08,6.02)	0.78 (0.39,1.54)	0.94 (0.09,9.88)	0.84 (0.41,1.72)	0.95 (0.17,5.31)	0.95 (0.30,3.05)	0.90 (0.43,1.88)	Lixisenatide	0.95 (0.51,1.78)	0.99 (0.50,1.98)	1.15 (0.58,2.29)	1.39 (0.46,4.18)	1.51 (0.43,5.27)	1.30 (0.60,2.79)
0.53 (0.21,1.34)	0.28 (0.01,6.29)	***0.64 (0.49,0.84)**	***0.62 (0.40,0.97)**	0.63 (0.34,1.15)	***0.70 (0.51,0.95)**	0.71 (0.50,1.02)	0.71 (0.38,1.34)	0.72 (0.45,1.17)	***0.76 (0.62,0.92)**	0.61 (0.05,7.67)	0.78 (0.53,1.14)	0.71 (0.09,5.78)	0.82 (0.62,1.07)	0.99 (0.10,9.55)	0.88 (0.62,1.25)	1.00 (0.20,4.97)	1.00 (0.38,2.67)	0.95 (0.65,1.40)	1.05 (0.56,1.97)	Placebo_or_ Control	1.05 (0.79,1.39)	1.22 (0.92,1.60)	1.46 (0.59,3.62)	1.59 (0.54,4.68)	1.36 (0.88,2.12)
0.51 (0.19,1.33)	0.26 (0.01,6.09)	***0.61 (0.41,0.91)**	0.60 (0.35,1.01)	0.60 (0.31,1.18)	0.67 (0.44,1.02)	0.68 (0.43,1.08)	0.68 (0.34,1.36)	0.69 (0.39,1.21)	0.72 (0.51,1.02)	0.58 (0.05,7.45)	0.74 (0.46,1.20)	0.68 (0.08,5.63)	0.78 (0.53,1.16)	0.94 (0.10,9.29)	0.85 (0.54,1.33)	0.96 (0.19,4.87)	0.96 (0.34,2.66)	0.91 (0.56,1.47)	1.01 (0.50,2.01)	0.96 (0.72,1.27)	Exenatide	1.16 (0.78,1.73)	1.39 (0.54,3.62)	1.52 (0.50,4.65)	1.30 (0.77,2.21)
0.44 (0.17,1.15)	0.23 (0.01,5.24)	***0.53 (0.36,0.78)**	***0.51 (0.30,0.86)**	0.52 (0.27,1.01)	***0.57 (0.38,0.87)**	***0.59 (0.38,0.92)**	0.58 (0.29,1.16)	0.60 (0.34,1.04)	***0.62 (0.45,0.87)**	0.50 (0.04,6.41)	0.64 (0.40,1.03)	0.59 (0.07,4.84)	***0.67 (0.46,0.99)**	0.81 (0.08,7.86)	0.73 (0.47,1.13)	0.83 (0.16,4.19)	0.82 (0.30,2.28)	0.78 (0.49,1.25)	0.87 (0.44,1.72)	0.82 (0.63,1.08)	0.86 (0.58,1.28)	Saxagliptin	1.20 (0.46,3.10)	1.31 (0.43,3.99)	1.12 (0.67,1.89)
0.36 (0.10,1.33)	0.19 (0.01,4.91)	0.44 (0.17,1.14)	0.43 (0.16,1.17)	0.43 (0.14,1.29)	0.48 (0.18,1.25)	0.49 (0.18,1.30)	0.49 (0.16,1.47)	0.50 (0.18,1.39)	0.52 (0.21,1.31)	0.42 (0.03,6.16)	0.53 (0.20,1.43)	0.49 (0.05,4.78)	0.56 (0.22,1.45)	0.68 (0.06,7.80)	0.61 (0.23,1.60)	0.69 (0.11,4.33)	0.69 (0.18,2.61)	0.65 (0.24,1.75)	0.72 (0.24,2.18)	0.69 (0.28,1.70)	0.72 (0.28,1.86)	0.83 (0.32,2.15)	Omarigliptin	1.09 (0.27,4.47)	0.94 (0.34,2.57)
0.33 (0.08,1.38)	0.17 (0.01,4.73)	0.40 (0.13,1.23)	0.39 (0.12,1.25)	0.40 (0.11,1.36)	0.44 (0.14,1.35)	0.45 (0.15,1.38)	0.44 (0.13,1.56)	0.45 (0.14,1.48)	0.48 (0.16,1.42)	0.38 (0.02,5.99)	0.49 (0.16,1.53)	0.45 (0.04,4.71)	0.51 (0.17,1.56)	0.62 (0.05,7.65)	0.56 (0.18,1.73)	0.63 (0.09,4.34)	0.63 (0.15,2.70)	0.60 (0.19,1.88)	0.66 (0.19,2.30)	0.63 (0.21,1.85)	0.66 (0.22,2.01)	0.76 (0.25,2.32)	0.92 (0.22,3.76)	Efpeglenatide	0.86 (0.27,2.75)
0.39 (0.14,1.08)	0.20 (0.01,4.75)	***0.47 (0.28,0.79)**	***0.46 (0.24,0.85)**	***0.46 (0.22,0.98)**	***0.51 (0.30,0.88)**	***0.52 (0.30,0.92)**	0.52 (0.24,1.12)	0.53 (0.28,1.02)	***0.56 (0.34,0.90)**	0.45 (0.03,5.84)	0.57 (0.32,1.02)	0.52 (0.06,4.43)	0.60 (0.36,1.01)	0.72 (0.07,7.30)	0.65 (0.37,1.14)	0.73 (0.14,3.87)	0.73 (0.25,2.15)	0.70 (0.39,1.25)	0.77 (0.36,1.66)	0.73 (0.47,1.14)	0.77 (0.45,1.30)	0.89 (0.53,1.50)	1.07 (0.39,2.94)	1.17 (0.36,3.74)	Oral_semaglutide

Data presents RR [95%CIs]. Network meta-analysis results are presented as estimate effect sizes for the outcome of overall asthma-COPD overlap syndrome risk. Interventions are reported in order of mean ranking of beneficially prophylactic effect on overall asthma-COPD overlap syndrome risk, and outcomes are expressed as hazard ratio (HR) (95% confidence intervals) (95%CIs). For the upper-right portion, HR of less than 1 indicates that the treatment specified in the row got more beneficial effect than that specified in the column. For the lower-left portion, HR of less than 1 indicates that the treatment specified in the column has more beneficial effect than that specified in the row. Bold results marked with * indicate statistical significance.

eTable 5I League table of secondary outcome: asthma risk

Dapagliflozin_Saxagliptin	2.83 (0.12,69.05)	2.90 (0.10,86.80)	3.58 (0.13,98.24)	4.04 (0.12,140.62)	4.74 (0.17,128.34)	4.98 (0.13,189.91)	5.12 (0.15,169.07)	5.42 (0.19,155.66)	5.47 (0.19,160.73)	5.96 (0.21,166.39)	6.74 (0.26,173.39)	6.83 (0.25,189.68)	7.09 (0.25,201.18)	7.13 (0.26,197.95)	8.38 (0.22,314.00)	8.76 (0.20,381.09)	9.11 (0.24,351.41)	10.13 (0.11,967.32)	9.96 (0.19,519.61)	9.73 (0.29,329.89)	10.28 (0.37,284.67)	20.27 (0.21,1935.86)	14.07 (0.50,392.57)
0.35 (0.01,8.60)	Dapagliflozin	1.02 (0.32,3.29)	1.26 (0.52,3.05)	1.43 (0.30,6.72)	1.67 (0.73,3.84)	1.76 (0.31,10.11)	1.81 (0.43,7.53)	1.91 (0.68,5.40)	1.93 (0.64,5.86)	2.10 (0.82,5.41)	***2.38 (1.31,4.31)**	2.41 (0.96,6.06)	2.50 (0.92,6.78)	2.52 (1.00,6.32)	2.96 (0.53,16.39)	3.09 (0.41,23.05)	3.21 (0.55,18.94)	3.57 (0.14,92.55)	3.51 (0.34,36.23)	3.43 (0.77,15.22)	***3.63 (1.46,9.04)**	7.15 (0.28,185.23)	***4.96 (1.94,12.72)**
0.35 (0.01,10.34)	0.98 (0.30,3.15)	Ertugliflozin	1.24 (0.37,4.09)	1.40 (0.24,8.02)	1.63 (0.51,5.22)	1.72 (0.25,11.83)	1.77 (0.34,9.12)	1.87 (0.50,6.98)	1.89 (0.48,7.47)	2.06 (0.59,7.14)	2.33 (0.85,6.36)	2.36 (0.69,8.05)	2.45 (0.68,8.85)	2.46 (0.72,8.40)	2.89 (0.44,19.24)	3.03 (0.35,26.40)	3.14 (0.45,22.11)	3.50 (0.12,100.03)	3.44 (0.29,40.64)	3.36 (0.62,18.31)	***3.55 (1.05,12.04)**	7.00 (0.24,200.20)	***4.86 (1.40,16.82)**
0.28 (0.01,7.67)	0.79 (0.33,1.91)	0.81 (0.24,2.68)	Inject_semaglutide	1.13 (0.23,5.44)	1.32 (0.56,3.12)	1.39 (0.24,8.16)	1.43 (0.35,5.81)	1.51 (0.52,4.41)	1.53 (0.50,4.63)	1.66 (0.62,4.44)	1.88 (0.98,3.60)	1.91 (0.73,4.97)	1.98 (0.71,5.56)	1.99 (0.77,5.19)	2.34 (0.41,13.23)	2.45 (0.32,18.56)	2.55 (0.42,15.29)	2.83 (0.11,74.06)	2.78 (0.27,29.11)	2.72 (0.60,12.33)	***2.87 (1.11,7.42)**	5.66 (0.22,148.22)	***3.93 (1.48,10.44)**
0.25 (0.01,8.60)	0.70 (0.15,3.30)	0.72 (0.12,4.12)	0.89 (0.18,4.26)	Lixisenatide	1.17 (0.25,5.49)	1.23 (0.14,10.90)	1.27 (0.18,8.73)	1.34 (0.25,7.08)	1.35 (0.24,7.48)	1.47 (0.30,7.35)	1.67 (0.40,6.97)	1.69 (0.34,8.33)	1.75 (0.34,9.04)	1.76 (0.36,8.69)	2.07 (0.24,17.81)	2.17 (0.20,23.73)	2.25 (0.25,20.33)	2.51 (0.08,83.34)	2.46 (0.17,35.61)	2.41 (0.33,17.39)	2.54 (0.52,12.46)	5.01 (0.15,166.79)	3.48 (0.70,17.33)
0.21 (0.01,5.72)	0.60 (0.26,1.38)	0.61 (0.19,1.95)	0.76 (0.32,1.78)	0.85 (0.18,4.00)	Liraglutide	1.05 (0.18,6.02)	1.08 (0.26,4.47)	1.14 (0.41,3.21)	1.15 (0.38,3.48)	1.26 (0.49,3.21)	1.42 (0.80,2.55)	1.44 (0.59,3.53)	1.50 (0.56,4.03)	1.51 (0.60,3.75)	1.77 (0.32,9.77)	1.85 (0.25,13.74)	1.92 (0.33,11.29)	2.14 (0.08,55.26)	2.10 (0.20,21.61)	2.06 (0.47,9.07)	2.17 (0.88,5.37)	4.28 (0.17,110.61)	***2.97 (1.17,7.55)**
0.20 (0.01,7.65)	0.57 (0.10,3.27)	0.58 (0.08,4.00)	0.72 (0.12,4.21)	0.81 (0.09,7.18)	0.95 (0.17,5.44)	Alogliptin	1.03 (0.13,8.35)	1.09 (0.17,6.94)	1.10 (0.17,7.30)	1.20 (0.20,7.25)	1.35 (0.26,7.01)	1.37 (0.23,8.22)	1.42 (0.23,8.87)	1.43 (0.24,8.57)	1.68 (0.17,16.77)	1.76 (0.14,22.03)	1.83 (0.18,19.08)	2.03 (0.06,74.25)	2.00 (0.12,32.63)	1.95 (0.23,16.58)	2.06 (0.35,12.30)	4.07 (0.11,148.60)	2.82 (0.47,17.08)
0.20 (0.01,6.45)	0.55 (0.13,2.31)	0.57 (0.11,2.92)	0.70 (0.17,2.84)	0.79 (0.11,5.45)	0.93 (0.22,3.83)	0.97 (0.12,7.91)	Tirzepatide	1.06 (0.22,5.00)	1.07 (0.22,5.28)	1.16 (0.26,5.17)	1.32 (0.36,4.82)	1.34 (0.31,5.84)	1.39 (0.30,6.37)	1.39 (0.32,6.10)	1.64 (0.21,12.91)	1.71 (0.17,17.36)	1.78 (0.21,14.76)	1.98 (0.06,62.50)	1.95 (0.14,26.27)	1.90 (0.29,12.51)	2.01 (0.46,8.75)	3.96 (0.13,125.08)	2.75 (0.62,12.18)
0.18 (0.01,5.30)	0.52 (0.19,1.48)	0.53 (0.14,1.99)	0.66 (0.23,1.92)	0.75 (0.14,3.94)	0.87 (0.31,2.45)	0.92 (0.14,5.86)	0.94 (0.20,4.45)	Canagliflozin	1.01 (0.28,3.58)	1.10 (0.36,3.38)	1.24 (0.53,2.91)	1.26 (0.42,3.80)	1.31 (0.41,4.21)	1.32 (0.44,3.94)	1.55 (0.25,9.51)	1.62 (0.20,13.18)	1.68 (0.26,10.96)	1.87 (0.07,51.19)	1.84 (0.16,20.47)	1.80 (0.36,8.94)	1.90 (0.65,5.54)	3.74 (0.14,102.45)	2.60 (0.85,7.95)
0.18 (0.01,5.38)	0.52 (0.17,1.57)	0.53 (0.13,2.10)	0.65 (0.22,1.99)	0.74 (0.13,4.09)	0.87 (0.29,2.61)	0.91 (0.14,6.06)	0.94 (0.19,4.63)	0.99 (0.28,3.52)	Exenatide	1.09 (0.33,3.59)	1.23 (0.48,3.15)	1.25 (0.39,4.04)	1.30 (0.38,4.45)	1.30 (0.42,4.09)	1.53 (0.24,9.80)	1.60 (0.19,13.57)	1.67 (0.25,11.33)	1.85 (0.07,51.97)	1.82 (0.16,20.97)	1.78 (0.34,9.32)	1.88 (0.59,6.00)	3.71 (0.13,104.02)	2.57 (0.78,8.45)
0.17 (0.01,4.69)	0.48 (0.18,1.22)	0.49 (0.14,1.69)	0.60 (0.23,1.60)	0.68 (0.14,3.39)	0.79 (0.31,2.03)	0.84 (0.14,5.07)	0.86 (0.19,3.82)	0.91 (0.30,2.80)	0.92 (0.28,3.02)	Empagliflozin	1.13 (0.54,2.36)	1.15 (0.41,3.17)	1.19 (0.40,3.53)	1.20 (0.44,3.30)	1.41 (0.24,8.22)	1.47 (0.19,11.48)	1.53 (0.25,9.49)	1.70 (0.06,45.31)	1.67 (0.16,17.93)	1.63 (0.35,7.68)	1.73 (0.64,4.64)	3.40 (0.13,90.68)	2.36 (0.84,6.65)
0.15 (0.01,3.82)	***0.42 (0.23,0.76)**	0.43 (0.16,1.18)	0.53 (0.28,1.02)	0.60 (0.14,2.51)	0.70 (0.39,1.26)	0.74 (0.14,3.83)	0.76 (0.21,2.78)	0.80 (0.34,1.88)	0.81 (0.32,2.08)	0.88 (0.42,1.84)	Placebo_or_Control	1.01 (0.50,2.05)	1.05 (0.47,2.34)	1.06 (0.52,2.14)	1.24 (0.25,6.20)	1.30 (0.19,8.86)	1.35 (0.25,7.19)	1.50 (0.06,36.86)	1.48 (0.15,14.11)	1.44 (0.37,5.66)	1.53 (0.76,3.05)	3.01 (0.12,73.77)	***2.09 (1.01,4.33)**
0.15 (0.01,4.06)	0.41 (0.16,1.04)	0.42 (0.12,1.45)	0.52 (0.20,1.36)	0.59 (0.12,2.91)	0.69 (0.28,1.70)	0.73 (0.12,4.36)	0.75 (0.17,3.28)	0.79 (0.26,2.39)	0.80 (0.25,2.59)	0.87 (0.32,2.41)	0.99 (0.49,1.99)	Albiglutide	1.04 (0.36,3.01)	1.04 (0.39,2.82)	1.23 (0.21,7.08)	1.28 (0.17,9.90)	1.33 (0.22,8.17)	1.48 (0.06,39.23)	1.46 (0.14,15.49)	1.42 (0.31,6.62)	1.50 (0.56,4.04)	2.97 (0.11,78.51)	2.06 (0.75,5.67)
0.14 (0.00,4.00)	0.40 (0.15,1.08)	0.41 (0.11,1.48)	0.50 (0.18,1.42)	0.57 (0.11,2.94)	0.67 (0.25,1.80)	0.70 (0.11,4.38)	0.72 (0.16,3.31)	0.76 (0.24,2.46)	0.77 (0.22,2.65)	0.84 (0.28,2.49)	0.95 (0.43,2.12)	0.96 (0.33,2.80)	Linagliptin	1.01 (0.35,2.92)	1.18 (0.20,7.11)	1.24 (0.15,9.88)	1.28 (0.20,8.19)	1.43 (0.05,38.64)	1.40 (0.13,15.38)	1.37 (0.28,6.68)	1.45 (0.50,4.18)	2.86 (0.11,77.34)	1.98 (0.67,5.86)
0.14 (0.01,3.89)	0.40 (0.16,1.00)	0.41 (0.12,1.39)	0.50 (0.19,1.31)	0.57 (0.12,2.79)	0.66 (0.27,1.65)	0.70 (0.12,4.18)	0.72 (0.16,3.14)	0.76 (0.25,2.28)	0.77 (0.24,2.41)	0.84 (0.30,2.30)	0.94 (0.47,1.91)	0.96 (0.35,2.59)	0.99 (0.34,2.89)	Dulaglutide	1.18 (0.22,6.31)	1.23 (0.16,9.49)	1.28 (0.21,7.83)	1.42 (0.05,37.58)	1.40 (0.13,14.84)	1.36 (0.30,6.25)	1.44 (0.61,3.41)	2.84 (0.11,75.22)	1.97 (0.72,5.43)
0.12 (0.00,4.47)	0.34 (0.06,1.87)	0.35 (0.05,2.30)	0.43 (0.08,2.41)	0.48 (0.06,4.14)	0.56 (0.10,3.12)	0.59 (0.06,5.92)	0.61 (0.08,4.81)	0.65 (0.11,3.97)	0.65 (0.10,4.17)	0.71 (0.12,4.15)	0.80 (0.16,4.00)	0.82 (0.14,4.71)	0.85 (0.14,5.09)	0.85 (0.16,4.57)	Efpeglenatide	1.05 (0.09,12.76)	1.09 (0.11,11.03)	1.21 (0.03,43.32)	1.19 (0.07,18.94)	1.16 (0.14,9.53)	1.23 (0.22,6.93)	2.42 (0.07,86.71)	1.68 (0.29,9.79)
0.11 (0.00,4.96)	0.32 (0.04,2.41)	0.33 (0.04,2.88)	0.41 (0.05,3.10)	0.46 (0.04,5.05)	0.54 (0.07,4.01)	0.57 (0.05,7.12)	0.58 (0.06,5.92)	0.62 (0.08,5.04)	0.62 (0.07,5.28)	0.68 (0.09,5.30)	0.77 (0.11,5.24)	0.78 (0.10,6.02)	0.81 (0.10,6.47)	0.81 (0.11,6.28)	0.96 (0.08,11.67)	Omarigliptin	1.04 (0.08,13.24)	1.16 (0.03,48.19)	1.14 (0.06,21.96)	1.11 (0.11,11.70)	1.17 (0.15,9.02)	2.31 (0.06,96.45)	1.61 (0.21,12.50)
0.11 (0.00,4.23)	0.31 (0.05,1.83)	0.32 (0.05,2.24)	0.39 (0.07,2.36)	0.44 (0.05,4.00)	0.52 (0.09,3.05)	0.55 (0.05,5.70)	0.56 (0.07,4.66)	0.60 (0.09,3.88)	0.60 (0.09,4.08)	0.65 (0.11,4.06)	0.74 (0.14,3.93)	0.75 (0.12,4.60)	0.78 (0.12,4.97)	0.78 (0.13,4.80)	0.92 (0.09,9.34)	0.96 (0.08,12.25)	Sotagliflozin	1.11 (0.03,41.08)	1.09 (0.07,18.11)	1.07 (0.12,9.24)	1.13 (0.18,6.89)	2.23 (0.06,82.21)	1.54 (0.25,9.56)
0.10 (0.00,9.42)	0.28 (0.01,7.24)	0.29 (0.01,8.18)	0.35 (0.01,9.24)	0.40 (0.01,13.27)	0.47 (0.02,12.07)	0.49 (0.01,17.94)	0.51 (0.02,15.95)	0.54 (0.02,14.65)	0.54 (0.02,15.14)	0.59 (0.02,15.66)	0.66 (0.03,16.30)	0.67 (0.03,17.85)	0.70 (0.03,18.94)	0.70 (0.03,18.62)	0.83 (0.02,29.66)	0.87 (0.02,36.06)	0.90 (0.02,33.21)	Bexagliflozin	0.98 (0.02,49.27)	0.96 (0.03,31.12)	1.01 (0.04,26.78)	2.00 (0.02,184.54)	1.39 (0.05,36.94)
0.10 (0.00,5.24)	0.28 (0.03,2.93)	0.29 (0.02,3.44)	0.36 (0.03,3.76)	0.41 (0.03,5.87)	0.48 (0.05,4.89)	0.50 (0.03,8.16)	0.51 (0.04,6.94)	0.54 (0.05,6.07)	0.55 (0.05,6.32)	0.60 (0.06,6.41)	0.68 (0.07,6.46)	0.69 (0.06,7.29)	0.71 (0.06,7.80)	0.72 (0.07,7.61)	0.84 (0.05,13.42)	0.88 (0.05,17.01)	0.91 (0.06,15.16)	1.02 (0.02,51.00)	Teneligliptin	0.98 (0.07,13.65)	1.03 (0.10,10.93)	2.04 (0.04,102.06)	1.41 (0.13,15.13)
0.10 (0.00,3.48)	0.29 (0.07,1.29)	0.30 (0.05,1.62)	0.37 (0.08,1.67)	0.42 (0.06,3.00)	0.49 (0.11,2.15)	0.51 (0.06,4.34)	0.53 (0.08,3.46)	0.56 (0.11,2.77)	0.56 (0.11,2.94)	0.61 (0.13,2.88)	0.69 (0.18,2.71)	0.70 (0.15,3.26)	0.73 (0.15,3.55)	0.73 (0.16,3.36)	0.86 (0.10,7.07)	0.90 (0.09,9.49)	0.94 (0.11,8.10)	1.04 (0.03,33.73)	1.02 (0.07,14.30)	Oral_semaglutide	1.06 (0.24,4.61)	2.08 (0.06,67.51)	1.45 (0.31,6.80)
0.10 (0.00,2.69)	***0.28 (0.11,0.69)**	***0.28 (0.08,0.96)**	***0.35 (0.13,0.90)**	0.39 (0.08,1.93)	0.46 (0.19,1.14)	0.48 (0.08,2.89)	0.50 (0.11,2.17)	0.53 (0.18,1.54)	0.53 (0.17,1.70)	0.58 (0.22,1.56)	0.66 (0.33,1.31)	0.66 (0.25,1.79)	0.69 (0.24,1.99)	0.69 (0.29,1.64)	0.82 (0.14,4.61)	0.85 (0.11,6.56)	0.89 (0.15,5.41)	0.99 (0.04,26.01)	0.97 (0.09,10.26)	0.95 (0.22,4.13)	Sitagliptin	1.97 (0.07,52.07)	1.37 (0.50,3.74)
0.05 (0.00,4.71)	0.14 (0.01,3.62)	0.14 (0.00,4.09)	0.18 (0.01,4.62)	0.20 (0.01,6.64)	0.23 (0.01,6.04)	0.25 (0.01,8.97)	0.25 (0.01,7.97)	0.27 (0.01,7.33)	0.27 (0.01,7.57)	0.29 (0.01,7.83)	0.33 (0.01,8.15)	0.34 (0.01,8.92)	0.35 (0.01,9.47)	0.35 (0.01,9.31)	0.41 (0.01,14.83)	0.43 (0.01,18.03)	0.45 (0.01,16.60)	0.50 (0.01,46.10)	0.49 (0.01,24.63)	0.48 (0.01,15.56)	0.51 (0.02,13.39)	Vildagliptin	0.69 (0.03,18.47)
0.07 (0.00,1.98)	***0.20 (0.08,0.52)**	***0.21 (0.06,0.71)**	***0.25 (0.10,0.68)**	0.29 (0.06,1.43)	***0.34 (0.13,0.86)**	0.35 (0.06,2.14)	0.36 (0.08,1.61)	0.39 (0.13,1.18)	0.39 (0.12,1.28)	0.42 (0.15,1.19)	***0.48 (0.23,0.99)**	0.49 (0.18,1.34)	0.50 (0.17,1.49)	0.51 (0.18,1.40)	0.60 (0.10,3.48)	0.62 (0.08,4.85)	0.65 (0.10,4.01)	0.72 (0.03,19.16)	0.71 (0.07,7.58)	0.69 (0.15,3.25)	0.73 (0.27,2.00)	1.44 (0.05,38.35)	Saxagliptin

Data presents RR [95%CIs]. Network meta-analysis results are presented as estimate effect sizes for the outcome of asthma risk. Interventions are reported in order of mean ranking of beneficially prophylactic effect on asthma risk, and outcomes are expressed as risk ratio (RR) (95% confidence intervals) (95%CIs). For the upper-right portion, RR of less than 1 indicates that the treatment specified in the row got more beneficial effect than that specified in the column. For the lower-left portion, RR of less than 1 indicates that the treatment specified in the column has more beneficial effect than that specified in the row. Bold results marked with * indicate statistical significance.

eTable 5J League table of secondary outcome: COPD risk

Teneligliptin	4.60 (0.15,143.04)	6.94 (0.27,176.18)	6.86 (0.26,178.25)	3.53 (0.04,307.68)	7.18 (0.28,181.12)	8.04 (0.32,199.37)	7.99 (0.32,199.59)	8.11 (0.33,200.38)	8.49 (0.32,221.69)	7.19 (0.12,421.47)	8.11 (0.21,314.81)	9.93 (0.41,243.19)	10.32 (0.42,254.94)	10.23 (0.40,261.27)	11.14 (0.39,315.10)	10.52 (0.42,261.88)	11.19 (0.44,281.37)	12.13 (0.50,295.38)	12.99 (0.53,320.69)	14.74 (0.56,387.53)	13.40 (0.54,330.82)	16.63 (0.56,497.82)	14.46 (0.57,365.44)	40.21 (0.44,3711.01)	18.27 (0.64,523.21)
0.22 (0.01,6.77)	Tirzepatide	1.51 (0.38,5.98)	1.49 (0.36,6.24)	0.77 (0.03,22.47)	1.56 (0.40,6.10)	1.75 (0.47,6.55)	1.74 (0.46,6.50)	1.76 (0.48,6.47)	1.85 (0.44,7.81)	1.56 (0.09,26.38)	1.76 (0.20,15.85)	2.16 (0.59,7.86)	2.25 (0.61,8.33)	2.22 (0.55,8.95)	2.42 (0.48,12.18)	2.29 (0.60,8.66)	2.43 (0.63,9.43)	2.64 (0.74,9.45)	2.83 (0.76,10.47)	3.21 (0.75,13.77)	2.92 (0.79,10.80)	3.62 (0.64,20.34)	3.14 (0.80,12.34)	8.75 (0.28,275.83)	3.97 (0.77,20.49)
0.14 (0.01,3.66)	0.66 (0.17,2.63)	Canagliflozin	0.99 (0.43,2.27)	0.51 (0.02,12.10)	1.03 (0.51,2.10)	1.16 (0.62,2.16)	1.15 (0.59,2.23)	1.17 (0.64,2.13)	1.22 (0.52,2.87)	1.04 (0.08,13.67)	1.17 (0.18,7.52)	1.43 (0.82,2.50)	1.49 (0.81,2.72)	1.47 (0.69,3.16)	1.61 (0.53,4.91)	1.52 (0.80,2.89)	1.61 (0.81,3.22)	*1.75 (1.04,2.94)	*1.87 (1.03,3.41)	2.12 (0.88,5.10)	*1.93 (1.06,3.52)	2.40 (0.67,8.60)	*2.08 (1.02,4.26)	5.79 (0.22,149.27)	2.63 (0.83,8.36)
0.15 (0.01,3.79)	0.67 (0.16,2.81)	1.01 (0.44,2.33)	Sotagliflozin	0.51 (0.02,12.54)	1.05 (0.47,2.35)	1.17 (0.56,2.45)	1.16 (0.54,2.51)	1.18 (0.58,2.42)	1.24 (0.48,3.16)	1.05 (0.08,14.24)	1.18 (0.18,7.92)	1.45 (0.73,2.86)	1.51 (0.73,3.09)	1.49 (0.63,3.51)	1.62 (0.50,5.31)	1.53 (0.72,3.26)	1.63 (0.74,3.61)	1.77 (0.92,3.39)	1.89 (0.93,3.88)	2.15 (0.82,5.61)	1.95 (0.96,4.00)	2.43 (0.64,9.23)	2.11 (0.93,4.77)	5.86 (0.22,154.63)	2.66 (0.79,9.02)
0.28 (0.00,24.76)	1.30 (0.04,38.22)	1.97 (0.08,46.89)	1.95 (0.08,47.47)	Ertugliflozin_ Sitagliptin	2.04 (0.09,47.24)	2.28 (0.10,53.02)	2.27 (0.10,53.11)	2.30 (0.10,53.30)	2.41 (0.10,59.04)	2.04 (0.04,113.68)	2.30 (0.06,84.42)	2.82 (0.12,64.68)	2.93 (0.13,67.80)	2.90 (0.13,64.54)	3.16 (0.12,84.05)	2.98 (0.13,69.68)	3.17 (0.13,74.82)	3.44 (0.15,78.55)	3.69 (0.16,85.31)	4.18 (0.17,103.22)	3.80 (0.16,88.00)	4.72 (0.17,132.92)	4.10 (0.17,97.21)	11.41 (0.13,1006.06)	5.18 (0.19,139.59)
0.14 (0.01,3.52)	0.64 (0.16,2.51)	0.97 (0.48,1.97)	0.96 (0.43,2.15)	0.49 (0.02,11.40)	Sitagliptin	1.12 (0.62,2.01)	1.11 (0.59,2.10)	1.13 (0.64,1.99)	1.18 (0.52,2.71)	1.00 (0.08,13.12)	1.13 (0.18,7.20)	1.38 (0.82,2.34)	1.44 (0.82,2.53)	1.43 (0.69,2.95)	1.55 (0.52,4.67)	1.47 (0.79,2.71)	1.56 (0.81,2.99)	*1.69 (1.04,2.74)	*1.81 (1.03,3.20)	2.05 (0.87,4.83)	*1.87 (1.06,3.30)	2.32 (0.66,8.20)	*2.02 (1.02,3.98)	5.60 (0.22,143.56)	2.55 (0.82,7.95)
0.12 (0.01,3.08)	0.57 (0.15,2.14)	0.86 (0.46,1.61)	0.85 (0.41,1.78)	0.44 (0.02,10.19)	0.89 (0.50,1.60)	Empagliflozin	0.99 (0.58,1.69)	1.01 (0.64,1.59)	1.06 (0.50,2.25)	0.89 (0.07,11.45)	1.01 (0.16,6.23)	1.23 (0.83,1.84)	1.28 (0.81,2.03)	1.27 (0.66,2.45)	1.39 (0.49,3.95)	1.31 (0.78,2.18)	1.39 (0.79,2.46)	*1.51 (1.07,2.13)	*1.62 (1.03,2.55)	1.83 (0.84,4.02)	*1.67 (1.06,2.62)	2.07 (0.61,6.98)	1.80 (0.99,3.27)	5.00 (0.20,125.81)	2.27 (0.77,6.74)
0.13 (0.01,3.13)	0.58 (0.15,2.15)	0.87 (0.45,1.68)	0.86 (0.40,1.85)	0.44 (0.02,10.34)	0.90 (0.48,1.69)	1.01 (0.59,1.72)	Dulaglutide	1.02 (0.61,1.68)	1.06 (0.48,2.34)	0.90 (0.07,11.26)	1.02 (0.16,6.35)	1.24 (0.79,1.96)	1.29 (0.78,2.15)	1.28 (0.64,2.56)	1.40 (0.48,4.07)	1.32 (0.75,2.30)	1.40 (0.76,2.59)	*1.52 (1.01,2.29)	1.63 (0.98,2.70)	1.85 (0.82,4.17)	*1.68 (1.01,2.78)	2.08 (0.62,7.04)	1.81 (0.95,3.43)	5.03 (0.20,127.65)	2.29 (0.75,6.94)
0.12 (0.00,3.04)	0.57 (0.15,2.08)	0.86 (0.47,1.55)	0.85 (0.41,1.73)	0.43 (0.02,10.06)	0.88 (0.50,1.56)	0.99 (0.63,1.57)	0.98 (0.59,1.63)	Inject_semaglutide	1.05 (0.50,2.19)	0.89 (0.07,11.28)	1.00 (0.16,6.13)	1.22 (0.85,1.76)	1.27 (0.83,1.95)	1.26 (0.67,2.38)	1.37 (0.49,3.87)	1.30 (0.80,2.11)	1.38 (0.80,2.39)	*1.50 (1.10,2.02)	*1.60 (1.05,2.45)	1.82 (0.84,3.91)	*1.65 (1.08,2.53)	2.05 (0.61,6.84)	1.78 (1.00,3.17)	4.96 (0.20,124.18)	2.25 (0.77,6.60)
0.12 (0.00,3.08)	0.54 (0.13,2.29)	0.82 (0.35,1.92)	0.81 (0.32,2.06)	0.42 (0.02,10.19)	0.85 (0.37,1.94)	0.95 (0.44,2.02)	0.94 (0.43,2.07)	0.96 (0.46,2.00)	Alogliptin	0.85 (0.06,11.58)	0.96 (0.14,6.46)	1.17 (0.58,2.37)	1.22 (0.58,2.55)	1.21 (0.50,2.89)	1.31 (0.40,4.35)	1.24 (0.57,2.69)	1.32 (0.58,2.98)	1.43 (0.73,2.81)	1.53 (0.73,3.20)	1.74 (0.65,4.61)	1.58 (0.76,3.30)	1.96 (0.51,7.55)	1.70 (0.74,3.93)	4.74 (0.18,125.57)	2.15 (0.63,7.38)
0.14 (0.00,8.15)	0.64 (0.04,10.79)	0.97 (0.07,12.73)	0.95 (0.07,12.96)	0.49 (0.01,27.33)	1.00 (0.08,13.07)	1.12 (0.09,14.32)	1.11 (0.09,13.90)	1.13 (0.09,14.37)	1.18 (0.09,16.13)	Retatrutide	1.13 (0.05,24.91)	1.38 (0.11,17.41)	1.44 (0.11,18.29)	1.42 (0.11,18.91)	1.55 (0.10,23.37)	1.46 (0.11,18.83)	1.56 (0.12,20.28)	1.69 (0.13,21.11)	1.81 (0.14,23.00)	2.05 (0.15,28.25)	1.86 (0.15,23.73)	2.31 (0.14,37.25)	2.01 (0.15,26.37)	5.59 (0.09,331.62)	2.54 (0.17,38.92)
0.12 (0.00,4.78)	0.57 (0.06,5.09)	0.86 (0.13,5.50)	0.85 (0.13,5.66)	0.43 (0.01,15.94)	0.88 (0.14,5.63)	0.99 (0.16,6.11)	0.98 (0.16,6.16)	1.00 (0.16,6.13)	1.05 (0.15,7.07)	0.89 (0.04,19.56)	Vildagliptin	1.22 (0.20,7.39)	1.27 (0.21,7.80)	1.26 (0.19,8.19)	1.37 (0.18,10.59)	1.30 (0.21,8.06)	1.38 (0.22,8.72)	1.50 (0.25,8.93)	1.60 (0.26,9.80)	1.82 (0.27,12.41)	1.65 (0.27,10.11)	2.05 (0.24,17.33)	1.78 (0.28,11.37)	4.96 (0.13,194.77)	2.25 (0.29,17.73)
0.10 (0.00,2.47)	0.46 (0.13,1.69)	0.70 (0.40,1.22)	0.69 (0.35,1.37)	0.36 (0.02,8.16)	0.72 (0.43,1.22)	0.81 (0.54,1.21)	0.80 (0.51,1.27)	0.82 (0.57,1.18)	0.86 (0.42,1.73)	0.72 (0.06,9.14)	0.82 (0.14,4.94)	Dapagliflozin	1.04 (0.72,1.50)	1.03 (0.57,1.87)	1.12 (0.41,3.08)	1.06 (0.69,1.63)	1.13 (0.68,1.86)	1.22 (1.00,1.50)	1.31 (0.91,1.88)	1.49 (0.71,3.10)	1.35 (0.94,1.94)	1.68 (0.51,5.48)	1.46 (0.85,2.48)	4.05 (0.16,100.75)	1.84 (0.64,5.27)
0.10 (0.00,2.39)	0.45 (0.12,1.65)	0.67 (0.37,1.23)	0.66 (0.32,1.36)	0.34 (0.01,7.91)	0.70 (0.39,1.22)	0.78 (0.49,1.23)	0.77 (0.46,1.29)	0.79 (0.51,1.21)	0.82 (0.39,1.72)	0.70 (0.05,8.87)	0.79 (0.13,4.82)	0.96 (0.67,1.39)	Liraglutide	0.99 (0.53,1.87)	1.08 (0.38,3.04)	1.02 (0.63,1.66)	1.08 (0.63,1.88)	1.18 (0.87,1.59)	1.26 (0.82,1.92)	1.43 (0.66,3.08)	1.30 (0.85,1.99)	1.61 (0.48,5.38)	1.40 (0.79,2.50)	3.90 (0.16,97.62)	1.77 (0.60,5.19)
0.10 (0.00,2.50)	0.45 (0.11,1.81)	0.68 (0.32,1.45)	0.67 (0.28,1.58)	0.34 (0.02,7.67)	0.70 (0.34,1.45)	0.79 (0.41,1.51)	0.78 (0.39,1.56)	0.79 (0.42,1.50)	0.83 (0.35,1.99)	0.70 (0.05,9.35)	0.79 (0.12,5.16)	0.97 (0.54,1.76)	1.01 (0.54,1.90)	Ertugliflozin	1.09 (0.35,3.39)	1.03 (0.52,2.02)	1.09 (0.53,2.25)	1.19 (0.68,2.07)	1.27 (0.68,2.39)	1.44 (0.59,3.54)	1.31 (0.70,2.47)	1.63 (0.45,5.93)	1.41 (0.67,2.97)	3.93 (0.15,101.91)	1.79 (0.55,5.77)
0.09 (0.00,2.54)	0.41 (0.08,2.07)	0.62 (0.20,1.90)	0.62 (0.19,2.01)	0.32 (0.01,8.41)	0.64 (0.21,1.94)	0.72 (0.25,2.06)	0.72 (0.25,2.09)	0.73 (0.26,2.05)	0.76 (0.23,2.52)	0.65 (0.04,9.73)	0.73 (0.09,5.62)	0.89 (0.32,2.45)	0.93 (0.33,2.61)	0.92 (0.29,2.86)	Bexagliflozin	0.94 (0.33,2.73)	1.00 (0.34,2.99)	1.09 (0.40,2.93)	1.17 (0.41,3.28)	1.32 (0.39,4.46)	1.20 (0.43,3.38)	1.49 (0.32,6.90)	1.30 (0.43,3.92)	3.61 (0.13,103.49)	1.64 (0.39,6.85)
0.10 (0.00,2.37)	0.44 (0.12,1.65)	0.66 (0.35,1.26)	0.65 (0.31,1.39)	0.34 (0.01,7.83)	0.68 (0.37,1.26)	0.76 (0.46,1.28)	0.76 (0.43,1.33)	0.77 (0.47,1.26)	0.81 (0.37,1.75)	0.68 (0.05,8.80)	0.77 (0.12,4.80)	0.94 (0.61,1.45)	0.98 (0.60,1.60)	0.97 (0.50,1.91)	1.06 (0.37,3.06)	Linagliptin	1.06 (0.59,1.93)	1.15 (0.79,1.69)	1.24 (0.76,2.00)	1.40 (0.63,3.12)	1.27 (0.79,2.07)	1.58 (0.46,5.40)	1.37 (0.74,2.56)	3.82 (0.15,96.61)	1.74 (0.58,5.22)
0.09 (0.00,2.25)	0.41 (0.11,1.59)	0.62 (0.31,1.24)	0.61 (0.28,1.36)	0.32 (0.01,7.43)	0.64 (0.33,1.23)	0.72 (0.41,1.27)	0.71 (0.39,1.32)	0.73 (0.42,1.25)	0.76 (0.34,1.71)	0.64 (0.05,8.38)	0.73 (0.11,4.59)	0.89 (0.54,1.46)	0.92 (0.53,1.60)	0.91 (0.44,1.88)	1.00 (0.33,2.96)	0.94 (0.52,1.70)	Albiglutide	1.08 (0.69,1.71)	1.16 (0.67,2.00)	1.32 (0.57,3.05)	1.20 (0.69,2.07)	1.49 (0.42,5.21)	1.29 (0.66,2.53)	3.59 (0.14,91.71)	1.63 (0.53,5.04)
0.08 (0.00,2.01)	0.38 (0.11,1.36)	*0.57 (0.34,0.96)	0.57 (0.29,1.08)	0.29 (0.01,6.63)	*0.59 (0.36,0.96)	*0.66 (0.47,0.93)	*0.66 (0.44,0.99)	*0.67 (0.49,0.91)	0.70 (0.36,1.37)	0.59 (0.05,7.41)	0.67 (0.11,3.99)	0.82 (0.67,1.00)	0.85 (0.63,1.15)	0.84 (0.48,1.47)	0.92 (0.34,2.47)	0.87 (0.59,1.27)	0.92 (0.58,1.46)	Placebo_or_Control	1.07 (0.79,1.44)	1.21 (0.60,2.46)	1.10 (0.82,1.49)	1.37 (0.43,4.40)	1.19 (0.73,1.95)	3.31 (0.13,81.86)	1.51 (0.54,4.22)
0.08 (0.00,1.90)	0.35 (0.10,1.31)	*0.53 (0.29,0.97)	0.53 (0.26,1.08)	0.27 (0.01,6.28)	*0.55 (0.31,0.97)	*0.62 (0.39,0.98)	0.61 (0.37,1.02)	*0.62 (0.41,0.96)	0.65 (0.31,1.37)	0.55 (0.04,7.05)	0.62 (0.10,3.82)	0.76 (0.53,1.10)	0.79 (0.52,1.21)	0.79 (0.42,1.48)	0.86 (0.31,2.41)	0.81 (0.50,1.31)	0.86 (0.50,1.49)	0.93 (0.69,1.26)	Exenatide	1.13 (0.53,2.44)	1.03 (0.68,1.57)	1.28 (0.38,4.27)	1.11 (0.63,1.98)	3.09 (0.12,77.53)	1.41 (0.48,4.11)
0.07 (0.00,1.78)	0.31 (0.07,1.34)	0.47 (0.20,1.13)	0.47 (0.18,1.21)	0.24 (0.01,5.90)	0.49 (0.21,1.14)	0.55 (0.25,1.20)	0.54 (0.24,1.23)	0.55 (0.26,1.19)	0.58 (0.22,1.53)	0.49 (0.04,6.72)	0.55 (0.08,3.76)	0.67 (0.32,1.40)	0.70 (0.32,1.51)	0.69 (0.28,1.71)	0.76 (0.22,2.55)	0.71 (0.32,1.59)	0.76 (0.33,1.76)	0.82 (0.41,1.67)	0.88 (0.41,1.90)	Lixisenatide	0.91 (0.42,1.96)	1.13 (0.29,4.41)	0.98 (0.42,2.32)	2.73 (0.10,72.77)	1.24 (0.36,4.32)
0.07 (0.00,1.84)	0.34 (0.09,1.27)	*0.52 (0.28,0.94)	0.51 (0.25,1.05)	0.26 (0.01,6.09)	*0.54 (0.30,0.94)	*0.60 (0.38,0.94)	*0.60 (0.36,0.99)	*0.61 (0.40,0.93)	0.63 (0.30,1.32)	0.54 (0.04,6.83)	0.61 (0.10,3.71)	0.74 (0.52,1.06)	0.77 (0.50,1.18)	0.76 (0.41,1.44)	0.83 (0.30,2.34)	0.78 (0.48,1.27)	0.83 (0.48,1.44)	0.91 (0.67,1.22)	0.97 (0.64,1.48)	1.10 (0.51,2.37)	Saxagliptin	1.24 (0.37,4.14)	1.08 (0.61,1.92)	3.00 (0.12,73.09)	1.36 (0.47,3.99)
0.06 (0.00,1.80)	0.28 (0.05,1.55)	0.42 (0.12,1.50)	0.41 (0.11,1.57)	0.21 (0.01,5.97)	0.43 (0.12,1.53)	0.48 (0.14,1.63)	0.48 (0.14,1.62)	0.49 (0.15,1.63)	0.51 (0.13,1.97)	0.43 (0.03,6.96)	0.49 (0.06,4.12)	0.60 (0.18,1.95)	0.62 (0.19,2.07)	0.61 (0.17,2.24)	0.67 (0.15,3.10)	0.63 (0.19,2.16)	0.67 (0.19,2.36)	0.73 (0.23,2.34)	0.78 (0.23,2.61)	0.89 (0.23,3.47)	0.81 (0.24,2.69)	Efpeglenatide	0.87 (0.24,3.09)	2.42 (0.08,73.38)	1.10 (0.23,5.22)
0.07 (0.00,1.75)	0.32 (0.08,1.25)	*0.48 (0.23,0.98)	0.47 (0.21,1.07)	0.24 (0.01,5.78)	*0.50 (0.25,0.98)	0.56 (0.31,1.01)	0.55 (0.29,1.05)	0.56 (0.32,1.00)	0.59 (0.25,1.35)	0.50 (0.04,6.52)	0.56 (0.09,3.58)	0.69 (0.40,1.17)	0.71 (0.40,1.27)	0.71 (0.34,1.49)	0.77 (0.26,2.33)	0.73 (0.39,1.36)	0.77 (0.40,1.51)	0.84 (0.51,1.37)	0.90 (0.51,1.60)	1.02 (0.43,2.41)	0.93 (0.52,1.65)	1.15 (0.32,4.08)	Oral_semaglutide	2.78 (0.11,71.34)	1.26 (0.40,3.96)
0.02 (0.00,2.30)	0.11 (0.00,3.61)	0.17 (0.01,4.45)	0.17 (0.01,4.50)	0.09 (0.00,7.73)	0.18 (0.01,4.57)	0.20 (0.01,5.03)	0.20 (0.01,5.04)	0.20 (0.01,5.06)	0.21 (0.01,5.59)	0.18 (0.00,10.60)	0.20 (0.01,7.93)	0.25 (0.01,6.14)	0.26 (0.01,6.43)	0.25 (0.01,6.59)	0.28 (0.01,7.95)	0.26 (0.01,6.61)	0.28 (0.01,7.10)	0.30 (0.01,7.46)	0.32 (0.01,8.09)	0.37 (0.01,9.78)	0.33 (0.01,8.12)	0.41 (0.01,12.55)	0.36 (0.01,9.22)	Dapagliflozin_ Saxagliptin	0.45 (0.02,13.20)
0.05 (0.00,1.57)	0.25 (0.05,1.30)	0.38 (0.12,1.21)	0.38 (0.11,1.27)	0.19 (0.01,5.20)	0.39 (0.13,1.23)	0.44 (0.15,1.30)	0.44 (0.14,1.33)	0.44 (0.15,1.30)	0.46 (0.14,1.59)	0.39 (0.03,6.03)	0.44 (0.06,3.50)	0.54 (0.19,1.55)	0.57 (0.19,1.66)	0.56 (0.17,1.81)	0.61 (0.15,2.55)	0.58 (0.19,1.73)	0.61 (0.20,1.89)	0.66 (0.24,1.86)	0.71 (0.24,2.08)	0.81 (0.23,2.81)	0.73 (0.25,2.15)	0.91 (0.19,4.32)	0.79 (0.25,2.48)	2.20 (0.08,63.91)	Omarigliptin

Data presents RR [95%CIs]. Network meta-analysis results are presented as estimate effect sizes for the outcome of COPD risk. Interventions are reported in order of mean ranking of beneficially prophylactic effect on COPD risk, and outcomes are expressed as risk ratio (RR) (95% confidence intervals) (95%CIs). For the upper-right portion, RR of less than 1 indicates that the treatment specified in the row got more beneficial effect than that specified in the column. For the lower-left portion, RR of less than 1 indicates that the treatment specified in the column has more beneficial effect than that specified in the row. Bold results marked with * indicate statistical significance.

eTable 5K League table of secondary outcome: status asthmaticus in asthmatic episode

Exenatide	0.99 (0.01,91.29)	1.00 (0.01,92.00)	1.00 (0.01,92.06)	2.11 (0.04,106.23)	2.99 (0.12,73.35)	2.99 (0.04,206.30)	9.01 (0.10,831.98)	9.01 (0.18,453.06)	8.94 (0.18,450.25)	14.93 (0.18,1230.50)
1.01 (0.01,93.52)	Saxagliptin	1.01 (0.01,93.12)	1.01 (0.01,93.18)	2.13 (0.04,107.52)	3.02 (0.12,74.24)	3.03 (0.04,208.81)	9.12 (0.10,842.07)	9.12 (0.18,458.56)	9.05 (0.18,455.71)	15.12 (0.18,1245.43)
1.00 (0.01,92.77)	0.99 (0.01,91.66)	Canagliflozin	1.00 (0.01,92.43)	2.12 (0.04,106.65)	3.00 (0.12,73.64)	3.00 (0.04,207.12)	9.05 (0.10,835.29)	9.05 (0.18,454.86)	8.98 (0.18,452.03)	15.00 (0.18,1235.39)
1.00 (0.01,92.71)	0.99 (0.01,91.60)	1.00 (0.01,92.30)	Lixisenatide	2.12 (0.04,106.58)	3.00 (0.12,73.59)	3.00 (0.04,206.99)	9.04 (0.10,834.74)	9.04 (0.18,454.56)	8.97 (0.18,451.73)	14.99 (0.18,1234.57)
0.47 (0.01,23.89)	0.47 (0.01,23.61)	0.47 (0.01,23.79)	0.47 (0.01,23.80)	Empagliflozin	1.42 (0.15,13.62)	1.42 (0.04,50.78)	4.27 (0.08,215.11)	4.27 (0.17,104.60)	4.24 (0.17,103.98)	7.08 (0.16,312.41)
0.33 (0.01,8.21)	0.33 (0.01,8.11)	0.33 (0.01,8.18)	0.33 (0.01,8.18)	0.71 (0.07,6.78)	Placebo_or_Control	1.00 (0.06,16.00)	3.02 (0.12,73.92)	3.01 (0.31,28.88)	2.99 (0.31,28.73)	5.00 (0.24,104.07)
0.33 (0.00,23.06)	0.33 (0.00,22.78)	0.33 (0.00,22.96)	0.33 (0.00,22.97)	0.70 (0.02,25.24)	1.00 (0.06,15.97)	Liraglutide	3.01 (0.04,207.61)	3.01 (0.08,107.61)	2.99 (0.08,106.96)	4.99 (0.08,304.58)
0.11 (0.00,10.25)	0.11 (0.00,10.12)	0.11 (0.00,10.20)	0.11 (0.00,10.21)	0.23 (0.00,11.78)	0.33 (0.01,8.13)	0.33 (0.00,22.88)	Tirzepatide	1.00 (0.02,50.24)	0.99 (0.02,49.93)	1.66 (0.02,136.45)
0.11 (0.00,5.58)	0.11 (0.00,5.52)	0.11 (0.00,5.56)	0.11 (0.00,5.56)	0.23 (0.01,5.73)	0.33 (0.03,3.18)	0.33 (0.01,11.87)	1.00 (0.02,50.27)	Sitagliptin	0.99 (0.04,24.29)	1.66 (0.04,73.00)
0.11 (0.00,5.63)	0.11 (0.00,5.57)	0.11 (0.00,5.61)	0.11 (0.00,5.61)	0.24 (0.01,5.79)	0.33 (0.03,3.21)	0.33 (0.01,11.98)	1.01 (0.02,50.73)	1.01 (0.04,24.66)	Linagliptin	1.67 (0.04,73.67)
0.07 (0.00,5.52)	0.07 (0.00,5.45)	0.07 (0.00,5.49)	0.07 (0.00,5.50)	0.14 (0.00,6.23)	0.20 (0.01,4.17)	0.20 (0.00,12.22)	0.60 (0.01,49.68)	0.60 (0.01,26.56)	0.60 (0.01,26.40)	Dapagliflozin

Data presents RR [95%CIs]. Network meta-analysis results are presented as estimate effect sizes for the outcome of status asthmaticus risk. Interventions are reported in order of mean ranking of beneficially prophylactic effect on status asthmaticus risk, and outcomes are expressed as risk ratio (RR) (95% confidence intervals) (95%CIs). For the upper-right portion, RR of less than 1 indicates that the treatment specified in the row got more beneficial effect than that specified in the column. For the lower-left portion, RR of less than 1 indicates that the treatment specified in the column has more beneficial effect than that specified in the row. Bold results marked with * indicate statistical significance.

eTable 5L League table of secondary outcome: emphysema in COPD episode

Liraglutide	0.79 (0.02,29.21)	0.79 (0.02,29.26)	1.32 (0.03,56.20)	1.30 (0.03,55.52)	1.73 (0.18,16.59)	1.97 (0.09,43.65)	1.97 (0.15,26.31)	3.93 (0.13,117.17)	3.94 (0.56,27.95)	5.87 (0.14,250.01)	4.53 (0.35,58.61)	5.91 (0.14,251.79)	8.16 (0.48,138.73)
1.27 (0.03,47.11)	Dapagliflozin	1.00 (0.01,73.37)	1.67 (0.02,137.93)	1.65 (0.02,136.28)	2.19 (0.06,81.40)	2.50 (0.05,119.96)	2.50 (0.08,81.04)	5.00 (0.08,304.72)	5.00 (0.24,104.19)	7.45 (0.09,613.69)	5.76 (0.18,182.09)	7.50 (0.09,618.02)	10.36 (0.27,403.32)
1.27 (0.03,47.02)	1.00 (0.01,73.11)	Dulaglutide	1.67 (0.02,137.67)	1.65 (0.02,136.02)	2.19 (0.06,81.25)	2.50 (0.05,119.74)	2.50 (0.08,80.89)	4.99 (0.08,304.14)	4.99 (0.24,103.99)	7.44 (0.09,612.55)	5.75 (0.18,181.75)	7.49 (0.09,616.86)	10.34 (0.27,402.56)
0.76 (0.02,32.34)	0.60 (0.01,49.22)	0.60 (0.01,49.30)	Exenatide	0.99 (0.01,91.29)	1.31 (0.03,55.88)	1.49 (0.03,81.62)	1.49 (0.04,55.92)	2.98 (0.04,205.80)	2.99 (0.12,73.35)	4.45 (0.05,411.12)	3.44 (0.09,125.78)	4.48 (0.05,414.02)	6.19 (0.14,276.39)
0.77 (0.02,32.73)	0.60 (0.01,49.82)	0.61 (0.01,49.90)	1.01 (0.01,93.52)	Saxagliptin	1.33 (0.03,56.56)	1.51 (0.03,82.61)	1.51 (0.04,56.60)	3.02 (0.04,208.30)	3.02 (0.12,74.24)	4.51 (0.05,416.11)	3.48 (0.10,127.31)	4.54 (0.05,419.04)	6.26 (0.14,279.74)
0.58 (0.06,5.56)	0.46 (0.01,16.91)	0.46 (0.01,16.94)	0.76 (0.02,32.52)	0.75 (0.02,32.13)	Sitagliptin	1.14 (0.05,25.26)	1.14 (0.09,15.23)	2.28 (0.08,67.81)	2.28 (0.32,16.18)	3.40 (0.08,144.69)	2.62 (0.20,33.92)	3.42 (0.08,145.73)	4.72 (0.28,80.29)
0.51 (0.02,11.25)	0.40 (0.01,19.17)	0.40 (0.01,19.20)	0.67 (0.01,36.55)	0.66 (0.01,36.12)	0.88 (0.04,19.44)	Oral_semaglutide	1.00 (0.05,18.90)	2.00 (0.05,78.09)	2.00 (0.18,22.05)	2.98 (0.05,162.63)	2.30 (0.13,42.28)	3.00 (0.05,163.78)	4.14 (0.18,97.08)
0.51 (0.04,6.78)	0.40 (0.01,12.96)	0.40 (0.01,12.98)	0.67 (0.02,25.06)	0.66 (0.02,24.76)	0.88 (0.07,11.72)	1.00 (0.05,18.91)	Inject_semaglutide	2.00 (0.08,51.50)	2.00 (0.37,10.92)	2.98 (0.08,111.47)	2.30 (0.22,24.50)	3.00 (0.08,112.27)	4.14 (0.29,59.16)
0.25 (0.01,7.57)	0.20 (0.00,12.21)	0.20 (0.00,12.23)	0.34 (0.00,23.10)	0.33 (0.00,22.83)	0.44 (0.01,13.08)	0.50 (0.01,19.58)	0.50 (0.02,12.90)	Canagliflozin	1.00 (0.06,16.00)	1.49 (0.02,102.80)	1.15 (0.05,28.94)	1.50 (0.02,103.52)	2.07 (0.07,65.01)
0.25 (0.04,1.80)	0.20 (0.01,4.16)	0.20 (0.01,4.17)	0.33 (0.01,8.21)	0.33 (0.01,8.11)	0.44 (0.06,3.11)	0.50 (0.05,5.51)	0.50 (0.09,2.73)	1.00 (0.06,15.96)	Placebo_or_Control	1.49 (0.06,36.53)	1.15 (0.22,5.97)	1.50 (0.06,36.80)	2.07 (0.27,16.04)
0.17 (0.00,7.26)	0.13 (0.00,11.05)	0.13 (0.00,11.07)	0.22 (0.00,20.74)	0.22 (0.00,20.49)	0.29 (0.01,12.54)	0.34 (0.01,18.32)	0.34 (0.01,12.55)	0.67 (0.01,46.19)	0.67 (0.03,16.46)	Alogliptin	0.77 (0.02,28.23)	1.01 (0.01,92.93)	1.39 (0.03,62.03)
0.22 (0.02,2.85)	0.17 (0.01,5.49)	0.17 (0.01,5.50)	0.29 (0.01,10.63)	0.29 (0.01,10.50)	0.38 (0.03,4.93)	0.43 (0.02,7.98)	0.43 (0.04,4.62)	0.87 (0.03,21.79)	0.87 (0.17,4.51)	1.29 (0.04,47.29)	Empagliflozin	1.30 (0.04,47.63)	1.80 (0.13,24.88)
0.17 (0.00,7.22)	0.13 (0.00,10.98)	0.13 (0.00,11.00)	0.22 (0.00,20.62)	0.22 (0.00,20.37)	0.29 (0.01,12.47)	0.33 (0.01,18.21)	0.33 (0.01,12.48)	0.67 (0.01,45.92)	0.67 (0.03,16.37)	0.99 (0.01,91.73)	0.77 (0.02,28.06)	Ertugliflozin	1.38 (0.03,61.67)
0.12 (0.01,2.08)	0.10 (0.00,3.76)	0.10 (0.00,3.76)	0.16 (0.00,7.22)	0.16 (0.00,7.13)	0.21 (0.01,3.60)	0.24 (0.01,5.66)	0.24 (0.02,3.45)	0.48 (0.02,15.12)	0.48 (0.06,3.74)	0.72 (0.02,32.10)	0.56 (0.04,7.69)	0.72 (0.02,32.33)	Linagliptin

Data presents RR [95%CIs]. Network meta-analysis results are presented as estimate effect sizes for the outcome of emphysema risk. Interventions are reported in order of mean ranking of beneficially prophylactic effect on emphysema risk, and outcomes are expressed as risk ratio (RR) (95% confidence intervals) (95%CIs). For the upper-right portion, RR of less than 1 indicates that the treatment specified in the row got more beneficial effect than that specified in the column. For the lower-left portion, RR of less than 1 indicates that the treatment specified in the column has more beneficial effect than that specified in the row. Bold results marked with * indicate statistical significance.

eTable 5M League table of secondary outcome: chronic bronchitis in COPD episode

Dulaglutide	1.00 (0.02,50.36)	1.00 (0.02,50.18)	1.50 (0.06,40.54)	1.50 (0.06,40.57)	1.49 (0.05,40.24)	2.98 (0.02,468.75)	3.00 (0.31,28.80)	2.99 (0.12,73.46)	3.32 (0.19,56.58)	4.48 (0.09,225.76)	4.51 (0.25,80.74)	5.99 (0.35,101.39)	8.98 (0.18,452.42)	8.99 (0.37,220.60)
1.00 (0.02,50.39)	Liraglutide	1.00 (0.01,92.06)	1.50 (0.03,81.82)	1.50 (0.03,81.87)	1.49 (0.03,81.19)	2.98 (0.12,72.92)	3.00 (0.12,73.56)	3.00 (0.06,150.88)	3.32 (0.09,124.98)	4.48 (0.05,414.19)	4.51 (0.12,176.52)	5.99 (0.16,224.37)	8.98 (0.10,830.00)	8.99 (0.18,453.09)
1.00 (0.02,50.56)	1.00 (0.01,92.70)	Linagliptin	1.50 (0.03,82.09)	1.50 (0.03,82.15)	1.49 (0.03,81.46)	2.99 (0.01,762.84)	3.01 (0.12,73.81)	3.01 (0.06,151.39)	3.33 (0.09,125.40)	4.50 (0.05,415.59)	4.53 (0.12,177.11)	6.02 (0.16,225.13)	9.01 (0.10,832.80)	9.02 (0.18,454.62)
0.67 (0.02,18.08)	0.67 (0.01,36.47)	0.67 (0.01,36.34)	Dapagliflozin	1.00 (0.03,29.82)	0.99 (0.03,29.57)	1.99 (0.01,333.41)	2.00 (0.18,22.07)	2.00 (0.07,54.14)	2.22 (0.12,42.21)	2.99 (0.05,163.49)	3.01 (0.15,60.13)	4.00 (0.21,75.67)	6.00 (0.11,327.62)	6.00 (0.22,162.60)
0.67 (0.02,18.06)	0.67 (0.01,36.43)	0.66 (0.01,36.30)	1.00 (0.03,29.77)	Lixisenatide	0.99 (0.03,29.54)	1.99 (0.01,333.05)	2.00 (0.18,22.04)	2.00 (0.07,54.08)	2.21 (0.12,42.16)	2.99 (0.05,163.30)	3.01 (0.15,60.06)	4.00 (0.21,75.58)	5.99 (0.11,327.26)	6.00 (0.22,162.41)
0.67 (0.02,18.22)	0.67 (0.01,36.75)	0.67 (0.01,36.62)	1.01 (0.03,30.03)	1.01 (0.03,30.05)	Saxagliptin	2.01 (0.01,335.97)	2.02 (0.18,22.23)	2.01 (0.07,54.56)	2.23 (0.12,42.54)	3.02 (0.06,164.74)	3.04 (0.15,60.60)	4.03 (0.21,76.25)	6.04 (0.11,330.14)	6.05 (0.22,163.85)
0.34 (0.00,52.76)	0.34 (0.01,8.20)	0.33 (0.00,85.22)	0.50 (0.00,84.14)	0.50 (0.00,84.20)	0.50 (0.00,83.50)	Sitagliptin	1.01 (0.01,92.65)	1.00 (0.01,157.97)	1.11 (0.01,140.19)	1.50 (0.01,383.42)	1.51 (0.01,196.17)	2.01 (0.02,252.09)	3.01 (0.01,768.28)	3.02 (0.02,474.40)
0.33 (0.03,3.21)	0.33 (0.01,8.19)	0.33 (0.01,8.16)	0.50 (0.05,5.51)	0.50 (0.05,5.51)	0.50 (0.04,5.47)	0.99 (0.01,91.68)	Placebo_or_Control	1.00 (0.10,9.60)	1.11 (0.20,6.12)	1.50 (0.06,36.70)	1.51 (0.25,9.01)	2.00 (0.37,10.91)	3.00 (0.12,73.56)	3.00 (0.31,28.84)
0.33 (0.01,8.20)	0.33 (0.01,16.82)	0.33 (0.01,16.76)	0.50 (0.02,13.54)	0.50 (0.02,13.55)	0.50 (0.02,13.44)	1.00 (0.01,156.57)	1.00 (0.10,9.62)	Canagliflozin	1.11 (0.06,18.90)	1.50 (0.03,75.41)	1.51 (0.08,26.97)	2.00 (0.12,33.86)	3.00 (0.06,151.11)	3.00 (0.12,73.68)
0.30 (0.02,5.14)	0.30 (0.01,11.35)	0.30 (0.01,11.31)	0.45 (0.02,8.60)	0.45 (0.02,8.61)	0.45 (0.02,8.54)	0.90 (0.01,113.21)	0.90 (0.16,5.00)	0.90 (0.05,15.40)	Empagliflozin	1.35 (0.04,50.89)	1.36 (0.11,16.16)	1.81 (0.16,20.10)	2.71 (0.07,101.99)	2.71 (0.16,46.24)
0.22 (0.00,11.23)	0.22 (0.00,20.60)	0.22 (0.00,20.52)	0.33 (0.01,18.24)	0.33 (0.01,18.25)	0.33 (0.01,18.10)	0.66 (0.00,169.49)	0.67 (0.03,16.40)	0.67 (0.01,33.63)	0.74 (0.02,27.86)	Efpeglenatide	1.01 (0.03,39.35)	1.34 (0.04,50.01)	2.00 (0.02,185.03)	2.01 (0.04,101.00)
0.22 (0.01,3.97)	0.22 (0.01,8.67)	0.22 (0.01,8.64)	0.33 (0.02,6.62)	0.33 (0.02,6.63)	0.33 (0.02,6.57)	0.66 (0.01,85.62)	0.66 (0.11,3.97)	0.66 (0.04,11.88)	0.74 (0.06,8.74)	0.99 (0.03,38.85)	Exenatide	1.33 (0.11,15.64)	1.99 (0.05,77.85)	1.99 (0.11,35.66)
0.17 (0.01,2.82)	0.17 (0.00,6.24)	0.17 (0.00,6.22)	0.25 (0.01,4.72)	0.25 (0.01,4.73)	0.25 (0.01,4.69)	0.50 (0.00,62.36)	0.50 (0.09,2.73)	0.50 (0.03,8.45)	0.55 (0.05,6.16)	0.75 (0.02,27.99)	0.75 (0.06,8.86)	Oral_semaglutide	1.50 (0.04,56.09)	1.50 (0.09,25.38)
0.11 (0.00,5.61)	0.11 (0.00,10.28)	0.11 (0.00,10.25)	0.17 (0.00,9.11)	0.17 (0.00,9.11)	0.17 (0.00,9.04)	0.33 (0.00,84.63)	0.33 (0.01,8.19)	0.33 (0.01,16.79)	0.37 (0.01,13.91)	0.50 (0.01,46.11)	0.50 (0.01,19.65)	0.67 (0.02,24.98)	Sotagliflozin	1.00 (0.02,50.44)
0.11 (0.00,2.73)	0.11 (0.00,5.60)	0.11 (0.00,5.58)	0.17 (0.01,4.51)	0.17 (0.01,4.51)	0.17 (0.01,4.48)	0.33 (0.00,52.14)	0.33 (0.03,3.20)	0.33 (0.01,8.17)	0.37 (0.02,6.29)	0.50 (0.01,25.11)	0.50 (0.03,8.98)	0.67 (0.04,11.28)	1.00 (0.02,50.32)	Inject_semaglutide

Data presents RR [95%CIs]. Network meta-analysis results are presented as estimate effect sizes for the outcome of chronic bronchitis risk. Interventions are reported in order of mean ranking of beneficially prophylactic effect on chronic bronchitis risk, and outcomes are expressed as risk ratio (RR) (95% confidence intervals) (95%CIs). For the upper-right portion, RR of less than 1 indicates that the treatment specified in the row got more beneficial effect than that specified in the column. For the lower-left portion, RR of less than 1 indicates that the treatment specified in the column has more beneficial effect than that specified in the row. Bold results marked with * indicate statistical significance.

eTable 5N League table of acceptability: treatment discontinuation rate

Tirzepatide	1.00 (0.77,1.30)	0.90 (0.46,1.74)	1.04 (0.80,1.35)	1.07 (0.85,1.34)	1.04 (0.62,1.75)	1.11 (0.89,1.38)	1.12 (0.87,1.44)	1.15 (0.85,1.55)	1.15 (0.90,1.47)	1.16 (0.93,1.46)	1.19 (0.77,1.84)	1.20 (0.64,2.25)	1.19 (0.86,1.63)	1.19 (0.88,1.61)	1.21 (0.94,1.55)	1.23 (0.91,1.65)	1.28 (0.72,2.29)	1.25 (0.94,1.68)	1.30 (0.81,2.11)	1.26 (0.96,1.65)	1.30 (0.88,1.92)	1.32 (0.90,1.95)	*1.37 (1.13,1.65)	*1.49 (1.14,1.95)	2.09 (0.99,4.42)
1.00 (0.77,1.30)	Canagliflozin	0.90 (0.46,1.73)	1.03 (0.80,1.35)	1.06 (0.85,1.34)	1.04 (0.62,1.74)	1.11 (0.90,1.36)	1.12 (0.87,1.42)	1.15 (0.86,1.54)	1.15 (0.90,1.46)	1.16 (0.93,1.45)	1.19 (0.77,1.83)	1.20 (0.64,2.24)	1.18 (0.87,1.62)	1.19 (0.89,1.60)	1.21 (0.95,1.54)	1.23 (0.92,1.64)	1.28 (0.72,2.28)	1.25 (0.94,1.67)	1.30 (0.81,2.10)	1.26 (0.97,1.64)	1.30 (0.89,1.91)	1.32 (0.90,1.94)	*1.36 (1.14,1.64)	*1.49 (1.15,1.93)	2.09 (0.99,4.40)
1.11 (0.58,2.16)	1.12 (0.58,2.16)	Retatrutide	1.16 (0.61,2.20)	1.19 (0.62,2.28)	1.16 (0.52,2.57)	1.24 (0.65,2.35)	1.25 (0.65,2.40)	1.28 (0.65,2.52)	1.28 (0.67,2.47)	1.30 (0.68,2.48)	1.33 (0.63,2.80)	1.34 (0.56,3.21)	1.32 (0.67,2.62)	1.33 (0.68,2.62)	1.35 (0.70,2.60)	1.37 (0.70,2.69)	1.43 (0.62,3.30)	1.40 (0.72,2.72)	1.45 (0.67,3.15)	1.41 (0.73,2.73)	1.45 (0.71,2.98)	1.47 (0.72,3.03)	1.52 (0.81,2.87)	1.66 (0.86,3.22)	2.33 (0.89,6.10)
0.96 (0.74,1.26)	0.97 (0.74,1.26)	0.87 (0.45,1.65)	Dulaglutide	1.03 (0.82,1.30)	1.00 (0.60,1.68)	1.07 (0.86,1.33)	1.08 (0.84,1.39)	1.11 (0.82,1.50)	1.11 (0.87,1.42)	1.12 (0.89,1.41)	1.15 (0.74,1.78)	1.16 (0.62,2.17)	1.14 (0.83,1.57)	1.15 (0.85,1.56)	1.17 (0.91,1.50)	1.19 (0.88,1.60)	1.24 (0.70,2.21)	1.21 (0.94,1.56)	1.26 (0.78,2.04)	1.22 (0.93,1.60)	1.26 (0.85,1.86)	1.28 (0.86,1.89)	*1.32 (1.09,1.60)	*1.44 (1.12,1.85)	2.02 (0.96,4.26)
0.94 (0.74,1.18)	0.94 (0.75,1.18)	0.84 (0.44,1.61)	0.97 (0.77,1.23)	Inject_semaglutide	0.97 (0.59,1.62)	1.04 (0.87,1.25)	1.05 (0.84,1.31)	1.08 (0.82,1.42)	1.08 (0.87,1.34)	1.09 (0.90,1.33)	1.12 (0.73,1.70)	1.13 (0.61,2.09)	1.11 (0.83,1.49)	1.12 (0.85,1.48)	1.14 (0.92,1.41)	1.15 (0.88,1.52)	1.21 (0.69,2.12)	1.18 (0.90,1.53)	1.22 (0.77,1.95)	1.19 (0.93,1.51)	1.22 (0.85,1.77)	1.24 (0.86,1.80)	*1.28 (1.10,1.49)	*1.40 (1.11,1.76)	1.97 (0.94,4.11)
0.96 (0.57,1.62)	0.96 (0.58,1.61)	0.86 (0.39,1.92)	1.00 (0.59,1.68)	1.03 (0.62,1.70)	Ertugliflozin_Sitagliptin	1.07 (0.66,1.73)	1.08 (0.65,1.79)	1.11 (0.65,1.89)	1.11 (0.67,1.84)	1.12 (0.68,1.84)	1.14 (0.61,2.14)	1.16 (0.54,2.50)	1.14 (0.72,1.82)	1.15 (0.67,1.97)	1.17 (0.70,1.94)	1.18 (0.69,2.02)	1.24 (0.60,2.56)	1.21 (0.71,2.05)	1.26 (0.65,2.42)	1.22 (0.72,2.04)	1.26 (0.70,2.26)	1.27 (0.71,2.30)	1.32 (0.81,2.13)	1.44 (0.85,2.41)	2.02 (0.85,4.81)
0.90 (0.72,1.12)	0.90 (0.73,1.11)	0.81 (0.42,1.54)	0.93 (0.75,1.16)	0.96 (0.80,1.15)	0.94 (0.58,1.52)	Sitagliptin	1.01 (0.83,1.23)	1.04 (0.80,1.34)	1.04 (0.87,1.24)	1.05 (0.89,1.23)	1.07 (0.71,1.61)	1.08 (0.59,1.99)	1.07 (0.82,1.40)	1.07 (0.83,1.39)	1.09 (0.90,1.32)	1.11 (0.86,1.43)	1.16 (0.66,2.02)	1.13 (0.88,1.44)	1.18 (0.75,1.85)	1.14 (0.91,1.42)	1.17 (0.83,1.66)	1.19 (0.83,1.70)	*1.23 (1.10,1.38)	*1.34 (1.08,1.67)	1.89 (0.91,3.92)
0.89 (0.70,1.15)	0.90 (0.70,1.14)	0.80 (0.42,1.55)	0.93 (0.72,1.19)	0.95 (0.76,1.19)	0.93 (0.56,1.55)	0.99 (0.81,1.21)	Saxagliptin	1.03 (0.78,1.36)	1.03 (0.82,1.29)	1.04 (0.85,1.28)	1.06 (0.69,1.63)	1.08 (0.58,2.00)	1.06 (0.78,1.44)	1.07 (0.80,1.42)	1.08 (0.86,1.36)	1.10 (0.83,1.46)	1.15 (0.65,2.03)	1.12 (0.85,1.48)	1.17 (0.73,1.87)	1.13 (0.88,1.45)	1.17 (0.80,1.70)	1.18 (0.81,1.72)	*1.22 (1.04,1.44)	*1.33 (1.04,1.71)	1.87 (0.91,3.85)
0.87 (0.64,1.17)	0.87 (0.65,1.17)	0.78 (0.40,1.53)	0.90 (0.67,1.22)	0.93 (0.70,1.22)	0.90 (0.53,1.54)	0.96 (0.75,1.25)	0.97 (0.73,1.29)	Alogliptin	1.00 (0.76,1.32)	1.01 (0.78,1.31)	1.03 (0.65,1.63)	1.05 (0.55,1.98)	1.03 (0.73,1.45)	1.04 (0.75,1.44)	1.05 (0.79,1.39)	1.07 (0.77,1.48)	1.12 (0.62,2.02)	1.09 (0.79,1.50)	1.13 (0.69,1.86)	1.10 (0.82,1.48)	1.13 (0.75,1.71)	1.15 (0.76,1.73)	1.19 (0.94,1.49)	1.30 (0.96,1.75)	1.82 (0.85,3.88)
0.87 (0.68,1.11)	0.87 (0.69,1.11)	0.78 (0.41,1.50)	0.90 (0.70,1.15)	0.93 (0.75,1.15)	0.90 (0.54,1.50)	0.96 (0.81,1.15)	0.97 (0.77,1.22)	1.00 (0.76,1.32)	Albiglutide	1.01 (0.83,1.23)	1.03 (0.68,1.58)	1.05 (0.56,1.94)	1.03 (0.77,1.39)	1.04 (0.78,1.38)	1.05 (0.85,1.30)	1.07 (0.81,1.41)	1.12 (0.63,1.97)	1.09 (0.83,1.43)	1.13 (0.71,1.81)	1.10 (0.86,1.40)	1.13 (0.78,1.64)	1.15 (0.79,1.67)	*1.19 (1.01,1.39)	*1.30 (1.01,1.65)	1.82 (0.87,3.81)
0.86 (0.68,1.08)	0.86 (0.69,1.07)	0.77 (0.40,1.47)	0.89 (0.71,1.12)	0.92 (0.75,1.11)	0.89 (0.54,1.47)	0.95 (0.81,1.12)	0.96 (0.78,1.18)	0.99 (0.76,1.29)	0.99 (0.81,1.21)	Empagliflozin	1.02 (0.68,1.55)	1.04 (0.56,1.90)	1.02 (0.77,1.35)	1.03 (0.79,1.34)	1.04 (0.85,1.28)	1.06 (0.81,1.37)	1.10 (0.63,1.93)	1.08 (0.83,1.39)	1.12 (0.71,1.77)	1.09 (0.87,1.36)	1.12 (0.78,1.61)	1.14 (0.79,1.63)	*1.18 (1.04,1.33)	*1.28 (1.02,1.61)	1.80 (0.87,3.75)
0.84 (0.54,1.30)	0.84 (0.55,1.30)	0.75 (0.36,1.59)	0.87 (0.56,1.35)	0.90 (0.59,1.37)	0.87 (0.47,1.63)	0.93 (0.62,1.41)	0.94 (0.61,1.44)	0.97 (0.61,1.53)	0.97 (0.63,1.48)	0.98 (0.65,1.48)	Vildagliptin	1.01 (0.50,2.07)	1.00 (0.62,1.59)	1.00 (0.63,1.59)	1.02 (0.67,1.56)	1.03 (0.66,1.63)	1.08 (0.55,2.11)	1.05 (0.67,1.66)	1.10 (0.61,1.98)	1.06 (0.69,1.65)	1.10 (0.65,1.84)	1.11 (0.66,1.87)	1.15 (0.77,1.70)	1.25 (0.81,1.94)	1.76 (0.77,4.01)
0.83 (0.44,1.55)	0.83 (0.45,1.55)	0.75 (0.31,1.78)	0.86 (0.46,1.61)	0.88 (0.48,1.64)	0.86 (0.40,1.86)	0.92 (0.50,1.69)	0.93 (0.50,1.72)	0.96 (0.50,1.81)	0.96 (0.52,1.77)	0.97 (0.53,1.78)	0.99 (0.48,2.02)	Bexagliflozin	0.99 (0.52,1.88)	0.99 (0.52,1.88)	1.01 (0.54,1.86)	1.02 (0.54,1.93)	1.07 (0.48,2.39)	1.04 (0.55,1.97)	1.08 (0.52,2.27)	1.05 (0.56,1.96)	1.08 (0.55,2.15)	1.10 (0.55,2.18)	1.14 (0.63,2.06)	1.24 (0.66,2.32)	1.74 (0.68,4.44)
0.84 (0.61,1.16)	0.84 (0.62,1.15)	0.76 (0.38,1.50)	0.87 (0.64,1.20)	0.90 (0.67,1.21)	0.88 (0.55,1.40)	0.94 (0.72,1.22)	0.94 (0.70,1.28)	0.97 (0.69,1.37)	0.97 (0.72,1.31)	0.98 (0.74,1.30)	1.00 (0.63,1.60)	1.02 (0.53,1.94)	Ertugliflozin	1.01 (0.71,1.42)	1.02 (0.76,1.38)	1.04 (0.74,1.46)	1.08 (0.59,1.98)	1.06 (0.75,1.48)	1.10 (0.66,1.83)	1.07 (0.78,1.46)	1.10 (0.72,1.68)	1.12 (0.73,1.70)	1.15 (0.89,1.49)	1.26 (0.92,1.73)	1.77 (0.82,3.80)
0.84 (0.62,1.13)	0.84 (0.62,1.13)	0.75 (0.38,1.48)	0.87 (0.64,1.18)	0.89 (0.68,1.18)	0.87 (0.51,1.49)	0.93 (0.72,1.21)	0.94 (0.71,1.24)	0.96 (0.69,1.34)	0.96 (0.73,1.28)	0.97 (0.75,1.27)	1.00 (0.63,1.58)	1.01 (0.53,1.92)	0.99 (0.70,1.41)	Dapagliflozin	1.01 (0.76,1.35)	1.03 (0.74,1.43)	1.08 (0.60,1.95)	1.05 (0.76,1.45)	1.09 (0.66,1.80)	1.06 (0.78,1.43)	1.09 (0.72,1.65)	1.11 (0.73,1.68)	1.15 (0.91,1.45)	1.25 (0.93,1.69)	1.76 (0.85,3.62)
0.83 (0.64,1.06)	0.83 (0.65,1.05)	0.74 (0.39,1.43)	0.86 (0.67,1.10)	0.88 (0.71,1.09)	0.86 (0.52,1.43)	0.92 (0.76,1.11)	0.92 (0.73,1.16)	0.95 (0.72,1.26)	0.95 (0.77,1.17)	0.96 (0.78,1.18)	0.98 (0.64,1.50)	0.99 (0.54,1.84)	0.98 (0.73,1.32)	0.99 (0.74,1.31)	Liraglutide	1.01 (0.77,1.34)	1.06 (0.60,1.87)	1.03 (0.79,1.36)	1.08 (0.67,1.72)	1.04 (0.81,1.34)	1.08 (0.74,1.57)	1.09 (0.75,1.59)	1.13 (0.96,1.33)	1.23 (0.97,1.56)	1.73 (0.83,3.62)
0.81 (0.60,1.10)	0.82 (0.61,1.09)	0.73 (0.37,1.43)	0.84 (0.63,1.14)	0.87 (0.66,1.14)	0.85 (0.50,1.44)	0.90 (0.70,1.17)	0.91 (0.69,1.20)	0.94 (0.68,1.30)	0.94 (0.71,1.24)	0.95 (0.73,1.23)	0.97 (0.61,1.53)	0.98 (0.52,1.86)	0.97 (0.69,1.36)	0.97 (0.70,1.35)	0.99 (0.75,1.30)	Sotagliflozin	1.05 (0.58,1.89)	1.02 (0.74,1.40)	1.06 (0.65,1.74)	1.03 (0.76,1.38)	1.06 (0.70,1.60)	1.08 (0.72,1.62)	1.11 (0.88,1.40)	1.21 (0.90,1.63)	1.70 (0.80,3.63)
0.78 (0.44,1.39)	0.78 (0.44,1.38)	0.70 (0.30,1.61)	0.81 (0.45,1.44)	0.83 (0.47,1.46)	0.81 (0.39,1.68)	0.86 (0.50,1.51)	0.87 (0.49,1.54)	0.90 (0.50,1.62)	0.90 (0.51,1.58)	0.91 (0.52,1.58)	0.93 (0.47,1.81)	0.94 (0.42,2.10)	0.92 (0.51,1.68)	0.93 (0.51,1.68)	0.94 (0.53,1.66)	0.96 (0.53,1.73)	Teneligliptin	0.98 (0.54,1.76)	1.02 (0.50,2.05)	0.98 (0.55,1.75)	1.02 (0.53,1.93)	1.03 (0.54,1.96)	1.06 (0.62,1.83)	1.16 (0.65,2.07)	1.63 (0.66,4.03)
0.80 (0.60,1.07)	0.80 (0.60,1.07)	0.72 (0.37,1.40)	0.83 (0.64,1.07)	0.85 (0.65,1.11)	0.83 (0.49,1.41)	0.89 (0.69,1.13)	0.89 (0.68,1.18)	0.92 (0.67,1.27)	0.92 (0.70,1.21)	0.93 (0.72,1.20)	0.95 (0.60,1.49)	0.96 (0.51,1.82)	0.95 (0.68,1.33)	0.95 (0.69,1.32)	0.97 (0.74,1.27)	0.98 (0.71,1.35)	1.03 (0.57,1.85)	Efpeglenatide	1.04 (0.64,1.71)	1.01 (0.75,1.35)	1.04 (0.69,1.56)	1.06 (0.70,1.58)	1.09 (0.87,1.36)	1.19 (0.89,1.59)	1.67 (0.79,3.56)
0.77 (0.47,1.24)	0.77 (0.48,1.24)	0.69 (0.32,1.49)	0.79 (0.49,1.28)	0.82 (0.51,1.30)	0.80 (0.41,1.53)	0.85 (0.54,1.34)	0.86 (0.54,1.37)	0.88 (0.54,1.45)	0.88 (0.55,1.41)	0.89 (0.56,1.41)	0.91 (0.50,1.65)	0.92 (0.44,1.94)	0.91 (0.55,1.51)	0.91 (0.56,1.51)	0.93 (0.58,1.48)	0.94 (0.57,1.55)	0.98 (0.49,1.98)	0.96 (0.59,1.57)	Lixisenatide	0.97 (0.60,1.56)	1.00 (0.57,1.74)	1.01 (0.58,1.77)	1.05 (0.67,1.63)	1.14 (0.71,1.85)	1.61 (0.69,3.74)
0.79 (0.60,1.04)	0.79 (0.61,1.03)	0.71 (0.37,1.38)	0.82 (0.63,1.08)	0.84 (0.66,1.07)	0.82 (0.49,1.38)	0.88 (0.70,1.10)	0.88 (0.69,1.14)	0.91 (0.68,1.23)	0.91 (0.71,1.17)	0.92 (0.73,1.16)	0.94 (0.61,1.46)	0.95 (0.51,1.78)	0.94 (0.68,1.29)	0.94 (0.70,1.28)	0.96 (0.75,1.23)	0.97 (0.72,1.31)	1.02 (0.57,1.81)	0.99 (0.74,1.33)	1.03 (0.64,1.67)	Linagliptin	1.03 (0.70,1.52)	1.05 (0.71,1.54)	1.08 (0.89,1.31)	1.18 (0.90,1.54)	1.66 (0.79,3.50)
0.77 (0.52,1.13)	0.77 (0.52,1.13)	0.69 (0.34,1.41)	0.79 (0.54,1.17)	0.82 (0.56,1.18)	0.80 (0.44,1.43)	0.85 (0.60,1.20)	0.86 (0.59,1.25)	0.88 (0.59,1.33)	0.88 (0.61,1.28)	0.89 (0.62,1.28)	0.91 (0.54,1.53)	0.92 (0.46,1.83)	0.91 (0.60,1.39)	0.91 (0.61,1.38)	0.93 (0.64,1.35)	0.94 (0.63,1.42)	0.98 (0.52,1.87)	0.96 (0.64,1.44)	1.00 (0.57,1.74)	0.97 (0.66,1.43)	Oral_semaglutide	1.01 (0.63,1.64)	1.05 (0.75,1.47)	1.14 (0.78,1.69)	1.61 (0.72,3.57)
0.76 (0.51,1.12)	0.76 (0.52,1.11)	0.68 (0.33,1.39)	0.78 (0.53,1.16)	0.81 (0.56,1.17)	0.79 (0.44,1.42)	0.84 (0.59,1.20)	0.84 (0.58,1.23)	0.87 (0.58,1.31)	0.87 (0.60,1.26)	0.88 (0.61,1.26)	0.90 (0.53,1.51)	0.91 (0.46,1.81)	0.90 (0.59,1.37)	0.90 (0.60,1.36)	0.92 (0.63,1.33)	0.93 (0.62,1.40)	0.97 (0.51,1.84)	0.95 (0.63,1.42)	0.99 (0.57,1.72)	0.95 (0.65,1.41)	0.99 (0.61,1.59)	Omarigliptin	1.03 (0.74,1.45)	1.13 (0.76,1.66)	1.58 (0.71,3.51)
*0.73 (0.60,0.89)	*0.73 (0.61,0.88)	0.66 (0.35,1.24)	*0.76 (0.63,0.92)	*0.78 (0.67,0.91)	0.76 (0.47,1.23)	*0.81 (0.73,0.91)	*0.82 (0.69,0.96)	0.84 (0.67,1.06)	*0.84 (0.72,0.99)	*0.85 (0.75,0.97)	0.87 (0.59,1.29)	0.88 (0.49,1.60)	0.87 (0.67,1.12)	0.87 (0.69,1.10)	0.89 (0.75,1.04)	0.90 (0.72,1.13)	0.94 (0.55,1.62)	0.92 (0.73,1.14)	0.95 (0.61,1.48)	0.92 (0.76,1.12)	0.95 (0.68,1.34)	0.97 (0.69,1.36)	Placebo_or_Control	1.09 (0.90,1.32)	1.53 (0.74,3.15)
*0.67 (0.51,0.88)	*0.67 (0.52,0.87)	0.60 (0.31,1.16)	*0.69 (0.54,0.89)	*0.71 (0.57,0.90)	0.70 (0.41,1.17)	*0.74 (0.60,0.93)	*0.75 (0.58,0.96)	0.77 (0.57,1.04)	*0.77 (0.60,0.99)	*0.78 (0.62,0.98)	0.80 (0.51,1.23)	0.81 (0.43,1.51)	0.80 (0.58,1.09)	0.80 (0.59,1.08)	0.81 (0.64,1.03)	0.82 (0.61,1.11)	0.86 (0.48,1.53)	0.84 (0.63,1.12)	0.87 (0.54,1.41)	0.85 (0.65,1.11)	0.87 (0.59,1.29)	0.89 (0.60,1.31)	0.92 (0.76,1.11)	Exenatide	1.40 (0.67,2.96)
0.48 (0.23,1.01)	0.48 (0.23,1.01)	0.43 (0.16,1.12)	0.49 (0.23,1.04)	0.51 (0.24,1.06)	0.50 (0.21,1.18)	0.53 (0.26,1.10)	0.53 (0.26,1.10)	0.55 (0.26,1.17)	0.55 (0.26,1.15)	0.56 (0.27,1.16)	0.57 (0.25,1.29)	0.57 (0.23,1.47)	0.57 (0.26,1.22)	0.57 (0.28,1.17)	0.58 (0.28,1.21)	0.59 (0.28,1.25)	0.61 (0.25,1.51)	0.60 (0.28,1.27)	0.62 (0.27,1.45)	0.60 (0.29,1.27)	0.62 (0.28,1.38)	0.63 (0.28,1.40)	0.65 (0.32,1.34)	0.71 (0.34,1.50)	Dapagliflozin_Saxagliptin

Data presents RR [95%CIs]. Network meta-analysis results are presented as estimate effect sizes for the outcome of treatment discontinuation rate. Interventions are reported in order of mean ranking of acceptability, and outcomes are expressed as risk ratio (RR) (95% confidence intervals) (95%CIs). For the upper-right portion, RR of less than 1 indicates that the treatment specified in the row got better acceptability than that specified in the column. For the lower-left portion, RR of less than 1 indicates that the treatment specified in the column has better acceptability than that specified in the row. Bold results marked with * indicate statistical significance.

Abbreviation: 95%CIs: 95% confidence intervals; DPP4 inhibitor: dipeptidyl-peptidase 4 inhibitor; GLP-1 agonist: glucagon-like peptide-1 agonist; NMA: network meta-analysis; RR: risk ratio; RCT: randomised controlled trial; SGLT2 inhibitor: sodium–glucose cotransporter 2 inhibitor

eTable 6 Meta-regression for primary outcome: overall asthma-COPD overlap syndrome risk associated with treatment duration

Regimen	Coef.	Std. Err.	*p* value	[95% Confidence	Interval]
Albiglutide	0.000909	0.0104871	0.931	-0.0196453	0.0214634
Alogliptin	0.0129828	0.0213396	0.543	-0.0288419	0.0548076
Bexagliflozin	0.0017652	0.4973459	0.997	-0.9730148	0.9765453
Canagliflozin	0.0076102	0.0087916	0.387	-0.0096209	0.0248414
Dapagliflozin	0.0012897	0.0013086	0.324	-0.0012752	0.0038546
Dapagliflozin_Saxagliptin	-0.2820394	1.198534	0.814	-2.631123	2.067044
Dulaglutide	-0.0025392	0.002488	0.307	-0.0074155	0.0023371
Efpeglenatide	0.0296921	0.0203771	0.145	-0.0102463	0.0696306
Empagliflozin	0.0004213	0.0036253	0.907	-0.0066841	0.0075267
Ertugliflozin	-0.0023706	0.0080582	0.769	-0.0181642	0.0134231
Ertugliflozin_Sitagliptin	-0.9670587	1.759392	0.583	-4.415404	2.481287
Exenatide	-0.001391	0.0060224	0.817	-0.0131947	0.0104127
Inject_semaglutide	0.0029002	0.0038431	0.450	-0.0046322	0.0104326
Linagliptin	-0.0008621	0.0018795	0.646	-0.004546	0.0028217
Liraglutide	-0.0011899	0.0034112	0.727	-0.0078757	0.0054959
Lixisenatide	0.0516232	0.3193303	0.872	-0.5742527	0.677499
Omarigliptin	0.0106822	0.0188857	0.572	-0.0263332	0.0476975
Oral_semaglutide	-0.0026082	0.0120598	0.829	-0.0262449	0.0210286
Retatrutide	-0.2095667	1.315657	0.873	-2.788208	2.369074

Saxagliptin	0.0058084	0.0085602	0.497	-0.0109692	0.022586
Sitagliptin	-0.003773	0.003028	0.213	-0.0097078	0.0021618
Sotagliflozin	-0.0121953	0.0085942	0.156	-0.0290396	0.004649
Teneligliptin	-0.0621295	0.0538116	0.248	-0.1675983	0.0433393
Tirzepatide	-0.0018209	0.0138234	0.895	-0.0289143	0.0252724
Vildagliptin	0.0030015	0.8152703	0.997	-1.594899	1.600902

eTable 7A SUCRA for primary outcome: overall asthma-COPD overlap syndrome risk

Treatment	SUCRA	PrBest	MeanRank
Tirzepatide	76.5	7.5	6.9
Ertugliflozin_Sitagliptin	75.3	43.2	7.2
Inject_semaglutide	73.8	0.3	7.6
Canagliflozin	73.6	0.9	7.6
Sotagliflozin	71.3	1.9	8.2
Empagliflozin	67.2	0.2	9.2
Dulaglutide	65.0	0.0	9.7
Alogliptin	64.2	1.7	10.0
Ertugliflozin	62.6	0.2	10.4
Dapagliflozin	59.3	0.0	11.2
Retatrutide	57.9	17.1	11.5
Sitagliptin	57.1	0.1	11.7
Teneligliptin	55.4	12.3	12.1
Liraglutide	53.1	0.0	12.7
Dapagliflozin_Saxagliptin	46.2	10.6	14.5
Linagliptin	44.4	0.0	14.9
Vildagliptin	43.9	3.4	15.0
Bexagliflozin	41.4	0.3	15.7
Albiglutide	38.9	0.0	16.3
Lixisenatide	34.6	0.0	17.3

Placebo_or_Control	33.1	0.0	17.7
Exenatide	30.5	0.0	18.4
Saxagliptin	20.1	0.0	21.0
Omarigliptin	19.8	0.0	21.1
Efpeglenatide	18.3	0.3	21.4
Oral_semaglutide	16.6	0.0	21.9

eTable 7B SUCRA for primary outcome: subgroup of asthma risk

Treatment	SUCRA	PrBest	MeanRank
Dapagliflozin_Saxagliptin	84.8	57.5	4.5
Dapagliflozin	83.5	2.6	4.8
Ertugliflozin	80.5	6.9	5.5
Inject_semaglutide	74.9	1.0	6.8
Lixisenatide	67.0	5.2	8.6
Liraglutide	62.7	0.1	9.6
Alogliptin	57.7	4.3	10.7
Tirzepatide	57.6	1.9	10.7
Canagliflozin	56.8	0.5	10.9
Exenatide	55.2	0.7	11.3
Empagliflozin	51.9	0.2	12.1
Placebo_or_Control	44.8	0.0	13.7
Albiglutide	44.7	0.0	13.7
Linagliptin	43.6	0.2	14.0
Dulaglutide	42.3	0.0	14.3
Efpeglenatide	39.7	1.8	14.9
Omarigliptin	39.0	2.6	15.0
Sotagliflozin	37.9	0.6	15.3
Bexagliflozin	37.8	7.9	15.3
Teneligliptin	36.9	2.8	15.5

Oral_semaglutide	34.0	0.2	16.2
Sitagliptin	26.4	0.0	17.9
Vildagliptin	24.5	3.0	18.4
Saxagliptin	15.8	0.0	20.4

eTable 7C SUCRA for primary outcome: subgroup of COPD risk

Treatment	SUCRA	PrBest	MeanRank
Teneligliptin	89.8	57.8	3.6
Tirzepatide	80.4	6.7	5.9
Canagliflozin	73.0	0.1	7.7
Sotagliflozin	72.6	0.7	7.9
Ertugliflozin_Sitagliptin	72.2	22.1	8.0
Sitagliptin	72.1	0.4	8.0
Empagliflozin	66.4	0.0	9.4
Dulaglutide	66.3	0.0	9.4
Inject_semaglutide	65.4	0.0	9.7
Alogliptin	60.8	0.2	10.8
Retatrutide	58.1	7.3	11.5
Vildagliptin	55.1	3.1	12.2
Dapagliflozin	49.6	0.0	13.6
Liraglutide	47.1	0.0	14.2
Ertugliflozin	46.7	0.0	14.3
Bexagliflozin	44.0	0.1	15.0
Linagliptin	43.8	0.0	15.1
Albiglutide	39.6	0.0	16.1
Placebo_or_Control	31.5	0.0	18.1
Exenatide	27.4	0.0	19.2

Lixisenatide	25.2	0.0	19.7
Saxagliptin	25.0	0.0	19.8
Efpeglenatide	24.2	0.0	19.9
Oral_semaglutide	22.7	0.0	20.3
Dapagliflozin_Saxagliptin	21.5	1.5	20.6
Omarigliptin	19.6	0.0	21.1

Abbreviation: 95%CIs: 95% confidence intervals; DPP4 inhibitor: dipeptidyl-peptidase 4 inhibitor; GLP-1 agonist: glucagon-like peptide-1 agonist; NMA: network meta-analysis; RR: risk ratio; RCT: randomised controlled trial; SGLT2 inhibitor: sodium–glucose cotransporter 2 inhibitor; SUCRA: summary under the cumulative ranking curve

eTable 8A Heterogeneity for primary outcome: overall asthma-COPD overlap syndrome risk

	Heterogeneity statistic	degrees of freedom	*p*	*I squared*	*Tau-squared*	Treatments used	
C - A	4.05	5	0.542	0.00%	0	A:	Placebo_or_Control
D - A	3.42	6	0.754	0.00%	0	B:	Liraglutide
F - A	3.33	2	0.189	39.90%	0.8244	C:	Albiglutide
H - A	3.05	11	0.99	0.00%	0	D:	Canagliflozin
I - A	0.29	2	0.867	0.00%	0	E:	Teneligliptin
K - A	5.76	9	0.764	0.00%	0	F:	Efpeglenatide
L - A	3.05	4	0.549	0.00%	0	G:	Retatrutide
B - A	4.59	6	0.598	0.00%	0	H:	Empagliflozin
N - A	2.01	3	0.569	0.00%	0	I:	Ertugliflozin
O - A	1.75	3	0.625	0.00%	0	J:	Vildagliptin
R - A	0	0	.	.%	0	K:	Dapagliflozin
P - A	0.34	2	0.844	0.00%	0	L:	Dulaglutide
S - A	4.9	10	0.898	0.00%	0	M:	Linagliptin
X - A	5.96	12	0.918	0.00%	0	N:	Sotagliflozin
X - D	0	0	.	.%	0	O:	Exenatide
X - C	0	1	0.996	0.00%	0	P:	Oral_semaglutide
X - L	0	0	.	.%	0	Q:	Ertugliflozin_Sitagliptin
O - L	0	0	.	.%	0	R:	Lixisenatide
X - S	0	0	.	.%	0	S:	Inject_semaglutide
S - B	0.71	1	0.399	0.00%	0	T:	Omarigliptin

W - V	0	0	.	.%	0	U:	Alogliptin
X - H	0.3	1	0.586	0.00%	0	V:	Dapagliflozin_Saxagliptin
X - B	0	0	.	.%	0	W:	Saxagliptin
X - P	0	0	.	.%	0	X:	Sitagliptin
O - B	0	0	.	.%	0	Y:	Tirzepatide
S - O	0	0	.	.%	0	Z:	Bexagliflozin
C - B	0	0	.	.%	0		
L - F	0	0	.	.%	0		
S - L	0	0	.	.%	0		
S - D	0	0	.	.%	0		
Y - A	1.47	6	0.962	0.00%	0		
Y - L	0	0	.	.%	0		
Y - S	0	0	.	.%	0		
Z - A	0	0	.	.%	0		
U - A	0.46	2	0.796	0.00%	0		
M - A	4.79	6	0.57	0.00%	0		
T - A	0.32	1	0.572	0.00%	0		
W - A	1.21	6	0.977	0.00%	0		
V - K	0	0	.	.%	0		
Q - I	0	0	.	.%	0		
X - I	0	0	.	.%	0		
X - Q	0	0	.	.%	0		

J - A	0	0	.	.%	0
E - A	1.33	1	0.248	25.00%	0.7715
G - A	0	0	.	.%	0
L - G	0	0	.	.%	0

eTable 8B Heterogeneity for primary outcome: subgroup of asthma risk

	Heterogeneity statistic	degrees of freedom	*p*	*I squared*	*Tau-squared*	Treatments used	
C - A	4.86	4	0.302	17.70%	0.1753	A:	Placebo_or_Control
D - A	3.14	6	0.79	0.00%	0	B:	Liraglutide
F - A	0.04	1	0.843	0.00%	0	C:	Albiglutide
H - A	5	7	0.66	0.00%	0	D:	Canagliflozin
I - A	0.71	2	0.7	0.00%	0	E:	Vildagliptin
K - A	1.17	4	0.882	0.00%	0	F:	Efpeglenatide
L - A	2.19	2	0.335	8.60%	0.1243	G:	Teneligliptin
B - A	2.6	4	0.627	0.00%	0	H:	Empagliflozin
N - A	0.34	1	0.562	0.00%	0	I:	Ertugliflozin
O - A	0.38	2	0.826	0.00%	0	J:	Omarigliptin
R - A	0	0	.	.%	0	K:	Dapagliflozin
P - A	4.7	2	0.095	57.50%	2.6195	L:	Dulaglutide
S - A	5.05	7	0.654	0.00%	0	M:	Linagliptin
V - A	2.99	9	0.965	0.00%	0	N:	Sotagliflozin
V - D	0	0	.	.%	0	O:	Exenatide
O - L	0	0	.	.%	0	P:	Oral_semaglutide
S - B	0	0	.	.%	0	Q:	Alogliptin
V - P	0	0	.	.%	0	R:	Lixisenatide
S - O	0	0	.	.%	0	S:	Inject_semaglutide
C - B	0	0	.	.%	0	T:	Dapagliflozin_Saxagliptin

L - F	0	0	.	.%	0	U:	Saxagliptin
X - S	0	0	.	.%	0	V:	Sitagliptin
W - A	0	0	.	.%	0	W:	Bexagliflozin
Q - A	0.29	2	0.865	0.00%	0	X:	Tirzepatide
M - A	0.97	4	0.914	0.00%	0		
J - A	0.35	1	0.555	0.00%	0		
U - A	1.76	4	0.779	0.00%	0		
T - K	0	0	.	.%	0		
V - H	0	0	.	.%	0		
E - A	0	0	.	.%	0		
G - A	0.36	1	0.549	0.00%	0		

eTable 8C Heterogeneity for primary outcome: subgroup of COPD risk

	Heterogeneity statistic	degrees of freedom	*p*	*I squared*	*Tau-squared*	Treatments used	
C - A	0.09	2	0.956	0.00%	0	A:	Placebo_or_Control
D - A	4.5	4	0.343	11.10%	0.0538	B:	Liraglutide
F - A	3.18	2	0.204	37.10%	1.0153	C:	Albiglutide
I - A	0	0	.	.%	0	D:	Canagliflozin
K - A	8.57	8	0.379	6.70%	0.0091	E:	Teneligliptin
L - A	0.78	2	0.676	0.00%	0	F:	Efpeglenatide
B - A	2.29	4	0.683	0.00%	0	G:	Vildagliptin
N - A	1.65	3	0.649	0.00%	0	H:	Empagliflozin
O - A	0.96	2	0.62	0.00%	0	I:	Ertugliflozin
R - A	0	0	.	.%	0	J:	Ertugliflozin_Sitagliptin
P - A	2.44	1	0.118	59.00%	0.302	K:	Dapagliflozin
S - A	2.6	6	0.858	0.00%	0	L:	Dulaglutide
X - A	3.6	5	0.609	0.00%	0	M:	Linagliptin
X - C	0	1	0.996	0.00%	0	N:	Sotagliflozin
X - S	0	0	.	.%	0	O:	Exenatide
W - V	0	0	.	.%	0	P:	Oral_semaglutide
X - B	0	0	.	.%	0	Q:	Retatrutide
X - P	0	0	.	.%	0	R:	Lixisenatide
O - B	0	0	.	.%	0	S:	Inject_semaglutide
L - F	0	0	.	.%	0	T:	Omarigliptin

S - L	0	0	.	.%	0	U:	Alogliptin
S - B	0	0	.	.%	0	V:	Dapagliflozin_Saxagliptin
S - D	0	0	.	.%	0	W:	Saxagliptin
Y - A	1.89	3	0.596	0.00%	0	X:	Sitagliptin
Y - L	0	0	.	.%	0	Y:	Tirzepatide
Y - S	0	0	.	.%	0	Z:	Bexagliflozin
Z - A	0	0	.	.%	0		
U - A	0.09	1	0.766	0.00%	0		
M - A	3.62	6	0.728	0.00%	0		
T - A	0	0	.	.%	0		
W - A	0.04	3	0.998	0.00%	0		
J - I	0	0	.	.%	0		
X - I	0	0	.	.%	0		
X - J	0	0	.	.%	0		
G - A	0	0	.	.%	0		
E - A	0	0	.	.%	0		
Q - A	0	0	.	.%	0		
Q - L	0	0	.	.%	0		

Abbreviation: 95%CIs: 95% confidence intervals; DPP4 inhibitor: dipeptidyl-peptidase 4 inhibitor; GLP-1 agonist: glucagon-like peptide-1 agonist; NMA: network meta-analysis; RR: risk ratio; RCT: randomised controlled trial; SGLT2 inhibitor: sodium–glucose cotransporter 2 inhibitor

eTable 9A Side-splitting model inconsistency for primary outcome: overall asthma-COPD overlap syndrome risk

Side	Direct		Indirect		Difference			tau		
	Coef.	Std. Err.	Coef.	Std. Err.	Coef.	Std. Err.	P>z		Treatments used	
A B	-0.1890351	0.1394864	-0.5669716	0.7927714	0.3779365	0.8049482	0.639	2.54E-08	A:	Placebo_or_Control
A C	-0.0527799	0.1988552	0.0007463	1.093471	-0.0535263	1.11344	0.962	9.17E-07	B:	Liraglutide
A D	-0.4349919	0.2269969	-1.897437	1.387014	1.462445	1.405455	0.298	5.43E-07	C:	Albiglutide
A E	.	.	.	.	.	.	.	.	D:	Canagliflozin
A F	0.4477922	0.5868779	0.5822328	1.55815	-0.1344406	1.665009	0.936	2.94E-08	E:	Teneligliptin
A G *	-0.3250197	1.624713	-0.9584883	3.268827	0.6334685	3.994819	0.874	4.86E-08	F:	Efpeglenatide
A H *	-0.3334874	0.1585278	-2.688108	1.70236	2.35462	1.714046	0.17	4.27E-07	G:	Retatrutide
A I	-0.3346137	0.2478655	0.1515357	1.64308	-0.4861495	1.661671	0.77	1.21E-07	H:	Empagliflozin
A J	.	.	.	.	.	.	.	.	I:	Ertugliflozin
A K	-0.2819978	0.0979404	2.345	2.308203	-2.626998	2.310279	0.256	3.58E-07	J:	Vildagliptin
A L *	-0.386787	0.1886997	0.3468677	0.777782	-0.7336547	0.8133672	0.367	4.55E-08	K:	Dapagliflozin
A M	.	.	.	.	.	.	.	.	L:	Dulaglutide
A N	.	.	.	.	.	.	.	.	M:	Linagliptin
A O	0.0266854	0.146912	0.9391521	1.02288	-0.9124666	1.033374	0.377	1.95E-07	N:	Sotagliflozin
A P	0.3542244	0.2276925	-1.382474	1.427028	1.736698	1.445079	0.229	1.17E-07	O:	Exenatide
A R	.	.	.	.	.	.	.	.	P:	Oral_semaglutide
A S	-0.4742116	0.1430448	0.2205702	0.6862461	-0.6947818	0.7023566	0.323	9.64E-08	Q:	Ertugliflozin_Sitagliptin
A T	.	.	.	.	.	.	.	.	R:	Lixisenatide
A U	.	.	.	.	.	.	.	.	S:	Inject_semaglutide

A W	0.2045739	0.1397836	-2.422424	2.306071	2.626998	2.310304	0.256	1.70E-08	T:	Omarigliptin
A X	-0.2238536	0.215581	-0.4371633	0.56113	0.2133097	0.6148205	0.729	1.33E-06	U:	Alogliptin
A Y	-0.3089638	0.5214564	-1.981236	1.073767	1.672272	1.193689	0.161	3.65E-07	V:	Dapagliflozin_Saxagliptin
A Z	.	.	.	.	.	.	.	.	W:	Saxagliptin
B C	-1.088784	1.631488	0.176235	0.2401439	-1.265019	1.649067	0.443	1.16E-07	X:	Sitagliptin
B O	1.958703	1.509034	0.2151387	0.2009179	1.743565	1.522351	0.252	3.72E-07	Y:	Tirzepatide
B S	0.2200701	1.115367	-0.2582968	0.1978684	0.4783669	1.132874	0.673	1.58E-07	Z:	Bexagliflozin
B X	-0.0061539	1.412036	-0.0558077	0.2414688	0.0496538	1.432534	0.972	3.13E-07		
C X	-1.042888	1.13438	-0.1521257	0.2815362	-0.8907619	1.168789	0.446	1.62E-07		
D S	1.103688	1.631438	0.0004946	0.2660928	1.103194	1.652996	0.505	4.13E-08		
D X	1.763067	1.580058	0.1631564	0.3010237	1.599911	1.607567	0.32	3.16E-08		
F L	-0.9162907	1.547595	-0.7818501	0.6141713	-0.1344407	1.665009	0.936	4.02E-08		
G L *	0.3035135	1.624855	-0.329955	3.268614	0.6334685	3.994819	0.874	1.58E-07		
H X	1.13179	0.930554	0.0254284	0.2574783	1.106362	0.9661998	0.252	5.79E-08		
I Q *	-1.076322	1.631752	-0.1040227	3.323938	-0.9722989	3.323342	0.77	7.36E-07		
I X *	-0.3994349	1.631144	0.0867145	0.3170479	-0.4861495	1.661671	0.77	4.02E-07		
K V	-1.041815	1.629122	1.585184	1.638101	-2.626999	2.310284	0.256	1.87E-07		
L O	-0.3137938	1.579678	0.3962958	0.2331165	-0.7100896	1.5977	0.657	9.27E-08		
L S	1.093616	1.631973	-0.1320346	0.2288383	1.225651	1.647939	0.457	2.40E-10		
L X	-0.8390868	0.6863195	0.2300583	0.2767574	-1.069145	0.7396876	0.148	9.08E-08		
L Y	-2.193049	1.630437	-0.0977505	0.5218314	-2.095299	1.711909	0.221	8.01E-08		
O S	-1.096146	1.631482	-0.479828	0.2024411	-0.6163182	1.643994	0.708	1.88E-07		

P X	1.095037	1.413203	-0.6416614	0.3018462	1.736698	1.445079	0.229	2.60E-07
Q X *	0.6768867	1.998479	1.649185	2.656058	-0.9722987	3.323342	0.77	1.62E-07
S X	-0.4017954	1.631868	0.2022974	0.242289	-0.6040928	1.649757	0.714	7.37E-09
S Y	-1.100033	1.413208	-0.061757	0.5163502	-1.038276	1.504585	0.49	1.08E-07
V W	-1.098612	1.629185	1.528386	1.638038	-2.626999	2.310284	0.256	2.21E-08

eTable 9B Side-splitting model inconsistency for primary outcome: subgroup of asthma risk

Side	Direct		Indirect		Difference			tau		
	Coef.	Std. Err.	Coef.	Std. Err.	Coef.	Std. Err.	P>z		Treatments used	
A B	-0.3649519	0.3035036	-0.0825837	1.416164	-0.2823682	1.448304	0.845	4.43E-07	A:	Placebo_or_Control
A C	0.0884317	0.3680664	-1.49107	1.659123	1.579502	1.699459	0.353	1.31E-06	B:	Liraglutide
A D *	-0.184261	0.4395582	-1.448516	2.681141	1.264255	2.716861	0.642	7.12E-07	C:	Albiglutide
A E	.	.	.	.	.	.	.	.	D:	Canagliflozin
A F	0.137836	0.9396432	0.4747886	1.671992	-0.3369525	1.917938	0.861	3.55E-07	E:	Vildagliptin
A G	.	.	.	.	.	.	.	.	F:	Efpeglenatide
A H *	-0.1583577	0.380384	1.598257	3.24622	-1.756614	3.290508	0.593	9.01E-07	G:	Teneligliptin
A I	.	.	.	.	.	.	.	.	H:	Empagliflozin
A J	.	.	.	.	.	.	.	.	I:	Ertugliflozin
A K *	-0.8658161	0.3033494	0.3830361	3609.05	-1.248852	3609.05	1	9.11E-06	J:	Omarigliptin
A L	-0.2014811	0.4096898	1.50694	1.162534	-1.708421	1.30284	0.19	4.88E-07	K:	Dapagliflozin
A M	.	.	.	.	.	.	.	.	L:	Dulaglutide
A N	.	.	.	.	.	.	.	.	M:	Linagliptin
A O	-0.282023	0.5089571	0.3584239	1.415613	-0.6404468	1.504285	0.67	5.74E-07	N:	Sotagliflozin
A P	0.8391398	0.7663454	-1.873894	1.67148	2.713034	1.838785	0.14	1.15E-06	O:	Exenatide
A Q	.	.	.	.	.	.	.	.	P:	Oral_semaglutide
A R	.	.	.	.	.	.	.	.	Q:	Alogliptin
A S	-0.7508756	0.3481357	0.5920757	1.151515	-1.342951	1.209476	0.267	2.39E-08	R:	Lixisenatide
A U	.	.	.	.	.	.	.	.	S:	Inject_semaglutide

A V	0.5521445	0.3996388	-0.2486061	1.028239	0.8007506	1.151762	0.487	7.80E-07	T:	Dapagliflozin_Saxagliptin
A W	.	.	.	.	.	.	.	.	U:	Saxagliptin
B C	-1.088784	1.631488	0.4907179	0.4758229	-1.579502	1.699459	0.353	2.51E-07	V:	Sitagliptin
B S	1.273465	1.576882	-0.4120808	0.4568102	1.685546	1.64369	0.305	0.0000363	W:	Bexagliflozin
D V	1.644775	1.581241	0.5072488	0.5809484	1.137526	1.679659	0.498	9.83E-07	X:	Tirzepatide
F L	-0.4054651	1.631477	-0.0685122	1.00835	-0.3369529	1.917938	0.861	2.16E-08		
H V	-0.4063994	1.580458	0.6551021	0.5332737	-1.061502	1.669579	0.525	1.00E-06		
K T *	-1.041815	1.629122	1.445525	7060.963	-2.487339	7060.963	1	0.0000125		
L O	-0.2956717	1.581282	-0.2612649	0.6246254	-0.0344068	1.692171	0.984	1.72E-07		
O S	-1.096146	1.631482	-0.3316946	0.6034791	-0.7644516	1.739518	0.66	2.67E-07		
P V	2.192226	1.63212	-0.5208076	0.8469443	2.713034	1.838785	0.14	1.10E-07		
S X	-2.197225	1.632124	0.9657801	0.7963295	-3.163005	1.816032	0.082	6.01E-08		

eTable 9C Side-splitting model inconsistency for primary outcome: subgroup of COPD risk

Side	Direct		Indirect		Difference			tau		
	Coef.	Std. Err.	Coef.	Std. Err.	Coef.	Std. Err.	P>z		Treatments used	
A B	-0.1413011	0.1569774	-0.9337693	0.9671431	0.7924682	0.9798019	0.419	1.72E-07	A:	Placebo_or_Control
A C	-0.1038804	0.2367823	0.7282362	1.480776	-0.8321166	1.503403	0.58	4.51E-06	B:	Liraglutide
A D	-0.5331202	0.2687888	-1.514636	1.638806	0.9815159	1.660702	0.555	8.25E-08	C:	Albiglutide
A E	.	.	.	.	.	.	.	.	D:	Canagliflozin
A F	0.3655328	0.638917	-0.0181557	1.644995	0.3836885	1.764717	0.828	8.71E-07	E:	Teneligliptin
A G	.	.	.	.	.	.	.	.	F:	Efpeglenatide
A I	-0.1723966	0.2886784	-0.1249722	1.650096	-0.0474244	1.675157	0.977	8.42E-07	G:	Vildagliptin
A K	.	.	.	.	.	.	.	.	H:	Empagliflozin
A L *	-0.4335009	0.2137392	-0.0868155	0.9934646	-0.3466854	1.016197	0.733	2.20E-06	I:	Ertugliflozin
A M	.	.	.	.	.	.	.	.	J:	Ertugliflozin_Sitagliptin
A N	.	.	.	.	.	.	.	.	K:	Dapagliflozin
A O	0.0505124	0.1530324	1.815479	1.517052	-1.764966	1.524751	0.247	3.32E-07	L:	Dulaglutide
A P	0.1920361	0.2539241	-0.5381569	1.651061	0.7301931	1.670473	0.662	4.66E-09	M:	Linagliptin
A Q *	-0.3250197	1.624713	-1.127186	3.275849	0.8021665	4.000567	0.841	1.37E-06	N:	Sotagliflozin
A R	.	.	.	.	.	.	.	.	O:	Exenatide
A S	-0.4162406	0.1576121	-0.0452057	0.8123876	-0.3710349	0.8285186	0.654	1.48E-06	P:	Oral_semaglutide
A T	.	.	.	.	.	.	.	.	Q:	Retatrutide
A U	.	.	.	.	.	.	.	.	R:	Lixisenatide
A W *	0.0994445	0.1519557	-1.068532	1777.745	1.167977	1777.745	0.999	5.55E-06	S:	Inject_semaglutide

A X	-0.5680225	0.2677426	-0.2755928	0.6600719	-0.2924298	0.7165831	0.683	7.01E-07	T:	Omarigliptin
A Y	-0.7211396	0.7856583	-1.514914	1.160953	0.7937742	1.40181	0.571	1.08E-06	U:	Alogliptin
A Z	.	.	.	.	.	.	.	.	V:	Dapagliflozin_Saxagliptin
B O	1.958703	1.509034	0.1937371	0.2183607	1.764966	1.524751	0.247	7.03E-07	W:	Saxagliptin
B S	-0.8000683	1.578344	-0.2298494	0.2201319	-0.5702189	1.593621	0.72	7.43E-07	X:	Sitagliptin
B X	-0.0061539	1.412036	-0.3794167	0.2949245	0.3732629	1.442507	0.796	6.64E-06	Y:	Tirzepatide
C X	-1.062213	1.134456	-0.3863856	0.3479345	-0.6758275	1.186268	0.569	4.24E-07	Z:	Bexagliflozin
D S	1.103688	1.631438	0.1221724	0.3103879	0.9815161	1.660702	0.555	1.72E-06		
F L	-0.4054651	1.631477	-0.7891539	0.6726874	0.3836888	1.764717	0.828	3.88E-06		
I J *	-1.076322	1.631752	-0.9814725	3.350905	-0.0948491	3.350314	0.977	6.25E-07		
I X *	-0.3994349	1.631144	-0.3520112	0.3814713	-0.0474237	1.675157	0.977	4.82E-06		
J X *	0.6768867	1.998479	0.7717278	2.689731	-0.0948411	3.350314	0.977	4.13E-06		
L Q *	-0.3035135	1.624855	0.498653	3.275637	-0.8021665	4.000567	0.841	1.42E-06		
L S	1.093616	1.631973	-0.0118699	0.2614368	1.105486	1.652781	0.504	5.47E-07		
L Y	-2.193049	1.630437	-0.2152523	0.7389894	-1.977797	1.790092	0.269	2.32E-07		
P X	-0.0035753	1.632118	-0.7337683	0.3559083	0.7301931	1.670473	0.662	7.73E-06		
S X	-0.4017954	1.631868	-0.1140526	0.2937646	-0.2877428	1.658099	0.862	2.87E-06		
S Y	5.59E-09	1.632124	-0.6803671	0.7255389	0.6803672	1.786123	0.703	5.41E-06		
V W *	-1.098612	1.629185	0.5165546	7822.392	-1.615167	7822.392	1	1.33E-06		

eTable 9D Design-by-treatment model and loop inconsistency for all primary outcomes

Inconsistency model	chi^2	*p* value of Prob>chi^2
Primary outcome: overall asthma-COPD overlap syndrome risk		
design-by-treatment	15.66	0.9591
loop inconsistency	8.43	0.8659
Primary outcome: subgroup of asthma risk		
design-by-treatment	13.82	0.5395
loop inconsistency	6.24	0.2832
Primary outcome: subgroup of COPD risk		
design-by-treatment	8.37	0.9725
loop inconsistency	3.87	0.9737

Abbreviation: 95%CIs: 95% confidence intervals; DPP4 inhibitor: dipeptidyl-peptidase 4 inhibitor; GLP-1 agonist: glucagon-like peptide-1 agonist; NMA: network meta-analysis; RR: risk ratio; RCT: randomised controlled trial; SGLT2 inhibitor: sodium–glucose cotransporter 2 inhibitor

eTable 10 GRADE for primary outcome: overall asthma-COPD overlap syndrome risk

	Comparison	Study limitations	Imprecision	Inconsistency	Indirectness	Publication bias	GRADE
1	Albiglutide:Alogliptin	No downgrade	No downgrade	No downgrade	No downgrade	No downgrade	MODERATE
2	Albiglutide:Bexagliflozin	No downgrade	No downgrade	No downgrade	No downgrade	No downgrade	MODERATE
3	Albiglutide:Canagliflozin	No downgrade	No downgrade	No downgrade	No downgrade	No downgrade	MODERATE
4	Albiglutide:Dapagliflozin	No downgrade	No downgrade	No downgrade	No downgrade	No downgrade	MODERATE
5	Albiglutide:Dapagliflozin_Saxagliptin	No downgrade	Downgrade because the opposite limit exceed	No downgrade	No downgrade	No downgrade	LOW
6	Albiglutide:Dulaglutide	No downgrade	No downgrade	No downgrade	No downgrade	No downgrade	MODERATE
7	Albiglutide:Efpeglenatide	No downgrade	No downgrade	No downgrade	No downgrade	No downgrade	MODERATE
8	Albiglutide:Empagliflozin	No downgrade	No downgrade	No downgrade	No downgrade	No downgrade	MODERATE
9	Albiglutide:Ertugliflozin	No downgrade	No downgrade	No downgrade	No downgrade	No downgrade	MODERATE
10	Albiglutide:Ertugliflozin_Sitagliptin	No downgrade	No downgrade	No downgrade	No downgrade	No downgrade	MODERATE
11	Albiglutide:Exenatide	No downgrade	No downgrade	No downgrade	No downgrade	No downgrade	MODERATE
12	Albiglutide:Inject_semaglutide	No downgrade	No downgrade	No downgrade	No downgrade	No downgrade	MODERATE
13	Albiglutide:Linagliptin	No downgrade	No downgrade	No downgrade	No downgrade	No downgrade	MODERATE
14	Albiglutide:Liraglutide	No downgrade	No downgrade	No downgrade	No downgrade	No downgrade	MODERATE
15	Albiglutide:Lixisenatide	No downgrade	No downgrade	No downgrade	No downgrade	No downgrade	MODERATE
16	Albiglutide:Omarigliptin	No downgrade	No downgrade	No downgrade	No downgrade	No downgrade	MODERATE
17	Albiglutide:Oral_semaglutide	No downgrade	No downgrade	No downgrade	No downgrade	No downgrade	MODERATE
18	Albiglutide:Placebo_or_Control	No downgrade	No downgrade	No downgrade	No downgrade	No downgrade	MODERATE
19	Albiglutide:Retatrutide	No downgrade	Downgrade because the opposite limit exceed	No downgrade	No downgrade	No downgrade	LOW
20	Albiglutide:Saxagliptin	No downgrade	No downgrade	No downgrade	No downgrade	No downgrade	MODERATE

21	Albiglutide:Sitagliptin	Downgrade because risk of bias	No downgrade	No downgrade	No downgrade	No downgrade	LOW
22	Albiglutide:Sotagliflozin	No downgrade	No downgrade	No downgrade	No downgrade	No downgrade	MODERATE
23	Albiglutide:Teneligliptin	No downgrade	No downgrade	No downgrade	No downgrade	No downgrade	MODERATE
24	Albiglutide:Tirzepatide	No downgrade	No downgrade	No downgrade	No downgrade	No downgrade	MODERATE
25	Albiglutide:Vildagliptin	No downgrade	No downgrade	No downgrade	No downgrade	No downgrade	MODERATE
26	Alogliptin:Bexagliflozin	No downgrade	No downgrade	No downgrade	No downgrade	No downgrade	MODERATE
27	Alogliptin:Canagliflozin	No downgrade	No downgrade	No downgrade	No downgrade	No downgrade	MODERATE
28	Alogliptin:Dapagliflozin	No downgrade	No downgrade	No downgrade	No downgrade	No downgrade	MODERATE
29	Alogliptin:Dapagliflozin_Saxagliptin	No downgrade	No downgrade	No downgrade	No downgrade	No downgrade	MODERATE
30	Alogliptin:Dulaglutide	No downgrade	No downgrade	No downgrade	No downgrade	No downgrade	MODERATE
31	Alogliptin:Efpeglenatide	No downgrade	No downgrade	No downgrade	No downgrade	No downgrade	MODERATE
32	Alogliptin:Empagliflozin	No downgrade	No downgrade	No downgrade	No downgrade	No downgrade	MODERATE
33	Alogliptin:Ertugliflozin	No downgrade	No downgrade	No downgrade	No downgrade	No downgrade	MODERATE
34	Alogliptin:Ertugliflozin_Sitagliptin	No downgrade	No downgrade	No downgrade	No downgrade	No downgrade	MODERATE
35	Alogliptin:Exenatide	No downgrade	No downgrade	No downgrade	No downgrade	No downgrade	MODERATE
36	Alogliptin:Inject_semaglutide	No downgrade	No downgrade	No downgrade	No downgrade	No downgrade	MODERATE
37	Alogliptin:Linagliptin	No downgrade	No downgrade	No downgrade	No downgrade	No downgrade	MODERATE
38	Alogliptin:Liraglutide	No downgrade	No downgrade	No downgrade	No downgrade	No downgrade	MODERATE
39	Alogliptin:Lixisenatide	No downgrade	No downgrade	No downgrade	No downgrade	No downgrade	MODERATE
40	Alogliptin:Omarigliptin	No downgrade	No downgrade	No downgrade	No downgrade	No downgrade	MODERATE
41	Alogliptin:Oral_semaglutide	No downgrade	No downgrade	No downgrade	No downgrade	No downgrade	MODERATE
42	Alogliptin:Placebo_or_Control	Downgrade because risk of bias	No downgrade	No downgrade	No downgrade	No downgrade	LOW

43	Alogliptin:Retatrutide	No downgrade	Downgrade because the opposite limit exceed	No downgrade	No downgrade	No downgrade	LOW
44	Alogliptin:Saxagliptin	No downgrade	No downgrade	No downgrade	No downgrade	No downgrade	MODERATE
45	Alogliptin:Sitagliptin	No downgrade	No downgrade	No downgrade	No downgrade	No downgrade	MODERATE
46	Alogliptin:Sotagliflozin	No downgrade	No downgrade	No downgrade	No downgrade	No downgrade	MODERATE
47	Alogliptin:Teneligliptin	No downgrade	No downgrade	No downgrade	No downgrade	No downgrade	MODERATE
48	Alogliptin:Tirzepatide	No downgrade	No downgrade	No downgrade	No downgrade	No downgrade	MODERATE
49	Alogliptin:Vildagliptin	No downgrade	No downgrade	No downgrade	No downgrade	No downgrade	MODERATE
50	Bexagliflozin:Canagliflozin	No downgrade	No downgrade	No downgrade	No downgrade	No downgrade	MODERATE
51	Bexagliflozin:Dapagliflozin	No downgrade	No downgrade	No downgrade	No downgrade	No downgrade	MODERATE
52	Bexagliflozin:Dapagliflozin_Saxagliptin	No downgrade	Downgrade because the opposite limit exceed	No downgrade	No downgrade	No downgrade	LOW
53	Bexagliflozin:Dulaglutide	No downgrade	No downgrade	No downgrade	No downgrade	No downgrade	MODERATE
54	Bexagliflozin:Efpeglenatide	No downgrade	No downgrade	No downgrade	No downgrade	No downgrade	MODERATE
55	Bexagliflozin:Empagliflozin	No downgrade	No downgrade	No downgrade	No downgrade	No downgrade	MODERATE
56	Bexagliflozin:Ertugliflozin	No downgrade	No downgrade	No downgrade	No downgrade	No downgrade	MODERATE
57	Bexagliflozin:Ertugliflozin_Sitagliptin	No downgrade	No downgrade	No downgrade	No downgrade	No downgrade	MODERATE
58	Bexagliflozin:Exenatide	No downgrade	No downgrade	No downgrade	No downgrade	No downgrade	MODERATE
59	Bexagliflozin:Inject_semaglutide	No downgrade	No downgrade	No downgrade	No downgrade	No downgrade	MODERATE
60	Bexagliflozin:Linagliptin	No downgrade	No downgrade	No downgrade	No downgrade	No downgrade	MODERATE
61	Bexagliflozin:Liraglutide	No downgrade	No downgrade	No downgrade	No downgrade	No downgrade	MODERATE
62	Bexagliflozin:Lixisenatide	No downgrade	No downgrade	No downgrade	No downgrade	No downgrade	MODERATE
63	Bexagliflozin:Omarigliptin	No downgrade	No downgrade	No downgrade	No downgrade	No downgrade	MODERATE
64	Bexagliflozin:Oral_semaglutide	No downgrade	No downgrade	No downgrade	No downgrade	No downgrade	MODERATE

65	Bexagliflozin:Placebo_or_Control	Downgrade because risk of bias	No downgrade	No downgrade	No downgrade	No downgrade	LOW
66	Bexagliflozin:Retatrutide	No downgrade	Downgrade because the opposite limit exceed	No downgrade	No downgrade	No downgrade	LOW
67	Bexagliflozin:Saxagliptin	No downgrade	No downgrade	No downgrade	No downgrade	No downgrade	MODERATE
68	Bexagliflozin:Sitagliptin	No downgrade	No downgrade	No downgrade	No downgrade	No downgrade	MODERATE
69	Bexagliflozin:Sotagliflozin	No downgrade	No downgrade	No downgrade	No downgrade	No downgrade	MODERATE
70	Bexagliflozin:Teneligliptin	No downgrade	No downgrade	No downgrade	No downgrade	No downgrade	MODERATE
71	Bexagliflozin:Tirzepatide	No downgrade	No downgrade	No downgrade	No downgrade	No downgrade	MODERATE
72	Bexagliflozin:Vildagliptin	No downgrade	No downgrade	No downgrade	No downgrade	No downgrade	MODERATE
73	Canagliflozin:Dapagliflozin	No downgrade	No downgrade	No downgrade	No downgrade	No downgrade	MODERATE
74	Canagliflozin:Dapagliflozin_Saxagliptin	No downgrade	No downgrade	No downgrade	No downgrade	No downgrade	MODERATE
75	Canagliflozin:Dulaglutide	No downgrade	No downgrade	No downgrade	No downgrade	No downgrade	MODERATE
76	Canagliflozin:Efpeglenatide	No downgrade	No downgrade	No downgrade	No downgrade	No downgrade	MODERATE
77	Canagliflozin:Empagliflozin	No downgrade	No downgrade	No downgrade	No downgrade	No downgrade	MODERATE
78	Canagliflozin:Ertugliflozin	No downgrade	No downgrade	No downgrade	No downgrade	No downgrade	MODERATE
79	Canagliflozin:Ertugliflozin_Sitagliptin	No downgrade	Downgrade because the opposite limit exceed	No downgrade	No downgrade	No downgrade	LOW
80	Canagliflozin:Exenatide	No downgrade	No downgrade	No downgrade	No downgrade	No downgrade	MODERATE
81	Canagliflozin:Inject_semaglutide	No downgrade	No downgrade	No downgrade	No downgrade	No downgrade	MODERATE
82	Canagliflozin:Linagliptin	No downgrade	No downgrade	No downgrade	No downgrade	No downgrade	MODERATE
83	Canagliflozin:Liraglutide	No downgrade	No downgrade	No downgrade	No downgrade	No downgrade	MODERATE
84	Canagliflozin:Lixisenatide	No downgrade	No downgrade	No downgrade	No downgrade	No downgrade	MODERATE
85	Canagliflozin:Omarigliptin	No downgrade	No downgrade	No downgrade	No downgrade	No downgrade	MODERATE
86	Canagliflozin:Oral_semaglutide	No downgrade	Upgrade due to large effect size	No downgrade	No downgrade	No downgrade	HIGH

87	Canagliflozin:Placebo_or_Control	No downgrade	Upgrade due to large effect size	No downgrade	No downgrade	No downgrade	HIGH
88	Canagliflozin:Retatrutide	No downgrade	Downgrade because the opposite limit exceed	No downgrade	No downgrade	No downgrade	LOW
89	Canagliflozin:Saxagliptin	No downgrade	Upgrade due to large effect size	No downgrade	No downgrade	No downgrade	HIGH
90	Canagliflozin:Sitagliptin	No downgrade	No downgrade	No downgrade	No downgrade	No downgrade	MODERATE
91	Canagliflozin:Sotagliflozin	No downgrade	No downgrade	No downgrade	No downgrade	No downgrade	MODERATE
92	Canagliflozin:Teneligliptin	No downgrade	No downgrade	No downgrade	No downgrade	No downgrade	MODERATE
93	Canagliflozin:Tirzepatide	No downgrade	No downgrade	No downgrade	No downgrade	No downgrade	MODERATE
94	Canagliflozin:Vildagliptin	No downgrade	No downgrade	No downgrade	No downgrade	No downgrade	MODERATE
95	Dapagliflozin:Dapagliflozin_Saxagliptin	No downgrade	No downgrade	No downgrade	No downgrade	No downgrade	MODERATE
96	Dapagliflozin:Dulaglutide	No downgrade	No downgrade	No downgrade	No downgrade	No downgrade	MODERATE
97	Dapagliflozin:Efpeglenatide	No downgrade	No downgrade	No downgrade	No downgrade	No downgrade	MODERATE
98	Dapagliflozin:Empagliflozin	No downgrade	No downgrade	No downgrade	No downgrade	No downgrade	MODERATE
99	Dapagliflozin:Ertugliflozin	No downgrade	No downgrade	No downgrade	No downgrade	No downgrade	MODERATE
100	Dapagliflozin:Ertugliflozin_Sitagliptin	No downgrade	No downgrade	No downgrade	No downgrade	No downgrade	MODERATE
101	Dapagliflozin:Exenatide	No downgrade	No downgrade	No downgrade	No downgrade	No downgrade	MODERATE
102	Dapagliflozin:Inject_semaglutide	No downgrade	No downgrade	No downgrade	No downgrade	No downgrade	MODERATE
103	Dapagliflozin:Linagliptin	No downgrade	No downgrade	No downgrade	No downgrade	No downgrade	MODERATE
104	Dapagliflozin:Liraglutide	No downgrade	No downgrade	No downgrade	No downgrade	No downgrade	MODERATE
105	Dapagliflozin:Lixisenatide	No downgrade	No downgrade	No downgrade	No downgrade	No downgrade	MODERATE
106	Dapagliflozin:Omarigliptin	No downgrade	No downgrade	No downgrade	No downgrade	No downgrade	MODERATE
107	Dapagliflozin:Oral_semaglutide	No downgrade	Upgrade due to large effect size	No downgrade	No downgrade	No downgrade	HIGH
108	Dapagliflozin:Placebo_or_Control	No downgrade	Upgrade due to large effect size	No downgrade	No downgrade	No downgrade	HIGH

109	Dapagliflozin:Retatrutide	No downgrade	Downgrade because the opposite limit exceed	No downgrade	No downgrade	No downgrade	LOW
110	Dapagliflozin:Saxagliptin	No downgrade	Upgrade due to large effect size	No downgrade	No downgrade	No downgrade	HIGH
111	Dapagliflozin:Sitagliptin	No downgrade	No downgrade	No downgrade	No downgrade	No downgrade	MODERATE
112	Dapagliflozin:Sotagliflozin	No downgrade	No downgrade	No downgrade	No downgrade	No downgrade	MODERATE
113	Dapagliflozin:Teneligliptin	No downgrade	No downgrade	No downgrade	No downgrade	No downgrade	MODERATE
114	Dapagliflozin:Tirzepatide	No downgrade	No downgrade	No downgrade	No downgrade	No downgrade	MODERATE
115	Dapagliflozin:Vildagliptin	No downgrade	No downgrade	No downgrade	No downgrade	No downgrade	MODERATE
116	Dapagliflozin_Saxagliptin:Dulaglutide	No downgrade	No downgrade	No downgrade	No downgrade	No downgrade	MODERATE
117	Dapagliflozin_Saxagliptin:Efpeglenatide	No downgrade	No downgrade	No downgrade	No downgrade	No downgrade	MODERATE
118	Dapagliflozin_Saxagliptin:Empagliflozin	No downgrade	No downgrade	No downgrade	No downgrade	No downgrade	MODERATE
119	Dapagliflozin_Saxagliptin:Ertugliflozin	No downgrade	No downgrade	No downgrade	No downgrade	No downgrade	MODERATE
120	Dapagliflozin_Saxagliptin:Ertugliflozin_Sitagliptin	No downgrade	Downgrade because the opposite limit exceed	No downgrade	No downgrade	No downgrade	LOW
121	Dapagliflozin_Saxagliptin:Exenatide	No downgrade	No downgrade	No downgrade	No downgrade	No downgrade	MODERATE
122	Dapagliflozin_Saxagliptin:Inject_semaglutide	No downgrade	No downgrade	No downgrade	No downgrade	No downgrade	MODERATE
123	Dapagliflozin_Saxagliptin:Linagliptin	No downgrade	Downgrade because the opposite limit exceed	No downgrade	No downgrade	No downgrade	LOW
124	Dapagliflozin_Saxagliptin:Liraglutide	No downgrade	No downgrade	No downgrade	No downgrade	No downgrade	MODERATE
125	Dapagliflozin_Saxagliptin:Lixisenatide	No downgrade	No downgrade	No downgrade	No downgrade	No downgrade	MODERATE
126	Dapagliflozin_Saxagliptin:Omarigliptin	No downgrade	No downgrade	No downgrade	No downgrade	No downgrade	MODERATE
127	Dapagliflozin_Saxagliptin:Oral_semaglutide	No downgrade	No downgrade	No downgrade	No downgrade	No downgrade	MODERATE
128	Dapagliflozin_Saxagliptin:Placebo_or_Control	No downgrade	No downgrade	No downgrade	No downgrade	No downgrade	MODERATE
129	Dapagliflozin_Saxagliptin:Retatrutide	No downgrade	Downgrade because the opposite limit exceed	No downgrade	No downgrade	No downgrade	LOW
130	Dapagliflozin_Saxagliptin:Saxagliptin	No downgrade	No downgrade	No downgrade	No downgrade	No downgrade	MODERATE

131	Dapagliflozin_Saxagliptin:Sitagliptin	No downgrade	No downgrade	No downgrade	No downgrade	No downgrade	MODERATE
132	Dapagliflozin_Saxagliptin:Sotagliflozin	No downgrade	No downgrade	No downgrade	No downgrade	No downgrade	MODERATE
133	Dapagliflozin_Saxagliptin:Teneligliptin	No downgrade	No downgrade	No downgrade	No downgrade	No downgrade	MODERATE
134	Dapagliflozin_Saxagliptin:Tirzepatide	No downgrade	No downgrade	No downgrade	No downgrade	No downgrade	MODERATE
135	Dapagliflozin_Saxagliptin:Vildagliptin	No downgrade	Downgrade because the opposite limit exceed	No downgrade	No downgrade	No downgrade	LOW
136	Dulaglutide:Efpeglenatide	No downgrade	No downgrade	No downgrade	No downgrade	No downgrade	MODERATE
137	Dulaglutide:Empagliflozin	No downgrade	No downgrade	No downgrade	No downgrade	No downgrade	MODERATE
138	Dulaglutide:Ertugliflozin	No downgrade	No downgrade	No downgrade	No downgrade	No downgrade	MODERATE
139	Dulaglutide:Ertugliflozin_Sitagliptin	No downgrade	No downgrade	No downgrade	No downgrade	No downgrade	MODERATE
140	Dulaglutide:Exenatide	No downgrade	No downgrade	No downgrade	No downgrade	No downgrade	MODERATE
141	Dulaglutide:Inject_semaglutide	No downgrade	No downgrade	No downgrade	No downgrade	No downgrade	MODERATE
142	Dulaglutide:Linagliptin	No downgrade	No downgrade	No downgrade	No downgrade	No downgrade	MODERATE
143	Dulaglutide:Liraglutide	No downgrade	No downgrade	No downgrade	No downgrade	No downgrade	MODERATE
144	Dulaglutide:Lixisenatide	No downgrade	No downgrade	No downgrade	No downgrade	No downgrade	MODERATE
145	Dulaglutide:Omarigliptin	No downgrade	No downgrade	No downgrade	No downgrade	No downgrade	MODERATE
146	Dulaglutide:Oral_semaglutide	No downgrade	Upgrade due to large effect size	No downgrade	No downgrade	No downgrade	HIGH
147	Dulaglutide:Placebo_or_Control	No downgrade	No downgrade	No downgrade	No downgrade	No downgrade	MODERATE
148	Dulaglutide:Retatrutide	No downgrade	Downgrade because the opposite limit exceed	No downgrade	No downgrade	No downgrade	LOW
149	Dulaglutide:Saxagliptin	No downgrade	Upgrade due to large effect size	No downgrade	No downgrade	No downgrade	HIGH
150	Dulaglutide:Sitagliptin	No downgrade	No downgrade	No downgrade	No downgrade	No downgrade	MODERATE
151	Dulaglutide:Sotagliflozin	No downgrade	No downgrade	No downgrade	No downgrade	No downgrade	MODERATE
152	Dulaglutide:Teneligliptin	No downgrade	No downgrade	No downgrade	No downgrade	No downgrade	MODERATE

153	Dulaglutide:Tirzepatide	No downgrade	No downgrade	No downgrade	No downgrade	No downgrade	MODERATE
154	Dulaglutide:Vildagliptin	No downgrade	No downgrade	No downgrade	No downgrade	No downgrade	MODERATE
155	Efpeglenatide:Empagliflozin	No downgrade	No downgrade	No downgrade	No downgrade	No downgrade	MODERATE
156	Efpeglenatide:Ertugliflozin	No downgrade	No downgrade	No downgrade	No downgrade	No downgrade	MODERATE
157	Efpeglenatide:Ertugliflozin_Sitagliptin	No downgrade	No downgrade	No downgrade	No downgrade	No downgrade	MODERATE
158	Efpeglenatide:Exenatide	No downgrade	No downgrade	No downgrade	No downgrade	No downgrade	MODERATE
159	Efpeglenatide:Inject_semaglutide	No downgrade	No downgrade	No downgrade	No downgrade	No downgrade	MODERATE
160	Efpeglenatide:Linagliptin	No downgrade	No downgrade	No downgrade	No downgrade	No downgrade	MODERATE
161	Efpeglenatide:Liraglutide	No downgrade	No downgrade	No downgrade	No downgrade	No downgrade	MODERATE
162	Efpeglenatide:Lixisenatide	No downgrade	No downgrade	No downgrade	No downgrade	No downgrade	MODERATE
163	Efpeglenatide:Omarigliptin	No downgrade	No downgrade	No downgrade	No downgrade	No downgrade	MODERATE
164	Efpeglenatide:Oral_semaglutide	No downgrade	No downgrade	No downgrade	No downgrade	No downgrade	MODERATE
165	Efpeglenatide:Placebo_or_Control	No downgrade	No downgrade	No downgrade	No downgrade	No downgrade	MODERATE
166	Efpeglenatide:Retatrutide	No downgrade	Downgrade because the opposite limit exceed	No downgrade	No downgrade	No downgrade	LOW
167	Efpeglenatide:Saxagliptin	No downgrade	No downgrade	No downgrade	No downgrade	No downgrade	MODERATE
168	Efpeglenatide:Sitagliptin	No downgrade	No downgrade	No downgrade	No downgrade	No downgrade	MODERATE
169	Efpeglenatide:Sotagliflozin	No downgrade	No downgrade	No downgrade	No downgrade	No downgrade	MODERATE
170	Efpeglenatide:Teneligliptin	No downgrade	No downgrade	No downgrade	No downgrade	No downgrade	MODERATE
171	Efpeglenatide:Tirzepatide	No downgrade	No downgrade	No downgrade	No downgrade	No downgrade	MODERATE
172	Efpeglenatide:Vildagliptin	No downgrade	No downgrade	No downgrade	No downgrade	No downgrade	MODERATE
173	Empagliflozin:Ertugliflozin	No downgrade	No downgrade	No downgrade	No downgrade	No downgrade	MODERATE
174	Empagliflozin:Ertugliflozin_Sitagliptin	No downgrade	No downgrade	No downgrade	No downgrade	No downgrade	MODERATE

175	Empagliflozin:Exenatide	No downgrade	No downgrade	No downgrade	No downgrade	No downgrade	MODERATE
176	Empagliflozin:Inject_semaglutide	No downgrade	No downgrade	No downgrade	No downgrade	No downgrade	MODERATE
177	Empagliflozin:Linagliptin	No downgrade	No downgrade	No downgrade	No downgrade	No downgrade	MODERATE
178	Empagliflozin:Liraglutide	No downgrade	No downgrade	No downgrade	No downgrade	No downgrade	MODERATE
179	Empagliflozin:Lixisenatide	No downgrade	No downgrade	No downgrade	No downgrade	No downgrade	MODERATE
180	Empagliflozin:Omarigliptin	No downgrade	No downgrade	No downgrade	No downgrade	No downgrade	MODERATE
181	Empagliflozin:Oral_semaglutide	No downgrade	Upgrade due to large effect size	No downgrade	No downgrade	No downgrade	HIGH
182	Empagliflozin:Placebo_or_Control	No downgrade	Upgrade due to large effect size	No downgrade	No downgrade	No downgrade	HIGH
183	Empagliflozin:Retatrutide	No downgrade	Downgrade because the opposite limit exceed	No downgrade	No downgrade	No downgrade	LOW
184	Empagliflozin:Saxagliptin	No downgrade	Upgrade due to large effect size	No downgrade	No downgrade	No downgrade	HIGH
185	Empagliflozin:Sitagliptin	Downgrade because risk of bias	No downgrade	No downgrade	No downgrade	No downgrade	LOW
186	Empagliflozin:Sotagliflozin	No downgrade	No downgrade	No downgrade	No downgrade	No downgrade	MODERATE
187	Empagliflozin:Teneligliptin	No downgrade	No downgrade	No downgrade	No downgrade	No downgrade	MODERATE
188	Empagliflozin:Tirzepatide	No downgrade	No downgrade	No downgrade	No downgrade	No downgrade	MODERATE
189	Empagliflozin:Vildagliptin	No downgrade	No downgrade	No downgrade	No downgrade	No downgrade	MODERATE
190	Ertugliflozin:Ertugliflozin_Sitagliptin	No downgrade	No downgrade	No downgrade	No downgrade	No downgrade	MODERATE
191	Ertugliflozin:Exenatide	No downgrade	No downgrade	No downgrade	No downgrade	No downgrade	MODERATE
192	Ertugliflozin:Inject_semaglutide	No downgrade	No downgrade	No downgrade	No downgrade	No downgrade	MODERATE
193	Ertugliflozin:Linagliptin	No downgrade	No downgrade	No downgrade	No downgrade	No downgrade	MODERATE
194	Ertugliflozin:Liraglutide	No downgrade	No downgrade	No downgrade	No downgrade	No downgrade	MODERATE
195	Ertugliflozin:Lixisenatide	No downgrade	No downgrade	No downgrade	No downgrade	No downgrade	MODERATE
196	Ertugliflozin:Omarigliptin	No downgrade	No downgrade	No downgrade	No downgrade	No downgrade	MODERATE

197	Ertugliflozin:Oral_semaglutide	No downgrade	No downgrade	No downgrade	No downgrade	No downgrade	MODERATE
198	Ertugliflozin:Placebo_or_Control	No downgrade	No downgrade	No downgrade	No downgrade	No downgrade	MODERATE
199	Ertugliflozin:Retatrutide	No downgrade	Downgrade because the opposite limit exceed	No downgrade	No downgrade	No downgrade	LOW
200	Ertugliflozin:Saxagliptin	No downgrade	No downgrade	No downgrade	No downgrade	No downgrade	MODERATE
201	Ertugliflozin:Sitagliptin	No downgrade	No downgrade	No downgrade	No downgrade	No downgrade	MODERATE
202	Ertugliflozin:Sotagliflozin	No downgrade	No downgrade	No downgrade	No downgrade	No downgrade	MODERATE
203	Ertugliflozin:Teneligliptin	No downgrade	No downgrade	No downgrade	No downgrade	No downgrade	MODERATE
204	Ertugliflozin:Tirzepatide	No downgrade	No downgrade	No downgrade	No downgrade	No downgrade	MODERATE
205	Ertugliflozin:Vildagliptin	No downgrade	No downgrade	No downgrade	No downgrade	No downgrade	MODERATE
206	Ertugliflozin_Sitagliptin:Exenatide	No downgrade	No downgrade	No downgrade	No downgrade	No downgrade	MODERATE
207	Ertugliflozin_Sitagliptin:Inject_semaglutide	No downgrade	No downgrade	No downgrade	No downgrade	No downgrade	MODERATE
208	Ertugliflozin_Sitagliptin:Linagliptin	No downgrade	No downgrade	No downgrade	No downgrade	No downgrade	MODERATE
209	Ertugliflozin_Sitagliptin:Liraglutide	No downgrade	No downgrade	No downgrade	No downgrade	No downgrade	MODERATE
210	Ertugliflozin_Sitagliptin:Lixisenatide	No downgrade	No downgrade	No downgrade	No downgrade	No downgrade	MODERATE
211	Ertugliflozin_Sitagliptin:Omarigliptin	No downgrade	No downgrade	No downgrade	No downgrade	No downgrade	MODERATE
212	Ertugliflozin_Sitagliptin:Oral_semaglutide	No downgrade	No downgrade	No downgrade	No downgrade	No downgrade	MODERATE
213	Ertugliflozin_Sitagliptin:Placebo_or_Control	No downgrade	No downgrade	No downgrade	No downgrade	No downgrade	MODERATE
214	Ertugliflozin_Sitagliptin:Retatrutide	No downgrade	Downgrade because the opposite limit exceed	No downgrade	No downgrade	No downgrade	LOW
215	Ertugliflozin_Sitagliptin:Saxagliptin	No downgrade	No downgrade	No downgrade	No downgrade	No downgrade	MODERATE
216	Ertugliflozin_Sitagliptin:Sitagliptin	No downgrade	No downgrade	No downgrade	No downgrade	No downgrade	MODERATE
217	Ertugliflozin_Sitagliptin:Sotagliflozin	No downgrade	Downgrade because the opposite limit exceed	No downgrade	No downgrade	No downgrade	LOW
218	Ertugliflozin_Sitagliptin:Teneligliptin	No downgrade	Downgrade because the opposite limit exceed	No downgrade	No downgrade	No downgrade	LOW

219	Ertugliflozin_Sitagliptin:Tirzepatide	No downgrade	Downgrade because the opposite limit exceed	No downgrade	No downgrade	No downgrade	LOW
220	Ertugliflozin_Sitagliptin:Vildagliptin	No downgrade	No downgrade	No downgrade	No downgrade	No downgrade	MODERATE
221	Exenatide:Inject_semaglutide	No downgrade	Upgrade due to large effect size	No downgrade	No downgrade	No downgrade	HIGH
222	Exenatide:Linagliptin	No downgrade	No downgrade	No downgrade	No downgrade	No downgrade	MODERATE
223	Exenatide:Liraglutide	Downgrade because risk of bias	No downgrade	No downgrade	No downgrade	No downgrade	LOW
224	Exenatide:Lixisenatide	No downgrade	No downgrade	No downgrade	No downgrade	No downgrade	MODERATE
225	Exenatide:Omarigliptin	No downgrade	No downgrade	No downgrade	No downgrade	No downgrade	MODERATE
226	Exenatide:Oral_semaglutide	No downgrade	No downgrade	No downgrade	No downgrade	No downgrade	MODERATE
227	Exenatide:Placebo_or_Control	No downgrade	No downgrade	No downgrade	No downgrade	No downgrade	MODERATE
228	Exenatide:Retatrutide	No downgrade	Downgrade because the opposite limit exceed	No downgrade	No downgrade	No downgrade	LOW
229	Exenatide:Saxagliptin	No downgrade	No downgrade	No downgrade	No downgrade	No downgrade	MODERATE
230	Exenatide:Sitagliptin	No downgrade	No downgrade	No downgrade	No downgrade	No downgrade	MODERATE
231	Exenatide:Sotagliflozin	No downgrade	No downgrade	No downgrade	No downgrade	No downgrade	MODERATE
232	Exenatide:Teneligliptin	No downgrade	No downgrade	No downgrade	No downgrade	No downgrade	MODERATE
233	Exenatide:Tirzepatide	No downgrade	No downgrade	No downgrade	No downgrade	No downgrade	MODERATE
234	Exenatide:Vildagliptin	No downgrade	No downgrade	No downgrade	No downgrade	No downgrade	MODERATE
235	Inject_semaglutide:Linagliptin	No downgrade	No downgrade	No downgrade	No downgrade	No downgrade	MODERATE
236	Inject_semaglutide:Liraglutide	No downgrade	No downgrade	No downgrade	No downgrade	No downgrade	MODERATE
237	Inject_semaglutide:Lixisenatide	No downgrade	No downgrade	No downgrade	No downgrade	No downgrade	MODERATE
238	Inject_semaglutide:Omarigliptin	No downgrade	No downgrade	No downgrade	No downgrade	No downgrade	MODERATE
239	Inject_semaglutide:Oral_semaglutide	No downgrade	Upgrade due to large effect size	No downgrade	No downgrade	No downgrade	HIGH
240	Inject_semaglutide:Placebo_or_Control	No downgrade	Upgrade due to large effect size	No downgrade	No downgrade	No downgrade	HIGH

241	Inject_semaglutide:Retatrutide	No downgrade	Downgrade because the opposite limit exceed	No downgrade	No downgrade	No downgrade	LOW
242	Inject_semaglutide:Saxagliptin	No downgrade	Upgrade due to large effect size	No downgrade	No downgrade	No downgrade	HIGH
243	Inject_semaglutide:Sitagliptin	No downgrade	No downgrade	No downgrade	No downgrade	No downgrade	MODERATE
244	Inject_semaglutide:Sotagliflozin	No downgrade	No downgrade	No downgrade	No downgrade	No downgrade	MODERATE
245	Inject_semaglutide:Teneligliptin	No downgrade	No downgrade	No downgrade	No downgrade	No downgrade	MODERATE
246	Inject_semaglutide:Tirzepatide	No downgrade	No downgrade	No downgrade	No downgrade	No downgrade	MODERATE
247	Inject_semaglutide:Vildagliptin	No downgrade	No downgrade	No downgrade	No downgrade	No downgrade	MODERATE
248	Linagliptin:Liraglutide	No downgrade	No downgrade	No downgrade	No downgrade	No downgrade	MODERATE
249	Linagliptin:Lixisenatide	No downgrade	No downgrade	No downgrade	No downgrade	No downgrade	MODERATE
250	Linagliptin:Omarigliptin	No downgrade	No downgrade	No downgrade	No downgrade	No downgrade	MODERATE
251	Linagliptin:Oral_semaglutide	No downgrade	No downgrade	No downgrade	No downgrade	No downgrade	MODERATE
252	Linagliptin:Placebo_or_Control	No downgrade	No downgrade	No downgrade	No downgrade	No downgrade	MODERATE
253	Linagliptin:Retatrutide	No downgrade	Downgrade because the opposite limit exceed	No downgrade	No downgrade	No downgrade	LOW
254	Linagliptin:Saxagliptin	No downgrade	No downgrade	No downgrade	No downgrade	No downgrade	MODERATE
255	Linagliptin:Sitagliptin	No downgrade	No downgrade	No downgrade	No downgrade	No downgrade	MODERATE
256	Linagliptin:Sotagliflozin	No downgrade	No downgrade	No downgrade	No downgrade	No downgrade	MODERATE
257	Linagliptin:Teneligliptin	No downgrade	No downgrade	No downgrade	No downgrade	No downgrade	MODERATE
258	Linagliptin:Tirzepatide	No downgrade	No downgrade	No downgrade	No downgrade	No downgrade	MODERATE
259	Linagliptin:Vildagliptin	No downgrade	No downgrade	No downgrade	No downgrade	No downgrade	MODERATE
260	Liraglutide:Lixisenatide	No downgrade	No downgrade	No downgrade	No downgrade	No downgrade	MODERATE
261	Liraglutide:Omarigliptin	No downgrade	No downgrade	No downgrade	No downgrade	No downgrade	MODERATE
262	Liraglutide:Oral_semaglutide	No downgrade	No downgrade	No downgrade	No downgrade	No downgrade	MODERATE

263	Liraglutide:Placebo_or_Control	No downgrade	No downgrade	No downgrade	No downgrade	No downgrade	MODERATE
264	Liraglutide:Retatrutide	No downgrade	Downgrade because the opposite limit exceed	No downgrade	No downgrade	No downgrade	LOW
265	Liraglutide:Saxagliptin	No downgrade	Upgrade due to large effect size	No downgrade	No downgrade	No downgrade	HIGH
266	Liraglutide:Sitagliptin	Downgrade because risk of bias	No downgrade	No downgrade	No downgrade	No downgrade	LOW
267	Liraglutide:Sotagliflozin	No downgrade	No downgrade	No downgrade	No downgrade	No downgrade	MODERATE
268	Liraglutide:Teneligliptin	No downgrade	No downgrade	No downgrade	No downgrade	No downgrade	MODERATE
269	Liraglutide:Tirzepatide	No downgrade	No downgrade	No downgrade	No downgrade	No downgrade	MODERATE
270	Liraglutide:Vildagliptin	No downgrade	No downgrade	No downgrade	No downgrade	No downgrade	MODERATE
271	Lixisenatide:Omarigliptin	No downgrade	No downgrade	No downgrade	No downgrade	No downgrade	MODERATE
272	Lixisenatide:Oral_semaglutide	No downgrade	No downgrade	No downgrade	No downgrade	No downgrade	MODERATE
273	Lixisenatide:Placebo_or_Control	No downgrade	No downgrade	No downgrade	No downgrade	No downgrade	MODERATE
274	Lixisenatide:Retatrutide	No downgrade	Downgrade because the opposite limit exceed	No downgrade	No downgrade	No downgrade	LOW
275	Lixisenatide:Saxagliptin	No downgrade	No downgrade	No downgrade	No downgrade	No downgrade	MODERATE
276	Lixisenatide:Sitagliptin	No downgrade	No downgrade	No downgrade	No downgrade	No downgrade	MODERATE
277	Lixisenatide:Sotagliflozin	No downgrade	No downgrade	No downgrade	No downgrade	No downgrade	MODERATE
278	Lixisenatide:Teneligliptin	No downgrade	No downgrade	No downgrade	No downgrade	No downgrade	MODERATE
279	Lixisenatide:Tirzepatide	No downgrade	No downgrade	No downgrade	No downgrade	No downgrade	MODERATE
280	Lixisenatide:Vildagliptin	No downgrade	No downgrade	No downgrade	No downgrade	No downgrade	MODERATE
281	Omarigliptin:Oral_semaglutide	No downgrade	No downgrade	No downgrade	No downgrade	No downgrade	MODERATE
282	Omarigliptin:Placebo_or_Control	No downgrade	No downgrade	No downgrade	No downgrade	No downgrade	MODERATE
283	Omarigliptin:Retatrutide	No downgrade	Downgrade because the opposite limit exceed	No downgrade	No downgrade	No downgrade	LOW
284	Omarigliptin:Saxagliptin	No downgrade	No downgrade	No downgrade	No downgrade	No downgrade	MODERATE

285	Omarigliptin:Sitagliptin	No downgrade	No downgrade	No downgrade	No downgrade	No downgrade	MODERATE
286	Omarigliptin:Sotagliflozin	No downgrade	No downgrade	No downgrade	No downgrade	No downgrade	MODERATE
287	Omarigliptin:Teneligliptin	No downgrade	No downgrade	No downgrade	No downgrade	No downgrade	MODERATE
288	Omarigliptin:Tirzepatide	No downgrade	No downgrade	No downgrade	No downgrade	No downgrade	MODERATE
289	Omarigliptin:Vildagliptin	No downgrade	No downgrade	No downgrade	No downgrade	No downgrade	MODERATE
290	Oral_semaglutide:Placebo_or_Control	No downgrade	No downgrade	No downgrade	No downgrade	No downgrade	MODERATE
291	Oral_semaglutide:Retatrutide	No downgrade	Downgrade because the opposite limit exceed	No downgrade	No downgrade	No downgrade	LOW
292	Oral_semaglutide:Saxagliptin	No downgrade	No downgrade	No downgrade	No downgrade	No downgrade	MODERATE
293	Oral_semaglutide:Sitagliptin	No downgrade	No downgrade	No downgrade	No downgrade	No downgrade	MODERATE
294	Oral_semaglutide:Sotagliflozin	No downgrade	Upgrade due to large effect size	No downgrade	No downgrade	No downgrade	HIGH
295	Oral_semaglutide:Teneligliptin	No downgrade	No downgrade	No downgrade	No downgrade	No downgrade	MODERATE
296	Oral_semaglutide:Tirzepatide	No downgrade	No downgrade	No downgrade	No downgrade	No downgrade	MODERATE
297	Oral_semaglutide:Vildagliptin	No downgrade	No downgrade	No downgrade	No downgrade	No downgrade	MODERATE
298	Retatrutide:Placebo_or_Control	No downgrade	Downgrade because the opposite limit exceed	No downgrade	No downgrade	No downgrade	LOW
299	Saxagliptin:Placebo_or_Control	No downgrade	No downgrade	No downgrade	No downgrade	No downgrade	MODERATE
300	Sitagliptin:Placebo_or_Control	Downgrade because risk of bias	No downgrade	No downgrade	No downgrade	No downgrade	LOW
301	Sotagliflozin:Placebo_or_Control	Downgrade because risk of bias	No downgrade	No downgrade	No downgrade	No downgrade	LOW
302	Teneligliptin:Placebo_or_Control	No downgrade	No downgrade	No downgrade	No downgrade	No downgrade	MODERATE
303	Tirzepatide:Placebo_or_Control	Downgrade because risk of bias	No downgrade	No downgrade	No downgrade	No downgrade	LOW
304	Vildagliptin:Placebo_or_Control	No downgrade	No downgrade	No downgrade	No downgrade	No downgrade	MODERATE
305	Retatrutide:Saxagliptin	No downgrade	Downgrade because the opposite limit exceed	No downgrade	No downgrade	No downgrade	LOW
306	Retatrutide:Sitagliptin	No downgrade	Downgrade because the opposite limit exceed	No downgrade	No downgrade	No downgrade	LOW

307	Retatrutide:Sotagliflozin	No downgrade	Downgrade because the opposite limit exceed	No downgrade	No downgrade	No downgrade	LOW
308	Retatrutide:Teneligliptin	No downgrade	Downgrade because the opposite limit exceed	No downgrade	No downgrade	No downgrade	LOW
309	Retatrutide:Tirzepatide	No downgrade	Downgrade because the opposite limit exceed	No downgrade	No downgrade	No downgrade	LOW
310	Retatrutide:Vildagliptin	No downgrade	Downgrade because the opposite limit exceed	No downgrade	No downgrade	No downgrade	LOW
311	Saxagliptin:Sitagliptin	No downgrade	No downgrade	No downgrade	No downgrade	No downgrade	MODERATE
312	Saxagliptin:Sotagliflozin	No downgrade	No downgrade	No downgrade	No downgrade	No downgrade	MODERATE
313	Saxagliptin:Teneligliptin	No downgrade	No downgrade	No downgrade	No downgrade	No downgrade	MODERATE
314	Saxagliptin:Tirzepatide	No downgrade	No downgrade	No downgrade	No downgrade	No downgrade	MODERATE
315	Saxagliptin:Vildagliptin	No downgrade	No downgrade	No downgrade	No downgrade	No downgrade	MODERATE
316	Sitagliptin:Sotagliflozin	No downgrade	No downgrade	No downgrade	No downgrade	No downgrade	MODERATE
317	Sitagliptin:Teneligliptin	No downgrade	No downgrade	No downgrade	No downgrade	No downgrade	MODERATE
318	Sitagliptin:Tirzepatide	No downgrade	No downgrade	No downgrade	No downgrade	No downgrade	MODERATE
319	Sitagliptin:Vildagliptin	No downgrade	No downgrade	No downgrade	No downgrade	No downgrade	MODERATE
320	Sotagliflozin:Teneligliptin	No downgrade	No downgrade	No downgrade	No downgrade	No downgrade	MODERATE
321	Sotagliflozin:Tirzepatide	No downgrade	No downgrade	No downgrade	No downgrade	No downgrade	MODERATE
322	Sotagliflozin:Vildagliptin	No downgrade	No downgrade	No downgrade	No downgrade	No downgrade	MODERATE
323	Teneligliptin:Tirzepatide	No downgrade	No downgrade	No downgrade	No downgrade	No downgrade	MODERATE
324	Teneligliptin:Vildagliptin	No downgrade	No downgrade	No downgrade	No downgrade	No downgrade	MODERATE
325	Tirzepatide:Vildagliptin	No downgrade	No downgrade	No downgrade	No downgrade	No downgrade	MODERATE

Abbreviation: 95%CIs: 95% confidence intervals; DPP4 inhibitor: dipeptidyl-peptidase 4 inhibitor; GLP-1 agonist: glucagon-like peptide-1 agonist; GRADE: grading of recommendations assessment, development, and evaluation; NMA: network meta-analysis; RR: risk ratio; RCT: randomised controlled trial; SGLT2 inhibitor: sodium–glucose cotransporter 2 inhibitor

Reference list of supplement tables:

1. Page MJ, McKenzie JE, Bossuyt PM, Boutron I, Hoffmann TC, Mulrow CD, et al. The PRISMA 2020 statement: an updated guideline for reporting systematic reviews. *Bmj* 2021; **372**: n71.
2. Figueira-Goncalves JM, Golpe R. Impact of Oral Antidiabetics Agents in the Prevention of COPD Exacerbations. *Arch Bronconeumol* 2023; **59**(7): 412-3.
3. Zhang MQ, Lin C, Cai XL, Jiao RY, Bai SZ, Li ZL, et al. The Association between GLP-1 Receptor-Based Agonists and the Incidence of Asthma in Patients with Type 2 Diabetes and/or Obesity: A Meta-Analysis. *Biomed Environ Sci* 2024; **37**(6): 607-16.
4. Wang Y, Zhou X. The relationship between use of SGLT2is and incidence of respiratory and infectious diseases and site-specific fractures: a meta-analysis based on 32 large RCTs. *Eur J Clin Pharmacol* 2024; **80**(4): 563-73.
5. Yu M, Wang R, Pei L, Zhang X, Wei J, Wen Y, et al. The relationship between the use of GLP-1 receptor agonists and the incidence of respiratory illness: a meta-analysis of randomized controlled trials. *Diabetol Metab Syndr* 2023; **15**(1): 164.
6. Qiu M, Zhao LM, Zhan ZL. Comprehensive Analysis of Adverse Events Associated With SGLT2is: A Meta-Analysis Involving Nine Large Randomized Trials. *Front Endocrinol (Lausanne)* 2021; **12**: 743807.
7. Wang A, Tang H, Zhang N, Feng X. Association between novel Glucose-Lowering drugs and risk of Asthma: A network Meta-Analysis of cardiorenal outcome trials. *Diabetes Res Clin Pract* 2022; **183**: 109080.
8. Yin DG, Qiu M, Duan XY. Association Between SGLT2is and Cardiovascular and Respiratory Diseases: A Meta-Analysis of Large Trials. *Front Pharmacol* 2021; **12**: 724405.
9. Shabil M, Patil J, Satapathy P, Gaidhane AM, Chennakesavulu K, Vadia N, et al. Effect of SGLT-2 inhibitors on COPD exacerbations in individuals with type 2 diabetes: A meta-analysis and Bayesian sensitivity analysis. *J Diabetes Investig* 2025; **16**(9): 1670-82.
10. Cooper DH, Akbarian N, Aaron SD, Luks V, Kendzerska T. Glucagon-like peptide 1 (GLP-1) receptor agonists and asthma and COPD

exacerbations in adults with diabetes: A systematic review. *Respir Med* 2025; **245**: 108197.
11. Satapathy P, Gaidhane AM, Vadia N, Menon SV, Chennakesavulu K, Panigrahi R, et al. Impact of SGLT-2i on COPD exacerbations in patients with type 2 diabetes mellitus: A systematic review and meta-analysis. *Diabetes Metab* 2025; **51**(4): 101646.
12. Shanmugavel Geetha H, Teo YX, Ravichandran S, Perkit NR, Gogtay M, Lal A, et al. Use of Sodium-glucose cotransporter 2 (SGLT 2) inhibitor is associated with reduced emergency room visits and hospitalizations in patients with Chronic obstructive pulmonary disease (COPD) and type 2 Diabetes Mellitus. *Respir Med* 2024; **234**: 107819.
13. Foer D, Strasser ZH, Cui J, Cahill KN, Boyce JA, Murphy SN, et al. Association of GLP-1 Receptor Agonists with Chronic Obstructive Pulmonary Disease Exacerbations among Patients with Type 2 Diabetes. *Am J Respir Crit Care Med* 2023; **208**(10): 1088-100.
14. Jeong HE, Park S, Noh Y, Bea S, Filion KB, Yu OHY, et al. Association of adverse respiratory events with sodium-glucose cotransporter 2 inhibitors versus dipeptidyl peptidase 4 inhibitors among patients with type 2 diabetes in South Korea: a nationwide cohort study. *BMC Med* 2023; **21**(1): 47.
15. Au PCM, Tan KCB, Lam DCL, Cheung BMY, Wong ICK, Kwok WC, et al. Association of Sodium-Glucose Cotransporter 2 Inhibitor vs Dipeptidyl Peptidase-4 Inhibitor Use With Risk of Incident Obstructive Airway Disease and Exacerbation Events Among Patients With Type 2 Diabetes in Hong Kong. *JAMA Netw Open* 2023; **6**(1): e2251177.
16. Pradhan R, Lu S, Yin H, Yu OHY, Ernst P, Suissa S, et al. Novel antihyperglycaemic drugs and prevention of chronic obstructive pulmonary disease exacerbations among patients with type 2 diabetes: population based cohort study. *Bmj* 2022; **379**: e071380.
17. Foer D, Beeler PE, Cui J, Karlson EW, Bates DW, Cahill KN. Asthma Exacerbations in Patients with Type 2 Diabetes and Asthma on Glucagon-like Peptide-1 Receptor Agonists. *Am J Respir Crit Care Med* 2021; **203**(7): 831-40.
18. Lai S, Feng Y, Li L, Zhao J, Wang Z, Wang Y. Association of GLP1RAs with risk of chronic obstructive pulmonary disease: evidence from drug target Mendelian randomization. *Diabetol Metab Syndr* 2025; **17**(1): 295.
19. Aronne LJ, Sattar N, Horn DB, Bays HE, Wharton S, Lin WY, et al. Continued Treatment With Tirzepatide for Maintenance of Weight Reduction in Adults With Obesity: The SURMOUNT-4 Randomized Clinical Trial. *Jama* 2024; **331**(1): 38-48.
20. Bliddal H, Bays H, Czernichow S, Udden Hemmingsson J, Hjelmesaeth J, Hoffmann Morville T, et al. Once-Weekly Semaglutide in Persons

with Obesity and Knee Osteoarthritis. *N Engl J Med* 2024; **391**(17): 1573-83.
21. Ji L, Agesen RM, Bain SC, Fu F, Gabery S, Geng J, et al. Efficacy and safety of oral semaglutide vs sitagliptin in a predominantly Chinese population with type 2 diabetes uncontrolled with metformin: PIONEER 12, a double-blind, Phase IIIa, randomised trial. *Diabetologia* 2024; **67**(9): 1800-16.
22. Lee BW, Cho YM, Kim SG, Ko SH, Lim S, Dahaoui A, et al. Efficacy and Safety of Once-Weekly Semaglutide Versus Once-Daily Sitagliptin as Metformin Add-on in a Korean Population with Type 2 Diabetes. *Diabetes Ther* 2024; **15**(2): 547-63.
23. McGowan BM, Bruun JM, Capehorn M, Pedersen SD, Pietilainen KH, Muniraju HAK, et al. Efficacy and safety of once-weekly semaglutide 2.4 mg versus placebo in people with obesity and prediabetes (STEP 10): a randomised, double-blind, placebo-controlled, multicentre phase 3 trial. *Lancet Diabetes Endocrinol* 2024; **12**(9): 631-42.
24. Mu Y, Bao X, Eliaschewitz FG, Hansen MR, Kim BT, Koroleva A, et al. Efficacy and safety of once weekly semaglutide 2.4 mg for weight management in a predominantly east Asian population with overweight or obesity (STEP 7): a double-blind, multicentre, randomised controlled trial. *Lancet Diabetes Endocrinol* 2024; **12**(3): 184-95.
25. Natale P, Tunnicliffe DJ, Toyama T, Palmer SC, Saglimbene VM, Ruospo M, et al. Sodium-glucose co-transporter protein 2 (SGLT2) inhibitors for people with chronic kidney disease and diabetes. *The Cochrane database of systematic reviews* 2024; **5**(5): CD015588.
26. Tuttle KR, Hauske SJ, Canziani ME, Caramori ML, Cherney D, Cronin L, et al. Efficacy and safety of aldosterone synthase inhibition with and without empagliflozin for chronic kidney disease: a randomised, controlled, phase 2 trial. *Lancet* 2024; **403**(10424): 379-90.
27. Zhao L, Cheng Z, Lu Y, Liu M, Chen H, Zhang M, et al. Tirzepatide for Weight Reduction in Chinese Adults With Obesity: The SURMOUNT-CN Randomized Clinical Trial. *Jama* 2024; **332**(7): 551-60.
28. Aroda VR, Frias JP, Ji L, Niemoeller E, Nguyen-Pascal ML, Denkel K, et al. Efficacy and safety of once-weekly efpeglenatide in people with suboptimally controlled type 2 diabetes: The AMPLITUDE-D, AMPLITUDE-L and AMPLITUDE-S randomized controlled trials. *Diabetes Obes Metab* 2023; **25**(8): 2084-95.
29. Buse JB, Nordahl Christensen H, Harty BJ, Mitchell J, Soule BP, Zacherle E, et al. Study design and baseline profile for adults with type 2 diabetes in the once-weekly subcutaneous SEmaglutide randomized PRAgmatic (SEPRA) trial. *BMJ Open Diabetes Res Care* 2023; **11**(3).

30. (SURPASS-CN-INS) EL. A Study of Tirzepatide (LY3298176) in Chinese Participants With Type 2 Diabetes (SURPASS-CN-INS) (NCT05691712). 2023. https://clinicaltrials.gov/study/NCT05691712?cond=NCT05691712&rank=1 (accessed 2025/9/1 2025).
31. Feng P, Sheng X, Ji Y, Urva S, Wang F, Miller S, et al. A Phase 1 Multiple Dose Study of Tirzepatide in Chinese Patients with Type 2 Diabetes. *Adv Ther* 2023; **40**(8): 3434-45.
32. Frias JP, Hsia S, Eyde S, Liu R, Ma X, Konig M, et al. Efficacy and safety of oral orforglipron in patients with type 2 diabetes: a multicentre, randomised, dose-response, phase 2 study. *Lancet* 2023; **402**(10400): 472-83.
33. Gao L, Lee BW, Chawla M, Kim J, Huo L, Du L, et al. Tirzepatide versus insulin glargine as second-line or third-line therapy in type 2 diabetes in the Asia-Pacific region: the SURPASS-AP-Combo trial. *Nat Med* 2023; **29**(6): 1500-10.
34. Garvey WT, Frias JP, Jastreboff AM, le Roux CW, Sattar N, Aizenberg D, et al. Tirzepatide once weekly for the treatment of obesity in people with type 2 diabetes (SURMOUNT-2): a double-blind, randomised, multicentre, placebo-controlled, phase 3 trial. *Lancet* 2023; **402**(10402): 613-26.
35. Jastreboff AM, Kaplan LM, Frias JP, Wu Q, Du Y, Gurbuz S, et al. Triple-Hormone-Receptor Agonist Retatrutide for Obesity - A Phase 2 Trial. *N Engl J Med* 2023; **389**(6): 514-26.
36. Ji L, Lu Y, Li Q, Fu L, Luo Y, Lei T, et al. Efficacy and safety of empagliflozin in combination with insulin in Chinese patients with type 2 diabetes and insufficient glycaemic control: A phase III, randomized, double-blind, placebo-controlled, parallel study. *Diabetes Obes Metab* 2023; **25**(7): 1839-48.
37. Loomba R, Abdelmalek MF, Armstrong MJ, Jara M, Kjaer MS, Krarup N, et al. Semaglutide 2.4 mg once weekly in patients with non-alcoholic steatohepatitis-related cirrhosis: a randomised, placebo-controlled phase 2 trial. *Lancet Gastroenterol Hepatol* 2023; **8**(6): 511-22.
38. Ramos EL, Dayan CM, Chatenoud L, Sumnik Z, Simmons KM, Szypowska A, et al. Teplizumab and beta-Cell Function in Newly Diagnosed Type 1 Diabetes. *N Engl J Med* 2023; **389**(23): 2151-61.
39. Rosenstock J, Frias JP, Rodbard HW, Tofe S, Sears E, Huh R, et al. Tirzepatide vs Insulin Lispro Added to Basal Insulin in Type 2 Diabetes: The SURPASS-6 Randomized Clinical Trial. *Jama* 2023; **330**(17): 1631-40.
40. Fox CK, Clark JM, Rudser KD, Ryder JR, Gross AC, Nathan BM, et al. Exenatide for weight-loss maintenance in adolescents with severe

obesity: A randomized, placebo-controlled trial. *Obesity (Silver Spring)* 2022; **30**(5): 1105-15.
41. Garvey WT, Batterham RL, Bhatta M, Buscemi S, Christensen LN, Frias JP, et al. Two-year effects of semaglutide in adults with overweight or obesity: the STEP 5 trial. *Nat Med* 2022; **28**(10): 2083-91.
42. Heise T, Mari A, DeVries JH, Urva S, Li J, Pratt EJ, et al. Effects of subcutaneous tirzepatide versus placebo or semaglutide on pancreatic islet function and insulin sensitivity in adults with type 2 diabetes: a multicentre, randomised, double-blind, parallel-arm, phase 1 clinical trial. *Lancet Diabetes Endocrinol* 2022; **10**(6): 418-29.
43. Kadowaki T, Isendahl J, Khalid U, Lee SY, Nishida T, Ogawa W, et al. Semaglutide once a week in adults with overweight or obesity, with or without type 2 diabetes in an east Asian population (STEP 6): a randomised, double-blind, double-dummy, placebo-controlled, phase 3a trial. *Lancet Diabetes Endocrinol* 2022; **10**(3): 193-206.
44. Kadowaki T, Chin R, Ozeki A, Imaoka T, Ogawa Y. Safety and efficacy of tirzepatide as an add-on to single oral antihyperglycaemic medication in patients with type 2 diabetes in Japan (SURPASS J-combo): a multicentre, randomised, open-label, parallel-group, phase 3 trial. *Lancet Diabetes Endocrinol* 2022; **10**(9): 634-44.
45. Nordisk N. Research Study Investigating How Well Semaglutide Works in People From Thailand and South Korea Living With Obesity. 2022. https://clinicaltrials.gov/study/NCT04998136?cond=NCT04998136&rank=1 (accessed 2026/01/31 2026).
46. Wada T, Mori-Anai K, Takahashi A, Matsui T, Inagaki M, Iida M, et al. Effect of canagliflozin on the decline of estimated glomerular filtration rate in chronic kidney disease patients with type 2 diabetes mellitus: A multicenter, randomized, double-blind, placebo-controlled, parallel-group, phase III study in Japan. *J Diabetes Investig* 2022; **13**(12): 1981-9.
47. Nassif ME, Spertus JA, Tang F, Windsor SL, Jones P, Thomas M, et al. Association Between Change in Ambulatory Hemodynamic Pressures and Symptoms of Heart Failure. *Circ Heart Fail* 2021; **14**(11): e008446.
48. Rodgers M, Migdal AL, Rodriguez TG, Chen ZZ, Nath AK, Gerszten RE, et al. Weight Loss Outcomes Among Early High Responders to Exenatide Treatment: A Randomized, Placebo Controlled Study in Overweight and Obese Women. *Front Endocrinol (Lausanne)* 2021; **12**: 742873.
49. Rosenstock J, Wysham C, Frias JP, Kaneko S, Lee CJ, Fernandez Lando L, et al. Efficacy and safety of a novel dual GIP and GLP-1 receptor

agonist tirzepatide in patients with type 2 diabetes (SURPASS-1): a double-blind, randomised, phase 3 trial. *Lancet* 2021; **398**(10295): 143-55.
50. Rubino D, Abrahamsson N, Davies M, Hesse D, Greenway FL, Jensen C, et al. Effect of Continued Weekly Subcutaneous Semaglutide vs Placebo on Weight Loss Maintenance in Adults With Overweight or Obesity: The STEP 4 Randomized Clinical Trial. *Jama* 2021; **325**(14): 1414-25.
51. Santos-Gallego CG, Vargas-Delgado AP, Requena-Ibanez JA, Garcia-Ropero A, Mancini D, Pinney S, et al. Randomized Trial of Empagliflozin in Nondiabetic Patients With Heart Failure and Reduced Ejection Fraction. *J Am Coll Cardiol* 2021; **77**(3): 243-55.
52. Stack AG, Han D, Goldwater R, Johansson S, Dronamraju N, Oscarsson J, et al. Dapagliflozin Added to Verinurad Plus Febuxostat Further Reduces Serum Uric Acid in Hyperuricemia: The QUARTZ Study. *J Clin Endocrinol Metab* 2021; **106**(5): e2347-e56.
53. Wadden TA, Bailey TS, Billings LK, Davies M, Frias JP, Koroleva A, et al. Effect of Subcutaneous Semaglutide vs Placebo as an Adjunct to Intensive Behavioral Therapy on Body Weight in Adults With Overweight or Obesity: The STEP 3 Randomized Clinical Trial. *Jama* 2021; **325**(14): 1403-13.
54. Wason S. Efficacy and Bone Safety of Sotagliflozin 400 and 200 mg Versus Placebo in Participants With Type 2 Diabetes Mellitus Who Have Inadequate Glycemic Control (SOTA-BONE). 2021. https://clinicaltrials.gov/study/NCT03386344?cond=NCT03386344&rank=1 (accessed 2024/10/28 2024).
55. Yang W, Xu X, Lei T, Ma J, Li L, Shen J, et al. Efficacy and safety of linagliptin as add-on therapy to insulin in Chinese patients with type 2 diabetes mellitus: A randomized, double-blind, placebo-controlled trial. *Diabetes Obes Metab* 2021; **23**(2): 642-7.
56. Capehorn MS, Catarig AM, Furberg JK, Janez A, Price HC, Tadayon S, et al. Efficacy and safety of once-weekly semaglutide 1.0mg vs once-daily liraglutide 1.2mg as add-on to 1-3 oral antidiabetic drugs in subjects with type 2 diabetes (SUSTAIN 10). *Diabetes Metab* 2020; **46**(2): 100-9.
57. Aroda VR, Rosenstock J, Terauchi Y, Altuntas Y, Lalic NM, Morales Villegas EC, et al. PIONEER 1: Randomized Clinical Trial of the Efficacy and Safety of Oral Semaglutide Monotherapy in Comparison With Placebo in Patients With Type 2 Diabetes. *Diabetes Care* 2019; **42**(9): 1724-32.
58. Herold KC, Bundy BN, Long SA, Bluestone JA, DiMeglio LA, Dufort MJ, et al. An Anti-CD3 Antibody, Teplizumab, in Relatives at Risk for Type 1 Diabetes. *N Engl J Med* 2019; **381**(7): 603-13.

59. Mosenzon O, Blicher TM, Rosenlund S, Eriksson JW, Heller S, Hels OH, et al. Efficacy and safety of oral semaglutide in patients with type 2 diabetes and moderate renal impairment (PIONEER 5): a placebo-controlled, randomised, phase 3a trial. *Lancet Diabetes Endocrinol* 2019; **7**(7): 515-27.
60. Mullins RJ, Mustapic M, Chia CW, Carlson O, Gulyani S, Tran J, et al. A Pilot Study of Exenatide Actions in Alzheimer's Disease. *Curr Alzheimer Res* 2019; **16**(8): 741-52.
61. Nassif ME, Windsor SL, Tang F, Khariton Y, Husain M, Inzucchi SE, et al. Dapagliflozin Effects on Biomarkers, Symptoms, and Functional Status in Patients With Heart Failure With Reduced Ejection Fraction: The DEFINE-HF Trial. *Circulation* 2019; **140**(18): 1463-76.
62. Pieber TR, Bode B, Mertens A, Cho YM, Christiansen E, Hertz CL, et al. Efficacy and safety of oral semaglutide with flexible dose adjustment versus sitagliptin in type 2 diabetes (PIONEER 7): a multicentre, open-label, randomised, phase 3a trial. *Lancet Diabetes Endocrinol* 2019; **7**(7): 528-39.
63. Pollock C, Stefansson B, Reyner D, Rossing P, Sjostrom CD, Wheeler DC, et al. Albuminuria-lowering effect of dapagliflozin alone and in combination with saxagliptin and effect of dapagliflozin and saxagliptin on glycaemic control in patients with type 2 diabetes and chronic kidney disease (DELIGHT): a randomised, double-blind, placebo-controlled trial. *Lancet Diabetes Endocrinol* 2019; **7**(6): 429-41.
64. Pratley R, Amod A, Hoff ST, Kadowaki T, Lingvay I, Nauck M, et al. Oral semaglutide versus subcutaneous liraglutide and placebo in type 2 diabetes (PIONEER 4): a randomised, double-blind, phase 3a trial. *Lancet* 2019; **394**(10192): 39-50.
65. Rodbard HW, Rosenstock J, Canani LH, Deerochanawong C, Gumprecht J, Lindberg SO, et al. Oral Semaglutide Versus Empagliflozin in Patients With Type 2 Diabetes Uncontrolled on Metformin: The PIONEER 2 Trial. *Diabetes Care* 2019; **42**(12): 2272-81.
66. Rosenstock J, Perl S, Johnsson E, Garcia-Sanchez R, Jacob S. Triple therapy with low-dose dapagliflozin plus saxagliptin versus dual therapy with each monocomponent, all added to metformin, in uncontrolled type 2 diabetes. *Diabetes Obes Metab* 2019; **21**(9): 2152-62.
67. Wang J, Li HQ, Xu XH, Kong XC, Sun R, Jing T, et al. The Effects of Once-Weekly Dulaglutide and Insulin Glargine on Glucose Fluctuation in Poorly Oral-Antidiabetic Controlled Patients with Type 2 Diabetes Mellitus. *Biomed Res Int* 2019; **2019**: 2682657.
68. Zinman B, Aroda VR, Buse JB, Cariou B, Harris SB, Hoff ST, et al. Efficacy, Safety, and Tolerability of Oral Semaglutide Versus Placebo Added to Insulin With or Without Metformin in Patients With Type 2 Diabetes: The PIONEER 8 Trial. *Diabetes Care* 2019; **42**(12): 2262-71.

69. Aronson R, Frias J, Goldman A, Darekar A, Lauring B, Terra SG. Long-term efficacy and safety of ertugliflozin monotherapy in patients with inadequately controlled T2DM despite diet and exercise: VERTIS MONO extension study. *Diabetes Obes Metab* 2018; **20**(6): 1453-60.
70. Buse JB, Garg SK, Rosenstock J, Bailey TS, Banks P, Bode BW, et al. Sotagliflozin in Combination With Optimized Insulin Therapy in Adults With Type 1 Diabetes: The North American inTandem1 Study. *Diabetes Care* 2018; **41**(9): 1970-80.
71. Chen Y, Liu X, Li Q, Ma J, Lv X, Guo L, et al. Saxagliptin add-on therapy in Chinese patients with type 2 diabetes inadequately controlled by insulin with or without metformin: Results from the SUPER study, a randomized, double-blind, placebo-controlled trial. *Diabetes Obes Metab* 2018; **20**(4): 1044-9.
72. Coskun T, Sloop KW, Loghin C, Alsina-Fernandez J, Urva S, Bokvist KB, et al. LY3298176, a novel dual GIP and GLP-1 receptor agonist for the treatment of type 2 diabetes mellitus: From discovery to clinical proof of concept. *Mol Metab* 2018; **18**: 3-14.
73. Danne T, Cariou B, Banks P, Brandle M, Brath H, Franek E, et al. HbA(1c) and Hypoglycemia Reductions at 24 and 52 Weeks With Sotagliflozin in Combination With Insulin in Adults With Type 1 Diabetes: The European inTandem2 Study. *Diabetes Care* 2018; **41**(9): 1981-90.
74. Dou J, Ma J, Liu J, Wang C, Johnsson E, Yao H, et al. Efficacy and safety of saxagliptin in combination with metformin as initial therapy in Chinese patients with type 2 diabetes: Results from the START study, a multicentre, randomized, double-blind, active-controlled, phase 3 trial. *Diabetes Obes Metab* 2018; **20**(3): 590-8.
75. Frias JP, Nauck MA, Van J, Kutner ME, Cui X, Benson C, et al. Efficacy and safety of LY3298176, a novel dual GIP and GLP-1 receptor agonist, in patients with type 2 diabetes: a randomised, placebo-controlled and active comparator-controlled phase 2 trial. *Lancet* 2018; **392**(10160): 2180-93.
76. Iwamoto N, Matsui A, Kazama H, Oura T. Subgroup Analysis Stratified by Baseline Pancreatic beta-cell Function in a Japanese Study of Dulaglutide in Patients with Type 2 Diabetes. *Diabetes Ther* 2018; **9**(1): 383-94.
77. Kaku K, Yamada Y, Watada H, Abiko A, Nishida T, Zacho J, et al. Safety and efficacy of once-weekly semaglutide vs additional oral antidiabetic drugs in Japanese people with inadequately controlled type 2 diabetes: A randomized trial. *Diabetes Obes Metab* 2018; **20**(5): 1202-12.
78. Ludvik B, Frias JP, Tinahones FJ, Wainstein J, Jiang H, Robertson KE, et al. Dulaglutide as add-on therapy to SGLT2 inhibitors in patients with

inadequately controlled type 2 diabetes (AWARD-10): a 24-week, randomised, double-blind, placebo-controlled trial. *Lancet Diabetes Endocrinol* 2018; **6**(5): 370-81.
79. Muller-Wieland D, Kellerer M, Cypryk K, Skripova D, Rohwedder K, Johnsson E, et al. Efficacy and safety of dapagliflozin or dapagliflozin plus saxagliptin versus glimepiride as add-on to metformin in patients with type 2 diabetes. *Diabetes Obes Metab* 2018; **20**(11): 2598-607.
80. Rodbard HW, Lingvay I, Reed J, de la Rosa R, Rose L, Sugimoto D, et al. Semaglutide Added to Basal Insulin in Type 2 Diabetes (SUSTAIN 5): A Randomized, Controlled Trial. *J Clin Endocrinol Metab* 2018; **103**(6): 2291-301.
81. Scott R, Morgan J, Zimmer Z, Lam RLH, O'Neill EA, Kaufman KD, et al. A randomized clinical trial of the efficacy and safety of sitagliptin compared with dapagliflozin in patients with type 2 diabetes mellitus and mild renal insufficiency: The CompoSIT-R study. *Diabetes Obes Metab* 2018; **20**(12): 2876-84.
82. Seino Y, Terauchi Y, Osonoi T, Yabe D, Abe N, Nishida T, et al. Safety and efficacy of semaglutide once weekly vs sitagliptin once daily, both as monotherapy in Japanese people with type 2 diabetes. *Diabetes Obes Metab* 2018; **20**(2): 378-88.
83. Tuttle KR, Lakshmanan MC, Rayner B, Busch RS, Zimmermann AG, Woodward DB, et al. Dulaglutide versus insulin glargine in patients with type 2 diabetes and moderate-to-severe chronic kidney disease (AWARD-7): a multicentre, open-label, randomised trial. *Lancet Diabetes Endocrinol* 2018; **6**(8): 605-17.
84. Zhu D, Gan S, Liu Y, Ma J, Dong X, Song W, et al. Dorzagliatin monotherapy in Chinese patients with type 2 diabetes: a dose-ranging, randomised, double-blind, placebo-controlled, phase 2 study. *Lancet Diabetes Endocrinol* 2018; **6**(8): 627-36.
85. Davies M, Pieber TR, Hartoft-Nielsen ML, Hansen OKH, Jabbour S, Rosenstock J. Effect of Oral Semaglutide Compared With Placebo and Subcutaneous Semaglutide on Glycemic Control in Patients With Type 2 Diabetes: A Randomized Clinical Trial. *Jama* 2017; **318**(15): 1460-70.
86. Du J, Liang L, Fang H, Xu F, Li W, Shen L, et al. Efficacy and safety of saxagliptin compared with acarbose in Chinese patients with type 2 diabetes mellitus uncontrolled on metformin monotherapy: Results of a Phase IV open-label randomized controlled study (the SMART study). *Diabetes Obes Metab* 2017; **19**(11): 1513-20.
87. Gadde KM, Vetter ML, Iqbal N, Hardy E, Ohman P, investigators D-N-s. Efficacy and safety of autoinjected exenatide once-weekly suspension versus sitagliptin or placebo with metformin in patients with type 2 diabetes: The DURATION-NEO-2 randomized clinical study.

Diabetes Obes Metab 2017; **19**(7): 979-88.
88. Gantz I, Okamoto T, Ito Y, Okuyama K, O'Neill EA, Kaufman KD, et al. A randomized, placebo- and sitagliptin-controlled trial of the safety and efficacy of omarigliptin, a once-weekly dipeptidyl peptidase-4 inhibitor, in Japanese patients with type 2 diabetes. *Diabetes Obes Metab* 2017; **19**(11): 1602-9.
89. Handelsman Y, Lauring B, Gantz I, Iredale C, O'Neill EA, Wei Z, et al. A randomized, double-blind, non-inferiority trial evaluating the efficacy and safety of omarigliptin, a once-weekly DPP-4 inhibitor, or glimepiride in patients with type 2 diabetes inadequately controlled on metformin monotherapy. *Curr Med Res Opin* 2017; **33**(10): 1861-8.
90. Home PD, Ahren B, Reusch JEB, Rendell M, Weissman PN, Cirkel DT, et al. Three-year data from 5 HARMONY phase 3 clinical trials of albiglutide in type 2 diabetes mellitus: Long-term efficacy with or without rescue therapy. *Diabetes Res Clin Pract* 2017; **131**: 49-60.
91. Lee SH, Gantz I, Round E, Latham M, O'Neill EA, Ceesay P, et al. A randomized, placebo-controlled clinical trial evaluating the safety and efficacy of the once-weekly DPP-4 inhibitor omarigliptin in patients with type 2 diabetes mellitus inadequately controlled by glimepiride and metformin. *BMC Endocr Disord* 2017; **17**(1): 70.
92. Meneilly GS, Roy-Duval C, Alawi H, Dailey G, Bellido D, Trescoli C, et al. Lixisenatide Therapy in Older Patients With Type 2 Diabetes Inadequately Controlled on Their Current Antidiabetic Treatment: The GetGoal-O Randomized Trial. *Diabetes Care* 2017; **40**(4): 485-93.
93. Mu Y, Pan C, Fan B, Hehnke U, Zhang X, Zhang X, et al. Efficacy and safety of linagliptin/metformin single-pill combination as initial therapy in drug-naive Asian patients with type 2 diabetes. *Diabetes Res Clin Pract* 2017; **124**: 48-56.
94. Pan C, Han P, Ji Q, Li C, Lu J, Yang J, et al. Efficacy and safety of alogliptin in patients with type 2 diabetes mellitus: A multicentre randomized double-blind placebo-controlled Phase 3 study in mainland China, Taiwan, and Hong Kong. *J Diabetes* 2017; **9**(4): 386-95.
95. Shankar RR, Inzucchi SE, Scarabello V, Gantz I, Kaufman KD, Lai E, et al. A randomized clinical trial evaluating the efficacy and safety of the once-weekly dipeptidyl peptidase-4 inhibitor omarigliptin in patients with type 2 diabetes inadequately controlled on metformin monotherapy. *Curr Med Res Opin* 2017; **33**(10): 1853-60.
96. Sorli C, Harashima SI, Tsoukas GM, Unger J, Karsbol JD, Hansen T, et al. Efficacy and safety of once-weekly semaglutide monotherapy versus placebo in patients with type 2 diabetes (SUSTAIN 1): a double-blind, randomised, placebo-controlled, parallel-group, multinational,

multicentre phase 3a trial. *Lancet Diabetes Endocrinol* 2017; **5**(4): 251-60.
97. Terauchi Y, Yamada Y, Ishida H, Ohsugi M, Kitaoka M, Satoh J, et al. Efficacy and safety of sitagliptin as compared with glimepiride in Japanese patients with type 2 diabetes mellitus aged >/= 60 years (START-J trial). *Diabetes Obes Metab* 2017; **19**(8): 1188-92.
98. Tinahones FJ, Gallwitz B, Nordaby M, Gotz S, Maldonado-Lutomirsky M, Woerle HJ, et al. Linagliptin as add-on to empagliflozin and metformin in patients with type 2 diabetes: Two 24-week randomized, double-blind, double-dummy, parallel-group trials. *Diabetes Obes Metab* 2017; **19**(2): 266-74.
99. Wang W, Ning G, Ma J, Liu X, Zheng S, Wu F, et al. A randomized clinical trial of the safety and efficacy of sitagliptin in patients with type 2 diabetes mellitus inadequately controlled by acarbose alone. *Curr Med Res Opin* 2017; **33**(4): 693-9.
100. Yu M, Brunt KV, Milicevic Z, Varnado O, Boye KS. Patient-reported Outcomes in Patients with Type 2 Diabetes Treated with Dulaglutide Added to Titrated Insulin Glargine (AWARD-9). *Clin Ther* 2017; **39**(11): 2284-95.
101. Dungan KM, Weitgasser R, Perez Manghi F, Pintilei E, Fahrbach JL, Jiang HH, et al. A 24-week study to evaluate the efficacy and safety of once-weekly dulaglutide added on to glimepiride in type 2 diabetes (AWARD-8). *Diabetes Obes Metab* 2016; **18**(5): 475-82.
102. Investigators F-ST. Glucose Variability in a 26-Week Randomized Comparison of Mealtime Treatment With Rapid-Acting Insulin Versus GLP-1 Agonist in Participants With Type 2 Diabetes at High Cardiovascular Risk. *Diabetes Care* 2016; **39**(6): 973-81.
103. Leiter LA, Cefalu WT, de Bruin TW, Xu J, Parikh S, Johnsson E, et al. Long-term maintenance of efficacy of dapagliflozin in patients with type 2 diabetes mellitus and cardiovascular disease. *Diabetes Obes Metab* 2016; **18**(8): 766-74.
104. Lepore JJ, Olson E, Demopoulos L, Haws T, Fang Z, Barbour AM, et al. Effects of the Novel Long-Acting GLP-1 Agonist, Albiglutide, on Cardiac Function, Cardiac Metabolism, and Exercise Capacity in Patients With Chronic Heart Failure and Reduced Ejection Fraction. *JACC Heart Fail* 2016; **4**(7): 559-66.
105. Mellander A, Billger M, Johnsson E, Traff AK, Yoshida S, Johnsson K. Hypersensitivity Events, Including Potentially Hypersensitivity-Related Skin Events, with Dapagliflozin in Patients with Type 2 Diabetes Mellitus: A Pooled Analysis. *Clinical drug investigation* 2016; **36**(11): 925-33.
106. Moses RG, Round E, Shentu Y, Golm GT, O'Neill E A, Gantz I, et al. A randomized clinical trial evaluating the safety and efficacy of sitagliptin added to the combination of sulfonylurea and metformin in patients with type 2 diabetes mellitus and inadequate glycemic control. *J Diabetes*

2016; **8**(5): 701-11.
107. Nauck M, Rizzo M, Johnson A, Bosch-Traberg H, Madsen J, Cariou B. Once-Daily Liraglutide Versus Lixisenatide as Add-on to Metformin in Type 2 Diabetes: A 26-Week Randomized Controlled Clinical Trial. *Diabetes Care* 2016; **39**(9): 1501-9.
108. Wang W, Yang J, Yang G, Gong Y, Patel S, Zhang C, et al. Efficacy and safety of linagliptin in Asian patients with type 2 diabetes mellitus inadequately controlled by metformin: A multinational 24-week, randomized clinical trial. *J Diabetes* 2016; **8**(2): 229-37.
109. Zang L, Liu Y, Geng J, Luo Y, Bian F, Lv X, et al. Efficacy and safety of liraglutide versus sitagliptin, both in combination with metformin, in Chinese patients with type 2 diabetes: a 26-week, open-label, randomized, active comparator clinical trial. *Diabetes Obes Metab* 2016; **18**(8): 803-11.
110. Blonde L, Jendle J, Gross J, Woo V, Jiang H, Fahrbach JL, et al. Once-weekly dulaglutide versus bedtime insulin glargine, both in combination with prandial insulin lispro, in patients with type 2 diabetes (AWARD-4): a randomised, open-label, phase 3, non-inferiority study. *Lancet* 2015; **385**(9982): 2057-66.
111. Cefalu WT, Leiter LA, de Bruin TW, Gause-Nilsson I, Sugg J, Parikh SJ. Dapagliflozin's Effects on Glycemia and Cardiovascular Risk Factors in High-Risk Patients With Type 2 Diabetes: A 24-Week, Multicenter, Randomized, Double-Blind, Placebo-Controlled Study With a 28-Week Extension. *Diabetes Care* 2015; **38**(7): 1218-27.
112. DeFronzo RA, Lewin A, Patel S, Liu D, Kaste R, Woerle HJ, et al. Combination of empagliflozin and linagliptin as second-line therapy in subjects with type 2 diabetes inadequately controlled on metformin. *Diabetes Care* 2015; **38**(3): 384-93.
113. Hartley P, Shentu Y, Betz-Schiff P, Golm GT, Sisk CM, Engel SS, et al. Efficacy and Tolerability of Sitagliptin Compared with Glimepiride in Elderly Patients with Type 2 Diabetes Mellitus and Inadequate Glycemic Control: A Randomized, Double-Blind, Non-Inferiority Trial. *Drugs Aging* 2015; **32**(6): 469-76.
114. Hirose T, Suzuki M, Tsumiyama I. Efficacy and Safety of Vildagliptin as an Add-on to Insulin with or without Metformin in Japanese Patients with Type 2 Diabetes Mellitus: A 12-week, Double-Blind, Randomized Study. *Diabetes Ther* 2015; **6**(4): 559-71.
115. Ji L, Zinman B, Patel S, Ji J, Bailes Z, Thiemann S, et al. Efficacy and safety of linagliptin co-administered with low-dose metformin once daily versus high-dose metformin twice daily in treatment-naive patients with type 2 diabetes: a double-blind randomized trial. *Adv Ther* 2015;

32(3): 201-15.
116. Kovacs CS, Seshiah V, Merker L, Christiansen AV, Roux F, Salsali A, et al. Empagliflozin as Add-on Therapy to Pioglitazone With or Without Metformin in Patients With Type 2 Diabetes Mellitus. *Clin Ther* 2015; **37**(8): 1773-88 e1.
117. Rosenstock J, Hansen L, Zee P, Li Y, Cook W, Hirshberg B, et al. Dual add-on therapy in type 2 diabetes poorly controlled with metformin monotherapy: a randomized double-blind trial of saxagliptin plus dapagliflozin addition versus single addition of saxagliptin or dapagliflozin to metformin. *Diabetes Care* 2015; **38**(3): 376-83.
118. Bajaj M, Gilman R, Patel S, Kempthorne-Rawson J, Lewis-D'Agostino D, Woerle HJ. Linagliptin improved glycaemic control without weight gain or hypoglycaemia in patients with type 2 diabetes inadequately controlled by a combination of metformin and pioglitazone: a 24-week randomized, double-blind study. *Diabet Med* 2014; **31**(12): 1505-14.
119. Barnett AH, Mithal A, Manassie J, Jones R, Rattunde H, Woerle HJ, et al. Efficacy and safety of empagliflozin added to existing antidiabetes treatment in patients with type 2 diabetes and chronic kidney disease: a randomised, double-blind, placebo-controlled trial. *Lancet Diabetes Endocrinol* 2014; **2**(5): 369-84.
120. Dungan KM, Povedano ST, Forst T, Gonzalez JG, Atisso C, Sealls W, et al. Once-weekly dulaglutide versus once-daily liraglutide in metformin-treated patients with type 2 diabetes (AWARD-6): a randomised, open-label, phase 3, non-inferiority trial. *Lancet* 2014; **384**(9951): 1349-57.
121. Henry RR, Staels B, Fonseca VA, Chou MZ, Teng R, Golm GT, et al. Efficacy and safety of initial combination treatment with sitagliptin and pioglitazone--a factorial study. *Diabetes Obes Metab* 2014; **16**(3): 223-30.
122. McGill JB, Barnett AH, Lewin AJ, Patel S, Neubacher D, von Eynatten M, et al. Linagliptin added to sulphonylurea in uncontrolled type 2 diabetes patients with moderate-to-severe renal impairment. *Diab Vasc Dis Res* 2014; **11**(1): 34-40.
123. Polidori D, Mari A, Ferrannini E. Canagliflozin, a sodium glucose co-transporter 2 inhibitor, improves model-based indices of beta cell function in patients with type 2 diabetes. *Diabetologia* 2014; **57**(5): 891-901.
124. Pratley RE, Fleck P, Wilson C. Efficacy and safety of initial combination therapy with alogliptin plus metformin versus either as monotherapy in drug-naive patients with type 2 diabetes: a randomized, double-blind, 6-month study. *Diabetes Obes Metab* 2014; **16**(7): 613-

21.
125. Umpierrez G, Tofe Povedano S, Perez Manghi F, Shurzinske L, Pechtner V. Efficacy and safety of dulaglutide monotherapy versus metformin in type 2 diabetes in a randomized controlled trial (AWARD-3). *Diabetes Care* 2014; **37**(8): 2168-76.
126. Van Gaal L, Souhami E, Zhou T, Aronson R. Efficacy and safety of the glucagon-like peptide-1 receptor agonist lixisenatide versus the dipeptidyl peptidase-4 inhibitor sitagliptin in young (<50 years) obese patients with type 2 diabetes mellitus. *J Clin Transl Endocrinol* 2014; **1**(2): 31-7.
127. White JL, Buchanan P, Li J, Frederich R. A randomized controlled trial of the efficacy and safety of twice-daily saxagliptin plus metformin combination therapy in patients with type 2 diabetes and inadequate glycemic control on metformin monotherapy. *BMC Endocr Disord* 2014; **14**: 17.
128. Alba M, Ahren B, Inzucchi SE, Guan Y, Mallick M, Xu L, et al. Sitagliptin and pioglitazone provide complementary effects on postprandial glucose and pancreatic islet cell function. *Diabetes Obes Metab* 2013; **15**(12): 1101-10.
129. Arjona Ferreira JC, Corry D, Mogensen CE, Sloan L, Xu L, Golm GT, et al. Efficacy and safety of sitagliptin in patients with type 2 diabetes and ESRD receiving dialysis: a 54-week randomized trial. *Am J Kidney Dis* 2013; **61**(4): 579-87.
130. Barnett AH, Huisman H, Jones R, von Eynatten M, Patel S, Woerle HJ. Linagliptin for patients aged 70 years or older with type 2 diabetes inadequately controlled with common antidiabetes treatments: a randomised, double-blind, placebo-controlled trial. *Lancet* 2013; **382**(9902): 1413-23.
131. Barnett AH, Charbonnel B, Li J, Donovan M, Fleming D, Iqbal N. Saxagliptin add-on therapy to insulin with or without metformin for type 2 diabetes mellitus: 52-week safety and efficacy. *Clinical drug investigation* 2013; **33**(10): 707-17.
132. Dobs AS, Goldstein BJ, Aschner P, Horton ES, Umpierrez GE, Duran L, et al. Efficacy and safety of sitagliptin added to ongoing metformin and rosiglitazone combination therapy in a randomized placebo-controlled 54-week trial in patients with type 2 diabetes. *J Diabetes* 2013; **5**(1): 68-79.
133. Ferrannini E, Berk A, Hantel S, Pinnetti S, Hach T, Woerle HJ, et al. Long-term safety and efficacy of empagliflozin, sitagliptin, and metformin: an active-controlled, parallel-group, randomized, 78-week open-label extension study in patients with type 2 diabetes. *Diabetes*

Care 2013; **36**(12): 4015-21.
134. Haak T, Meinicke T, Jones R, Weber S, von Eynatten M, Woerle HJ. Initial combination of linagliptin and metformin in patients with type 2 diabetes: efficacy and safety in a randomised, double-blind 1-year extension study. *International journal of clinical practice* 2013; **67**(12): 1283-93.
135. Herold KC, Gitelman SE, Ehlers MR, Gottlieb PA, Greenbaum CJ, Hagopian W, et al. Teplizumab (anti-CD3 mAb) treatment preserves C-peptide responses in patients with new-onset type 1 diabetes in a randomized controlled trial: metabolic and immunologic features at baseline identify a subgroup of responders. *Diabetes* 2013; **62**(11): 3766-74.
136. Herold KC, Gitelman SE, Willi SM, Gottlieb PA, Waldron-Lynch F, Devine L, et al. Teplizumab treatment may improve C-peptide responses in participants with type 1 diabetes after the new-onset period: a randomised controlled trial. *Diabetologia* 2013; **56**(2): 391-400.
137. Kadowaki T, Kondo K. Efficacy, safety and dose-response relationship of teneligliptin, a dipeptidyl peptidase-4 inhibitor, in Japanese patients with type 2 diabetes mellitus. *Diabetes Obes Metab* 2013; **15**(9): 810-8.
138. Philis-Tsimikas A, Del Prato S, Satman I, Bhargava A, Dharmalingam M, Skjoth TV, et al. Effect of insulin degludec versus sitagliptin in patients with type 2 diabetes uncontrolled on oral antidiabetic agents. *Diabetes Obes Metab* 2013; **15**(8): 760-6.
139. Rosenstock J, Seman LJ, Jelaska A, Hantel S, Pinnetti S, Hach T, et al. Efficacy and safety of empagliflozin, a sodium glucose cotransporter 2 (SGLT2) inhibitor, as add-on to metformin in type 2 diabetes with mild hyperglycaemia. *Diabetes Obes Metab* 2013; **15**(12): 1154-60.
140. Rosenstock J, Raccah D, Koranyi L, Maffei L, Boka G, Miossec P, et al. Efficacy and safety of lixisenatide once daily versus exenatide twice daily in type 2 diabetes inadequately controlled on metformin: a 24-week, randomized, open-label, active-controlled study (GetGoal-X). *Diabetes Care* 2013; **36**(10): 2945-51.
141. Rosenstock J, Wilson C, Fleck P. Alogliptin versus glipizide monotherapy in elderly type 2 diabetes mellitus patients with mild hyperglycaemia: a prospective, double-blind, randomized, 1-year study. *Diabetes Obes Metab* 2013; **15**(10): 906-14.
142. Schernthaner G, Gross JL, Rosenstock J, Guarisco M, Fu M, Yee J, et al. Canagliflozin compared with sitagliptin for patients with type 2 diabetes who do not have adequate glycemic control with metformin plus sulfonylurea: a 52-week randomized trial. *Diabetes Care* 2013; **36**(9): 2508-15.

143. Barnett AH, Patel S, Harper R, Toorawa R, Thiemann S, von Eynatten M, et al. Linagliptin monotherapy in type 2 diabetes patients for whom metformin is inappropriate: an 18-week randomized, double-blind, placebo-controlled phase III trial with a 34-week active-controlled extension. *Diabetes Obes Metab* 2012; **14**(12): 1145-54.
144. DeFronzo RA, Burant CF, Fleck P, Wilson C, Mekki Q, Pratley RE. Efficacy and tolerability of the DPP-4 inhibitor alogliptin combined with pioglitazone, in metformin-treated patients with type 2 diabetes. *J Clin Endocrinol Metab* 2012; **97**(5): 1615-22.
145. Frederich R, McNeill R, Berglind N, Fleming D, Chen R. The efficacy and safety of the dipeptidyl peptidase-4 inhibitor saxagliptin in treatment-naive patients with type 2 diabetes mellitus: a randomized controlled trial. *Diabetol Metab Syndr* 2012; **4**(1): 36.
146. Haak T, Meinicke T, Jones R, Weber S, von Eynatten M, Woerle HJ. Initial combination of linagliptin and metformin improves glycaemic control in type 2 diabetes: a randomized, double-blind, placebo-controlled study. *Diabetes Obes Metab* 2012; **14**(6): 565-74.
147. Hermans MP, Delibasi T, Farmer I, Lohm L, Maheux P, Piatti P, et al. Effects of saxagliptin added to sub-maximal doses of metformin compared with uptitration of metformin in type 2 diabetes: the PROMPT study. *Curr Med Res Opin* 2012; **28**(10): 1635-45.
148. Kawamori R, Inagaki N, Araki E, Watada H, Hayashi N, Horie Y, et al. Linagliptin monotherapy provides superior glycaemic control versus placebo or voglibose with comparable safety in Japanese patients with type 2 diabetes: a randomized, placebo and active comparator-controlled, double-blind study. *Diabetes Obes Metab* 2012; **14**(4): 348-57.
149. Lewin AJ, Arvay L, Liu D, Patel S, von Eynatten M, Woerle HJ. Efficacy and tolerability of linagliptin added to a sulfonylurea regimen in patients with inadequately controlled type 2 diabetes mellitus: an 18-week, multicenter, randomized, double-blind, placebo-controlled trial. *Clin Ther* 2012; **34**(9): 1909-19 e15.
150. Rosenstock J, Aggarwal N, Polidori D, Zhao Y, Arbit D, Usiskin K, et al. Dose-ranging effects of canagliflozin, a sodium-glucose cotransporter 2 inhibitor, as add-on to metformin in subjects with type 2 diabetes. *Diabetes Care* 2012; **35**(6): 1232-8.
151. Ross SA, Rafeiro E, Meinicke T, Toorawa R, Weber-Born S, Woerle HJ. Efficacy and safety of linagliptin 2.5 mg twice daily versus 5 mg once daily in patients with type 2 diabetes inadequately controlled on metformin: a randomised, double-blind, placebo-controlled trial. *Curr Med Res Opin* 2012; **28**(9): 1465-74.
152. Russell-Jones D, Cuddihy RM, Hanefeld M, Kumar A, Gonzalez JG, Chan M, et al. Efficacy and safety of exenatide once weekly versus

metformin, pioglitazone, and sitagliptin used as monotherapy in drug-naive patients with type 2 diabetes (DURATION-4): a 26-week double-blind study. *Diabetes Care* 2012; **35**(2): 252-8.
153. Seino Y, Hiroi S, Hirayama M, Kaku K. Efficacy and safety of alogliptin added to sulfonylurea in Japanese patients with type 2 diabetes: A randomized, double-blind, placebo-controlled trial with an open-label, long-term extension study. *J Diabetes Investig* 2012; **3**(6): 517-25.
154. Seino Y, Miyata Y, Hiroi S, Hirayama M, Kaku K. Efficacy and safety of alogliptin added to metformin in Japanese patients with type 2 diabetes: a randomized, double-blind, placebo-controlled trial with an open-label, long-term extension study. *Diabetes Obes Metab* 2012; **14**(10): 927-36.
155. Yang W, Guan Y, Shentu Y, Li Z, Johnson-Levonas AO, Engel SS, et al. The addition of sitagliptin to ongoing metformin therapy significantly improves glycemic control in Chinese patients with type 2 diabetes. *J Diabetes* 2012; **4**(3): 227-37.
156. Gomis R, Espadero RM, Jones R, Woerle HJ, Dugi KA. Efficacy and safety of initial combination therapy with linagliptin and pioglitazone in patients with inadequately controlled type 2 diabetes: a randomized, double-blind, placebo-controlled study. *Diabetes Obes Metab* 2011; **13**(7): 653-61.
157. Nowicki M, Rychlik I, Haller H, Warren ML, Suchower L, Gause-Nilsson I, et al. Saxagliptin improves glycaemic control and is well tolerated in patients with type 2 diabetes mellitus and renal impairment. *Diabetes Obes Metab* 2011; **13**(6): 523-32.
158. Owens DR, Swallow R, Dugi KA, Woerle HJ. Efficacy and safety of linagliptin in persons with type 2 diabetes inadequately controlled by a combination of metformin and sulphonylurea: a 24-week randomized study. *Diabet Med* 2011; **28**(11): 1352-61.
159. Seino Y, Fujita T, Hiroi S, Hirayama M, Kaku K. Alogliptin plus voglibose in Japanese patients with type 2 diabetes: a randomized, double-blind, placebo-controlled trial with an open-label, long-term extension. *Curr Med Res Opin* 2011; **27 Suppl 3**: 21-9.
160. Yang W, Pan CY, Tou C, Zhao J, Gause-Nilsson I. Efficacy and safety of saxagliptin added to metformin in Asian people with type 2 diabetes mellitus: a randomized controlled trial. *Diabetes Res Clin Pract* 2011; **94**(2): 217-24.
161. Bailey CJ, Gross JL, Pieters A, Bastien A, List JF. Effect of dapagliflozin in patients with type 2 diabetes who have inadequate glycaemic control with metformin: a randomised, double-blind, placebo-controlled trial. *Lancet* 2010; **375**(9733): 2223-33.
162. Bergenstal RM, Wysham C, Macconell L, Malloy J, Walsh B, Yan P, et al. Efficacy and safety of exenatide once weekly versus sitagliptin or

pioglitazone as an adjunct to metformin for treatment of type 2 diabetes (DURATION-2): a randomised trial. *Lancet* 2010; **376**(9739): 431-9.
163. Iwamoto Y, Taniguchi T, Nonaka K, Okamoto T, Okuyama K, Arjona Ferreira JC, et al. Dose-ranging efficacy of sitagliptin, a dipeptidyl peptidase-4 inhibitor, in Japanese patients with type 2 diabetes mellitus. *Endocr J* 2010; **57**(5): 383-94.
164. Pratley RE, Nauck M, Bailey T, Montanya E, Cuddihy R, Filetti S, et al. Liraglutide versus sitagliptin for patients with type 2 diabetes who did not have adequate glycaemic control with metformin: a 26-week, randomised, parallel-group, open-label trial. *Lancet* 2010; **375**(9724): 1447-56.
165. Rosenstock J, Inzucchi SE, Seufert J, Fleck PR, Wilson CA, Mekki Q. Initial combination therapy with alogliptin and pioglitazone in drug-naive patients with type 2 diabetes. *Diabetes Care* 2010; **33**(11): 2406-8.
166. Chacra AR, Tan GH, Apanovitch A, Ravichandran S, List J, Chen R, et al. Saxagliptin added to a submaximal dose of sulphonylurea improves glycaemic control compared with uptitration of sulphonylurea in patients with type 2 diabetes: a randomised controlled trial. *International journal of clinical practice* 2009; **63**(9): 1395-406.
167. Lilly E. Protege Encore Study- Clinical Trial of Teplizumab (MGA031) in Children and Adults With Recent-Onset Type 1 Diabetes Mellitus (Protege Encore Study). 2009. https://clinicaltrials.gov/study/NCT00920582?cond=NCT00920582&rank=1 (accessed 2025/05/15 2025).
168. Garber A, Henry R, Ratner R, Garcia-Hernandez PA, Rodriguez-Pattzi H, Olvera-Alvarez I, et al. Liraglutide versus glimepiride monotherapy for type 2 diabetes (LEAD-3 Mono): a randomised, 52-week, phase III, double-blind, parallel-treatment trial. *Lancet* 2009; **373**(9662): 473-81.
169. Jadzinsky M, Pfutzner A, Paz-Pacheco E, Xu Z, Allen E, Chen R, et al. Saxagliptin given in combination with metformin as initial therapy improves glycaemic control in patients with type 2 diabetes compared with either monotherapy: a randomized controlled trial. *Diabetes Obes Metab* 2009; **11**(6): 611-22.
170. Mohan V, Yang W, Son HY, Xu L, Noble L, Langdon RB, et al. Efficacy and safety of sitagliptin in the treatment of patients with type 2 diabetes in China, India, and Korea. *Diabetes Res Clin Pract* 2009; **83**(1): 106-16.
171. Nauck M, Frid A, Hermansen K, Shah NS, Tankova T, Mitha IH, et al. Efficacy and safety comparison of liraglutide, glimepiride, and placebo, all in combination with metformin, in type 2 diabetes: the LEAD (liraglutide effect and action in diabetes)-2 study. *Diabetes Care* 2009; **32**(1): 84-90.

172. Nauck MA, Ellis GC, Fleck PR, Wilson CA, Mekki Q, Alogliptin Study G. Efficacy and safety of adding the dipeptidyl peptidase-4 inhibitor alogliptin to metformin therapy in patients with type 2 diabetes inadequately controlled with metformin monotherapy: a multicentre, randomised, double-blind, placebo-controlled study. *International journal of clinical practice* 2009; **63**(1): 46-55.
173. Pratley RE, Reusch JE, Fleck PR, Wilson CA, Mekki Q, Alogliptin Study G. Efficacy and safety of the dipeptidyl peptidase-4 inhibitor alogliptin added to pioglitazone in patients with type 2 diabetes: a randomized, double-blind, placebo-controlled study. *Curr Med Res Opin* 2009; **25**(10): 2361-71.
174. Rosenstock J, Rendell MS, Gross JL, Fleck PR, Wilson CA, Mekki Q. Alogliptin added to insulin therapy in patients with type 2 diabetes reduces HbA(1C) without causing weight gain or increased hypoglycaemia. *Diabetes Obes Metab* 2009; **11**(12): 1145-52.
175. DeFronzo RA, Fleck PR, Wilson CA, Mekki Q, Alogliptin Study G. Efficacy and safety of the dipeptidyl peptidase-4 inhibitor alogliptin in patients with type 2 diabetes and inadequate glycemic control: a randomized, double-blind, placebo-controlled study. *Diabetes Care* 2008; **31**(12): 2315-7.
176. Raz I, Chen Y, Wu M, Hussain S, Kaufman KD, Amatruda JM, et al. Efficacy and safety of sitagliptin added to ongoing metformin therapy in patients with type 2 diabetes. *Curr Med Res Opin* 2008; **24**(2): 537-50.
177. Goldstein BJ, Feinglos MN, Lunceford JK, Johnson J, Williams-Herman DE, Sitagliptin 036 Study G. Effect of initial combination therapy with sitagliptin, a dipeptidyl peptidase-4 inhibitor, and metformin on glycemic control in patients with type 2 diabetes. *Diabetes Care* 2007; **30**(8): 1979-87.
178. Charbonnel B, Karasik A, Liu J, Wu M, Meininger G, Sitagliptin Study G. Efficacy and safety of the dipeptidyl peptidase-4 inhibitor sitagliptin added to ongoing metformin therapy in patients with type 2 diabetes inadequately controlled with metformin alone. *Diabetes Care* 2006; **29**(12): 2638-43.
179. Rosenstock J, Brazg R, Andryuk PJ, Lu K, Stein P, Sitagliptin Study G. Efficacy and safety of the dipeptidyl peptidase-4 inhibitor sitagliptin added to ongoing pioglitazone therapy in patients with type 2 diabetes: a 24-week, multicenter, randomized, double-blind, placebo-controlled, parallel-group study. *Clin Ther* 2006; **28**(10): 1556-68.
180. Akasaka H, Sugimoto K, Shintani A, Taniuchi S, Yamamoto K, Iwakura K, et al. Effects of ipragliflozin on left ventricular diastolic function in

patients with type 2 diabetes and heart failure with preserved ejection fraction: The EXCEED randomized controlled multicenter study. *Geriatr Gerontol Int* 2022; **22**(4): 298-304.
181. Arturi F, Succurro E, Miceli S, Cloro C, Ruffo M, Maio R, et al. Liraglutide improves cardiac function in patients with type 2 diabetes and chronic heart failure. *Endocrine* 2017; **57**(3): 464-73.
182. Bailey TS, Takacs R, Tinahones FJ, Rao PV, Tsoukas GM, Thomsen AB, et al. Efficacy and safety of switching from sitagliptin to liraglutide in subjects with type 2 diabetes (LIRA-SWITCH): a randomized, double-blind, double-dummy, active-controlled 26-week trial. *Diabetes Obes Metab* 2016; **18**(12): 1191-8.
183. Bergenstal RM, Forti A, Chiasson JL, Woloschak M, Boldrin M, Balena R. Efficacy and safety of taspoglutide versus sitagliptin for type 2 diabetes mellitus (T-emerge 4 trial). *Diabetes Ther* 2012; **3**(1): 13.
184. Blonde L, Dagogo-Jack S, Banerji MA, Pratley RE, Marcellari A, Braceras R, et al. Comparison of vildagliptin and thiazolidinedione as add-on therapy in patients inadequately controlled with metformin: results of the GALIANT trial--a primary care, type 2 diabetes study. *Diabetes Obes Metab* 2009; **11**(10): 978-86.
185. Bosi E, Camisasca RP, Collober C, Rochotte E, Garber AJ. Effects of vildagliptin on glucose control over 24 weeks in patients with type 2 diabetes inadequately controlled with metformin. *Diabetes Care* 2007; **30**(4): 890-5.
186. Bosi E, Dotta F, Jia Y, Goodman M. Vildagliptin plus metformin combination therapy provides superior glycaemic control to individual monotherapy in treatment-naive patients with type 2 diabetes mellitus. *Diabetes Obes Metab* 2009; **11**(5): 506-15.
187. Carbone S, Billingsley HE, Canada JM, Bressi E, Rotelli B, Kadariya D, et al. The effects of canagliflozin compared to sitagliptin on cardiorespiratory fitness in type 2 diabetes mellitus and heart failure with reduced ejection fraction: The CANA-HF study. *Diabetes/metabolism research and reviews* 2020; **36**(8): e3335.
188. Chan JC, Scott R, Arjona Ferreira JC, Sheng D, Gonzalez E, Davies MJ, et al. Safety and efficacy of sitagliptin in patients with type 2 diabetes and chronic renal insufficiency. *Diabetes Obes Metab* 2008; **10**(7): 545-55.
189. Chow E, Wang K, Lim CKP, Tsoi STF, Fan B, Poon E, et al. Dorzagliatin, a Dual-Acting Glucokinase Activator, Increases Insulin Secretion and Glucose Sensitivity in Glucokinase Maturity-Onset Diabetes of the Young and Recent-Onset Type 2 Diabetes. *Diabetes* 2023; **72**(2): 299-308.

190. Dayem KA, Younis O, Zarif B, Attia S, AbdelSalam A. Impact of dapagliflozin on cardiac function following anterior myocardial infarction in non-diabetic patients - DACAMI (a randomized controlled clinical trial). *Int J Cardiol* 2023; **379**: 9-14.
191. de Boer SA, Heerspink HJL, Juarez Orozco LE, van Roon AM, Kamphuisen PW, Smit AJ, et al. Effect of linagliptin on pulse wave velocity in early type 2 diabetes: A randomized, double-blind, controlled 26-week trial (RELEASE). *Diabetes Obes Metab* 2017; **19**(8): 1147-54.
192. Dei Cas A, Micheli MM, Aldigeri R, Gardini S, Ferrari-Pellegrini F, Perini M, et al. Long-acting exenatide does not prevent cognitive decline in mild cognitive impairment: a proof-of-concept clinical trial. *Journal of endocrinological investigation* 2024; **47**(9): 2339-49.
193. Dubourg J, Perrimond-Dauchy S, Felices M, Bolze S, Voiriot P, Fouqueray P. Absence of QTc prolongation in a thorough QT study with imeglimin, a first in class oral agent for type 2 diabetes mellitus. *Eur J Clin Pharmacol* 2020; **76**(10): 1393-400.
194. Dubourg J, Ueki K, Grouin JM, Fouqueray P. Efficacy and safety of imeglimin in Japanese patients with type 2 diabetes: A 24-week, randomized, double-blind, placebo-controlled, dose-ranging phase 2b trial. *Diabetes Obes Metab* 2021; **23**(3): 800-10.
195. Dubourg J, Fouqueray P, Thang C, Grouin JM, Ueki K. Efficacy and Safety of Imeglimin Monotherapy Versus Placebo in Japanese Patients With Type 2 Diabetes (TIMES 1): A Double-Blind, Randomized, Placebo-Controlled, Parallel-Group, Multicenter Phase 3 Trial. *Diabetes Care* 2021; **44**(4): 952-9.
196. Ejiri K, Miyoshi T, Kihara H, Hata Y, Nagano T, Takaishi A, et al. Effect of Luseogliflozin on Heart Failure With Preserved Ejection Fraction in Patients With Diabetes Mellitus. *J Am Heart Assoc* 2020; **9**(16): e015103.
197. Fonseca V, Schweizer A, Albrecht D, Baron MA, Chang I, Dejager S. Addition of vildagliptin to insulin improves glycaemic control in type 2 diabetes. *Diabetologia* 2007; **50**(6): 1148-55.
198. Fouqueray P, Pirags V, Inzucchi SE, Bailey CJ, Schernthaner G, Diamant M, et al. The efficacy and safety of imeglimin as add-on therapy in patients with type 2 diabetes inadequately controlled with metformin monotherapy. *Diabetes Care* 2013; **36**(3): 565-8.
199. Fouqueray P, Pirags V, Diamant M, Schernthaner G, Lebovitz HE, Inzucchi SE, et al. The efficacy and safety of imeglimin as add-on therapy in patients with type 2 diabetes inadequately controlled with sitagliptin monotherapy. *Diabetes Care* 2014; **37**(7): 1924-30.
200. Garber AJ, Schweizer A, Baron MA, Rochotte E, Dejager S. Vildagliptin in combination with pioglitazone improves glycaemic control in patients with type 2 diabetes failing thiazolidinedione monotherapy: a randomized, placebo-controlled study. *Diabetes Obes Metab* 2007; **9**(2):

166-74.
201. Garber AJ, Foley JE, Banerji MA, Ebeling P, Gudbjornsdottir S, Camisasca RP, et al. Effects of vildagliptin on glucose control in patients with type 2 diabetes inadequately controlled with a sulphonylurea. *Diabetes Obes Metab* 2008; **10**(11): 1047-56.
202. Gu T, Ma J, Zhang Q, Zhu L, Zhang H, Xu L, et al. Comparative effect of saxagliptin and glimepiride with a composite endpoint of adequate glycaemic control without hypoglycaemia and without weight gain in patients uncontrolled with metformin therapy: Results from the SPECIFY study, a 48-week, multi-centre, randomized, controlled trial. *Diabetes Obes Metab* 2019; **21**(4): 939-48.
203. Hagi K, Kochi K, Watada H, Kaku K, Ueki K. Effect of patient characteristics on the efficacy and safety of imeglimin monotherapy in Japanese patients with type 2 diabetes mellitus: A post-hoc analysis of two randomized, placebo-controlled trials. *J Diabetes Investig* 2023; **14**(9): 1101-9.
204. Hagi K, Kochi K, Watada H, Kaku K, Ueki K. Factors contributing to the clinical effectiveness of imeglimin monotherapy in Japanese patients with type 2 diabetes mellitus. *J Diabetes Investig* 2024; **15**(9): 1239-47.
205. Hagi K, Kochi K, Watada H, Kaku K, Ueki K. Differences in imeglimin response in subgroups of patients with type 2 diabetes stratified by data-driven cluster analysis: A post-hoc analysis of imeglimin clinical trial data. *Diabetes Obes Metab* 2024; **26**(9): 3732-42.
206. Hanefeld M, Herman GA, Wu M, Mickel C, Sanchez M, Stein PP, et al. Once-daily sitagliptin, a dipeptidyl peptidase-4 inhibitor, for the treatment of patients with type 2 diabetes. *Curr Med Res Opin* 2007; **23**(6): 1329-39.
207. Hao Z, Zhang Y. Different Doses of Empagliflozin in Patients with Heart Failure with Reduced Ejection Fraction. *Int Heart J* 2022; **63**(5): 852-6.
208. Henry RR, Smith SR, Schwartz SL, Mudaliar SR, Deacon CF, Holst JJ, et al. Effects of saxagliptin on beta-cell stimulation and insulin secretion in patients with type 2 diabetes. *Diabetes Obes Metab* 2011; **13**(9): 850-8.
209. Hermansen K, Kipnes M, Luo E, Fanurik D, Khatami H, Stein P, et al. Efficacy and safety of the dipeptidyl peptidase-4 inhibitor, sitagliptin, in patients with type 2 diabetes mellitus inadequately controlled on glimepiride alone or on glimepiride and metformin. *Diabetes Obes Metab* 2007; **9**(5): 733-45.
210. Hollander P, Li J, Allen E, Chen R, Investigators CV. Saxagliptin added to a thiazolidinedione improves glycemic control in patients with type

2 diabetes and inadequate control on thiazolidinedione alone. *J Clin Endocrinol Metab* 2009; **94**(12): 4810-9.
211. Ito J, Hagi K, Kochi K, Ueki K, Watada H, Kaku K. Gastrointestinal symptoms in patients receiving imeglimin in combination with metformin: A post-hoc analysis of imeglimin clinical trial data. *J Diabetes Investig* 2025; **16**(4): 629-38.
212. Ji L, Li L, Kuang J, Yang T, Kim DJ, Kadir AA, et al. Efficacy and safety of fixed-dose combination therapy, alogliptin plus metformin, in Asian patients with type 2 diabetes: A phase 3 trial. *Diabetes Obes Metab* 2017; **19**(5): 754-8.
213. Kaku K, Shimoda M, Osonoi T, Iwamoto M, Kaneto H. Efficacy and safety of imeglimin add-on to DPP-4 inhibitor therapy in Japanese patients with type 2 diabetes mellitus: An interim analysis of the randomised, double-blind FAMILIAR trial. *Diabetes Obes Metab* 2025; **27**(6): 3212-22.
214. Kanazawa I, Tanaka KI, Notsu M, Tanaka S, Kiyohara N, Koike S, et al. Long-term efficacy and safety of vildagliptin add-on therapy in type 2 diabetes mellitus with insulin treatment. *Diabetes Res Clin Pract* 2017; **123**: 9-17.
215. Katsuno T, Shiraiwa T, Iwasaki S, Park H, Watanabe N, Kaneko S, et al. Benefit of Early Add-on of Linagliptin to Insulin in Japanese Patients With Type 2 Diabetes Mellitus: Randomized-Controlled Open-Label Trial (TRUST2). *Adv Ther* 2021; **38**(3): 1514-35.
216. Kikuchi M, Abe N, Kato M, Terao S, Mimori N, Tachibana H. Vildagliptin dose-dependently improves glycemic control in Japanese patients with type 2 diabetes mellitus. *Diabetes Res Clin Pract* 2009; **83**(2): 233-40.
217. Kikuchi M, Haneda M, Koya D, Tobe K, Onishi Y, Couturier A, et al. Efficacy and tolerability of vildagliptin as an add-on to glimepiride in Japanese patients with Type 2 diabetes mellitus. *Diabetes Res Clin Pract* 2010; **89**(3): 216-23.
218. Kim MK, Rhee EJ, Han KA, Woo AC, Lee MK, Ku BJ, et al. Efficacy and safety of teneligliptin, a dipeptidyl peptidase-4 inhibitor, combined with metformin in Korean patients with type 2 diabetes mellitus: a 16-week, randomized, double-blind, placebo-controlled phase III trial. *Diabetes Obes Metab* 2015; **17**(3): 309-12.
219. Kothny W, Shao Q, Groop PH, Lukashevich V. One-year safety, tolerability and efficacy of vildagliptin in patients with type 2 diabetes and moderate or severe renal impairment. *Diabetes Obes Metab* 2012; **14**(11): 1032-9.
220. Kothny W, Foley J, Kozlovski P, Shao Q, Gallwitz B, Lukashevich V. Improved glycaemic control with vildagliptin added to insulin, with or without metformin, in patients with type 2 diabetes mellitus. *Diabetes Obes Metab* 2013; **15**(3): 252-7.

221. Lee MMY, Brooksbank KJM, Wetherall K, Mangion K, Roditi G, Campbell RT, et al. Effect of Empagliflozin on Left Ventricular Volumes in Patients With Type 2 Diabetes, or Prediabetes, and Heart Failure With Reduced Ejection Fraction (SUGAR-DM-HF). *Circulation* 2021; **143**(6): 516-25.
222. Lukashevich V, Del Prato S, Araga M, Kothny W. Efficacy and safety of vildagliptin in patients with type 2 diabetes mellitus inadequately controlled with dual combination of metformin and sulphonylurea. *Diabetes Obes Metab* 2014; **16**(5): 403-9.
223. Macauley M, Hollingsworth KG, Smith FE, Thelwall PE, Al-Mrabeh A, Schweizer A, et al. Effect of vildagliptin on hepatic steatosis. *J Clin Endocrinol Metab* 2015; **100**(4): 1578-85.
224. McGill JB, Sloan L, Newman J, Patel S, Sauce C, von Eynatten M, et al. Long-term efficacy and safety of linagliptin in patients with type 2 diabetes and severe renal impairment: a 1-year, randomized, double-blind, placebo-controlled study. *Diabetes Care* 2013; **36**(2): 237-44.
225. McMurray JJV, Ponikowski P, Bolli GB, Lukashevich V, Kozlovski P, Kothny W, et al. Effects of Vildagliptin on Ventricular Function in Patients With Type 2 Diabetes Mellitus and Heart Failure: A Randomized Placebo-Controlled Trial. *JACC Heart Fail* 2018; **6**(1): 8-17.
226. Mita T, Katakami N, Yoshii H, Onuma T, Kaneto H, Osonoi T, et al. Alogliptin, a Dipeptidyl Peptidase 4 Inhibitor, Prevents the Progression of Carotid Atherosclerosis in Patients With Type 2 Diabetes: The Study of Preventive Effects of Alogliptin on Diabetic Atherosclerosis (SPEAD-A). *Diabetes Care* 2016; **39**(1): 139-48.
227. Mita T, Katakami N, Shiraiwa T, Yoshii H, Onuma T, Kuribayashi N, et al. Sitagliptin Attenuates the Progression of Carotid Intima-Media Thickening in Insulin-Treated Patients With Type 2 Diabetes: The Sitagliptin Preventive Study of Intima-Media Thickness Evaluation (SPIKE): A Randomized Controlled Trial. *Diabetes Care* 2016; **39**(3): 455-64.
228. Neves JS, Vasques-Novoa F, Borges-Canha M, Leite AR, Sharma A, Carvalho D, et al. Risk of adverse events with liraglutide in heart failure with reduced ejection fraction: A post hoc analysis of the FIGHT trial. *Diabetes Obes Metab* 2023; **25**(1): 189-97.
229. Nielsen R, Jorsal A, Tougaard RS, Rasmussen JJ, Schou M, Videbaek L, et al. The impact of the glucagon-like peptide-1 receptor agonist liraglutide on natriuretic peptides in heart failure patients with reduced ejection fraction with and without type 2 diabetes. *Diabetes Obes Metab* 2020; **22**(11): 2141-50.
230. Ning G, Wang W, Li L, Ma J, Lv X, Yang M, et al. Vildagliptin as add-on therapy to insulin improves glycemic control without increasing risk

of hypoglycemia in Asian, predominantly Chinese, patients with type 2 diabetes mellitus. *J Diabetes* 2016; **8**(3): 345-53.
231. Nomoto H, Takahashi A, Nakamura A, Kurihara H, Takeuchi J, Nagai S, et al. Add-on imeglimin versus metformin dose escalation regarding glycemic control in patients with type 2 diabetes treated with a dipeptidyl peptidase-4 inhibitor plus low-dose metformin: study protocol for a multicenter, prospective, randomized, open-label, parallel-group comparison study (MEGMI study). *BMJ Open Diabetes Res Care* 2022; **10**(6).
232. Nordisk N. A Research Study to Look Into How Well Semaglutide Medicine Works at Different Doses in People With Type 2 Diabetes and Overweight. 2022. https://clinicaltrials.gov/study/NCT05486065?cond=NCT05486065&rank=1 (accessed 2025/05/20 2025).
233. Odawara M, Hamada I, Suzuki M. Efficacy and Safety of Vildagliptin as Add-on to Metformin in Japanese Patients with Type 2 Diabetes Mellitus. *Diabetes Ther* 2014; **5**(1): 169-81.
234. Olansky L, Reasner C, Seck TL, Williams-Herman DE, Chen M, Terranella L, et al. A treatment strategy implementing combination therapy with sitagliptin and metformin results in superior glycaemic control versus metformin monotherapy due to a low rate of addition of antihyperglycaemic agents. *Diabetes Obes Metab* 2011; **13**(9): 841-9.
235. Oyanagi T, Kawanabe S, Tsukiyama H, Nishine A, Nakamura Y, Nakagawa T, et al. The Effects of Imeglimin on Muscle Strength in Patients with Type 2 Diabetes: A Prospective Cohort Study. *Diabetes Ther* 2024; **15**(11): 2323-36.
236. Pacini G, Mari A, Fouqueray P, Bolze S, Roden M. Imeglimin increases glucose-dependent insulin secretion and improves beta-cell function in patients with type 2 diabetes. *Diabetes Obes Metab* 2015; **17**(6): 541-5.
237. Palau P, Amiguet M, Dominguez E, Sastre C, Mollar A, Seller J, et al. Short-term effects of dapagliflozin on maximal functional capacity in heart failure with reduced ejection fraction (DAPA-VO(2)): a randomized clinical trial. *Eur J Heart Fail* 2022; **24**(10): 1816-26.
238. Peng XV, Marcinak JF, Raanan MG, Cao C. Combining the G-protein-coupled receptor 40 agonist fasiglifam with sitagliptin improves glycaemic control in patients with type 2 diabetes with or without metformin: A randomized, 12-week trial. *Diabetes Obes Metab* 2017; **19**(8): 1127-34.
239. Pirags V, Lebovitz H, Fouqueray P. Imeglimin, a novel glimin oral antidiabetic, exhibits a good efficacy and safety profile in type 2 diabetic patients. *Diabetes Obes Metab* 2012; **14**(9): 852-8.
240. Raz I, Hanefeld M, Xu L, Caria C, Williams-Herman D, Khatami H, et al. Efficacy and safety of the dipeptidyl peptidase-4 inhibitor sitagliptin

as monotherapy in patients with type 2 diabetes mellitus. *Diabetologia* 2006; **49**(11): 2564-71.
241. Reilhac C, Dubourg J, Thang C, Grouin JM, Fouqueray P, Watada H. Efficacy and safety of imeglimin add-on to insulin monotherapy in Japanese patients with type 2 diabetes (TIMES 3): A randomized, double-blind, placebo-controlled phase 3 trial with a 36-week open-label extension period. *Diabetes Obes Metab* 2022; **24**(5): 838-48.
242. Rosenstock J, Foley JE, Rendell M, Landin-Olsson M, Holst JJ, Deacon CF, et al. Effects of the dipeptidyl peptidase-IV inhibitor vildagliptin on incretin hormones, islet function, and postprandial glycemia in subjects with impaired glucose tolerance. *Diabetes Care* 2008; **31**(1): 30-5.
243. Rosenstock J, Sankoh S, List JF. Glucose-lowering activity of the dipeptidyl peptidase-4 inhibitor saxagliptin in drug-naive patients with type 2 diabetes. *Diabetes Obes Metab* 2008; **10**(5): 376-86.
244. Saito D, Kanazawa A, Shigihara N, Sato F, Uchida T, Sato J, et al. Efficacy and Safety of Vildagliptin as an Add-On Therapy in Inadequately Controlled Type 2 Diabetes Patients Treated With Basal Insulin. *J Clin Med Res* 2017; **9**(3): 193-9.
245. Sanyal AJ, Kaplan LM, Frias JP, Brouwers B, Wu Q, Thomas MK, et al. Triple hormone receptor agonist retatrutide for metabolic dysfunction-associated steatotic liver disease: a randomized phase 2a trial. *Nat Med* 2024; **30**(7): 2037-48.
246. Scherbaum WA, Schweizer A, Mari A, Nilsson PM, Lalanne G, Jauffret S, et al. Efficacy and tolerability of vildagliptin in drug-naive patients with type 2 diabetes and mild hyperglycaemia*. *Diabetes Obes Metab* 2008; **10**(8): 675-82.
247. Strain WD, Lukashevich V, Kothny W, Hoellinger MJ, Paldanius PM. Individualised treatment targets for elderly patients with type 2 diabetes using vildagliptin add-on or lone therapy (INTERVAL): a 24 week, randomised, double-blind, placebo-controlled study. *Lancet* 2013; **382**(9890): 409-16.
248. Takahashi A, Nomoto H, Onishi K, Manda S, Miya A, Kameda H, et al. A comparative study of the effects of imeglimin add-on or metformin dose escalation on glycaemic variability in subjects with type 2 diabetes treated with low-dose metformin (MEGMI-CGM study). *Diabetes Obes Metab* 2024; **26**(8): 3471-4.
249. Takahashi A, Nomoto H, Yokoyama H, Yokozeki K, Furusawa S, Oe Y, et al. Efficacy of imeglimin treatment versus metformin dose escalation on glycemic control in subjects with type 2 diabetes treated with a dipeptidyl peptidase-4 inhibitor plus low-dose metformin: A multicenter, prospective, randomized, open-label, parallel-group comparison study (MEGMI study). *Diabetes Obes Metab* 2025; **27**(3): 1466-76.

250. Theurey P, Thang C, Pirags V, Mari A, Pacini G, Bolze S, et al. Phase 2 trial with imeglimin in patients with Type 2 diabetes indicates effects on insulin secretion and sensitivity. *Endocrinol Diabetes Metab* 2022; **5**(6): e371.
251. Usui R, Hamamoto Y, Imura M, Omori Y, Yamazaki Y, Kuwata H, et al. Differential effects of imeglimin and metformin on insulin and incretin secretion-An exploratory randomized controlled trial. *Diabetes Obes Metab* 2025; **27**(2): 856-65.
252. Yang W, Xing X, Lv X, Li Y, Ma J, Yuan G, et al. Vildagliptin added to sulfonylurea improves glycemic control without hypoglycemia and weight gain in Chinese patients with type 2 diabetes mellitus. *J Diabetes* 2015; **7**(2): 174-81.
253. Yang W, Zhu D, Gan S, Dong X, Su J, Li W, et al. Dorzagliatin add-on therapy to metformin in patients with type 2 diabetes: a randomized, double-blind, placebo-controlled phase 3 trial. *Nat Med* 2022; **28**(5): 974-81.
254. Younis A, Eskenazi D, Goldkorn R, Leor J, Naftali-Shani N, Fisman EZ, et al. The addition of vildagliptin to metformin prevents the elevation of interleukin 1ss in patients with type 2 diabetes and coronary artery disease: a prospective, randomized, open-label study. *Cardiovasc Diabetol* 2017; **16**(1): 69.
255. Zhu D, Li X, Ma J, Zeng J, Gan S, Dong X, et al. Dorzagliatin in drug-naive patients with type 2 diabetes: a randomized, double-blind, placebo-controlled phase 3 trial. *Nat Med* 2022; **28**(5): 965-73.
256. Zhu XX, Zhu DL, Li XY, Li YL, Jin XW, Hu TX, et al. Dorzagliatin (HMS5552), a novel dual-acting glucokinase activator, improves glycaemic control and pancreatic beta-cell function in patients with type 2 diabetes: A 28-day treatment study using biomarker-guided patient selection. *Diabetes Obes Metab* 2018; **20**(9): 2113-20.
257. Altintas Dogan AD, Hilberg O, Hess S, Jensen TT, Bladbjerg EM, Juhl CB. Respiratory Effects of Treatment with a Glucagon-Like Peptide-1 Receptor Agonist in Patients Suffering from Obesity and Chronic Obstructive Pulmonary Disease. *Int J Chron Obstruct Pulmon Dis* 2022; **17**: 405-14.
258. Gerards MC, Venema GE, Patberg KW, Kross M, Potter van Loon BJ, Hageman IMG, et al. Dapagliflozin for prednisone-induced hyperglycaemia in acute exacerbation of chronic obstructive pulmonary disease. *Diabetes Obes Metab* 2018; **20**(5): 1306-10.
